# Protein modification *via* alkyne hydrosilylation using a substoichiometric amount of ruthenium(ii) catalyst†
†Dedicated to Professor Stuart L. Schreiber on the occasion of his 60^th^ birthday.
‡
‡Electronic supplementary information (ESI) available. See DOI: 10.1039/c6sc05313k



**DOI:** 10.1039/c6sc05313k

**Published:** 2017-03-14

**Authors:** Terence T.-L. Kwan, Omar Boutureira, Elizabeth C. Frye, Stephen J. Walsh, Moni K. Gupta, Stephen Wallace, Yuteng Wu, Fengzhi Zhang, Hannah F. Sore, Warren R. J. D. Galloway, Jason W. Chin, Martin Welch, Gonçalo J. L. Bernardes, David R. Spring

**Affiliations:** a Department of Chemistry , University of Cambridge , Lensfield Rd , Cambridge CB2 1EW , UK . Email: spring@ch.cam.ac.uk; b Medical Research Council , Laboratory of Molecular Biology , Francis Crick Avenue, Cambridge Biomedical Campus , Cambridge CB2 0QH , UK; c School of Biological Sciences , University of Edinburgh , The King's Buildings , Edinburgh , EH9 3FF , UK; d Department of Biochemistry , University of Cambridge , Tennis Court Road , Cambridge CB2 1QW , UK; e Instituto de Medicina Molecular , Faculdade de Medicina , Universidade de Lisboa , Avenida Professor Egas Moniz , 1649-028 , Lisboa , Portugal

## Abstract

Transition metal catalysis has emerged as a powerful strategy to expand synthetic flexibility of protein modification. Herein, we report a cationic Ru(ii) system that enables the first example of alkyne hydrosilylation between dimethylarylsilanes and *O*-propargyl-functionalized proteins using a substoichiometric amount or low-loading of Ru(ii) catalyst to achieve the first C–Si bond formation on full-length substrates. The reaction proceeds under physiological conditions at a rate comparable to other widely used bioorthogonal reactions. Moreover, the resultant *gem*-disubstituted vinylsilane linkage can be further elaborated through thiol–ene coupling or fluoride-induced protodesilylation, demonstrating its utility in further rounds of targeted modifications.

## Abstract 2

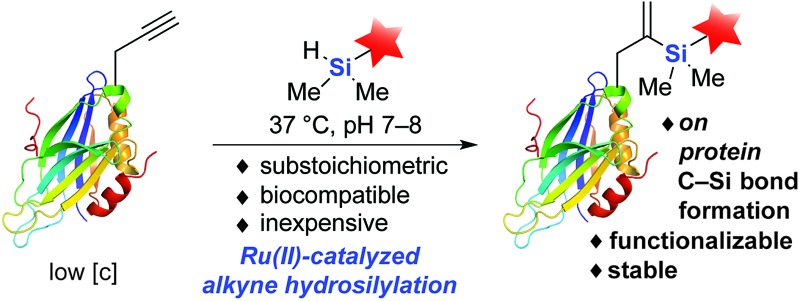
The development of site-specific modification of alkyne-functionalized proteins using dimethylarylsilanes and substoichiometric or low-loading of Ru(ii) catalysts is reported. Furthermore, the resultant gem-vinylsilane can undergo further targeted chemical modifications, highlighting its potential for single-site, dual-modification applications.

## Introduction

**Fig. 1:**
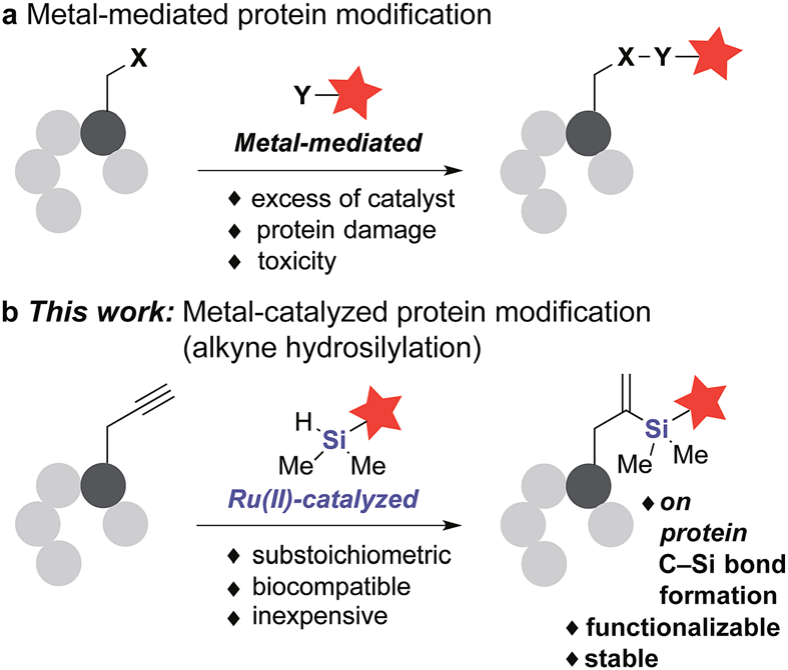
(a) General metal-mediated protein modification protocols using excess of metal catalysts and (b) our approach *via* Ru(ii)-catalyzed alkyne hydrosilylation.

The chemical modification of biomolecules has emerged as a powerful tool to study cellular systems.^1–3^ Alongside recombinant methods,^4–7^ advances in organic chemistry have fueled the development of an increasing number of chemical reactions capable of modifying proteins at both genetically and chemically predefined sites.^8–13^ These bioorthogonal reactions have transformed our ability to visualize cellular processes, and have enabled the development of new therapeutic strategies to treat diseases.^14–16^ Within this “toolbox” of bioorthogonal reactions, transition metal-mediated reactions are arguably the most underdeveloped.^17–20^ This is likely due to transition metals' propensity for unproductive chelation within the biological milieu, resulting in the need for high catalyst loadings to achieve acceptable reaction rates and labeling efficiency (Fig. 1a). Previous examples of transition metal-mediated reactions include Cu(i) azide–alkyne cycloaddition,^21,22^ Ru(ii) cross-metathesis,^23–26^ Pd(ii) cysteine bioconjugation,^27^ Suzuki–Miyaura^28,29^ and some of their intracellular variants.^30,31^ However, most published reaction conditions utilize high catalyst loading and the development of a truly catalytic transition metal-mediated bioconjugation strategy has received little attention.

Here we report a new Ru-catalyzed alkyne hydrosilylation reaction for protein modification. Using the water-soluble Ru catalyst [Cp*Ru(MeCN)_3_]PF_6_ (**1**) and dimethylaryl hydrosilane derivatives, this methodology enables the efficient labeling of multiple protein targets modified both stochastically and site-specifically with an alkyne-containing moiety (Fig. 1b). In addition, hydrosilylation has orthogonal chemical reactivity to ketone-hydrazine condensation reaction *in vitro*, and the resultant *gem*-disubstituted vinylsilane product can be further modified *via* thiol–ene coupling and fluoride-induced protodesilylation, demonstrating the potential of this methodology for use in both orthogonal dual labeling and single-site, multiple-probe imaging applications. To the best of our knowledge, this represents the first example of a C–Si bond formation on protein substrates using substoichiometric or low-loading of transition metal catalysts – a feature that we hope will reinstate this mode of catalysis as a viable avenue for future research in the field.

## Results and discussion

Although hydrosilylation has gained widespread utility in organic synthesis and in the industrial production of organosilicon compounds,^32–35^ aqueous alkyne hydrosilylation is largely underdeveloped. Inspired by the development of a cationic ruthenium catalyst [Cp*Ru(MeCN)_3_]PF_6_ **1** by Trost and Ball,^36,37^ we examined the catalyst's ability to catalyze hydrosilylation under biocompatible, aqueous conditions. We started our investigation by reacting 3,6,9,12-tetraoxapentadec-14-yne **2** as a model alkyne and a variety of water-soluble hydrosilanes with **1** (5 mol%). Despite previously reported reactivities of trialkoxy and trialkyl silanes, no vinylsilane products were observed under the reaction conditions tested (Table 1, entries 1–3).

**Table 1:** Optimisation of hydrosilylation conditions (alkoxy *vs.* alkyl/aryl hydrosilanes)

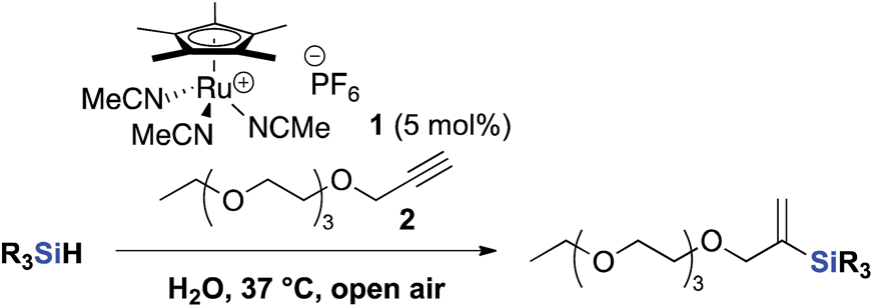			
Entry	Silane (R_3_SiH)	Conversion^*a*^ (%)	*t* (min)
1	(EtO)_3_SiH	NR	O/N
2	(TMSO)_3_SiH	NR	O/N
3	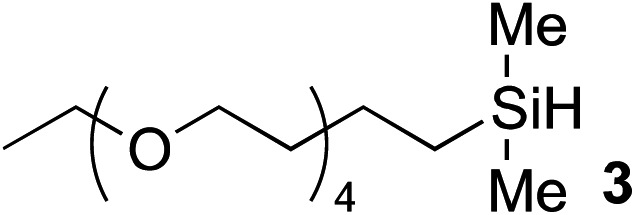	NR	O/N
4	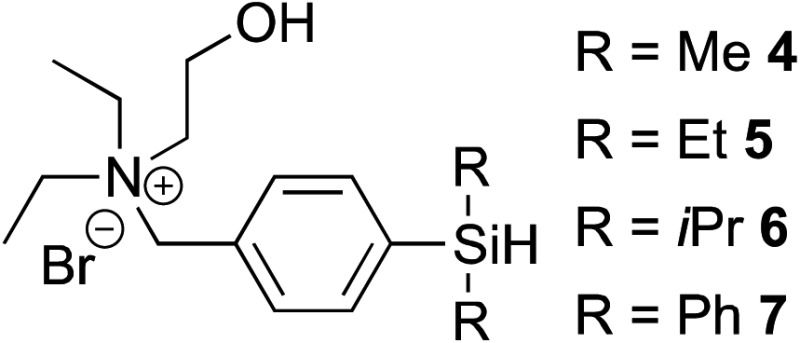	100 (92^*b*^)	<5
5		100	70
6		NR	O/N
7		24	80
8^*c*^	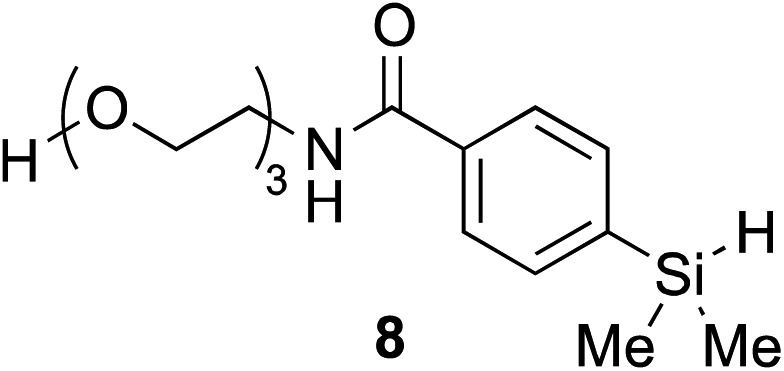	99^*b*^	30
9^*c*^ ^,^ ^*d*^ ^,^ ^*e*^		71^*b*^	30

^*a*^Determined by ^1^H-NMR using 4,4-dimethyl-4-silapentane-1-sulfonic acid (DSS) as an internal standard.

^*b*^Isolated yield.

^*c*^50% *t*-BuOH in PBS (pH 7.4).

^*d*^Reaction conducted in the presence of 10% human plasma.

^*e*^10 mol% hippuric acid (BzNHCH_2_CO_2_H) as an additive. O/N: overnight; NR: no reaction (>95% starting alkyne recovered).

Gratifyingly, hydrosilylation of dimethylaryl hydrosilane **4** with **2** proceeded smoothly (Table 1, entry 4), achieving full conversion with 92% isolated yield in less than 5 min. This apparent high reactivity may be attributed to the strong affinity for Cp*Ru complexes to coordinate with aromatic rings,^37^ allowing hydrosilylation to proceed rapidly in aqueous solution and open air, thus reinforcing the use of aryldialkyl hydrosilanes in further experiments. The reaction proceeded with a 2^nd^ order rate constant *k*_2_ ∼ 1.0 M^–1^ s^–1^ (see ESI, Fig. S20‡), which is comparable to Ru(ii) cross-metathesis and strain-promoted alkyne–azide cycloadditions. Furthermore, **4** was found to be stable in buffered conditions at neutral pH, with a half-life (*t*_1/2_) > 1 week (see ESI, Fig. S21‡).

One of the side reactions of aqueous hydrosilylation is the hydrolysis of hydrosilane to form silanol (Si–OH). In an effort to reduce silanol formation, we installed substituents adjacent to the Si–H bond with varying degree of steric hindrance (compounds **5–7**) in the hope to increase selectivity for hydrosilylation over silanol formation. However, none of the tested analogues gave better selectivity or reaction rates (Table 1, entries 5–7). In particular, **6** and **7** showed incomplete conversion despite prolonged reaction times (Table 1, entries 6 and 7).

The hydrophobicity of chemical probes and modifications often require the use of organic co-solvents in the reaction mixture. Alcohol-based solvents were found to be tolerated as co-solvents in aqueous hydrosilylation and achieved similar reaction rates to those using pure water (see ESI, Fig. S22‡). Thus, the hydrosilylation of **2** with triethylene glycol hydrosilane derivative **8** in 50% *t*-BuOH in phosphate buffered saline (PBS) at pH 7.4 gave the corresponding vinylsilane in 99% isolated yield (Table 1, entry 8). In the presence of human plasma, the reaction initially proceeded extremely slowly and gave only trace of product. It was suspected that **1** is inactive in hydrosilylation due to nonproductive chelation to ruthenium in 10% human plasma. Remarkably, the addition of hippuric acid (BzNHCH_2_CO_2_H) as an additive/ligand helped to stabilize the Ru(ii) complex from rapid exchange processes with, for example, histidine^38,39^ and aspartic acid^40^ residues in plasma protein and restored the activity of **1**, with the corresponding vinylsilane product isolated in a good yield (Table 1, entry 9). This result demonstrates that our novel hydrosilylation methodology for protein modification can proceed under physiological conditions.

The scope of this reaction was further evaluated using a variety of small molecule alkynes representative of amino acids, carbohydrates, and hydrophobic drugs such as alkynes **9**, **11–13** that may be considered substructural motifs of 3-*O*-methyl-DOPA (3-OMD), which is one of the most important metabolites of l-DOPA. We first investigated whether nearby chalcogens on the terminal alkyne group could increase the rate of hydrosilylation. With no nearby coordinating groups, the reaction with alkyne **9** proceeded slowly, requiring a long reaction time to reach 68% yield (Table 2, entry 1). Contrary to the reported chalcogen effect in protein cross-metathesis,^23,26^ *S*-propargyl **11** and *Se*-propargyl **12** inhibited hydrosilylation and the respective vinylsilane products were not detected, despite extended reaction times (Table 2, entries 2 and 3). Surprisingly, *O*-propargyl **13** showed the most promise, affording vinylsilane **14** in 91% isolated yield (Table 2, entry 4). This is likely due to the intricate balance between ruthenium-coordination (X = O) and inhibition (X = S, Se).

**Table 2:** Alkyne scope for aqueous hydrosilylation

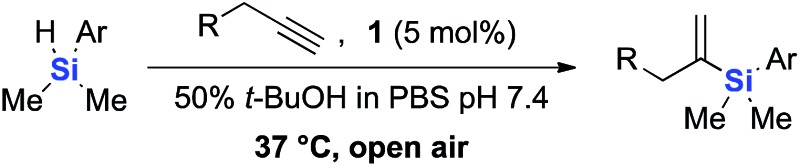			
Entry	Silane (ArMe_2_SiH)	Alkyne	Product^*a*^ (yield%)
1^*b*^	**8**	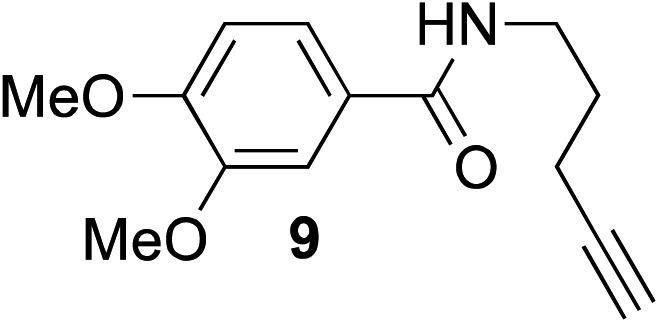	**10** (68%)
2	**8**	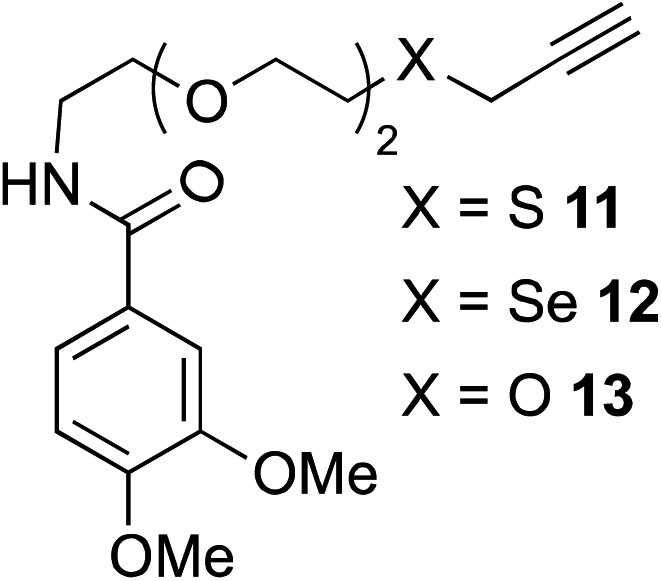	NR
3	**8**		NR
4	**8**		**14** (91%)
5^*c*^	PhMe_2_SiH (**15**)	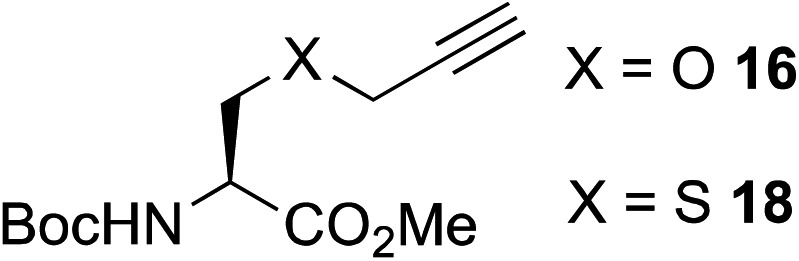	**17** (98%)
6^*c*^	**15**		**19** (29%)
7^*c*^	**15**	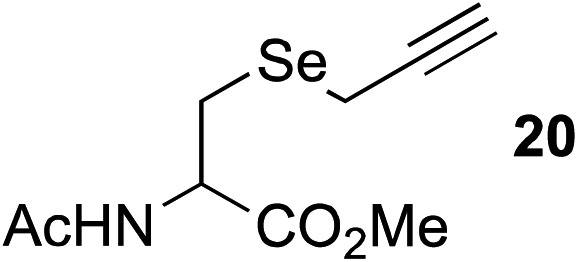	NR
8^*c*^	**8**	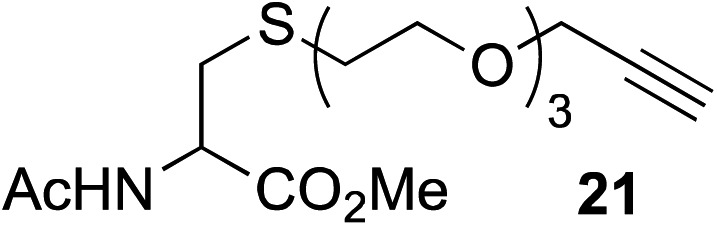	**22** (85%)
9^*c*^	**8**	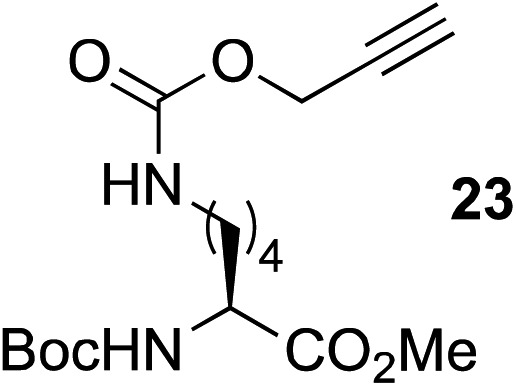	**24** (81%)
10	**8**	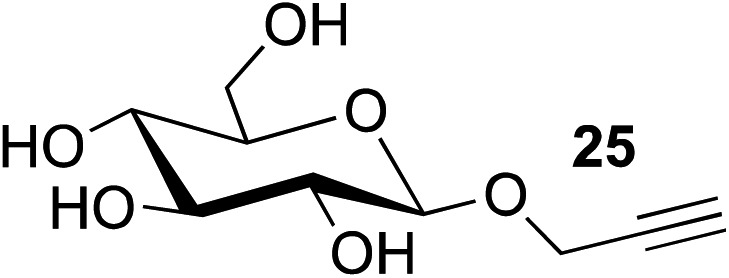	**26** (82%)

^*a*^Isolated yield.

^*b*^5 h reaction time.

^*c*^10 mol% hippuric acid added as an additive. NR: no reaction (>95% starting alkyne recovered).

**Scheme 1:**
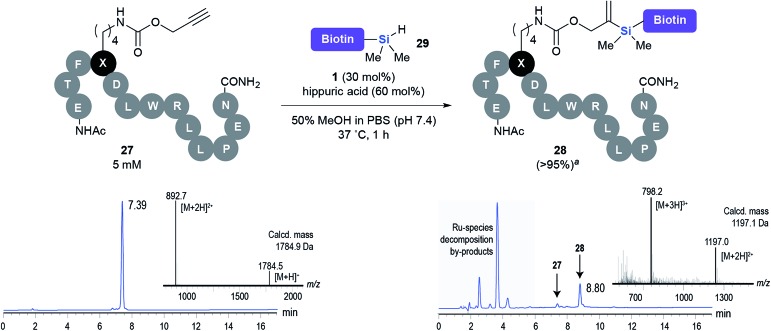
Hydrosilylation of peptide **27** with biotinylated hydrosilane **29**. ^*a*^Conversion (%) determined by HPLC.

This observation was further confirmed by the decreasing isolated yields when reacting PhMe_2_SiH **15** with *O*-propargyl-serine **16**, *S*-propargyl-cysteine **18**, and *Se*-propargyl-selenocysteine **20** derivatives (Table 2, entries 5–7). Nonetheless, hydrosilylation proceeded smoothly on amino acids **21** and **23**, as well as alkyne-sugar derivative **25**, affording the corresponding products in excellent isolated yields (Table 2, entries 8–10). These examples are of particular importance, as strategies for the *in vivo* incorporation of such moieties into proteins and cell-surface glycans have been developed.^41–43^ Full conversion was also achieved on a model peptide **27** with biotinylated hydrosilane **29**, demonstrating the potential for Ru(ii) aqueous hydrosilylation protocol to modify more complex biomolecules (Scheme 1). Furthermore, the stability of the resulting *gem*-disubstituted vinylsilane moiety was assessed under physiological conditions and in the presence of biological thiols, with no observable degradation at 37 °C for up to 24 h (see ESI, Fig. S24‡).

**Scheme 2:**
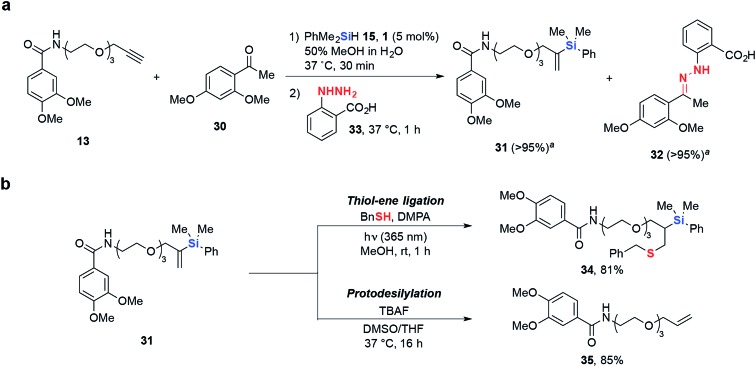
(a) Tandem hydrosilylation and hydrazine condensation reaction. (b) *gem*-Disubstituted vinylsilane reactivity under thiol–ene coupling conditions and fluoride-induced protodesilylation, giving thioether **34** and *O*-allyl **35** in 81% and 85% isolated yields, respectively. ^*a*^Conversion (%) determined by HPLC.

Biocompatible chemical transformations are often most powerful when used in conjunction with each other, allowing for the site-specific incorporation of multiple chemical modifications into a single biomolecule.^44^ Encouragingly, hydrosilylation was found to be compatible with the widely used α-substituted amine/carbonyl condensation,^45–48^ where *O*-propargyl **13** reacted smoothly with hydrosilane **15** in the presence of ketone **30**, achieving >95% conversion to desired vinylsilane **31** in 30 min. Subsequent addition of hydrazine **33** resulted in complete conversion of **30** to hydrazone **32**. This demonstrates the utility of tandem hydrosilylation and condensation reactions *via* a step-wise, one-pot ligation strategy without any undesired interference to their reactivities (Scheme 2a).

Most site-selective dual-labeling efforts require the incorporation of two unique bioorthogonal functional groups^49,50^ or the use of bifunctional substrates,^51,52^ which can be a synthetic challenge and limits the wide adoption of such methods. Moreover, it would be advantageous to have the ability to selectively remove synthetically incorporated chemical modifications, allowing for potential “switch on/off” applications. To address these issues, we sought to further elaborate the *gem*-disubstituted vinylsilane linkage *via* radical thiol–ene^53–55^ and fluoride-induced protodesilylation reactions^56,57^ (Scheme 2b). To illustrate the dual-labeling methodology, we incubated model vinylsilane substrate **31** with benzyl mercaptan, 10 mol% 2,2-dimethoxy-2-phenylacetophenone (DMPA) and irradiated at 365 nm to give doubly-modified derivative **34** in excellent isolated yield (81%). Furthermore, the *gem*-disubstituted vinylsilane linkage can be cleaved by treatment with TBAF to give the corresponding *O*-allyl **35**, demonstrating the potential for chemical Si-modifications installed *via* hydrosilylation to be selectively removed.

**Fig. 2:**
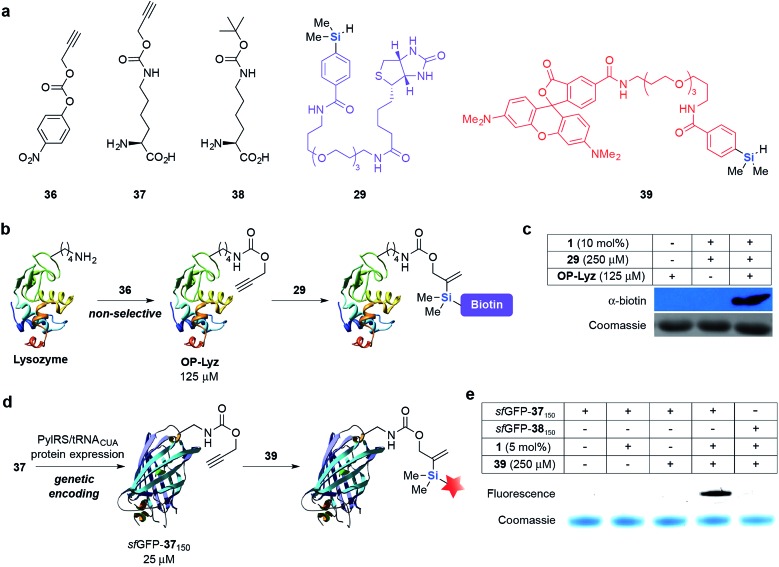
Selective labeling of *O*-propargyl (OP) modified protein substrates *via* hydrosilylation. (a) The structures of unnatural amino acids **37** and **38** and other reagents used in this study. (b) Modification of solvent-exposed lysine residues on lysozyme (Lyz) with **36** and subsequent labeling with **29**. (c) Selective labeling of OP-Lyz *via* hydrosilylation with **29** and **1**. Lyz (–) and OP-Lyz (+) (125 μM) was independently incubated with **29** (250 μM) and **1** (10 mol%) for 2 h at 37 °C and the presence of biotinylated protein was detected by Western blot using α-biotin-HRP conjugated antibody. (d) Genetic encoding and fluorescent labeling of **37** *via* hydrosilylation. (e) In-gel fluorescence demonstrating specific labeling of *sf*GFP-**37**_150_ with **39**. In (c) and (e), equal protein loading was verified by Coomassie staining.

With these promising results in hand, we conducted protein-labeling experiments *via* hydrosilylation on different protein systems. First, lysine residues on lysozyme (Lyz) were non-selectively modified with **36** to give *O*-propargyl modified Lyz (OP-Lyz) (Fig. 2a and b). When treated with biotinylated hydrosilane **29** and only 10 mol% of **1**, we were pleased to observe selective labeling of OP-Lyz over Lyz with negligible background labeling (Fig. 2c). Similarly, when the reaction time or concentration of **29** was held constant (1 h and 250 μM, respectively), dose- and time-dependent labeling was observed, even at very low catalyst loading (2 mol%) (see ESI, Fig. S1‡). Inductively coupled plasma-mass spectrometry (ICP-MS) determined that ruthenium content was <10 ppb after purification when using 10 mol% catalyst (see ESI‡ for details). To the best of our knowledge this result is the first demonstration of a protein modification protocol mediated by a substoichiometric amount of transition metal catalyst.

Next, we incorporated *O*-propargyl groups site-specifically into a super-folder GFP (*sf*GFP) protein (Fig. 2d).^58^ Briefly, PylRS/pylT pair, the wild-type orthogonal *Methanosarcina barkeri* pyrrolysyl-*t*RNA synthetase and *t*RNA_CUA_ pair and C-terminally hexahistidine-tagged *sf*GFP containing an amber codon (TAG) at position 150 (*sf*GFP_150TAG_His_6_) were introduced into *E. coli*. Addition of **37** (5 mM) led to the amino acid dependent synthesis of full-length *sf*GFP-**37**_150_ in good yield (15 mg L^–1^ of culture). A similar approach was used to obtain *sf*GFP-**38**_150_ as a negative control for labeling experiments (Fig. S2 and S3‡). We subsequently incubated *sf*GFP-**37**_150_ with fluorescent hydrosilane **39** and **1** (5 mol%) in PBS (pH 7.4) at 37 °C. A fluorescent band was detected after 24 h of incubation with limited background fluorescence observed. This result is particularly noteworthy because no fluorescence was observed when *sf*GFP-**38**_150_ was reacted under the same conditions, highlighting the bioorthogonality and specificity of this reaction towards *O*-propargyl groups (Fig. 2e). The formation of the expected ligated protein was further confirmed by LC-MS (see ESI, Fig. S4‡).

**Scheme 3:**
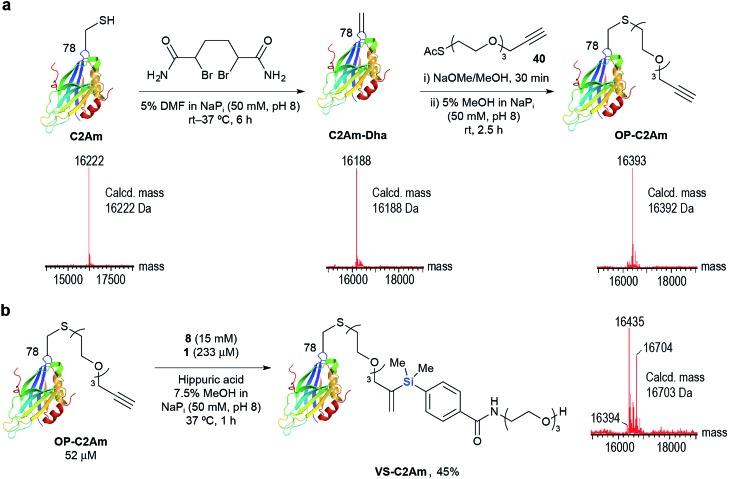
(a) Site-specific incorporation of **40** into C2Am *via* C2Am-Dha and (b) subsequent hydrosilylation with **8**. Found masses corresponding to OP-C2Am (16 394 Da), Oxidized-OP-C2Am (16 435 Da), and VS-C2Am (16 704 Da).

As an alternative to recombinant techniques, we also site-specifically incorporated the alkyne handle through chemical modifications at cysteine. Using the methodology developed by Davis and co-workers,^59^ the single cysteine mutant of the C2A domain of Synaptotagmin I C2Am (eukaryotic marker of apoptosis) was converted to OP-C2Am *via* the dehydroalanine-tagged protein intermediate in >95% conversion (Scheme 3a). Gratifyingly, **1** successfully mediated hydrosilylation of OP-C2Am with **8** at 37 °C for 1 h to afford VS-C2Am as detected by LC-MS (Scheme 3b). Compared to the high loading of transition metal complexes used in typical metal-mediated protein modification protocols, Ru(ii) complex **1** mediated aqueous hydrosilylation offers a milder alternative and these results highlight its potential for site-specific chemical protein modification using either substoichiometric or low-catalyst loading systems.

Having succinctly demonstrated the ability to carry out alkyne hydrosilylation on numerous protein systems, efforts were then directed towards ascertaining whether it was possible to modify the protein-incorporated vinylsilane through our earlier described radical thiol–ene and protodesilylation reactions. Vinylsilane-modified lysozyme (VS-Lyz) was chosen for initial studies. We found that treatment of VS-Lyz with a protected cysteine amino acid and DMPA under *hν* irradiation yielded Cys-Lyz, as detected by LC-MS (see ESI, Fig. S18‡). Similarly, treatment of VS-Lyz with TBAF·3H_2_O afforded Ene-Lyz (see ESI, Fig. S19‡). These proof-of-principle experiments show that the vinylsilane can be further modified after its introduction on a protein through Ru(ii)-catalyzed aqueous hydrosilylation.

## Conclusions

In conclusion, we have demonstrated that *O*-propargyl groups and dimethylaryl hydrosilanes (HSiMe_2_Ar) are effective coupling partners for Ru(ii) complex **1** catalyzed aqueous hydrosilylation, where alkyne-labeled small-molecules and peptides are site-specifically modified in good to excellent yields. Furthermore, hydrosilylation is shown to have orthogonal reactivity to the widely used bioorthogonal hydrazine condensation reaction, giving rise to potential biomolecule dual-labeling applications. Furthermore, *O*-propargyl tagged proteins (*via* chemical and genetic strategies) successfully undergo site-specific hydrosilylation in the presence of substoichiometric or low loading of **1** to achieve the first C–Si bond on protein substrates. Finally, the resultant *gem*-disubstituted vinylsilane linkage serves as a reactive chemical handle for thiol–ene coupling and protodesilylation, highlighting the potential for single-site dual-modification and the selective removal of vinylsilane modifications. Hence, we believe this work greatly expands the reaction conditions and substrate complexity of hydrosilylation and complements the growing interest in metal-mediated protein modification strategies.
