# Supplementary material for: Protein modification via alkyne hydrosilylation using a substoichiometric amount of ruthenium(ii) catalyst
Source: Chem Sci. 2017 Mar 14;8(5):3871–8. doi: 10.1039/c6sc05313k (PMC5578368; doi:10.1039/c6sc05313k)

Electronic Supplementary Material (ESI) for Chemical Science.
This journal is © The Royal Society of Chemistry 2017

Protein Modification via Alkyne Hydrosilylation Using A Substoichiometric Amount of Ruthenium(II) Catalyst

Terence T.-L. Kwan,[a] Omar Boutureira,[a] Elizabeth C. Frye,[a] Stephen J. Walsh,[a] Moni K. Gupta,[a] Stephen Wallace,[bc] Yuteng Wu,[a] Fengzhi Zhang,[a] Hannah F. Sore,[a] Jason W. Chin,[b] Martin Welch,[d] Gonçalo J. L. Bernardes[ae] and David R. Spring*[a]

[a.] *Department of Chemistry, University of Cambridge, Lensfield Rd, Cambridge CB2 1EW (UK). Email: spring@ch.cam.ac.uk*
[b.] *Medical Research Council, Laboratory of Molecular Biology, Francis Crick Avenue, Cambridge Biomedical Campus, Cambridge CB2 0QH (UK).*
[c.] *School of Biological Sciences, University of Edinburgh, The King's Buildings, Edinburgh, EH9 3FF (UK).*
[d.] *Department of Biochemistry, University of Cambridge, Tennis Court Road, Cambridge CB2 1QW (UK).*
[e.] *Instituto de Medicina Molecular, Faculdade de Medicina, Universidade de Lisboa, Avenida Professor Egas Moniz, 1649-028, Lisboa, Portugal.*

Table of Contents

1. General considerations S2
2. Synthetic procedures S5
   2.1. Hydrosilane substrates S5
   2.2. Alkyne substrates S15
   2.3. Vinylsilane products S24
   2.4. Synthesis and hydrosilylation of peptide **27** S33
3. Protein modification S36
   3.1. Preparation and hydrosilylation of **OP-Lyz** S36
   3.2. *sf*GFP protein expression and purification S37
   3.3. Hydrosilylation of *sf*GFP-$\mathbf{37}_{150}$ S39
   3.4. Preparation and hydrosilylation of **SP-C2Am** and **OP-C2Am** S40
   3.5. Modification of **VS-Lyz** S51
4. Kinetic analysis of aqueous hydrosilylation S55
5. Stability of hydrosilane S56
6. Organic co-solvent effect on aqueous alkyne hydrosilylation S57
7. Compatibility study with carbonyl condensation S58
8. Stability of vinylsilane **26** S61
9. Thiol-ene and protodesilylation of model vinylsilane **31** S62
10. References S64
11. NMR spectra S65

1. General considerations

All non-aqueous reactions were carried out under nitrogen or argon with dry and freshly distilled solvents using oven-dried glassware unless otherwise stated. Room temperature (rt) refers to ambient temperature. A temperature of 0 ºC was maintained using ice-water bath. A temperature of –78 ºC was maintained using an acetone-dry ice bath. All reagents and solvents were used as obtained from commercial sources unless otherwise stated. Tetrahydrofuran (THF) was dried over Na wire and distilled from a mixture of calcium hydride and lithium aluminium hydride with triphenylmethane as indicator. Dichloromethane, methanol, acetonitrile, and ethyl acetate were distilled from calcium hydride. Diethyl ether was distilled from a combination of calcium hydride and lithium aluminium hydride. Petroleum ether refers to the distilled fraction between 40–60 ºC. Pd/C refers to palladium on activated charcoal (10% Pd basis).

Nuclear magnetic resonance spectra (NMR) were recorded using an internal deuterium lock at ambient probe temperatures (unless otherwise stated) on Bruker DPX-400, Bruker Avance DRX-400, Bruker Avance 500 BB-ATM and Bruker Avance 500 Cryo Ultrashield spectrometers. ^{1}H-NMR and ^{13}C-NMR spectra assignments are supported by DEPT-135 editing, COSY (2D, ^{1}H–^{1}H correlations), HSQC (2D, one bond ^{1}H–^{13}C correlations), HMBC (2D, multi-bonds ^{1}H–^{13}C correlations), or by analogy to fully interpreted spectra of related compounds.

In proton magnetic resonance spectra (^{1}H-NMR): chemical shifts (δ) are quoted in ppm to the nearest 0.01 ppm and are referenced to the residual non-deuterated solvent peak ($CDCl_3$: 7.26, DMSO-d_6: 2.50, CD_3OD: 3.31); coupling constants (*J*) are reported in Hz to the nearest 0.1 Hz. Data are reported as follows: chemical shift, integration, multiplicity [br, broad; s, singlet; d, doublet; t, triplet; q, quartet; quint, quintet; spt, septet; m, multiplet; or as a combination of these (*e.g.* dd, dt, etc.)], coupling constant(s) and assignment. Diastereotopic protons are assigned as H-a and H-b, where the H-a indicates the lower field proton. In carbon magnetic resonance spectra (^{13}C-NMR): chemical shifts (δ) are quoted in ppm to the nearest 0.1 ppm and are referenced to the

deuterated solvent peak ($CDCl_3$: 77.2, DMSO-d_6: 39.5, CD_3OD: 49.0). Data are reported as follow: chemical shift and assignment.

TLC was carried out on glass plates pre-coated with Merck silica gel 60 F254, visualised by quenching of UV fluorescence (λ_{max} = 254 nm) or by staining with potassium permanganate. Retention factors (R_f) are quoted to 0.01. Flash column chromatography (FCC) was carried out using slurry-packed Merck 9385 Kieselgel 60 silica gel (230–400 mesh) under a positive pressure of nitrogen.

Melting points (m.p.) were obtained using a Büchi Melting Point B-545 melting point apparatus and are uncorrected.

Optical rotations were recorded on an Anton-Paar MCP 100 polarimeter. $[\alpha]_D^{20}$ values are reported in 10^{-1} deg·cm^2·g^{-1} at 598 nm, concentration (*c*) is given in g·(100 mL)$^{-1}$.

Infrared (IR) spectra were recorded on a Perkin-Elmer Spectrum One (FT–IR) spectrometer with internal referencing as neat films. Selected absorption maxima (ν_{max}) are reported in wavenumbers (cm^{-1}) and the following abbreviations are used: w, weak; m, medium; str, strong; br, broad.

Analytical high performance liquid chromatography (HPLC) was run on an Agilent 1260 Infinity machine, using a Supelcosil™ ABZ+PLUS column (150 mm × 4.6 mm, 3 μm) with a linear gradient system (solvent A: 0.05% (v/v) TFA in water, solvent B: 0.05% (v/v) TFA in acetonitrile) over 15 min at a flow rate of 1 mL·min^{-1}, and UV detection (λ_{max} = 220 and 254 nm). Retention times (t_r) are reported to the nearest 0.01 min.

Preparative high performance liquid chromatography (HPLC) was run on an Agilent 1260 Infinity machine, using a Supelcosil™ ABZ+PLUS column (250 mm × 21.2 mm, 5 μm) with a linear gradient system (solvent A: 0.1% (v/v) TFA in water, solvent B: 0.05% (v/v) TFA in acetonitrile) over 20 min at a flow rate of 20 mL·min^{-1}, visualised by UV absorbance (λ_{max} = 220 or 254 nm).

Low-resolution mass spectra (LRMS) were recorded using liquid chromatography and mass spectroscopy (LC–MS). Only molecular ions are reported. ESI refers to electron spray ionisation technique. LC–MS System: Waters ACQUITY H-Class UPLC with an ESCi Multi-Mode Ionisation Waters SQ Detector 2 spectrometer using MassLynx 4.1 software; LC system: solvent: 2 mM NH_4OAc in water/acetonitrile (95:5); solvent B: acetonitrile; solvent C: 2% formic acid; column: ACQUITY UPLC CSH C18 (2.1 mm × 50 mm, 1.7 µm, 130 Å) at 40 ºC; gradient: 5–95% B with constant 5% C over 1 min at flow rate of 0.6 $mL \cdot min^{-1}$; detector: PDA eλ Detector 220–800 nm, interval 1.2 nm.

High-resolution mass spectra (HRMS) were obtained with a Micromass Q-TOF mass spectrometer or a Waters LCT Premier Time of Flight mass spectrometer. Reported mass values are within the error limits of ± 5 ppm mass units. Only molecular ions are reported. ESI refers to the electrospray ionisation technique.

Protein Mass Spectrometry

*sf*GFP substrates

Using an Agilent 1200 LC-MS system, ESI–MS was carried out with a 6130 Quadrupole spectrometer. The solvent system consisted of 0.2% formic acid in H_2O as buffer A, and 0.2% formic acid in acetonitrile (MeCN) as buffer B. LC–ESI–MS on proteins was carried out using a Phenomenex Jupiter C4 column (150 × 2 mm, 5 µm) and samples were analysed in the positive mode, following protein UV absorbance at 214 and 280 nm. Total protein masses were calculated by deconvolution within the MS Chemstation software (Agilent Technologies).

C2Am substrates

LC–MS was performed on a Xevo G2-S TOF mass spectrometer coupled to an Acquity UPLC system using an Acquity UPLC BEH300 C4 column (1.7 µm, 2.1 × 50 mm). Water with 0.1% formic acid (solvent A) and 70% acetonitrile and 29% water with 0.075% formic acid (solvent B), were used as the mobile phase at a flow rate of 0.2 mL min^{-1}. The gradient was programmed as follows: from 72% A to 100% B for 25 min then 100% B for 2 minutes and 72% A for 18 min. The electrospray source was operated with a capillary voltage of 2.0 kV and a cone voltage of 40 V. Nitrogen was used as the desolvation gas at a total flow of 850 $L \cdot h^{-1}$. Total mass spectra were

reconstructed from the ion series using the MaxEnt algorithm preinstalled on MassLynx software (v. 4.1 from Waters) according to the manufacturer's instructions.

2. Synthetic procedures

2.1. Hydrosilane substrates

N-(4-Bromobenzyl)-N-ethylethanamine (S1)

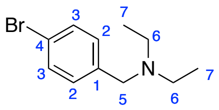

4-Bromobenzylbromide (13.8 g, 55.0 mmol) was added to a solution of DIPEA (13.1 mL, 75.2 mmol) and diethylamine (5.20 mL, 50.3 mmol) in MeCN (100 mL) and stirred at rt for 3 h. The solvent was removed *in vacuo*, the residue re-dissolved in CH_2Cl_2 (50 mL) and washed with H_2O (50 mL). The layers were separated and the aqueous layer was extracted with CH_2Cl_2 (3 × 100 mL). The combined organic layers were washed with brine (100 mL), dried ($MgSO_4$), concentrated *in vacuo* and purified by FCC (1:4 Et_2O:PE) to give **S1** as a pale yellow liquid (6.40 g, 53%). $\boldsymbol{R_f}$ 0.22 (1:4 Et_2O:PE); $\boldsymbol{\nu}_{max}$ (neat)/cm^{-1} 2968, 2797, 1591, 1487, 1166, 1069, 1011, 768; $\boldsymbol{\delta}_{\mathbf{H}}$ (400 MHz, $CDCl_3$) 7.42 (2H, d, J = 8.4 Hz, 2 × H3), 7.22 (2H, d, J = 8.5 Hz, 2 × H2), 3.50 (2H, s, H5), 2.50 (4H, q, J = 7.1 Hz, 2 × H6), 1.03 (6H, t, J = 7.1 Hz, H7); $\boldsymbol{\delta}_{\mathbf{C}}$ (101 MHz, $CDCl_3$) 139.3 (C4), 131.3 (2 × C2), 130.6 (2 × C3), 120.4 (C1), 57.0 (C5), 46.8 (C6), 11.9 (C7); **HRMS** (ESI) *m/z* found $[M+H]^+$ 242.0555, $C_{11}H_{17}N^{79}Br^+$ required 242.0544. This data is consistent with that previously reported.[1]

General synthetic procedure 1 (GSP 1) for the synthesis of N-(4-(alkylsilyl)benzyl)-N-ethylethan amine (S2–S5)

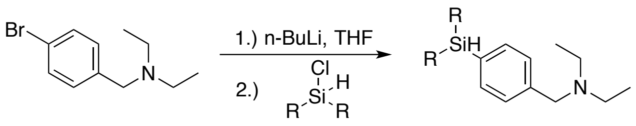

Tertiary amine **S1** (1 equiv.) was dissolved in tetrahydrofuran and cooled to –78 ºC. *n*-BuLi (1 equiv.) was added portion-wise and the mixture was stirred for 30 min at –78 ºC. The corresponding chlorosilane (1.1 equiv.) was added drop-wise and stirred at –78 ºC for 30 min, and stirred at rt for a further 1 h. The reaction was quenched with sat.

NH_4Cl solution and extracted with CH_2Cl_2. The combined organic layers were dried ($MgSO_4$), concentrated *in vacuo* and purified by flash column chromatography.

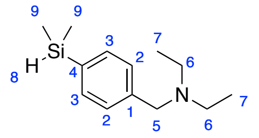

***N*-(4-(Dimethylsilyl)benzyl)-*N*-ethylethanamine (S2)** (Pale yellow oil, 3.90 g, 89%): **S1** (4.80 g, 20.0 mmol), *n*-BuLi (13.3 mL, 20.0 mmol), chlorodimethylsilane (2.20 g, 22.0 mmol) and THF (50 mL) were reacted according to GSP 1 and purified by FCC (1:19 $MeOH:CH_2Cl_2$). $\boldsymbol{R_f}$ 0.38 (1:9 $MeOH:CH_2Cl_2$); $\boldsymbol{\nu_{max}}$ (neat)/cm^{-1} 2967, 2800, 2117, 1601, 1384, 1249, 1110, 877; $\boldsymbol{\delta_H}$ (500 MHz, $CDCl_3$) 7.49 (2H, d, J = 7.9 Hz, 2 × H3), 7.34 (2H, d, J = 7.9 Hz, 2 × H2), 4.42 (1H, spt, J = 3.7 Hz, H8), 3.57 (2H, s, H5), 2.53 (4H, q, J = 7.1 Hz, 2 × H6), 1.05 (6H, t, J = 7.1 Hz, 2 × H7), 0.34 (6H, d, J = 3.7 Hz, 2 × H9); $\boldsymbol{\delta_C}$ (126 MHz, $CDCl_3$) 141.1 (C1), 135.6 (C4), 134.1 (2 × C3), 128.6 (2 × C2), 57.6 (C5), 46.9 (2 × C6), 11.8 (C7), –3.6 (2 × C9); **HRMS** (ESI) *m/z* found $[M+H]^+$ 222.1665, $C_{13}H_{24}N^{28}Si^+$ required 222.1673.

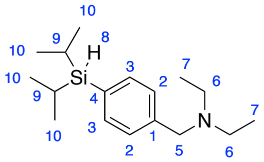

***N*-(4-(Diisopropylsilyl)benzyl)-*N*-ethylethanamine (S3)** (Yellow oil, 2.1 g, 97%): **S1** (1.9 g, 7.9 mmol), *n*-BuLi (4.9 mL, 7.9 mmol), diisopropylchlorosilane (1.5 mL, 8.7 mmol) and THF (50 mL) were reacted according to GSP 1 and purified by FCC (1:49 to 2:23 $MeOH:CH_2Cl_2$). $\boldsymbol{R_f}$ 0.40 (2:23 $MeOH:CH_2Cl_2$); $\boldsymbol{\nu}_{max}$ (neat)/cm^{-1} 2940, 2801, 2100, 1601, 1462; $\boldsymbol{\delta_H}$ (400 MHz, $CDCl_3$) 7.46 (2H, d, J = 8.0 Hz, 2 × H3), 7.32 (2H, d, J = 8.0 Hz, 2 × H2), 3.95 (1H, t, J = 3.1 Hz, H8), 3.58 (2H, s, H5), 2.54 (4H, q, J = 7.1 Hz, 2 × H6), 1.24 (2H, d spt, J = 3.1, 7.3 Hz, 2 × H9), 1.08 (6H, d, J = 7.2, 2 × H10), 1.06 (6H, t, J = 7.1 Hz, 2 × H7), 1.00 (6H, d, J = 7.3 Hz, 2 × H10'); $\boldsymbol{\delta}_C$ (101 MHz, $CDCl_3$) 140.9 (C1), 135.5 (2 × C2), 132.2 (C4), 128.4 (2 × C3), 57.6 (C5), 46.9 (2 × C6), 18.8 (2 × C10), 18.7 (2 × C10'), 11.8 (2 × C7), 10.9 (C × C9); **HRMS** (ESI) *m/z* found $[M+H]^+$ 278.2304, $C_{17}H_{32}N^{28}Si^+$ required 278.2299.

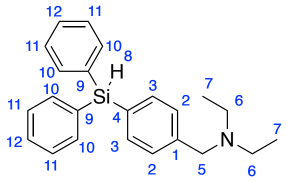

***N*-(4-(Diphenylsilyl)benzyl)-*N*-ethylethanamine (S4)** (Yellow oil, 0.62 g, 67%): **S1** (0.65 g, 2.7 mmol), *n*-BuLi (1.8 mL, 2.7 mmol), diphenylchlorosilane (0.57 mL, 3.0 mmol) and THF (50 mL) were reacted according to GSP 1 and purified by FCC (1:49 to 3:47 $MeOH:CH_2Cl_2$). $\boldsymbol{R_f}$ 0.40 (2:25 $MeOH:CH_2Cl_2$); $\boldsymbol{\nu_{max}}$ (neat)/cm^{-1} 3068, 2968, 2932, 2801, 2121, 1600, 1428, 1384; $\boldsymbol{\delta_H}$ (500 MHz, $CDCl_3$) 7.38–7.62 (14H, m, 2

× H2, 2 × H3, 4 × H10, 4 × H11, 2 × H12), 5.50 (1H, s, H8), 3.60 (2H, s, H5), 2.55 (4H, q, J = 7.1, 2 × H6), 1.07 (6H, t, J = 7.1 Hz, 2 × H7); δ_C (126 MHz, $CDCl_3$) 142.0, 135.9, 135.8, 133.7, 131.3, 129.9, 128.7, 128.1, 57.6 (C5), 46.9 (2 × C6), 11.9 (2 × C7); **HRMS** (ESI) *m/z* found $[M+H]^+$ 346.1971, $C_{23}H_{28}N^{28}Si^+$ required 346.1986.

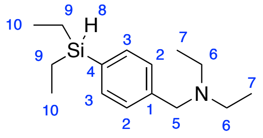

***N*-(4-(Diethylsilyl)benzyl)-*N*-ethylethanamine** (**S5**) (Yellow oil, 1.30 g, 55%): **S1** (2.30 g, 9.50 mmol), *n*-BuLi (6.30 mL, 9.50 mmol), diethylchlorosilane (1.30 g, 10.4 mmol) and THF (50 mL) were reacted according to GSP 1 and purified by FCC (1:49 to 3:47 MeOH:CH_2Cl_2). R_f 0.60 (1:9 MeOH:CH_2Cl_2); ν_{max} (neat)/cm^{-1} 2958, 2936, 2874, 2802, 2104, 1601, 1457, 1383; δ_H (400 MHz, $CDCl_3$) 7.48 (2H, d, J = 7.9 Hz, 2 × H3), 7.34 (2H, d, J = 7.9 Hz, 2 × H2), 4.21 (1H, p, J = 3.3 Hz, H8), 3.58 (2H, s, H5), 2.54 (4H, q, J = 7.1 Hz, 2 × H6), 1.06 (6H, t, J = 7.1 Hz, 2 × H7), 1.03 (6H, t, J = 7.8, 2 × H10), 0.81–0.88 (4H, m, 2 × H9); δ_C (101 MHz, $CDCl_3$) 140.9 (C1), 134.7 (2 × C3), 133.7 (C4), 128.5 (2 × C2), 57.6 (C5), 46.8 (2 × C6), 11.8 (2 × C7), 8.3 (2 × C10), 3.6 (2 × C9); **HRMS** (ESI) *m/z* found $[M+H]^+$ 250.1980, $C_{15}H_{28}N^{28}Si^+$ required 250.1986.

GSP 2 for the synthesis of *N*-(4-(dialkylsilyl)benzyl)-*N*,*N*-diethyl-2-hydroxyethanammonium bromide (4–7)

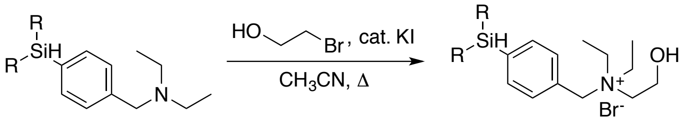

The corresponding amine (1 equiv.), 2-bromoethanol (5 equiv.), potassium iodide (0.05 equiv.) and molecular sieves (4 Å MS) were dissolved in anhydrous acetonitrile and refluxed for 4–6 days. Upon completion, the mixture was filtered through a small layer of SiO_2, washed with acetonitrile, concentrated *in vacuo* and purified by FCC.

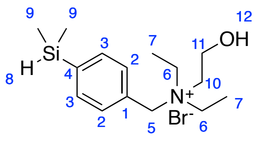

***N*-(4-(Dimethylsilyl)benzyl)-*N*,*N*-diethyl-2-hydroxyethanammonium bromide (4)** (White gum, 2.41 g, 74%): **S2** (2.10 g, 9.40 mmol), 2-bromoethanol (3.50 mL, 50.0 mmol), potassium iodide (80.0 mg, 0.500 mmol) and MeCN (50 mL) were reacted according to GSP 2 and purified by FCC (1:19 MeOH:CH_2Cl_2). R_f 0.21 (1:9 MeOH:CH_2Cl_2); ν_{max} (neat)/cm^{-1} 3282, 2959, 2123, 1623, 1457; δ_H (500 MHz, $CDCl_3$)

7.57 (2H, d, J = 8.1 Hz, 2 × H2), 7.52 (2H, d, J = 8.1 Hz, 2 × H3), 4.74 (2H, s, H5), 4.39 (1H, spt, J = 3.8 Hz, H8), 4.21 (2H, t, J = 4.6 Hz, H11), 4.00 (1H, br s, H12), 3.41–3.52 (6H, m, 2 × H6, H10), 1.47 (6H, t, J = 7.2 Hz, 2 × H7), 0.32 (6H, d, J = 3.8 Hz, 2 × H9); δ_C (126 MHz, $CDCl_3$) 141.3 (C1), 135.1 (2 × C2), 132.1 (2 × C3), 127.7 (C4), 62.5 (C5), 59.0 (C10), 55.6 (C11), 54.0 (2 × C6), 8.8 (2 × C7), –3.9 (2 × C9); **HRMS** (ESI) m/z found $[M–Br]^+$ 266.1922, $C_{15}H_{28}NO^{28}Si^+$ required 266.1935.

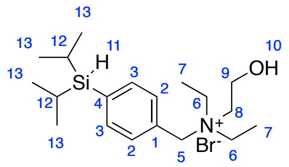

***N*-(4-(Diisopropylsilyl)benzyl)-*N*,*N*-diethyl-2-hydroxyethanammonium bromide (6)** (White solid, 1.21 g, 83%): **S3** (1.00 g, 3.61 mmol), 2-bromoethanol (1.02 mL, 14.4 mmol), potassium iodide (40.0 mg, 0.240 mmol) and MeCN (20 mL) were reacted according to GSP 2 and purified by FCC (1:49 to 1:4 $MeOH:CH_2Cl_2$). R_f 0.23 (1:9 $MeOH:CH_2Cl_2$); **m.p.** = 122–123.5 ºC; ν_{max} (neat)/cm^{-1} 3276, 2942, 2863, 2111, 1625, 1460, 1397, 1213; δ_H (500 MHz, $CDCl_3$) 7.60 (2H, d, J = 8.1 Hz, 2 × H2), 7.53 (2H, d, J = 8.1 Hz, 2 × H3), 5.27 (1H, t, J = 5.7 Hz, H10), 4.75 (2H, s, H5), 4.26–4.27 (2H, m, H9), 3.95 (1H, t, J = 3.2 Hz, H11), 3.46–3.58 (6H, m, 2 × H6, H8), 1.50 (6H, t, J = 7.2 Hz, 2 × H7), 1.24 (2H, d spt, J = 3.2, 7.4 Hz, 2 × H12), 1.06 (6H, d, J = 7.4 Hz, 2 × H13), 0.97 (6H, d, J = 7.4 Hz, 2 × H13'); δ_C (126 MHz, $CDCl_3$) 138.6 (C1), 136.6 (2 × C2), 131.9 (2 × C3), 127.5 (C4), 62.7 (C5), 59.1 (C8), 55.7 (C9), 54.1 (2 × C6), 18.7 (2 × C13), 18.6 (2 × C13'), 10.7 (2 × C12), 8.8 (2 × C7); **HRMS** (ESI) m/z found $[M–Br]^+$ 322.2567, $C_{19}H_{36}ON^{28}Si^+$ required 322.2561.

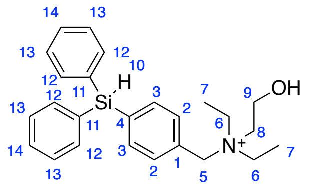

***N*-(4-(Diphenylsilyl)benzyl)-*N*,*N*-diethyl-2-hydroxyethanammonium bromide (7)** (White sticky solid, 1.47 g, 82%): **S4** (1.32 g, 3.82 mmol), 2-bromoethanol (1.10 mL, 15.3 mmol), potassium iodide (40.0 mg, 0.24 mmol) and MeCN (20 mL) were reacted according to GSP 2 and purified by FCC (1:49 to 1:4 $MeOH:CH_2Cl_2$). R_f 0.19 (1:9 $MeOH:CH_2Cl_2$); ν_{max} (neat)/cm^{-1} 3384, 2980, 2125, 1600, 1500, 1429, 1385; δ_H (500 MHz, CD_3OD) 7.71 (2H, d, J = 8.1 Hz, 2 × H3), 7.63 (2H, d, J = 8.1 Hz, 2 × H2), 7.55–7.57 (4H, m, 4 × H12), 7.44–7.47 (2H, m, 2 × H14), 7.38–7.41 (4H, m, 4 × H13), 5.45 (1H, s, H10), 4.66 (2H, s, H5), 4.06 (2H, t, J = 4.9 Hz, H9), 3.35–3.41 (6H, m, 2 × H6, H8), 1.45 (6H, t, J = 7.2 Hz, 2 × H7); δ_C (126 MHz, CD_3OD) 138.4 (C1), 137.5 (2 × C3), 136.7 (4 × C12), 133.7 (C4), 133.6 (2 × C2), 131.3

(2 × C14), 130.5 (2 × C1), 129.4 (4 × C13), 63.0 (C5), 59.6 (C8), 56.6 (C9), 55.0 (2 × C6), 8.4 (2 × C7); **HRMS** (ESI) *m/z* found [M–Br]$^+$ 390.2233, $C_{25}H_{32}ON^{28}Si^+$ required 390.2248.

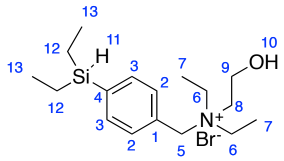

***N*-(4-(Diethylsilyl)benzyl)-*N*,*N*-diethyl-2-hydroxyethanammonium bromide (5)** (White sticky solid, 0.71 g, 28%): **S5** (1.7 g, 6.8 mmol), 2-bromoethanol (2.0 mL, 27 mmol), potassium iodide (88 mg, 0.53 mmol) and MeCN (50 mL) were reacted according to GSP 2 and purified by FCC (1:49 to 1:4 MeOH:CH_2Cl_2). $\boldsymbol{R_f}$ 0.26 (1:9 MeOH:CH_2Cl_2); $\boldsymbol{\nu_{max}}$ (neat)/cm^{-1} 3290, 2954, 2875, 2107, 1625, 1457, 1399; $\boldsymbol{\delta_H}$ (500 MHz, $CDCl_3$) 7.59 (2H, d, *J* = 8.0 Hz, 2 × H3), 7.53 (2H, d, *J* = 8.1 Hz, 2 × H2), 5.34 (1H, t, *J* = 5.8 Hz, H10), 4.76 (2H, s, H5), 4.25 (2H, br s, H9), 4.20 (1H, p, *J* = 3.3 Hz, H11), 3.44–3.57 (6H, m, 2 × H6, H8), 1.49 (6H, t, *J* = 7.2 Hz, 2 × H7), 1.00 (6H, t, *J* = 7.8 Hz, 2 × H13), 0.81–0.87 (4H, m, 2 × H12); $\boldsymbol{\delta_C}$ (126 MHz, $CDCl_3$) 139.8 (C1), 135.8 (2 × C3), 132.1 (2 × C2), 127.6 (C4), 62.6 (C5), 59.1 (C8), 55.7 (C9), 54.0 (2 × C6), 8.7 (2 × C7), 8.1 (2 × C13), 3.3 (2 × C12); **HRMS** (ESI) *m/z* found [M–Br]$^+$ 294.2237, $C_{17}H_{32}ON^{28}Si^+$ required 294.2248.

16-Bromo-3,6,9,12-tetraoxahexadecane (S6)

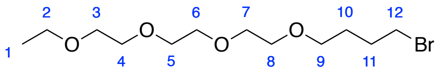

NaH (1.6 g, 40 mmol) was added portion wise to a stirring solution of 2-(2-(2-ethoxyethoxy)ethoxy)ethan-1-ol (7.0 mL, 40 mmol) in anhydrous THF (40 mL) at 0 ºC. The mixture was stirred until no effervescence was observed. 1,4-Dibromobutane (14 mL, 120 mmol) was subsequently added to the reaction and stirred at rt for 16 h. The reaction was then diluted with water (100 mL) and CH_2Cl_2 (50 mL). The layers were separated and the aqueous layer was extracted further with CH_2Cl_2. The combined organic layers were dried ($MgSO_4$), concentrated *in vacuo* and purified by FCC (3:97 to 1:4 EtOAc:petroleum ether) to give **S6** as a yellow liquid (2.90 g, 23%). $\boldsymbol{R_f}$ 0.24 (3:7 EtOAc:petroleum ether); $\boldsymbol{\nu_{max}}$ (neat)/cm^{-1} 2865, 1444, 1349, 1249; $\boldsymbol{\delta_H}$ (400 MHz, $CDCl_3$) 3.47–3.65 (16H, m, H2–H8, H12), 3.43 (2H, t, *J* = 6.8 Hz, H9), 1.94 (2H, quint, *J* = 7.1 Hz, H11), 1.72 (2H, p, *J* = 6.9 Hz, H10), 1.20 (3H, td, *J* = 7.0, 0.6 Hz, H1); $\boldsymbol{\delta_C}$ (101 MHz, $CDCl_3$) 70.8, 70.7, 70.4, 70.3, 69.9 (C2–C8, C12), 66.8 (C2), 33.9 (C9),

29.8 (C11), 28.3 (C10), 15.3 (C1); **HRMS** (ESI) *m/z* found $[M+H]^+$ 313.1010, $C_{12}H_{26}O_4{}^{79}Br^+$ required 313.1009.

2-Methyl-7,10,13,16-tetraoxa-2-silaoctadecane (3)

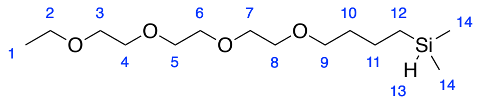

Turbo Grignard (9.20 mL, 11.9 mmol) was added to a stirring solution of **S6** (2.86 g, 9.12 mmol) in THF (20 mL) and refluxed for two days. Chlorodimethylsilane (1.10 mL, 10.0 mmol) was then added dropwise to this stirring solution and refluxed for a further three days. The reaction was subsequently quenched with water (50 mL) and extracted with CH_2Cl_2 (3 × 50 mL). The combined organic layers were dried ($MgSO_4$), concentrated *in vacuo* and purified by FCC (3:97 to 1:9 EtOAc:petroleum ether) to give **3** as a colourless liquid (2.00 g, 75%). $\boldsymbol{R_f}$ 0.35 (3:7 EtOAc:petroleum ether); $\boldsymbol{\nu}_{max}$ (neat)/cm^{-1} 2862, 2108, 1456, 1350, 1249; $\boldsymbol{\delta_H}$ (400 MHz, $CDCl_3$) 3.82 (1H, spt, *J* = 3.5 Hz, H13), 3.55–3.65 (12 H, m, 6 × OC$\underline{H}_2$), 3.51 (2H, qd, *J* = 7.0, 0.5 Hz, H2), 3.44 (2H, t, *J* = 6.6 Hz, H9), 1.60 (2H, p, *J* = 7.1 Hz, H10), 1.35–1.42 (2H, m, H11), 1.20 (3H, td, *J* = 7.0, 0.6 Hz, H1), 0.58 (2H, td, *J* = 8.2, 3.0 Hz, H12), 0.04 (6H, dd, *J* = 3.7, 0.7 Hz, 2 × H14); $\boldsymbol{\delta_C}$ (101 MHz, $CDCl_3$) 71.3, 70.8, 70.7, 70.2, 69.9 (C3–C9), 66.7 (C2), 33.1 (C10), 21.1 (C11), 15.3 (C1), 14.1 (C12), –4.4 (2 × C14); **HRMS** (ESI) *m/z* found $[M+H]^+$ 293.2138, $C_{14}H_{33}O_4{}^{28}Si^+$ required 293.2143.

4-(Dimethylsilyl)benzoic acid (S7)

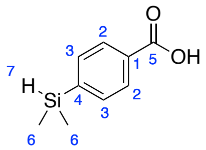

i-PrMgCl (2 M in THF, 22.2 mL, 44.4 mmol) was added to a stirring solution of 4-iodobenzoic acid (5.00 g, 20.2 mmol) in THF (150 mL) at –40 ºC. The mixture was stirred for 1.5 h at –40 ºC, and chlorodimethylsilane (2.46 mL, 22.2 mmol) was added. The resulting mixture was allowed to warm to rt and stirred 16 h. Upon completion, the reaction was quenched with 1 M aqueous HCl (100 mL) and extracted with Et_2O (3 ×

100 mL). The combined organic layers were washed with brine (50 mL), dried ($MgSO_4$), concentrated *in vacuo* and purified by FCC (1:99 to 3:97 MeOH:CH_2Cl_2) to give **S7** as a white solid (3.3 g, 91%). R_f 0.18 (1:19 MeOH:CH_2Cl_2); δ_H (500 MHz, $CDCl_3$) 8.08 (2H, d, *J* = 8.1 Hz, 2 × H2), 7.66 (2H, d, *J* = 8.1 Hz, 2 × H3), 4.47 (1H, spt, *J* = 3.8 Hz, H7), 0.38 (6H, d, *J* = 3.8, 2 × H6); δ_C (126 MHz, $CDCl_3$) 171.9 (C5), 145.0 (C4), 134.1 (2 × C3), 129.7 (C1), 129.1 (2 × C2), –4.0 (2 × C6); **HRMS** (ESI) *m/z* found $[M–H]^-$ 179.0531, $C_9H_{11}O_2{}^{28}Si^-$ required 179.0534. This data is consistent with that previously reported.[2]

4-(Dimethylsilyl)-*N*-(2-(2-(2-hydroxyethoxy)ethoxy)ethyl)benzamide (8)

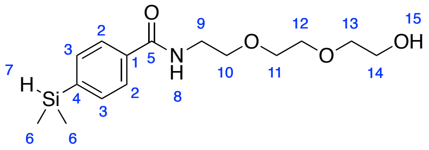

CDI (108 mg, 0.666 mmol) was added to a solution of **S7** (100 mg, 0.555 mmol) in CH_2Cl_2 (1.5 mL) and stirred for 30 min. 2-(2-(2-Aminoethoxy)ethoxy)ethan-1-ol (99.3 mg, 0.666 nmol) and TEA (93.0 µL, 0.666 nmol) in CH_2Cl_2 (1.5 mL) were subsequently added to the activated carboxylic acid mixture and stirred at rt for 16 h. Upon completion, the solvent was removed *in vacuo* and purified by FCC (3:97 to 1:24 MeOH:CH_2Cl_2) to give **8** as a pale yellow oil (152 mg, 84%). R_f 0.26 (1:20 MeOH:CH_2Cl_2); ν_{max} (neat)/cm^{-1} 3339, 2873, 2120, 1641, 1542, 1108, 881; δ_H (500 MHz, $CDCl_3$) 7.78 (2H, d, *J* = 8.2 Hz, 2 × H2), 7.61 (2H, d, *J* = 8.2 Hz, 2 × H3), 6.84 (1H, s, H8), 4.44 (1H, spt, *J* = 3.8 Hz, H7), 3.60–3.73 (12H, m, H9–H14), 2.56 (1H, t, *J* = 5.7 Hz, H15), 0.36 (6H, d, *J* = 3.8 Hz, 2 × H6); δ_C (126 MHz, $CDCl_3$) 167.5 (C5), 141.8 (C4), 135.0 (C1), 134.2 (2 × C2), 126.2 (2 × C3), 72.5 (one C from C9–C14), 70.4 (two C from C9–C14), 70.0 (one C from C9–C14), 61.8 (one C from C10–C14), 39.7 (C9), –4.0 (2 × C6); **HRMS** (ESI) *m/z* found $[M+H]^+$ 312.1617, $C_{15}H_{26}NO_4{}^{28}Si^+$ required 312.1631.

4-(Dimethylsilyl)-*N*-(15-oxo-19-((3a*S*,4*S*,6a*R*)-2-oxohexahydro-1*H*-thieno[3,4-*d*]imidazol-4-yl)-4,7,10-trioxa-14-azanonadecyl)benzamide (29)

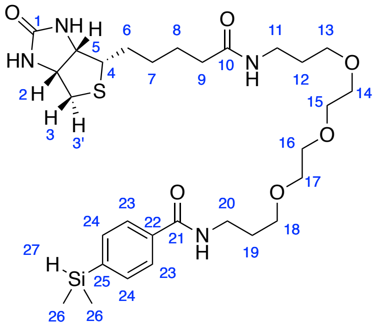

TFA (500 μL, 6.53 mmol) was added to a solution of *tert*-butyl (15-oxo-19-((3a*S*,4*S*,6a*R*)-2-oxohexahydro-1*H*-thieno[3,4-*d*]imidazol-4-yl)-4,7,10-trioxa-14-azanonadecyl)carbamate (50.0 mg, 0.091 mmol) in CH_2Cl_2 (1 mL) and stirred for 1 h at rt. The reaction mixture was concentrated *in vacuo* to give the corresponding amine. **S7** (25.0 mg, 0.137 mmol) and CDI (22.3 mg, 0.137 mmol) were dissolved in CH_2Cl_2 (3 mL) and stirred for 30 min at rt. This reaction mixture was subsequently transferred to the previously prepared amine. TEA (19.1 μL, 0.137 mmol) was added and the reaction mixture was stirred for 18 h at rt. Upon completion, the solvent was removed *in vacuo* and purified by preparative TLC (1:9 to 17.5:82.5 MeOH:CH_2Cl_2) to give **29** as a pale yellow oil (25.0 mg, 45%). R_f 0.35 (1:9 MeOH:CH_2Cl_2); ν_{max} (neat)/cm^{-1} 3280, 2923, 2867, 2120, 1688, 1632, 1251; δ_H (500 MHz, $CDCl_3$) 7.79 (2H, d, J = 8.3 Hz, 2 × H24), 7.64 (2H, d, J = 8.3 Hz, 2 × H23), 4.48 (1H, ddd, J = 7.9, 5.0, 0.8 Hz, H2), 4.44 (1H, spt, J = 3.8 Hz, H27), 4.29 (1H, dd, J = 7.9, 4.5 Hz, H5), 3.47–3.66 (14H, m, H13–H18, H20), 3.24 (2H, t, J = 6.8 Hz, H11), 3.17–3.21 (1H, m, H4), 2.91 (1H, dd, J = 12.8, 5.0 Hz, H3’), 2.70 (1H, d, J = 12.7 Hz, H3), 2.18 (2H, t, J = 7.3 Hz, H9), 1.89 (2H, q, J = 6.4 Hz, H19), 1.55–1.76 (6H, m, H6, H8, H12), 1.39–1.47 (2H, m, H7), 0.36 (6H, d, J = 3.8 Hz, 2 × H26); δ_C (126 MHz, $CDCl_3$) 175.9 (C10), 170.1 (C21), 166.1 (C1), 143.0 (C25), 136.5 (C22), 135.2 (2 × C24), 127.5 (2 × C23), 71.6 (one C from C13–C18), 71.5 (one C from C13–C18), 71.3 (one C from C13–C18), 71.2 (one C from C13–C18), 70.3 (one C from C13–C18), 69.9 (one C from C13–C18), 63.4 (C5), 61.6 (C2), 57.0 (C4), 41.0 (C3), 38.7 (C20), 37.8 (C11), 36.9 (C9), 30.4 (C12, C19), 29.8

(C7), 29.5 (C8), 26.9 (C6), –3.9 (2 × C26); **HRMS** (ESI) m/z found $[M+H]^+$ 609.3149, $C_{29}H_{49}N_4O_6{}^{32}S^{28}Si^+$ required 609.3142.

***tert*-Butyl (1-(3',6'-bis(dimethylamino)-3-oxo-3*H*-spiro[isobenzofuran-1,9'-xanthen]-5-yl)-1-oxo-6,9,12-trioxa-2-azapentadecan-15-yl)carbamate (S8)**

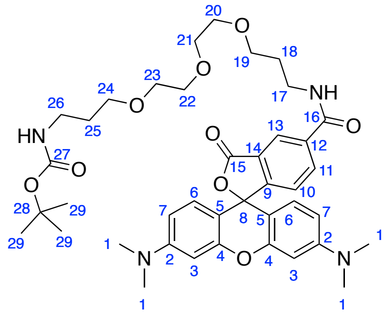

HATU (175 mg, 0.460 mmol) was added to a solution of 5-TAMRA (100 mg, 0.232 mmol) in DMF (1 mL) and stirred for 10 min. *tert*-Butyl (3-(2-(2-(3-aminopropoxy)ethoxy)ethoxy)propyl)carbamate (89.1 mg, 0.278 mmol) and TEA (170 µL, 1.22 mmol) were subsequently added to the activated carboxylic acid mixture and stirred at rt for 16 h. Upon completion, the solvent was removed *in vacuo* and purified by FCC (1:9 MeOH:CH_2Cl_2) to give **S8** as a dark red oil (60.0 mg, 36%). $\boldsymbol{R_f}$ 0.19 (1:4 MeOH:CH_2Cl_2); $\boldsymbol{\nu}_{max}$ (neat)/cm^{-1} 3724, 3627, 1646, 1595, 1496, 1349, 1187, 1132; $\boldsymbol{\delta}_H$ (500 MHz, CD_3OD) 8.77 (1H, d, *J* = 1.6 Hz, H13), 8.27 (1H, dd, *J* = 7.9, 1.9 Hz, H11), 7.54 (1H, d, *J* = 7.9 Hz, H10), 7.15 (2H, d, *J* = 9.5 Hz, 2 × H6), 7.07 (2H, dd, *J* = 9.5, 2.5 Hz, 2 × H7), 6.99 (2H, d, *J* = 2.4 Hz, 2 × H3), 3.56–3.69 (12H, m, H17, H19–H23), 3.49 (2H, t, *J* = 6.2 Hz, H24), 3.32 (12H, s, 4 × H1), 3.10 (2H, t, *J* = 6.8 Hz, H26), 1.96 (2H, quint, *J* = 6.4 Hz, H18), 1.69 (2H, quint, *J* = 6.5 Hz, H25), 1.42 (9H, s, 3 × H29); $\boldsymbol{\delta}_C$ (126 MHz, CD_3OD) 168.0 (C16), 167.3 (C15), 160.7 (C8), 159.1 (2 × C2 or 2 × C4), 159.0 (2 × C2 or 2 × C4), 158.5 (C27), 138.1 (C9), 137.8 (C12), 132.8 (C14), 132.3 (C11), 132.0 (2 × C6, C10), 131.3 (C13), 115.6 (2 × C7), 114.7 (2 × C5), 97.5 (2 × C3), 79.9 (C28), 71.5 × 2 (2 C from C19–C24), 71.3 (1 C from C19–C24), 71.2 (1 C from C19–C24), 70.3 (1 C from C19–C24), 69.9 (1 C from C19–C24), 40.9 (4 × C1), 39.0 (C17), 38.7 (C26), 30.9 (C25), 30.4 (C18), 28.8 (3 × C29); **HRMS** (ESI) *m/z* found $[M+H]^+$ 733.3777, $C_{40}H_{53}N_4O_9{}^+$ required 733.3807.

3',6'-Bis(dimethylamino)-*N*-(1-(4-(dimethylsilyl)phenyl)-1-oxo-6,9,12-trioxa-2-azapentadecan-15-yl)-3-oxo-3*H*-spiro[isobenzofuran-1,9'-xanthene]-5-carboxamide (39)

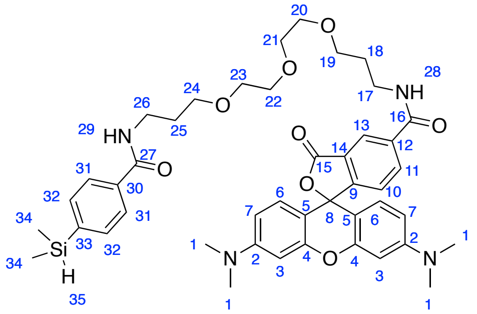

4 M HCl in dioxane (1.20 mL, 4.80 mmol) was added to **S8** (17.0 mg, 0.023 mmol) and stirred for 5 h at rt. The reaction mixture was concentrated *in vacuo* to give the corresponding amine. Carboxylic acid **S7** (12.6 mg, 0.070 mmol) and HATU (29.3 mg, 0.077 mmol) were dissolved in DMF (1 mL) and stirred for 30 min at rt. This reaction mixture was subsequently transferred to the previously prepared amine. TEA (48.8 µL, 0.350 mmol) was added and the reaction mixture was stirred for 18 h at rt. Upon completion, the solvent was removed *in vacuo* and purified by preparative TLC (3:7 MeOH:CH_2Cl_2) to give **39** as a dark red oil (5.20 mg, 32%). $\boldsymbol{R_f}$ 0.47 (3:7 MeOH:CH_2Cl_2); $\boldsymbol{\nu}_{max}$ (neat)/cm^{-1} 3617, 2925, 1750, 1595, 1429, 1349, 1189, 1118; $\boldsymbol{\delta}_{\mathbf{H}}$ (500 MHz, DMSO-d_6) 8.80 (1H, t, J = 5.5 Hz, H28), 8.43–8.45 (2H, m, H13, H29), 8.21 (1H, dd, J = 8.0, 1.6 Hz, H11), 7.81 (2H, d, J = 8.2 Hz, 2 × H31), 7.61 (2H, d, J = 8.2 Hz, 2 × H32), 7.30 (1H, d, J = 8.0 Hz, H10), 6.46–6.52 (6H, m, 2 × H3, 2 × H6, 2 × H7), 4.38 (1H, spt, J = 3.7 Hz, H35), 3.43–3.54 (12H, m, H19–H24), 3.28–3.38 (unresolved due to overlap with solvent peak, H17, H26), 2.94 (12H, s, 4 × H1), 1.72–1.82 (4H, m, H18, H25), 0.34 (6H, d, J = 3.8 Hz, 2 × H34); $\boldsymbol{\delta}_{\mathbf{C}}$ (126 MHz, DMSO-d_6) 168.4 (C15), 166.1 (C27), 164.7 (C16), 154.8 (C9), 152.1 (2 × C2 or 2 × C4), 152.0 (2 × C2 or 2 × C4), 140.2 (C33), 136.2 (C12), 135.4 (C30), 134.5 (C11), 133.8 (2 × C32), 128.4 (2 × C6), 126.8 (C14), 126.4 (2 × C31), 124.1 (C10), 123.1 (C13), 109.0 (2 × C3), 105.6 (2 × C5), 98.0 (2 × C7), 84.7 (C8), 69.8 (2 C from C19–C24), 69.6 (2 C from C19–C24), 68.3 (1 C from C19–C24), 68.2 (1 C from C19–C24), 39.0–40.0 (C1, unresolved due to overlap with solvent peak), 36.9 (C17 or C26), 36.7 (C17 or C26),

29.4 (C18 or C25), 29.2 (C18 or C25), –4.1 (2 × C34); **HRMS** (ESI) *m/z* found $[M+H]^+$ 795.3762, $C_{44}H_{55}N_4O_8{}^{28}Si^+$ required 795.3789.

2.2. Alkyne substrates

3,6,9,12-Tetraoxapentadec-14-yne (2)

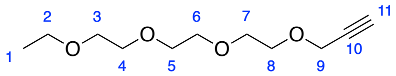

Tri(ethylene glycol) monoethyl ether (5.35 g, 30.0 mmol) was dissolved in THF (25 mL) and cooled to 0 ºC. NaH (720 mg, 60% dispersion in mineral oil, 30.0 mmol) was slowly added and stirred at rt for 30 min. Propargyl chloride (2.24 g, 30.0 mmol) was then added and stirred for 16 h. The reaction was diluted in CH_2Cl_2 (50 mL) and washed with water (100 mL). The phases were separated and the aqueous layer was extracted with CH_2Cl_2 (3 × 25 mL). The combined organic extracts were washed with brine (50 mL), dried ($MgSO_4$), concentrated *in vacuo* and purified by FCC (1:9 EtOAc:CH_2Cl_2) to give **2** as a pale yellow oil (4.10 g, 64%). $\boldsymbol{R_f}$ 0.27 (1:9 EtOAc:CH_2Cl_2); $\boldsymbol{\nu}_{max}$ (neat)/cm^{-1} 3249, 2867, 1457, 1350, 1104, 1033, 844; $\boldsymbol{\delta}_{\mathbf{H}}$ (500 MHz, $CDCl_3$) 4.19 (2H, d, *J* = 2.4 Hz, H9), 3.56–3.70 (12H, m, H3–H8), 3.51 (2H, q, *J* = 7.0 Hz, H2), 2.41 (1H, t, *J* = 2.4 Hz, H11), 1.19 (3H, t, *J* = 7.0 Hz, H1); $\boldsymbol{\delta}_{\mathbf{C}}$ (126 MHz, $CDCl_3$) 79.8 (C10), 74.6 (C11), 70.8 (1 C from C3–C8), 70.7 × 2 (2 C from C3–C8), 70.5 (1 C from C3–C8), 69.9 (1 C from C3–C8), 69.2 (1 C from C3–C8), 66.7 (C2), 58.5 (C9), 15.3 (C1); **HRMS** (ESI) *m/z* found $[M+H]^+$ 217.1432, $C_{11}H_{21}O_4{}^+$ required 217.1440.

3,4-Dimethoxy-*N*-(pent-4-yn-1-yl)benzamide (9)

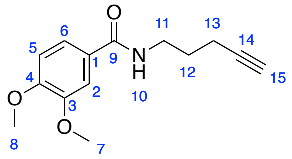

3,4-Dimethoxybenzoic acid (263 mg, 1.44 mmol) and CDI (234 mg, 1.44 mmol) were dissolved in CH_2Cl_2 (8 mL) and stirred for 30 min at rt. 4-Pentyn-1-amine (117 µL, 1.20 mmol) and TEA (201 µL, 1.44 mmol) were subsequently added and the reaction mixture was stirred at rt for 18 h. Upon completion, sat. $NaHCO_3$ (10 mL) was added and the mixture was extracted with CH_2Cl_2 (3 × 10 mL). The combined organic layers were dried (Na_2SO_4), concentrated *in vacuo* and purified by FCC (2:3 to 1:1

EtOAc:petroleum ether) to give **9** as a yellow oil (274 mg, 92%). ***R**$_f$* 0.13 (1:1 EtOAc:PE); ***v**$_{max}$* (neat)/cm^{-1} 3291, 2937, 2839, 1631, 1505, 1267, 1229, 1131, 1023; ***δ**$_H$* (500 MHz, $CDCl_3$) 7.42 (1H, d, *J* = 2.1 Hz, H2), 7.26 (1H, dd, *J* = 8.3, 2.1 Hz, H6), 6.86 (1H, d, *J* = 8.4 Hz, H5), 6.34 (1H, s, H10), 3.93 (3H, s, H7), 3.92 (3H, s, H8), 3.58 (2H, app q, *J* = 6.5 Hz, H11), 2.31 (2H, td, *J* = 10.3, 2.7 Hz, H13), 2.02 (1H, t, *J* = 2.7 Hz, H15), 1.85 (2H, app quint, *J* = 6.8 Hz, H12); ***δ**$_C$* (126 MHz, $CDCl_3$) 167.1 (C9), 151.7 (C4), 149.0 (C3), 127.3 (C1), 119.1 (C6), 110.6 (C2), 110.2 (C5), 83.7 (C14), 69.3 (C15), 56.0 (C7, C8), 39.3 (C11), 28.1 (C12), 16.3 (C13); **HRMS** (ESI) *m/z* found $[M+H]^+$ 248.1282, $C_{14}H_{18}NO_3^+$ required 248.1287.

_N_-(2-(2-(2-Hydroxyethoxy)ethoxy)ethyl)-3,4-dimethoxybenzamide (S9)

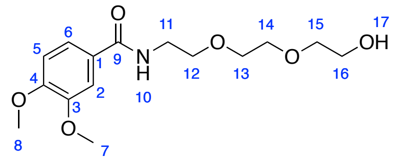

CDI (2.75 g, 17.0 mmol) was added to a solution of 3,4-dimethoxybenzoic acid (3.37 g, 18.5 mmol) in CH_2Cl_2 (60 mL) and stirred at rt for 30 min. 2-(2-(2-aminoethoxy)ethoxy)ethan-1-ol (2.30 g, 15.4 mmol) and TEA (2.58 mL, 18.5 mmol) in CH_2Cl_2 (40 mL) were subsequently added to the reaction mixture and stirred at rt for 16 h. Upon completion, the solvent was removed *in vacuo* and purified by FCC (1:99 to 1:49 MeOH:CH_2Cl_2) to give **S9** as a yellow wax (2.40 g, 50%). ***R**$_f$* 0.17 (1:19 MeOH:CH_2Cl_2); ***v**$_{max}$* (neat)/cm^{-1} 3361, 2869, 1634, 1507, 1268, 1231, 1131, 1023, 768; ***δ**$_H$* (500 MHz, $CDCl_3$) 7.46 (1H, d, *J* = 1.4 Hz, H2), 7.34 (1H, dd, *J* = 8.3, 1.6 Hz, H6), 6.86 (1H, d, *J* = 8.4 Hz, H5), 6.76 (1H, s, H10), 3.93 (3H, s, H7), 3.91 (3H, s, H8), 3.61–3.72 (12H, m, H11–H16), 2.68 (1H, t, *J* = 5.7 Hz, H17); ***δ**$_C$* (126 MHz, $CDCl_3$) 167.1 (C9), 151.7 (C4), 148.9 (C3), 127.1 (C1), 119.5 (C6), 110.7 (C2), 110.2 (C5), 72.5 (1 C from C12–C16), 70.4 (2 C from C12–C16), 70.1 (1 C from C12–C16), 61.7 (1 C from C12–C16), 56.0 × 2 (C7, C8), 39.7 (C11); **HRMS** (ESI) *m/z* found $[M+H]^+$ 314.1620, $C_{15}H_{24}NO_6^+$ required 314.1604.

2-(2-(2-(3,4-Dimethoxybenzamido)ethoxy)ethoxy)ethyl 4-methylbenzene-sulfonate (S10)

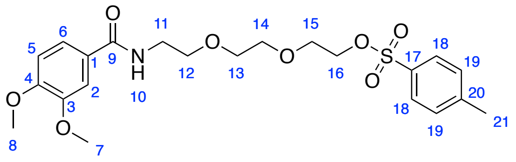

TsCl (465 mg, 2.39 mmol) was added to a mixture of **S9** (500 mg, 1.60 mmol), TEA (445 µL, 3.19 mmol) and TMA·HCl (15.3 mg, 0.160 mmol) in acetonitrile (4 mL) at 0 ºC. The reaction mixture was stirred at 0 ºC for 20 min, rt for 40 min, before being poured into water (10 mL) and extracted with EtOAc (3 × 5 mL). The combined organic layers were dried (Na_2SO_4), concentrated *in vacuo* and purified by FCC (0:100 to 1:99 MeOH:CH_2Cl_2) to give **S10** as a viscous oil (667 mg, 89%). $\boldsymbol{R_f}$ 0.40 (1:19 MeOH:CH_2Cl_2); $\boldsymbol{\nu}_{max}$ (neat)/cm^{-1} 3379, 2928 , 1636, 1506, 1352, 1131; $\boldsymbol{\delta}_{\mathbf{H}}$ (500 MHz, $CDCl_3$) 7.77 (2H, d, *J* = 8.2 Hz, 2 × H19), 7.44 (1H, d, *J* = 1.5 Hz, H2), 7.32 (2H, d, *J* = 8.1 Hz, 2 × H18), 7.29 (1H, dd, *J* = 8.4, 1.7 Hz, H6), 6.88 (1H, d, *J* = 8.4 Hz, H5), 6.62 (1H, s, H10), 4.16 (2H, t, *J* = 4.7 Hz, H16), 3.93 (3H, s, H7), 3.91 (3H, s, H8), 3.70 (2H, t, *J* = 4.7 Hz, H15), 3.61–3.64 (8H, m, H11–H14), 2.43 (3H, s, H21); $\boldsymbol{\delta}_{\mathbf{C}}$ (126 MHz, $CDCl_3$) 167.0 (C9), 151.7 (C4), 148.9 (C3), 144.9 (C17), 132.9 (C20), 129.8 (2 × C19), 127.9 (2 × C18), 127.1 (C1), 119.3 (C6), 110.7 (C2), 110.3 (C5), 70.7 (1 C from C12–C16), 70.2 (1 C from C12–C16), 70.0 (1 C from C12–C16), 69.1 (1 C from C12–C16), 68.7 (1 C from C12–C16), 56.0 (C7, C8), 39.7 (C11), 21.6 (C21); **HRMS** (ESI) *m/z* found $[M+H]^+$ 468.1709, $C_{22}H_{30}NO_8{}^{32}S^+$ required 468.1692.

***S*-(2-(2-(2-(3,4-Dimethoxybenzamido)ethoxy)ethoxy)ethyl) ethanethioate (S11)**

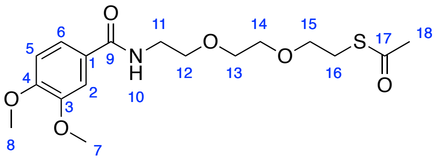

S10 (500 mg, 1.07 mmol) and KSAc (183 mg, 1.60 mmol) were dissolved in DMF (4 mL) and stirred under Ar for 18 h at rt. Upon completion, sat. NH_4Cl (3 mL), CH_2Cl_2 (4 mL) and brine (600 µL) were added to the reaction mixture, stirred vigorously for 15

min and extracted with CH_2Cl_2 (3 × 10 mL). The combined organic layers were dried (Na_2SO_4), concentrated *in vacuo* and purified by FCC (1:99 MeOH:CH_2Cl_2) to give **S11** as a brown oil (400 mg, 82%). $\boldsymbol{R_f}$ 0.50 (1:19 MeOH:CH_2Cl_2); $\boldsymbol{\nu_{max}}$ (neat)/cm^{-1} 3335, 2866, 1690, 1637, 1507, 1269, 1231, 1131, 1024, 769; $\boldsymbol{\delta_H}$ (500 MHz, CD_3OD) 7.45–7.48 (2H, m, H2, H6), 7.00 (1H, d, *J* = 8.2 Hz, H5), 3.88 (3H, s, H7), 3.88 (3H, s, H8), 3.55–3.66 (10H, m, H11–H15), 3.03 (2H, t, *J* = 6.5 Hz, H16), 2.28 (3H, s, H18); $\boldsymbol{\delta_C}$ (126 MHz, CD_3OD) 197.0 (C17), 169.8 (C9), 153.5 (C4), 150.2 (C3), 128.0 (C1), 121.9 (C6), 112.0 (C2), 111.9 (C5), 71.3 × 2 (2 C from C12–C15), 70.8 (1 C from C12–C15), 70.7 (1 C from C12–C15), 56.5 (C7 or C8), 56.4 (C7 or C8), 40.9 (C11), 30.4 (C18), 29.5 (C16); **HRMS** (ESI) *m/z* found $[M+Na]^+$ 394.1297, $C_{17}H_{25}NO_6{}^{32}SNa^+$ required 394.1300.

3,4-Dimethoxy-*N*-(2-(2-(2-(prop-2-yn-1-ylthio)ethoxy)ethoxy)ethyl)-benzamide (11)

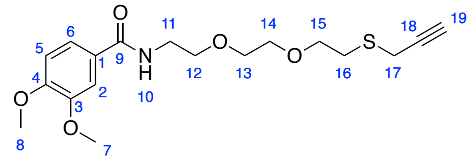

S11 (2.80 g, 8.10 mmol) and NaOMe (529 mg, 10.5 mmol) were dissolved in Ar-purged MeOH (37 mL). The deprotection was stirred until completion, as determined by TLC. Propargyl bromide (2.50 mL, 24.0 mmol) was then subsequently added to the reaction mixture and stirred for 3 h. The reaction mixture was neutralised with dilute HCl in MeOH, concentrated *in vacuo*, re-dissolved in CH_2Cl_2 (15 mL) and washed with water (2 × 15 mL). The combined organic layers were dried (Na_2SO_4), concentrated *in vacuo* and purified by FCC (1:99 MeOH:CH_2Cl_2) to give **11** as a brown oil (2.60 g, 93%). $\boldsymbol{R_f}$ 0.27 (1:19 MeOH:CH_2Cl_2); $\boldsymbol{\nu_{max}}$ (neat)/cm^{-1} 3284, 2869, 1636, 1506, 1268, 1230, 1131, 1023, 768; $\boldsymbol{\delta_H}$ (500 MHz, $CDCl_3$) 7.44 (1H, d, *J* = 2.1 Hz, H2), 7.30 (1H, dd, *J* = 8.3, 2.1 Hz, H6), 6.87 (1H, d, *J* = 8.4 Hz, H5), 6.60 (1H, s, H10), 3.94 (3H, s, H7), 3.92 (3H, s, H8), 3.72 (2H, t, *J* = 6.6 Hz, H15), 3.64–3.69 (8H, m, H11–H14), 3.28 (2H, d, *J* = 2.7 Hz, H17), 2.87 (2H, t, *J* = 6.5 Hz, H16), 2.22 (1H, t, *J* = 2.6 Hz, H19); $\boldsymbol{\delta_C}$ (126 MHz, $CDCl_3$) 167.1 (C9), 151.7 (C4), 148.9 (C3), 127.3 (C1), 119.3 (C6), 110.7 (C2), 110.2 (C5), 80.0 (C18), 71.2 (C19), 70.6 (1 C from C12–C15), 70.2 (2 C from C12–C15), 70.0 (1 C from C12–C15), 56.0 × 2 (C7, C8), 39.7 (C11), 30.9 (C16), 19.7 (C17); **HRMS** (ESI) *m/z* found $[M+H]^+$ 368.1532, $C_{18}H_{26}NO_5{}^{32}S^+$ required

368.1532.

3,4-Dimethoxy-*N*-(2-(2-(2-selenocyanatoethoxy)ethoxy)ethyl)benzamide (S12)

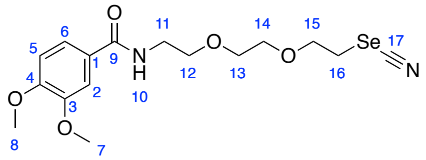

KSeCN (240 mg, 1.70 mmol) was added to a mixture of **S10** (650 mg, 1.40 mmol) and 18-crown-6 (7.40 mg, 0.028 mmol) in THF (15 mL) and refluxed for 18 h. Upon completion, the mixture was poured into sat. $NaHCO_3$ (10 mL) and extracted with EtOAc (3 × 10 mL). The combined organic layers were dried (Na_2SO_4), concentrated *in vacuo* and purified by FCC (0:100 to 1:99 MeOH:CH_2Cl_2) to give **S12** as a light yellow oil (510 mg, 91%). $\boldsymbol{R_f}$ 0.38 (1:19 MeOH:CH_2Cl_2); $\boldsymbol{\nu}_{max}$ (neat)/cm^{-1} 3369, 2869, 2150, 1637, 1507, 1269, 1230, 1131, 1023, 769; $\boldsymbol{\delta_H}$ (500 MHz, $CDCl_3$) 7.44 (1H, d, J = 2.0 Hz, H2), 7.30 (1H, dd, J = 8.4, 2.1 Hz, H6), 6.87 (1H, d, J = 8.4 Hz, H5), 6.59 (1H, s, H10), 3.93 (3H, s, H7), 3.92 (3H, s, H8), 3.88 (2H, t, J = 6.0 Hz, H15), 3.64–3.69 (8H, m, H11–H14), 3.23 (2H, t, J = 6.0 Hz, H16); $\boldsymbol{\delta_C}$ (126 MHz, $CDCl_3$) 167.1 (C9), 151.7 (C4), 149.0 (C3), 127.2 (C1), 119.3 (C6), 110.7 (C2), 110.2 (C5), 101.6 (C17), 70.5 (1 C from C12–C15), 70.1 (1 C from C12–C15), 70.0 (1 C from C12–C15), 69.4 (1 C from C12–C15), 56.0 × 2 (C7, C8), 39.7 (C11), 29.0 (C16); **HRMS** (ESI) *m/z* found $[M+H]^+$ 403.0759, $C_{16}H_{23}N_2O_5{}^{80}Se^+$ required 403.0772.

3,4-Dimethoxy-*N*-(2-(2-(2-(prop-2-yn-1-ylselanyl)ethoxy)ethoxy)ethyl)-benzamide (12)

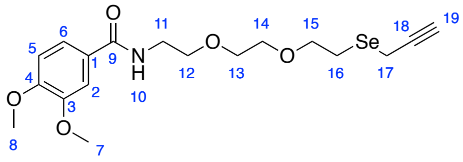

$NaBH_4$ (9.43 mg, 0.249 mmol) was added to **S12** (100 mg, 0.249 mmol) in Ar-purged ethanol (1 mL) at 0 ºC and stirred for 30 min. Propargyl bromide (30.5 µL, 0.274 mmol) was subsequently added and stirred at 0 ºC until completion, as determined by TLC. 1 M aqueous HCl (1 mL) was added and the reaction mixture was extracted with CH_2Cl_2 (3 × 5 mL). The combined organic layers were dried (Na_2SO_4), concentrated *in vacuo* and

purified by FCC (0:100 to 1:99 MeOH:CH_2Cl_2) to give **12** as a yellow oil (93.0 mg, 90%). ***R*$_f$** 0.46 (1:19 MeOH:CH_2Cl_2); **ν_{max}** (neat)/cm^{-1} 3285, 2868, 1638, 1507, 1269, 1231, 1132, 1024, 769; **δ_H** (500 MHz, $CDCl_3$) 7.44 (1H, d, *J* = 2.0 Hz, H2), 7.30 (1H, dd, *J* = 8.3, 2.1 Hz, H6), 6.87 (1H, d, *J* = 8.4 Hz, H5), 6.60 (1H, s, H10), 3.94 (3H, s, H7), 3.92 (3H, s, H8), 3.79 (2H, t, *J* = 6.8 Hz, H15), 3.65–3.69 (8H, m, H11–H14), 3.22 (2H, d, *J* = 2.7 Hz, H17), 2.93 (2H, t, *J* = 6.8 Hz, H16), 2.23 (1H, t, *J* = 2.7 Hz, H19); **δ_C** (126 MHz, $CDCl_3$) 167.1 (C9), 151.7 (C4), 149.0 (C3), 127.3 (C1), 119.3 (C6), 110.7 (C2), 110.2 (C5), 80.9 (C18), 71.3 (C19), 71.2 (1 C from C12–C15), 70.2 (1 C from C12–C15), 70.1 (1 C from C12–C15), 69.6 (1 C from C12–C15), 56.0 × 2 (C7, C8), 39.7 (C11), 23.5 (C16), 7.3 (C17); **HRMS** (ESI) *m/z* found $[M+H]^+$ 416.0992, $C_{18}H_{26}NO_5{}^{80}Se^+$ required 416.0976.

3,4-Dimethoxy-*N*-(2-(2-(2-(prop-2-yn-1-yloxy)ethoxy)ethoxy)ethyl)benzamide (13)

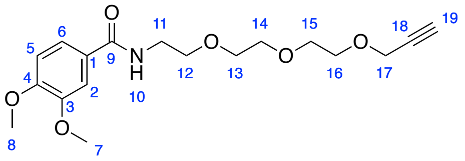

CDI (615 mg, 3.78 mmol) was added to a solution of 3,4-dimethoxybenzoic acid (690 mg, 3.78 mmol) in CH_2Cl_2 (30 mL) and stirred at rt for 30 min. 2-(2-(2-(prop-2-yn-1-yloxy)ethoxy)ethoxy)ethan-1-amine (590 mg, 3.15 mmol) and TEA (530 µL, 3.78 mmol) in CH_2Cl_2 (20 mL) were subsequently added to the reaction mixture and stirred at rt for 16 h. Upon completion, the solvent was removed *in vacuo* and purified by FCC (1:99 to 1:49 MeOH:CH_2Cl_2) to give **13** as a pale yellow oil (865 mg, 78%). ***R*$_f$** 0.22 (1:19 MeOH:CH_2Cl_2); **ν_{max}** (neat)/cm^{-1} 3260, 2871, 1636, 1507, 1268, 1131, 1097, 1024; **δ_H** (500 MHz, $CDCl_3$) 7.45 (1H, d, *J* = 2.1 Hz, H2), 7.32 (1H, dd, *J* = 8.3, 2.1 Hz, H6), 6.87 (1H, d, *J* = 8.4 Hz, H5), 6.75 (1H, s, H10), 4.15 (2H, d, *J* = 2.4 Hz, H17), 3.93 (3H, s, H7), 3.92 (3H, s, H8), 3.65–3.68 (12H, m, H11–H16), 2.41 (1H, t, *J* = 2.4 Hz, H19); **δ_C** (126 MHz, $CDCl_3$) 167.0 (C9), 151.6 (C4), 148.9 (C3), 127.3 (C1), 119.5 (C6), 110.7 (C2), 110.2 (C5), 79.5 (C18), 74.6 (C19), 70.5 (1 C from C12–C16), 70.3 (1 C from C12–C16), 70.2 (1 C from C12–C16), 70.0 (1 C from C12–C16), 69.0 (1 C from C12–C16), 58.4 (C17), 56.0 × 2 (C7, C8), 39.8 (C11); **HRMS** (ESI) *m/z* found $[M+H]^+$ 352.1768, $C_{18}H_{26}NO_6{}^+$ required 352.1760.

3-Selenocyanatoprop-1-yne (S13)

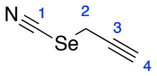

Propargyl bromide (9.28 mL, 83.2 mmol) was added to a solution of KSeCN (4.00 g, 27.8 mmol) in DMF (28 mL) and stirred at 0 ºC. Upon completion (as determined by TLC), reaction was diluted with Et_2O (300 mL) and washed sequentially with water (2 × 100 mL) and brine (300 mL). The organic layers were combined, dried (Na_2SO_4), concentrated *in vacuo* and purified by FCC (1:4 EtOAc:petroleum ether) to give **S13** as a brown oil (3.20 g, 80%). $\boldsymbol{R_f}$ 0.31 (1:4 EtOAc:PE); $\boldsymbol{\delta_H}$ (500 MHz, $CDCl_3$) 3.75 (2H, d, *J* = 2.7 Hz, H2), 2.53 (1H, t, *J* = 2.7 Hz, H4); $\boldsymbol{\delta_C}$ (126 MHz, $CDCl_3$) 100.6 (C1), 77.0 (C3), 75.9 (C4), 14.2 (C2); **HRMS** (ESI) *m/z* found $[M+H]^+$ 145.9504, $C_4H_4N^{80}Se^+$ required 145.9509. This data is consistent with that previously reported.[3]

Methyl 2-acetamido-3-(prop-2-yn-1-ylselanyl)propanoate (20)

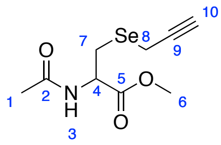

$NaBH_4$ (52.6 mg, 1.39 mmol) was added portion-wise to a solution of **S13** (200 mg, 1.39 mmol) in Ar-purged MeOH (7 mL) and stirred at rt for 5 min. This solution was then added dropwise to a solution of methyl 2-acetamidoacrylate (49.8 mg, 0.348 mmol) in PBS buffer at pH 7.4 (2 mL) and stirred at rt for 1 h. Upon completion, the reaction was diluted with EtOAc (50 mL) and washed subsequently with 1 M aqueous HCl (50 mL), sat. $NaHCO_3$ (50 mL), water (50 mL) and brine (50 mL). The organic layers were dried (Na_2SO_4), concentrated *in vacuo* and purified by FCC (1:1 EtOAc:petroleum ether) to give **20** as a pale yellow oil (51.0 mg, 55%). $\boldsymbol{R_f}$ 0.22 (3:5 EtOAc:PE); $\boldsymbol{\nu_{max}}$ (neat)/cm^{-1} 3269, 2955, 2156, 1740, 1653, 1527, 1436, 1372, 1211, 1173; $\boldsymbol{\delta_H}$ (400 MHz, $CDCl_3$) 6.32 (1H, d, *J* = 5.8 Hz, H3), 4.97 (2H, dt, *J* = 7.7, 5.0 Hz, H4), 3.78 (3H, s, H6), 3.15–3.28 (4H, m, H7, H8), 2.31 (1H, t, *J* = 2.7 Hz, H10), 2.05 (3H, s, H1); $\boldsymbol{\delta_C}$ (101 MHz, $CDCl_3$) 171.5 (C5), 169.9 (C2), 80.5 (C9), 72.2 (C10), 52.9

(C6), 52.0 (C4), 26.7 (C7), 23.3 (C1), 8.1 (C8); **HRMS** (ESI) *m/z* found $[M+Na]^+$ 285.9958, $C_9H_{13}NO_3{}^{80}SeNa^+$ required 285.9958.

_S_-(2-(2-(2-(Prop-2-yn-1-yloxy)ethoxy)ethoxy)ethyl) ethanethioate (40)

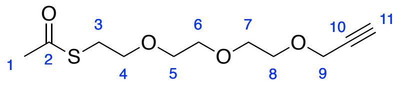

KI (161 mg, 0.968 mmol) and KSAc (1.22 g, 10.6 mmol) were added to a stirring solution of 3-(2-(2-(2-chloroethoxy)ethoxy)ethoxy)prop-1-yne (2.00 g, 9.68 mmol) in acetonitrile (50 ml) and refluxed for 16 h. Upon completion, the solution was cooled to rt and diluted with water (75 mL) and extracted with EtOAc (4 × 25 mL). The combined organic layers were dried (Na_2SO_4), concentrated *in vacuo* and purified by FCC (1:99 MeOH:CH_2Cl_2) to give **40** as a yellow oil (1.72 g, 71%). $\boldsymbol{R_f}$ 0.10 (1:99 MeOH:CH_2Cl_2); $\boldsymbol{\delta_H}$ (500 MHz, $CDCl_3$) 4.20 (2H, d, *J* = 2.3 Hz, H9), 3.63–3.70 (8H, m, H5–H8), 3.60 (2H, t, *J* = 6.5 Hz, H4), 3.09 (2H, t, *J* = 6.5 Hz, H3), 2.42 (1H, t, *J* = 2.3 Hz, H11), 2.33 (3H, s, H1); $\boldsymbol{\delta_C}$ (126 MHz, $CDCl_3$) 195.7 (C2), 79.8 (C10), 74.6 (C11), 70.7 (1 C from C4–C8), 70.6 (1 C from C4–C8), 70.5 (1 C from C4–C8), 69.9 (1 C from C4–C8), 69.3 (1 C from C4–C8), 58.6 (C9), 30.7 (C1), 29.0 (C3); **HRMS** (ESI) *m/z* found $[M+Na]^+$ 269.0827, $C_{11}H_{18}O_4{}^{32}SNa^+$ required 269.0824. This data is consistent with that previously reported.[4]

Methyl 15-acetamido-4,7,10-trioxa-13-thiahexadec-1-yn-16-oate (21)

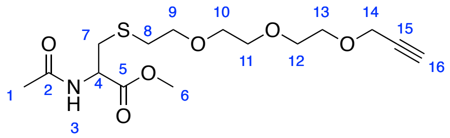

40 (172 mg, 0.698 mmol) and NaOMe (49.0 mg, 0.907 mmol) were dissolved in Ar-purged MeOH (4 mL). The deprotection was stirred until completion, as determined by TLC. The mixture was transferred dropwise to a stirring solution of methyl 2-acetamidoacrylate (50.0 mg, 0.349 mmol) in PBS buffer at pH 7.4 (2 mL) and stirred at rt for 2 h. On completion, the reaction was diluted with EtOAc (10 mL) and washed with 1 M aqueous HCl (10 mL), sat. $NaHCO_3$ (10 mL), water (10 mL) and brine (10 mL). The organic layers were dried (Na_2SO_4), concentrated *in vacuo* and purified by FCC (0:100 to 3:97 MeOH:CH_2Cl_2) to give **21** as a yellow oil (97.0 mg, 80%). $\boldsymbol{R_f}$ 0.17

(3:97 MeOH:CH_2Cl_2); ν_{max} (neat)/cm^{-1} 3266, 2920, 2870, 1743, 1657, 1534, 1440, 1372, 1214, 1091, 1033; δ_H (500 MHz, $CDCl_3$) 6.58 (1H, d, J = 7.1 Hz, H3), 4.80–4.84 (1H, m, H4), 4.20 (2H, d, J = 2.4 Hz, H14), 3.76 (3H, s, H6), 3.61–3.72 (10H, m, H9–H13), 3.05 (2H, d, J = 5.2 Hz, H7), 2.67–2.77 (2H, m, H8), 2.43 (1H, t, J = 2.4 Hz, H16), 2.05 (3H, s, H1); δ_C (126 MHz, $CDCl_3$) 171.4 (C5), 170.1 (C2), 79.8 (C15), 74.7 (C16), 71.3 (1 C from C9–C13), 70.7 (1 C from C9–C13), 70.6 (1 C from C9–C13), 70.5 (1 C from C9–C13), 69.2 (1 C from C9–C13), 58.5 (C14), 52.8 (C6), 52.5 (C4), 34.8 (C7), 32.5 (C8), 23.2 (C1); **HRMS** (ESI) *m/z* found $[M+Na]^+$ 370.1294, $C_{15}H_{25}NO_6{}^{28}SiNa^+$ required 370.1295.

S-(Prop-2-yn-1-yl) ethanethioate (S15)

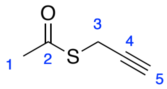

Thioacetic acid (4.70 mL, 65.7 mmol) in THF (15 mL) was added to a solution of NaH (60% in mineral oil, 2.76 g, 69.0 mmol) in THF (100 mL) at 0 ºC. The resulting mixture was stirred for 30 min at rt, and was subsequently cooled to 0 ºC and propargyl bromide (3.65 mL, 32.8 mmol) was added. The reaction was stirred for an additional 30 min at rt. Upon completion, the reaction was quenched by water (80 mL) and the layers were separated. The aqueous layer was further extracted with Et_2O (4 × 35 mL), and the combined organic layers were washed with water (2 × 35 mL), dried ($MgSO_4$), concentrated *in vacuo* and purified by FCC (1:99 Et_2O:petroleum ether) to give **S15** as a yellow oil (3.13 mg, 84%). R_f 0.33 (1:9 Et_2O:PE); δ_H (400 MHz, $CDCl_3$) 3.64 (2H, d, J = 2.7 Hz, H3), 2.37 (3H, s, H1), 2.18 (1H, t, J = 2.7 Hz, H5); δ_C (101 MHz, $CDCl_3$) 194.0 (C2), 78.9 (C4), 71.0 (C5), 30.3 (C1), 17.6 (C3); **HRMS** (ESI) *m/z* found $[M+H]^+$ 115.0217, $C_5H_7O^{32}S^+$ required 115.0218. This data is consistent with that previously reported.[5]

2.3. Vinylsilane products

N,N-Diethyl-2-hydroxy-N-(4-(2-methyl-3-methylene-5,8,11,14-tetraoxa-2-silahexadecan-2-yl)benzyl)ethanammonium bromide (S16, Table 1, entry 4)

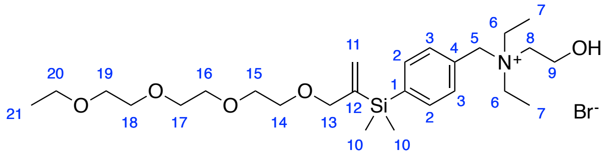

Hydrosilane **4** (65.0 mg, 0.188 mmol), alkyne **2** (27.0 mg, 0.125 mmol) and [Cp*Ru(MeCN)$_3$]PF$_6$ **1** (3.15 mg, 0.006 mmol) were dissolved in H_2O (1.3 mL). The reaction mixture was stirred at rt for 30 min. Upon completion, the solvent was removed *in vacuo* and the crude mixture was purified by FCC (1:19 to 1:4 MeOH:CH_2Cl_2) and to give **S16** as a viscous brown oil (64.0 mg, 91%). $\boldsymbol{R_f}$ 0.37 (3:17 MeOH:CH_2Cl_2); $\boldsymbol{\nu}_{max}$ (neat)/cm^{-1} 3298, 2864, 1631, 1456, 1400, 1249, 1102, 812, 776; $\boldsymbol{\delta_H}$ (500 MHz, $CDCl_3$) 7.63 (2H, app br s, 2 × H3), 7.43 (2H, app br s, 2 × H2), 5.91 (1H, s, H11–a), 5.49 (1H, s, H11–b), 4.61 (2H, br s, H5), 4.35 (2H, br s, H9), 4.06 (2H, s, H13), 3.45–3.63 (20H, m, 2 × H6, H8, H14–H20), 1.49 (6H, br s, 2 × H7), 1.19 (3H, t, J = 6.9 Hz, H21), 0.41 (6H, s, 2 × H10); $\boldsymbol{\delta_C}$ (126 MHz, $CDCl_3$) 146.4 (C4), 142.5 (C1), 135.3 (2 × C3), 131.8 (2 × C2), 127.9 (C11), 127.0 (C12), 75.4 (C13), 70.8 (1 C from C14–C20), 70.7 (2 C from C14–C20), 70.6 (1 C from C14–C20), 69.9 (1 C from C14–C20), 69.6 (1 C from C14–C20), 66.8 (1 C from C14–C20), 62.4 (C5), 59.3 (C9), 56.0 (C8), 53.8 (2 × C6), 15.2 (2 × C7), 8.3 (C21), –3.1 (2 × C10); **HRMS** (ESI) *m/z* found [M–Br]$^+$ 482.3323, $C_{26}H_{48}NO_5{}^{28}Si^+$ required 482.3302.

GSP for hydrosilylation (Table 1 (entries 8–9) and Table 2)

GSP 3

[RuCp*(MeCN)$_3$]PF$_6$ **1** (0.05 equiv.) in PBS buffer at pH 7.4 (1 mL) was added to a solution of silane (1.5 equiv.) and alkyne (1 equiv.) in 1 mL *t*BuOH at 37 °C. The reaction mixture was stirred for 30 min to 2 h. CH_2Cl_2 (2 mL) and H_2O (2 mL) were added to quench the reaction and the mixture was extracted with CH_2Cl_2 (3 × 2 mL). The combined organic layers were dried (Na_2SO_4), concentrated *in vacuo* and purified by FCC.

GSP 4

[RuCp*(MeCN)$_3$] **1** (0.05 equiv.) and hippuric acid (0.1 equiv.) in PBS buffer at pH 7.4 (1 mL) was stirred and the resulting catalytic system [Ru-hippuric] formed *in situ* was added directly to a solution of silane (1.5 equiv.) and alkyne (1 equiv.) in 1 mL *t*BuOH at 37 °C. The reaction mixture was stirred for 30 min to 2 h. CH_2Cl_2 (2 mL) and H_2O (2 mL) were added to quench the reaction and the mixture was extracted with CH_2Cl_2 (3 × 2 mL). The combined organic layers were dried (Na_2SO_4), concentrated *in vacuo* and purified by FCC.

***N*-(2-(2-(2-Hydroxyethoxy)ethoxy)ethyl)-4-(2-methyl-3-methylene-5,8,11,14-tetraoxa-2-silahexadecan-2-yl)benzamide (S17, Table 1, entries 8–9)**

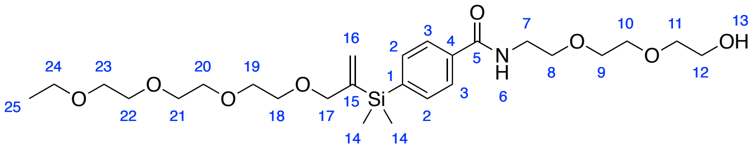

Alkyne **2** (21.6 mg, 100 μmol), silane **8** (46.7 mg, 150 μmol) and [Cp*Ru(MeCN)$_3$]PF$_6$ **1** (2.52 mg, 5.00 μmol) were reacted according to GSP 3 and purified by FCC (1:49 to 1:24 MeOH:CH_2Cl_2) to give **S17** as a pale yellow oil (52.2 mg, 99 μmol, 99%). Alternatively, alkyne **2** (21.6 mg, 100 μmol), silane **8** (46.7 mg, 150 μmol), [Cp*Ru(MeCN)$_3$]PF$_6$ **1** (2.52 mg, 5.00 μmol) and hippuric acid (1.79 mg, 10.0 μmol) were reacted in the presence of 0.2 mL plasma from human, 0.8 mL PBS at pH 7.4 and 1 mL *t*BuOH from human according to GSP 4. Instead of aqueous workup, the crude reaction mixture was lyophilised and purified by FCC (1:49 to 1:24 MeOH:CH_2Cl_2) to give **xx** as a pale yellow oil (37.4 mg, 71 μmol, 71%).

$\boldsymbol{R_f}$ 0.32 (1:19 MeOH:CH_2Cl_2); $\boldsymbol{\nu_{max}}$ (neat)/cm^{-1} 2862, 1640, 1542, 1303, 1104, 819; $\boldsymbol{\delta_H}$ (500 MHz, DMSO-*d*$_6$) 8.50 (1H, t, *J* = 5.6 Hz, H6), 7.81 (2H, d, *J* = 8.3 Hz, 2 × H3), 7.59 (2H, d, *J* = 8.3 Hz, 2 × H2), 5.84–5.86 (1H, m, H16–a), 5.42–5.43 (1H, m, H16–b), 4.57 (1H, t, *J* = 5.5 Hz, H13), 4.01 (2H, t, *J* = 1.5 Hz, H17), 3.39–3.55 (26H, m, H7–H12, H18–H24), 1.08 (3H, t, *J* = 7.0 Hz, H25), 0.38 (6H, s, 2 × H14); $\boldsymbol{\delta_C}$ (126 MHz, DMSO-*d*$_6$) 166.3 (C5), 147.0 (C15), 141.3 (C4), 134.9 (C1), 133.7 (2 × C2), 126.5 (C16), 126.3 (2 × C3), 74.3 (C17), 72.3 (1 C from C8–C12 or C18–C24), 69.8 (3 C from C8–C12 or C18–C24), 69.7 × 3 (3 C from C8–C12 or C18–C24), 69.2 (1 C from

C8–C12 or C18–C24), 69.1 (1 C from C8–C12 or C18–C24), 68.9 (1 C from C8–C12 or C18–C24), 65.5 (1 C from C8–C12 or C18–C24), 60.2 (1 C from C8–C12 or C18–C24), 39.0–40.0 (C7, unresolved due to overlap with solvent peak), 15.1 (C25), –3.2 (2 × C14); **HRMS** (ESI) *m/z* found $[M+Na]^+$ 550.2788, $C_{26}H_{45}NO_8{}^{28}SiNa^+$ required 550.2812.

_N_-(4-((4-((2-(2-(2-Hydroxyethoxy)ethoxy)ethyl)carbamoyl)phenyl)dimethylsilyl)pent-4-en-1-yl)-3,4-dimethoxybenzamide (10)

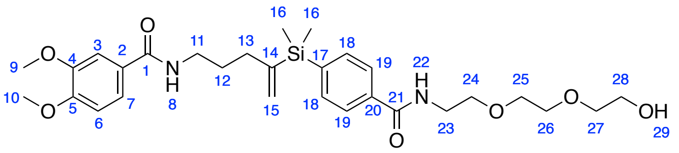

Alkyne **9** (24.7 mg, 100 µmol), silane **8** (46.7 mg, 150 µmol) and $[Cp^*Ru(MeCN)_3]PF_6$ **1** (2.52 mg, 5.00 µmol) were reacted according to GSP 3 and purified by FCC (3:97 to 1:19 MeOH:CH_2Cl_2) to give **10** as a pale yellow oil (38.0 mg, 68 µmol, 68%).

R_f 0.16 (1:19 MeOH:CH_2Cl_2); $\boldsymbol{\nu}_{max}$ (neat)/cm^{-1} 3325, 2933, 1633, 1508, 1269, 1130, 818; $\boldsymbol{\delta}_{\mathbf{H}}$ (500 MHz, DMSO-d_6) 8.49 (1H, t, *J* = 5.6 Hz, H22), 8.28 (1H, t, *J* = 5.6 Hz, H8), 7.80 (2H, d, *J* = 8.2 Hz, 2 × H19), 7.56 (2H, d, *J* = 8.2 Hz, 2 × H18), 7.41 (1H, dd, *J* = 8.4, 2.1 Hz, H7), 7.39 (1H, d, *J* = 2.0 Hz, H3), 6.99 (1H, d, *J* = 8.5 Hz, H6), 5.73 (1H, m, H15–a), 5.41 (1H, m, H15–b), 4.57 (1H, t, *J* = 5.5 Hz, H29), 3.79 (3H, s, H10), 3.78 (3H, s, H9), 3.39–3.55 (12H, m, H23–28), 3.18 (2H, dt, *J* = 6.6, 6.5 Hz, H11), 2.12 (2H, t, *J* = 7.8 Hz, H13), 1.57 (2H, tt, *J* = 7.5, 7.4 Hz, H12), 0.37 (6H, s, 2 × H16); $\boldsymbol{\delta}_{\mathbf{C}}$ (126 MHz, DMSO-d_6) 166.3 (C21), 165.6 (C1), 151.1 (C5), 149.1 (C14), 148.2 (C4), 141.5 (C17), 135.0 (C20), 133.6 (2 × C18), 127.0 (C2), 126.4 (2 × C19, C15), 120.2 (C7), 110.8 (C6), 110.6 (C3), 72.3 (1 C from C24–C27), 69.7 × 2 (2 C from C24–C27), 68.9 (1 C from C24–C27), 60.2 (C28), 55.6 (C9 or C10), 55.5 (C9 or C10), 39.0–40.0 (C11, C23, unresolved due to overlap with solvent peak), 32.9 (C13), 28.6 (C12), –3.1 (2 × C16); **HRMS** (ESI) *m/z* found $[M+H]^+$ 559.2852, $C_{29}H_{43}N_2O_7{}^{28}Si^+$ required 559.2840.

N-(2-(4-((2-(2-(2-Hydroxyethoxy)ethoxy)ethyl)carbamoyl)phenyl)-2-methyl-3-methylene-5,8,11-trioxa-2-silatridecan-13-yl)-3,4-dimethoxybenzamide (14)

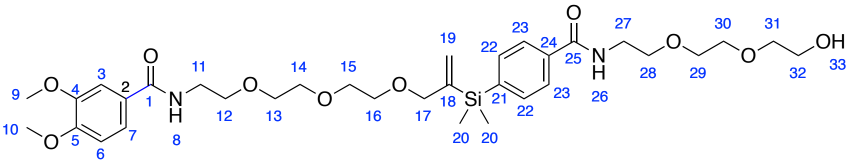

Alkyne **13** (35.1 mg, 100 µmol), silane **8** (46.7 mg, 150 µmol) and [Cp*Ru(MeCN)$_3$]PF$_6$ **1** (2.52 mg, 5.00 µmol) were reacted according to GSP 3 and purified by FCC (3:97 to 1:19 MeOH:CH$_2$Cl$_2$) to give **14** as a pale yellow oil (60.3 mg, 91.0 µmol, 91%).

R$_f$ 0.11 (1:19 MeOH:CH$_2$Cl$_2$); ***ν$_{max}$*** (neat)/cm^{-1} 3337, 2867, 1636, 1542, 1508, 1268, 1103, 1024, 818; ***δ*$_H$** (500 MHz, DMSO-d_6) 8.50 (1H, t, J = 5.6 Hz, H26), 8.37 (1H, t, J = 5.6 Hz, H8), 7.81 (2H, d, J = 8.2 Hz, 2 × H23), 7.57 (2H, d, J = 8.3 Hz, 2 × H22), 7.46 (1H, dd, J = 8.4, 2.1 Hz, H7), 7.43 (1H, d, J = 2.0 Hz, H3), 6.99 (1H, d, J = 8.5 Hz, H6), 5.82–5.84 (1H, m, H19–a), 5.41 (1H, m, H19–b), 4.57 (1H, t, J = 5.5 Hz, H33), 3.98 (2H, t, J = 1.4 Hz, H17), 3.79 (3H, s, H10), 3.78 (3H, s, H9), 3.36–3.55 (24H, m, H11–H16, H27–H32), 0.37 (6H, s, 2 × H20); ***δ*$_C$** (126 MHz, DMSO-d_6) 166.3 (C25), 165.8 (C1), 151.1 (C5), 148.2 (C4), 147.0 (C18), 141.3 (C21), 134.9 (C24), 133.7 (2 × C22), 126.7 (C2), 126.6 (C19), 126.3 (2 × C23), 120.4 (C7), 110.8 (C6), 110.6 (C3), 74.2 (C17), 72.3 (1 C from C12–C16 or C28–C32), 69.8 (1 C from C12–C16 or C28–C32), 69.7 × 2 (4 C from C12–C16 or C28–C32), 69.1 (1 C from C12–C16 or C28–C32), 69.0 (1 C from C12–C16 or C28–C32), 68.9 (1 C from C12–C16 or C28–C32), 60.2 (1 C from C12–C16 or C28–C32), 55.6 (C9 or C10), 55.5 (C9 or C10), 39.0–40.0 (C11, 27, unresolved due to overlap with solvent peak), –3.2 (2 × C20); **HRMS** (ESI) m/z found [M+H]$^+$ 663.3325, C$_{33}$H$_{51}$N$_2$O$_{10}$^{28}Si$^+$ required 663.3313.

Methyl *N*-(Boc)-*O*-(2-(dimethyl(phenyl)silyl)allyl)-L-serinate (17)

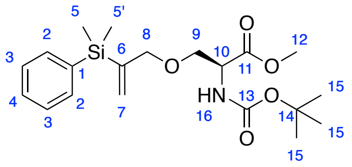

Alkyne **16** (25.7 mg, 100 µmol), silane **15** (20.4 mg, 150 µmol), hippuric acid (1.79 mg, 10.0 µmol) and [Cp*Ru(MeCN)$_3$]PF$_6$ **1** (2.52 mg, 5.00 µmol) were reacted according to GSP 4 and purified by FCC (7:93 to 1:9 EtOAc:PE) to give **17** as a clear oil (38.5 mg, 97.8 µmol, 98%).

***R**$_f$* 0.42 (1:4 EtOAc:PE); **[α]$_D$^{20}** +7.0 (*c* = 0.10 in MeOH); ***ν**$_{max}$* (neat)/cm^{-1} 2956, 1751, 1717, 1499, 1366, 1248, 1208, 1164, 1110, 819; ***δ**$_H$* (500 MHz, CDCl$_3$) 7.49–7.51 (2H, m, 2 × H2), 7.34–7.37 (3H, m, 2 × H3, H4), 5.82–5.83 (1H, m, H7–a), 5.46–5.47 (1H, m, H7–b), 5.24 (1H, d, *J* 8.6 Hz, H16), 4.37–4.40 (1H, m, H10), 4.03 (2H, s, H8), 3.78 (1H, dd, *J* = 9.4, 3.0 Hz, H9–a), 3.72 (3H, s, H12), 3.54 (1H, dd, *J* = 9.4, 3.3 Hz, H9–b), 1.45 (9H, s, 3 × H15), 0.37 (3H, s, H5 or H5'), 0.37 (3H, s, H5 or H5'); ***δ**$_C$* (126 MHz, CDCl$_3$) 171.3 (C11), 155.6 (C13), 146.7 (C6), 137.8 (C1), 134.0 (2 × C2), 129.3 (C4), 128.0 (2 × C3), 127.3 (C7), 80.0 (C14), 75.8 (C8), 70.5 (C9), 54.1 (C10), 52.5 (C12), 28.5 (3 × C15), –2.9 (C5, C5'); **HRMS** (ESI) *m/z* found [M+H]$^+$ 394.2044, $C_{20}H_{32}NO_5{}^{28}Si^+$ required 394.2050.

Methyl *N*-(Boc)-*S*-(2-(dimethyl(phenyl)silyl)allyl)-L-cysteinate (19)

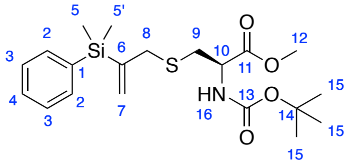

Alkyne **18** (27.3 mg, 100 µmol), silane **15** (20.4 mg, 150 µmol), hippuric acid (1.79 mg, 10.0 µmol) and [Cp*Ru(MeCN)$_3$]PF$_6$ **1** (2.52 mg, 5.00 µmol) were reacted according to GSP 4 and purified by FCC (7:93 to 1:9 EtOAc:PE) to give **19** as a clear oil (12.0 mg, 29.3 µmol, 29%).

***R**$_f$* 0.39 (1:4 EtOAc:PE); **[α]$_D$^{20}** –17.9 (*c* = 0.33 in MeOH); ***ν**$_{max}$* (neat)/cm^{-1} 2956, 1748, 1716, 1499, 1366, 1248, 1230, 1165, 817; ***δ**$_H$* (400 MHz, CDCl$_3$) 7.51–7.54 (2H, m, 2 ×

H2), 7.34–7.37 (3H, m, 2 × H3, H4), 5.77–5.78 (1H, m, H7–a), 5.50 (1H, d, *J* = 2.4 Hz, H7–b), 5.23 (1H, d, *J* = 7.7 Hz, H16), 4.46 (1H, q, *J* = 5.9 Hz, H10), 3.72 (3H, s, H12), 3.22 (2H, d, *J* = 3.3 Hz, H8), 2.77 (1H, dd, *J* = 13.9, 4.7 Hz, H9–a), 2.71 (1H, dd, *J* = 13.8, 5.5 Hz, H9–b), 1.44 (9H, s, 3 × H15), 0.43 (6H, s, 2 × H5); ***δ*$_C$** (101 MHz, $CDCl_3$) 171.8 (C11), 155.2 (C13), 145.5 (C6), 137.7 (C1), 134.1 (2 × C2), 129.4 (C7), 129.3 (C4), 127.9 (2 × C3), 80.2 (C14), 53.2 (C10), 52.6 (C12), 38.8 (C8), 33.3 (C9), 28.4 (3 × C15), –2.4 (C5 or C5'), –2.5 (C5 or C5'); **HRMS** (ESI) *m/z* found $[M+Na]^+$ 432.1658, $C_{20}H_{31}NO_4{}^{32}S^{28}SiNa^+$ required 432.1641.

Methyl 16-acetamido-2-(4-((2-(2-(2-hydroxyethoxy)ethoxy)ethyl)carbamoyl) phenyl)-2-methyl-3-methylene-5,8,11-trioxa-14-thia-2-silaheptadecan-17-oate (22)

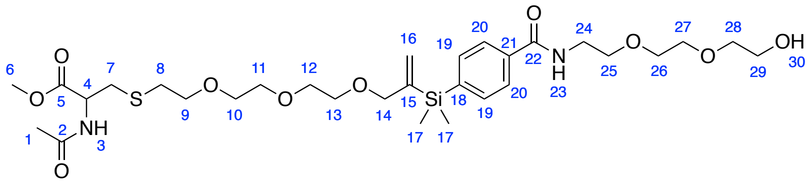

Alkyne **21** (34.7 mg, 100 µmol), silane **8** (46.7 mg, 150 µmol), $[Cp^*Ru(MeCN)_3]PF_6$ **1** (2.52 mg, 5 µmol) and hippuric acid (1.79 mg, 10.0 µmol) were reacted according to GSP 4 and purified by FCC (3:97 to 1:19 MeOH:CH_2Cl_2) to give **22** as a pale yellow oil (56.0 mg, 85.0 µmol, 85%).

R$_f$ 0.15 (1:19 MeOH:CH_2Cl_2); ***ν*$_{max}$** (neat)/cm^{-1} 3302, 2868, 1746, 1642, 1539, 1104, 819; ***δ*$_H$** (500 MHz, $CDCl_3$) 7.77 (2H, d, *J* = 8.2 Hz, 2 × H19), 7.60 (2H, d, *J* = 8.2 Hz, 2 × H20), 6.93 (1H, s, H23), 6.68 (1H, d, *J* = 7.5 Hz, H3), 5.89–5.90 (1H, m, H16–a), 5.49 (1H, m, H16–b), 4.78–4.82 (1H, m, H4), 4.05 (2H, t, *J* = 1.3 Hz, H14), 3.74 (3H, s, H6), 3.44–3.72 (22H, m, H9–H13, H24–H29), 3.03 (2H, d, *J* = 5.2 Hz, H7), 2.66–2.76 (3H, m, H8, H30), 2.03 (3H, s, H1), 0.41 (6H, s, 2 × H17); ***δ*$_C$** (126 MHz, $CDCl_3$) 171.4 (C5), 170.2 (C2), 167.8 (C22), 146.8 (C15), 142.5 (C18), 135.0 (C21), 134.3 (2 × C20), 127.5 (C16), 126.2 (2 × C19), 75.4 (C14), 72.7 (1 C from C9–C13 or C25–C28), 71.3 (1 C from C9–C13 or C25–C28), 70.7 (1 C from C9–C13 or C25–C28), 70.6 (1 C from C9–C13 or C25–C28), 70.5 (3 C from C9–C13 or C25–C28), 70.2 (1 C from C9–C13 or C25–C28), 69.6 (1 C from C9–C13 or C25–C28), 61.9 (C29), 52.7 (C6), 52.5 (C4), 39.9 (C24), 34.7 (C7), 32.5 (C8), 23.2 (C1), –3.0 (2 × C17); **HRMS** (ESI) *m/z* found $[M+H]^+$ 659.3008, $C_{30}H_{51}N_2O_{10}{}^{32}S^{28}Si^+$ required 659.3034.

Methyl N^2-(Boc)-N^6-(((2-((4-((2-(2-(2-hydroxyethoxy)ethoxy)ethyl)carbamoyl) phenyl)dimethylsilyl)allyl)oxy)carbonyl)-L-lysinate (24)

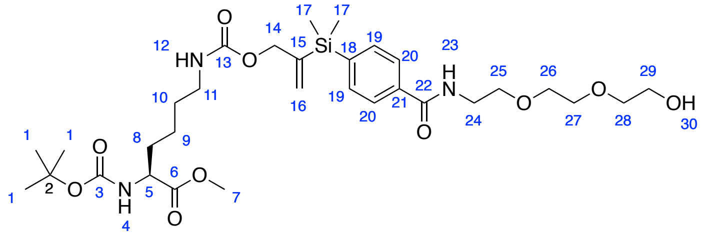

Alkyne **23** (34.2 mg, 100 μmol), silane **8** (46.7 mg, 150 μmol) and [Cp*Ru(MeCN)$_3$]PF$_6$ **1** (2.52 mg, 5.00 μmol) and hippuric acid (1.79 mg, 10.0 μmol) were reacted according to GSP 4 and purified by FCC (1:24 MeOH:CH_2Cl_2) to give **24** as a pale yellow oil (52.3 mg, 80.0 μmol, 80%).

R$_f$ 0.20 (1:24 MeOH:CH_2Cl_2); **$[\alpha]_D^{20}$** –5.0 (*c* = 0.10 in MeOH); ***ν$_{max}$*** (neat)/cm^{-1} 3331, 2932, 1700, 1646, 1533, 1366, 1250, 1164, 1105, 819; ***δ*$_H$** (500 MHz, DMSO-*d*$_6$) 7.97 (1H, s, H23), 7.80 (2H, d, *J* = 8.2 Hz, 2 × H20), 7.59 (2H, d, *J* = 8.1 Hz, 2 × H19), 6.50 (2H, br s, H4, H12), 5.89 (1H, m, H16–a), 5.48 (1H, m, H16–b), 4.59 (2H, s, H14), 3.95–3.99 (2H, m, H5, H30), 3.65 (3H, s, H7), 3.44–3.61 (12H, m, H24–H29), 2.95–2.99 (2H, m, H11), 1.58–1.72 (2H, m, H8), 1.28–1.44 (13H, m, 3 × H1, H9, H10), 0.42 (6H, s, 2 × H17); ***δ*$_C$** (126 MHz, DMSO-*d*$_6$) 173.2 (C6), 166.3 (C22), 155.8 (C13), 155.6 (C3), 145.6 (C15), 140.7 (C18), 135.1 (C21), 133.7 (2 × C19), 126.4 (2 × C20), 126.1 (C16), 78.2 (C2), 72.3 (1 C from C25–C28), 69.7 × 2 (2 C from C25–C28), 68.9 (1 C from C25–C28), 66.3 (C14), 60.2 (C29), 53.5 (C5), 51.7 (C7), 39.0–40.0 (C11, C24, unresolved due to overlap with solvent peak), 30.3 (C8), 28.9 (C10), 28.2 (3 × C1), 22.8 (C9), –3.3 (2 × C17); **HRMS** (ESI) *m/z* found $[M+Na]^+$ 676.3215, $C_{31}H_{51}N_3O_{10}{}^{28}SiNa^+$ required 676.3241.

4-(Dimethyl(3-(((2*R*,3*R*,4*S*,5*S*,6*R*)-3,4,5-trihydroxy-6-(hydroxymethyl)-tetra hydro-2*H*-pyran-2-yl)oxy)prop-1-en-2-yl)silyl)-*N*-(2-(2-(2-hydroxyethoxy) ethoxy)ethyl) benzamide (26)

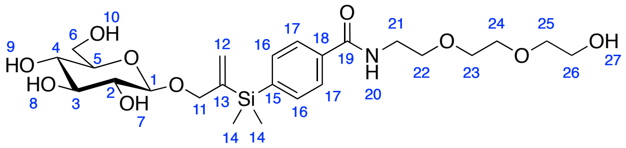

Alkyne **25** (21.8 mg, 100 µmol), silane **8** (46.7 mg, 150 µmol) and [Cp*Ru(MeCN)$_3$]PF$_6$ **1** (2.52 mg, 5.00 µmol) were reacted according to GSP 3. Instead of aqueous workup, the crude reaction mixture was lyophilised and purified by FCC (1:19 to 1:4 MeOH:CH$_2$Cl$_2$) to give **26** as a pale yellow wax (42.9 mg, 81.0 µmol, 81%).

R$_f$ 0.14 (1:10 MeOH:CH$_2$Cl$_2$); **[α]$_D$^{20}** –16.0 (c = 0.47 in MeOH); ***ν$_{max}$*** (neat)/cm^{-1} 3332, 2929, 1636, 1543, 1309, 1250, 1072, 1025, 818; ***δ*$_H$** (500 MHz, DMSO-d_6) 8.51 (1H, t, J = 5.6 Hz, H20), 7.81 (2H, d, J = 8.2 Hz, 2 × H16), 7.60 (2H, d, J = 8.2 Hz, 2 × H17), 6.00–6.01 (1H, m, H12–a), 5.42–5.43 (1H, m, H12–b), 4.97 (1H, d, J = 5.1 Hz, H7), 4.92 (1H, d, J = 4.9 Hz, H8), 4.88 (1H, d, J = 4.9 Hz, H9), 4.58 (1H, t, J = 5.5 Hz, H27), 4.45 (1H, t, J = 5.9 Hz, H10), 4.36 (1H, dt, J = 13.9, 1.6 Hz, H11–a), 4.13 (1H, d, J = 7.8 Hz, H1), 4.07 (1H, dt, J = 13.9, 1.6 Hz, H11–b), 3.63 (1H, dd, J = 11.0, 6.1 Hz, H6–a), 3.40–3.55 (13H, m, H6–b, H21–H26), 3.10–3.15 (1H, m, H3), 3.03–3.05 (2H, m, H4, H5), 2.97–3.01 (1H, m, H2), 0.40 (6H, s, 2 × H14); ***δ*$_C$** (126 MHz, DMSO-d_6) 166.4 (C19), 146.0 (C13), 141.2 (C15), 135.0 (C18), 133.8 (2 × C17), 126.3 (2 × C16), 126.2 (C12), 102.1 (C1), 76.9 (C3, C5), 73.6 (C2), 72.3 (1 C from C22–C25), 70.8 (C11), 70.0 (C4), 69.7 × 2 (2 C from C22–C25), 68.9 (1 C from C22–C25), 61.0 (C6), 60.2 (C26), 39.0–40.0 (C21, unresolved due to overlap with solvent peak), –3.3 (2 × C14); **HRMS** (ESI) m/z found [M+Na]$^+$ 552.2224, C$_{24}$H$_{39}$NO$_{10}$^{28}SiNa$^+$ required 552.2241.

2.4. Synthesis and hydrosilylation of peptide **27**

Fmoc Solid-Phase Peptide Synthesis

Peptide synthesis was carried out on solid-phase using a Fmoc-protecting group strategy on a CEM Liberty Automated Microwave Peptide Synthesiser using Merck Rink Amide MBHA resin LL (0.38 mmol/g). All peptide couplings were performed with Fmoc-protected amino acids (5 equiv.) in DMF, HATU (5 equiv.) in DMF, and *N*,*N*-diisopropylethylamine (10 equiv.) in NMP. Arginine was coupled using double couplings for 15 min each without microwave irradiation. All other amino acids were coupled using single couplings with 25 W power at 75 ºC for 15 min. The side chain protecting groups used were *t*-Bu for aspartic acid, glutamic acid, threonine, Boc for tryptophan, Pbf for arginine, and Trt for asparagine.

Fmoc deprotection was carried out with 20% piperidine in DMF, using 45 W power at 75 ºC over 3 min. N-terminal capping with acetyl was performed manually by treating the resin-bound peptide with acetic anhydride (10 equiv.) and *N*,*N*-diisopropylethylamine (10 equiv.) in CH_2Cl_2 for 1.5 h. Cleavage was achieved with a cocktail of trifluoroacetic acid (92.5%), triisopropylsilane (2.5%), H_2O (2.5%), CH_2Cl_2 (2.5%) for 2 h. The cleavage solution was then evaporated under a stream of nitrogen. The crude residue was triturated with Et_2O prior to purification by semi-preparative HPLC.

***N*[2]-(Fmoc)-*N*[6]-((prop-2-yn-1-yloxy)carbonyl)-L-Lys (X)**

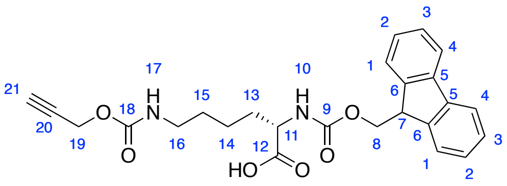

Propargyl chloroformate (267 µL, 2.74 mmol) in dioxane (50 mL) was added dropwise to a stirring solution of Fmoc-Lys-OH·HCl (1.00 g, 2.47 mmol) and $NaHCO_3$ (1.15 g, 13.7 mmol) in water (50 mL) at 0 ºC. After stirring for 3 h at rt, the dioxane was removed *in vacuo* and the remaining aqueous layer was acidified with 2 M aqueous HCl and extracted with EtOAc (3 × 30 mL). The combined organic extracts were washed with brine (50 mL), dried ($NaSO_4$) and concentrated *in vacuo* to

give amino acid **X** as a waxy off-white solid (867 mg, 78%). ***R****f* 0.34 (5:94:1 MeOH:CH_2Cl_2:acetic acid); **[α]D**[20] +10.0 (*c* = 0.10 in MeOH); ***δ*H** (400 MHz, DMSO-*d*6) 7.89 (2H, d, *J* = 7.5 Hz, 2 × H4), 7.69 (2H, d, *J* = 7.4 Hz, 2 × H1), 7.41 (2H, t, *J* = 7.4 Hz, 2 × H3), 7.30–7.34 (3H, m, 2 × H2, H17), 6.77 (1H, br s, H10), 4.58 (2H, d, *J* = 2.2 Hz, H19), 4.19–4.33 (3H, m, H7, H8), 3.70–3.71 (1H, m, H11), 3.46 (1H, t, *J* = 2.4 Hz, H21), 2.94 (2H, q, *J* = 6.5 Hz, H16), 1.65–1.71 (1H, m, H13–a), 1.49–1.56 (1H, m, H13–b), 1.34–1.38 (2H, m, H15), 1.23–1.27 (2H, m, H14); ***δ*C** (126 MHz, DMSO-*d*6) 175.3 (C12), 155.6 (C9 or C18), 155.3 (C9 or C18), 144.1 (2 × C6), 140.8 (2 × C5), 127.7 (2 × C3), 127.2 (2 × C2), 125.3 (2 × C1), 120.2 (2 × C4), 79.6 (C20), 77.0 (C21), 65.4 (C8), 55.3 (C11), 51.4 (C19), 46.8 (C7), 40.5 (C16), 32.0 (C13), 29.4 (C15), 22.6 (C14); **HRMS** (ESI) *m/z* found $[M+Na]^+$ 473.1676, $C_{25}H_{26}N_2O_6Na^+$ required 473.1683. This data is consistent with that previously reported.[6]

Peptide Characterisation

Peptide **27** Sequence: Ac-ETF**X**DLWRLLPEN-NH_2, where **X** is *N*[6]-((prop-2-yn-1-yloxy)carbonyl)-L-Lys.

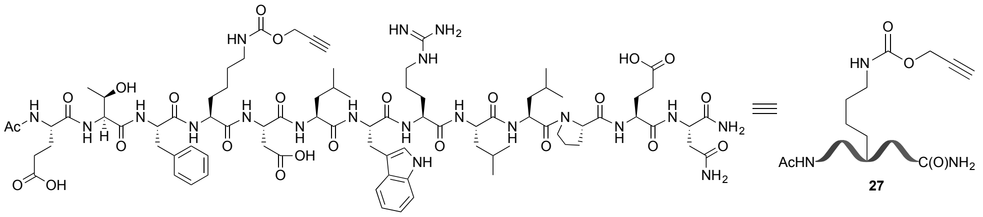

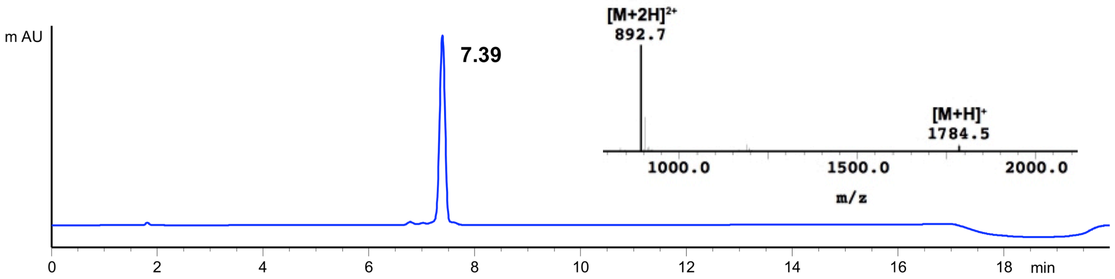

Analytical HPLC (30 to 60% B over 15 minutes, t_r = 7.39 min) of purified **27**. LC–MS (ESI) *m/z* found $[M+2H]^{2+}$ 892.7, required 892.5.

Hydrosilylation of 27

To a solution of **27** (4.46 mg, 2.5 μmol) and biotinylated silane **29** (1.95 mg, 3.2 μmol) in MeOH (125 μL) was added **1** (1.89 mg, 0.4 μmol) and hippuric acid (0.1 mg, 0.8 μmol) in PBS buffer at pH 7.4 (125 μL) and stirred at 37 ºC for 30 min. A further addition of **29** (1.95 mg, 3.2 μmol) in MeOH (125 μL), **1** (1.89 mg, 0.4 μmol) and

hippuric acid (0.1 mg, 0.8 μmol) in PBS buffer at pH 7.4 (125 μL) was added to the reaction mixture, bringing the final concentration of peptide to 5 mM, with silane **29** (2.5 equiv.), **1** (30 mol%) and hippuric acid (0.6 equiv.) in 1:1 MeOH:PBS buffer at pH 7.4. The reaction mixture was stirred for a further 30 min and analysed by HPLC and LC–MS, showing full conversion (>95%) to product peptide **28**.

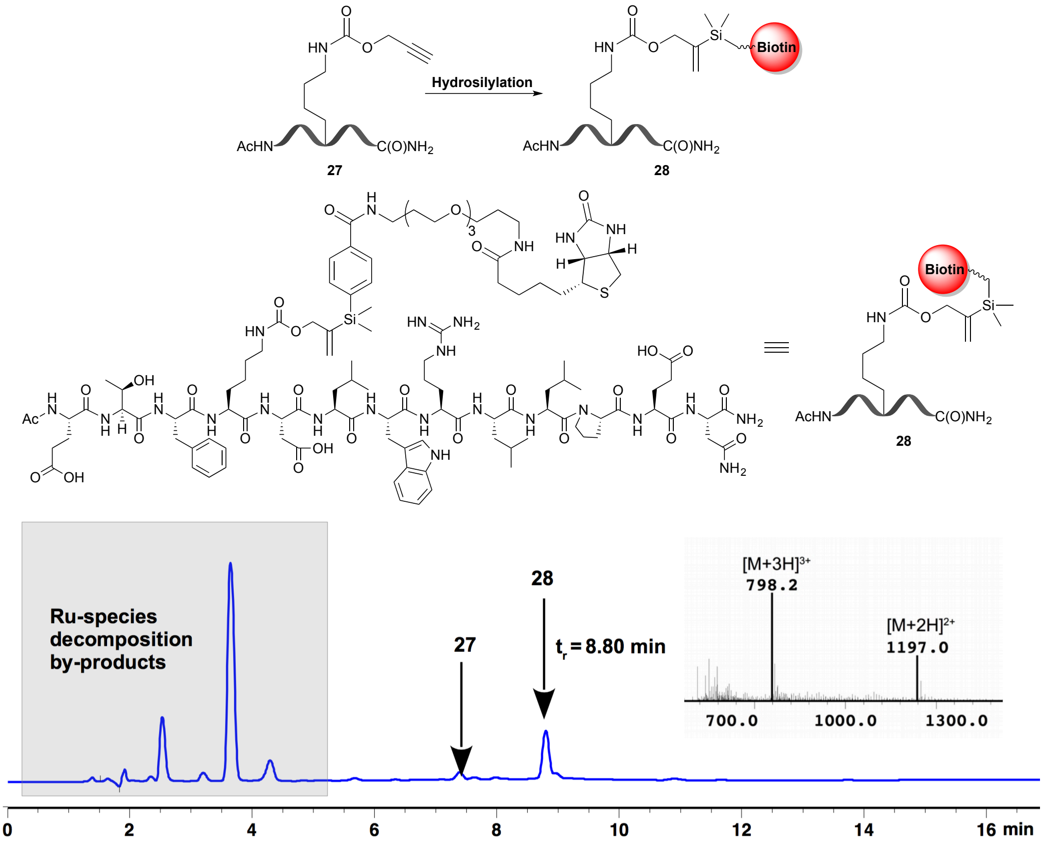

Hydrosilylation on **27** to give modified peptide **28**, and analytical HPLC (30 to 60% B over 15 min) of crude reaction mixture at t = 1 h. LC–MS (ESI) m/z found $[M+3H]^{3+}$ 798.2, required 798.4.

3. Protein modification

3.1. Preparation and hydrosilylation of **OP-Lyz**

Preparation of **OP-Lyz**

36 (11 mg, 0.05 mmol) was dissolved in DMSO (250 μL) and transferred directly to PBS buffer at pH 7.4 (2.25 mL) containing lysozyme (10 mg, Sigma). The mixture was agitated for 1 h and the protein was purified on a PD-10 desalting column (GE Healthcare Life Sciences). The column was pre-equilibrated with PBS buffer at pH 7.4 (25 mL), the protein mixture added to the column and eluted with PBS buffer at pH 7.4 (3.5 mL). Protein concentrations were assayed by a NanoDrop2000 (Thermo Scientific).

Western blot procedures

Proteins mixtures, as described in **Figure 1C & S1**, were prepared in 4 × sample buffer (containing 10 mM dithiothreitol (DTT) as the reducing agent) and boiled at 95 ºC for 10 min. Samples were loaded onto 12% SDS-PAGE gels and electrophoresed in running buffer at a constant voltage of 160 V. PageRuler Prestained Protein Ladder (Fermentas; 10–170 kDa) was used as an indicator of molecular weight.

Proteins separated on 12% SDS-PAGE gels were transferred to polyvinylidene fluoride (PVDF) membranes by wet transfer at a constant ampere of 250 mA in transfer buffer for 90 min at 4 ºC. Following 3 × 10 min washes in wash buffer, membranes were blocked for 15 h in 5% (w/v) non-fat dried milk/Tween PBS (PBST) at rt. Membranes were then incubated for 1 h with anti-biotin−Peroxidase antibody produced in goat (1:5000 dilution, Sigma) and washed 4 × in wash buffer. Lastly, membranes were developed using Immobilon^{TM} Western Chemiluminescent HRP Substrate (Millipore).

Coomassie staining of SDS-PAGE gels was performed by immersing the gel in Coomassie stain and placing on a rotating table overnight for staining to occur. The gel was then washed 4 × 10 min in Coomassie destain before an image of the gel was captured using the GeneGenius gel documentation system (Syngene, UK).

Ruthenium content

The ruthenium content after standard purification using Zeba^{TM} Spin desalting column (ThermoFisher Scientific) was determined to be <10 parts per billion (ppb) for reaction

with a catalyst loading of 10 mol% and 29 ppb at a catalyst loading of 50 mol%. Analysis was conducted by Butterworth Laboratories, 54-56 Waldegrave Road, Teddington, TW11 8NY, UK.

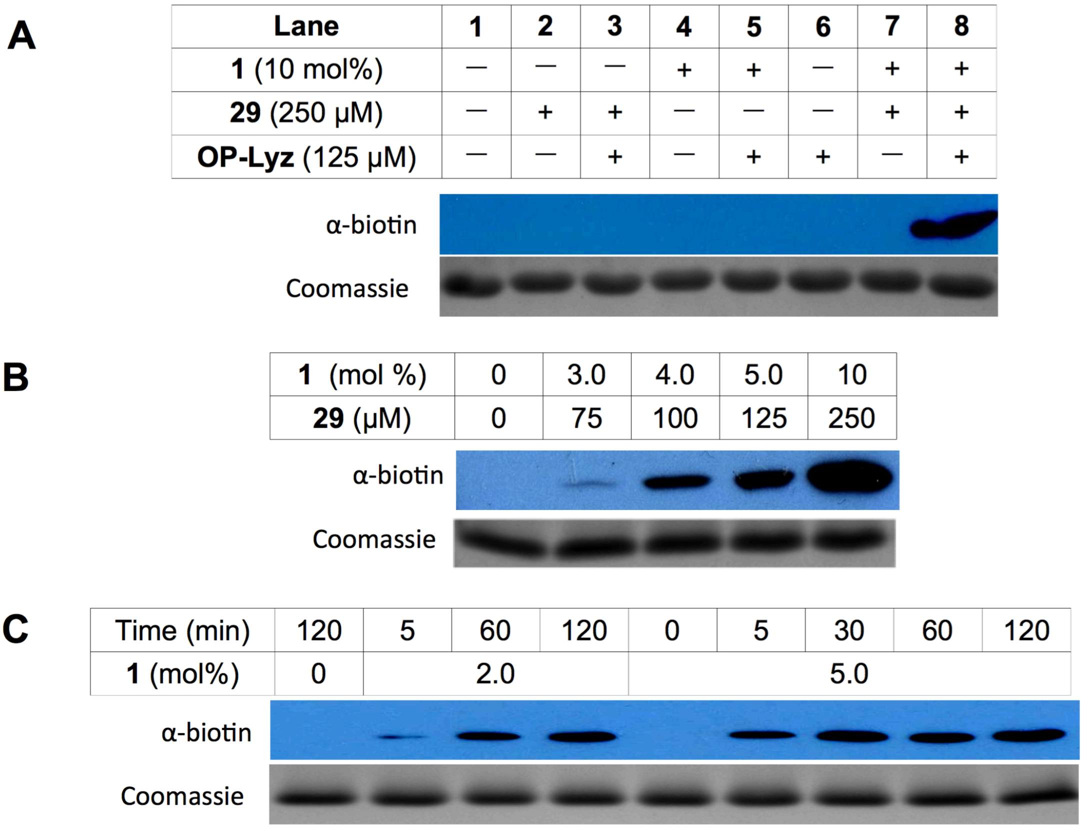

Figure S1. (A) Selective labeling of **OP-Lyz** with hydrosilane **29** and Ru cat. **1**. Different combination of reagents were treated with **Lyz** (–) or **OP-Lyz** (+) at 37 °C for 2 h. Only in the presence of **OP-Lyz**, **29** and **1** was a biotin-signal detected (Lane 8). (B) **29** and **1** displayed a dose-dependent labeling of **OP-Lyz** (125 μM) when reaction time was held constant at 2 h. (C) Time-dependent hydrosilylation labeling of **OP-Lyz** (125 μM) when **29** was held constant at 250 μM. The reaction mixtures were quenched by the addition of 100-fold alkyne **2**. Remarkably, even at low catalyst loading (2.0% and 5.0% w.r.t. protein concentration), labeling was observed. Equal protein loading was verified by Coomassie stain.

3.2. *sf*GFP protein expression and purification

To express *sf*GFP with incorporated unnatural amino acid **37**, we transformed *E. coli* DH10B cells with *p*BK-*Pyl*RS (which encodes wild-type pyrrolysine *t*RNA-synthetase) and p*sfGFP*150TAG*Pyl*T-His$_6$ (which encodes *Mb*tRNA$_{CUA}$ and a C-terminally hexahistidine tagged *sf*GFP gene with an amber codon at position 150). Cells were recovered in 1 mL of S.O.B. media (supplemented with 0.2% glucose) for 1 h at 37 ºC, before incubation (16 h, 37 ºC, 230 rpm) in 100 ml of LB containing kanamycin (50 μg/mL) and tetracyclin (25 μg/mL). 20 mL of this overnight culture was used to inoculate 100 mL of LB supplemented with kanamycin (25 μg/mL) and tetracyclin (12

μg/mL) and incubated at 37 ºC. At OD_{600} = 0.4–0.6, a solution of **37** in H_2O was added to a final concentration of 5 mM. After 30 min, protein expression was induced by the addition of arabinose to a final concentration of 0.2%. After 16 h of induction, cells were harvested by centrifugation and frozen at –80 ºC until required. Cells were thawed on ice and suspended in 30 mL of lysis buffer (10 mM Tris-HCl, 20 mM imidazole, 100 μg/mL DNaseA, 1 × Roche protease tablet and 20 mL BugBuster®) and rotated at rt for 20 min. The soluble extract was clarified by centrifugation (25 min, 21000 g, 4 ºC). 300 μL of pre-washed Ni^{2+}-NTA beads (Qiagen) were added to the extract and the mixture was incubated with agitation for 1 h at 4 ºC. Beads were collected by centrifugation (10 min, 1000 g) and resuspended in wash buffer (3 × 30 mL, 20 mM Tris-HCl, 30 mM imidazole, 300 mM NaCl, pH 8) and spun down at 100 g for 10 min. The beads were suspended in 10 mL of wash buffer and transferred to a column. The protein was eluted with 3 mL of wash buffer supplemented with 200 mM imidazole. The protein mixture was then buffer exchanged into PBS buffer at pH 7.4 through a PD-10 desalting column (GE Healthcare Life Sciences). The purity of the resultant protein mixture was analysed by 4–12% SDS-PAGE and their mass confirmed by mass spectrometry. Western blots were performed with an antibody against the hexahistidine tag (Cell Signalling Technology, His Tag 27E8 mouse mAb (HRP conjugate) #9991). *sf*GFP with incorporated Boc-lysine (**38**) was prepared in a similar fashion.

Wild-type *sf*GFP sequence with residue 150 highlighted

MVSKGEELFTGVVPILVELDGDVNGHKFSVRGEGEGDATNGKLTLKFICTTGK
LPVPWPTLVTTLTYGVQCFSRYPDHMKRHDFFKSAMPEGYVQERTISFKDDGT
YKTRAEVKFEGDTLVNRIELKGIDFKEDGNILGHKLEYNFNSH***N***VYITADKQKN
GIKANFKIRHNVEDGSVQLADHYQQNTPIGDGPVLLPDNHYLSTQSVLSKDPNE
KRDHMVLLEFVTAAGITHGMDELYKGSHHHHHH

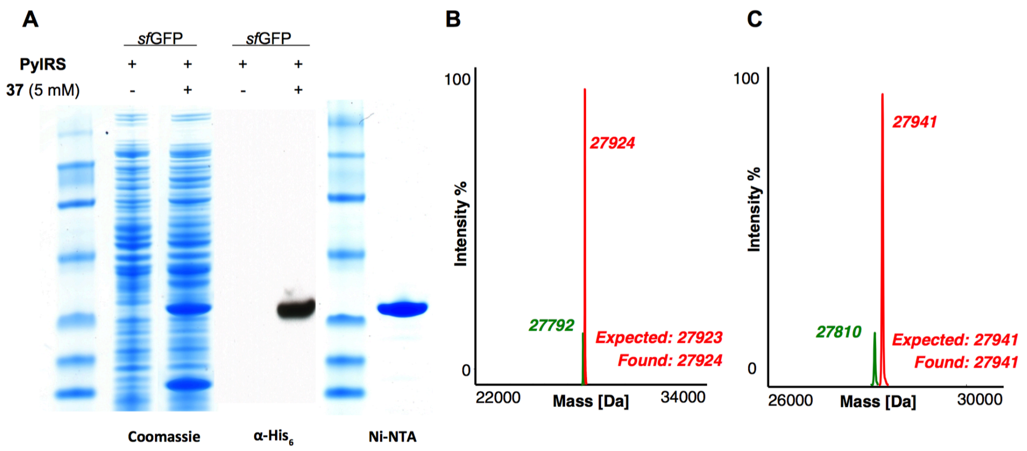

Figure S2. The genetic incorporation of **37** in *E. Coli*. (A) Amino acid dependent overexpression of *sf*GFP-$\mathbf{37}_{150}$. Protein was detected by Western blot with anti-His_6 antibody and Coomassie stain. *sf*GFP-$\mathbf{37}_{150}$ was purified using Ni-NTA beads. (B) and (C) ESI–MS data for **37** and **38** incorporation, respectively. The minor green peak represents proteolysis of the *N*-terminal methionine (–131 Da).

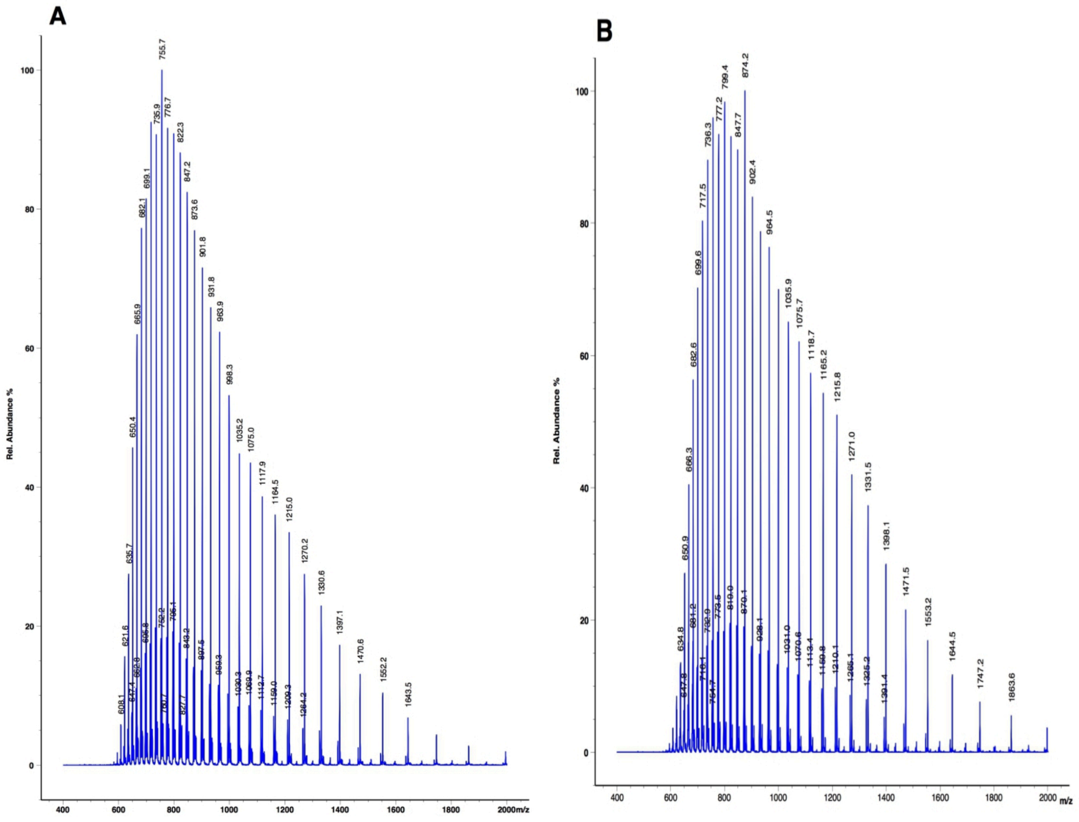

Figure S3. ESI–MS ionisation peaks of (A) *sf*GFP-$\mathbf{37}_{150}$ and (B) *sf*GFP-$\mathbf{38}_{150}$

3.3. Hydrosilylation of *sf*GFP-$\mathbf{37}_{150}$

Figure 1E

*sf*GFP-$\mathbf{37}_{150}$ or *sf*GFP-$\mathbf{38}_{150}$ (25 μM) was incubated with hydrosilane **39** (250 μM, 10 equiv.) and Ru cat. **1** (1.25 μM, 5 mol%) at 37 ºC for 24 h and quenched with alkyne **2** (0.5 M). The crude reaction mixtures were analysed by 4–12% SDS-PAGE to assess

protein levels. Gels were either Coomassie stained or scanned with a Typhoon imager in order to visualize fluorescent bands. Together with other control experiments, hydrosilylation was shown to be selective towards *O*-propargyl groups on the surface of GFP, despite the wide array of functionalities present within the biomolecule.

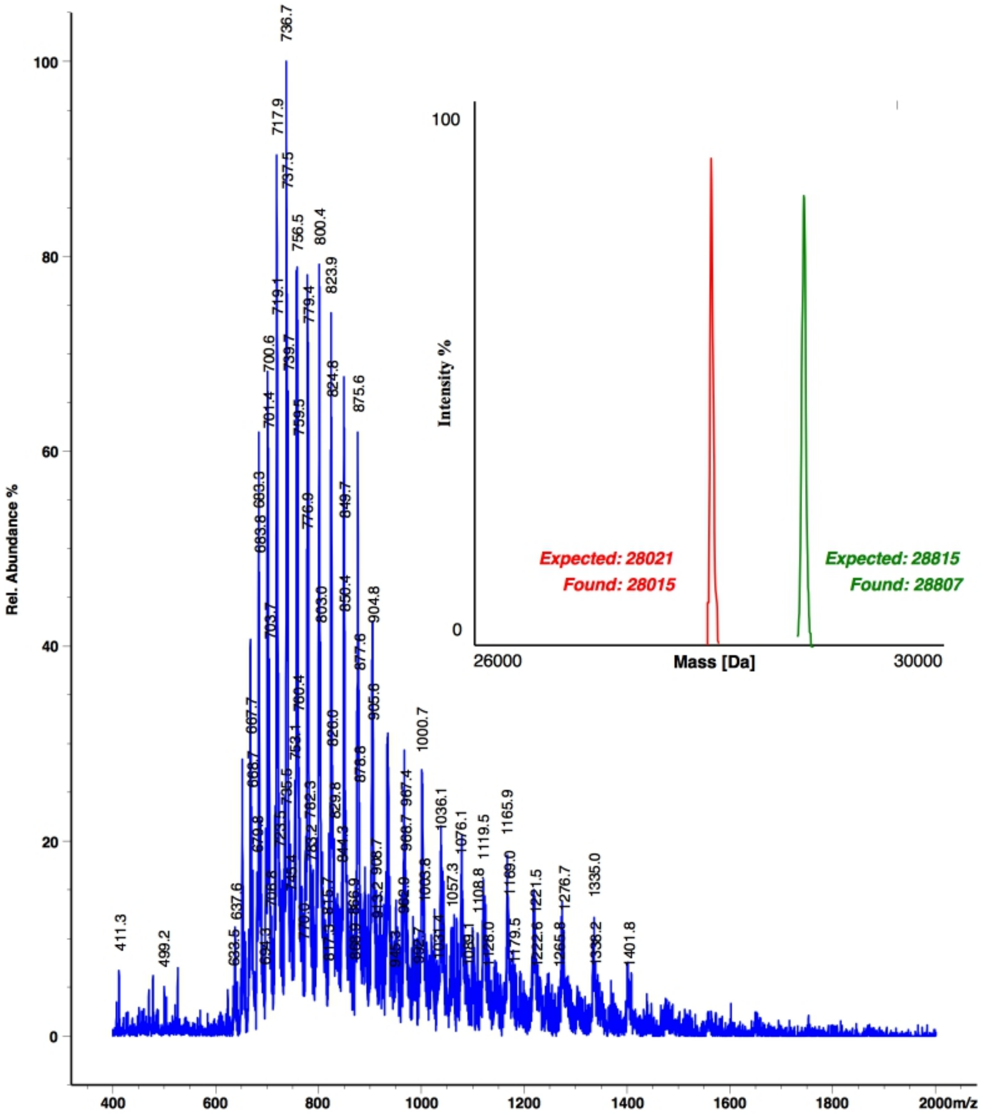

Figure S4. Crude reaction mixture after 24 h was analysed by LC–MS. The spectrum shows ~50% conversion to fluorescently-labelled *sf*GFP-**37**$_{150}$. The peak in red corresponds to *sf*GFP-**37**$_{150}$ with a phosphate adduct (+98), and the peak in green corresponds to ligated *sf*GFP with a phosphate adduct (+98).

3.4. Preparation and hydrosilylation of **SP-C2Am** and **OP-C2Am**

C2A Domain of Synaptotagmin-I (C2Am)[7]

Sequence of C2Am S78C (modified residue highlighted)

GSPGISGGGGGILDSMVEKLGKLQYSLDYDFQNNQLLVGIIQAAELPALDMGGT
SDPYVKVFLLPDKKKKFETKVHRKTLNPVFNEQFTFKVPYCELGGKTLVMAVY
D FDRFSKHDII GEFKVPMNTV DFGHVTEEWR DLQSAEK

Calculated average isotopic mass = 16222.53 (*N*-terminal Met cleaved)

A typical analysis of a conjugation reaction by LC–MS is described below. The total ion chromatogram, combined ion series, and deconvoluted spectra are shown for C2Am S78C (**C2Am**). Identical analyses were carried out for all the conjugation reactions performed in this work.

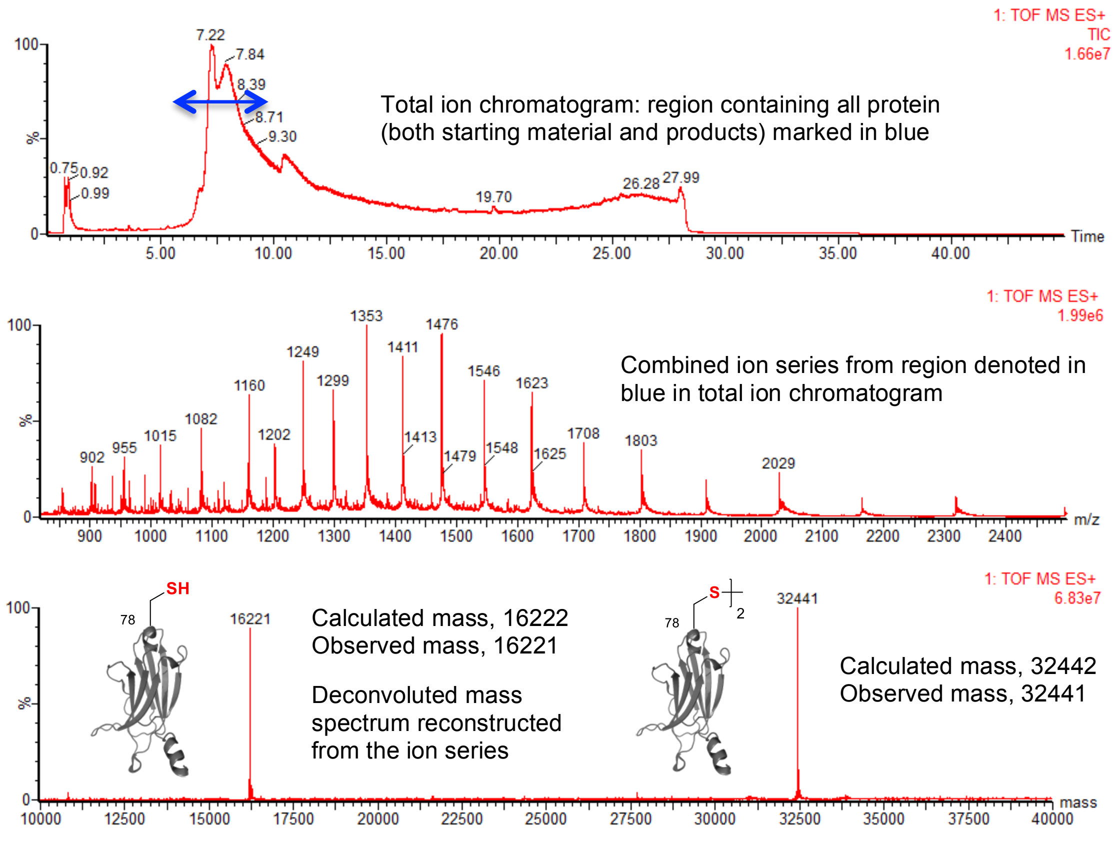

Figure S5. ESI–MS spectrum of non-reduced **C2Am**.

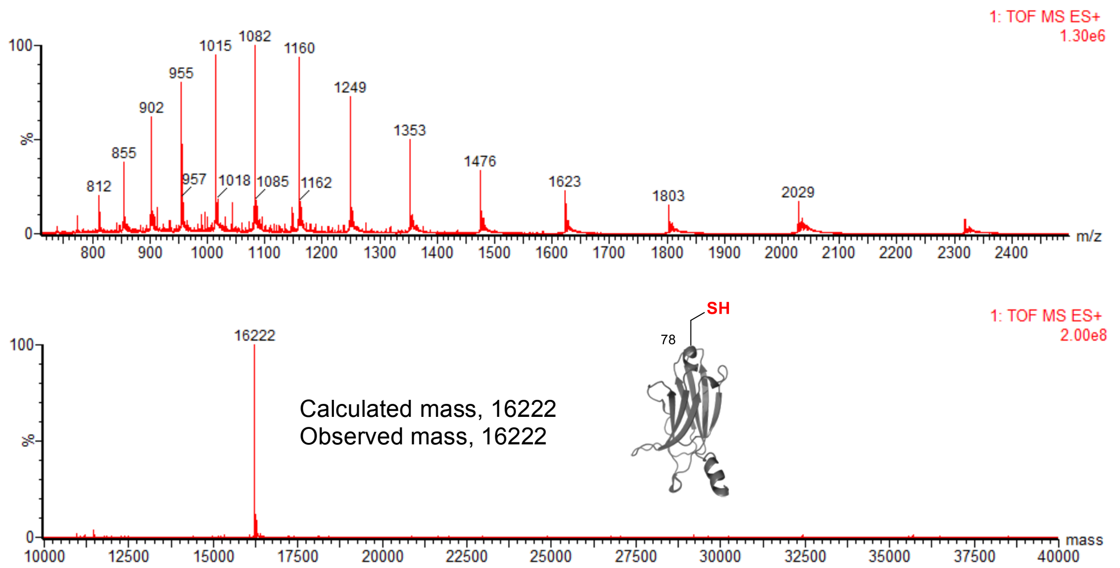

Figure S6. ESI–MS of reduced **C2Am**.

Control: reaction of Ellman's reagent with **C2Am**

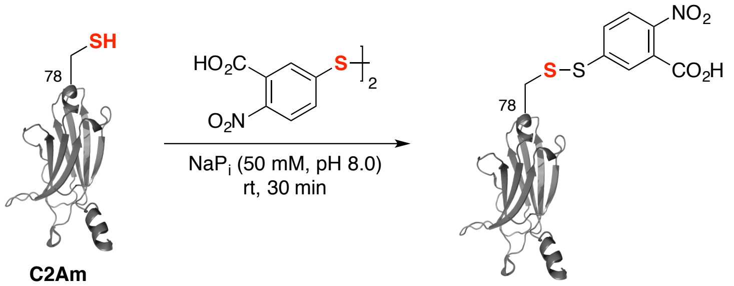

All manipulations were carried out at room temperature. D/L-Dithiothreitol (DTT) (0.11 mg, 770 nmol) was added as a solid to a solution of **C2Am** (25 µL, 1 mg/mL, 1.54 nmol) in 50 mM sodium phosphate buffer at pH 8.0 and the resulting mixture shaken for 30 min. Small molecules were removed from the reaction mixture by loading the sample onto a Zeba™ Spin desalting column (ThermoFisher Scientific) previously equilibrated with 50 mM sodium phosphate buffer at pH 8.0. The sample was eluted by centrifugation (2 min, 1500 × g). A 10 µL aliquot of **C2Am** (*ca.* 1 mg/mL, 0.616 nmol) was transferred to a 0.5 mL eppendorf tube. Ellman's reagent (0.3 µL of a 20 mg/mL stock solution in H_2O) was added and the resulting mixture vortexed for 30 seconds. After 30 min of additional shaking, a 2 µL aliquot was analysed by LC–MS (2 µL aliquot diluted by 8 µL of 50 mM sodium phosphate buffer at pH 8.0) and complete conversion to the expected Ellman's product (calculated mass, 16419; observed mass, 16419) was observed.

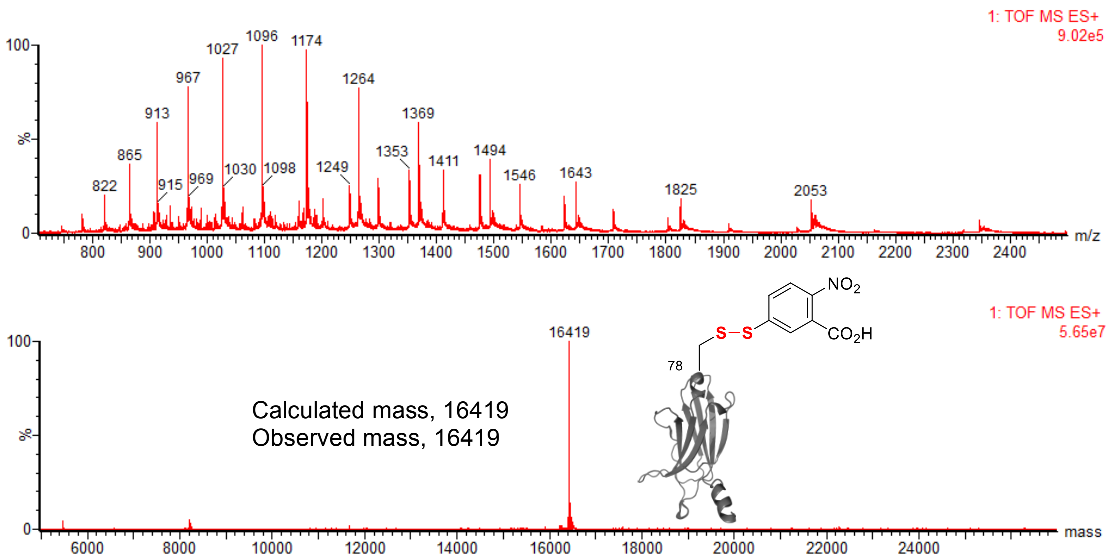

Figure S7. ESI–MS of the reaction of Ellman's reagent with **C2Am**.

<u>Reaction of α,α'-di-bromoadipyl(bis)amide with C2Am S78C (**C2Am**)</u>

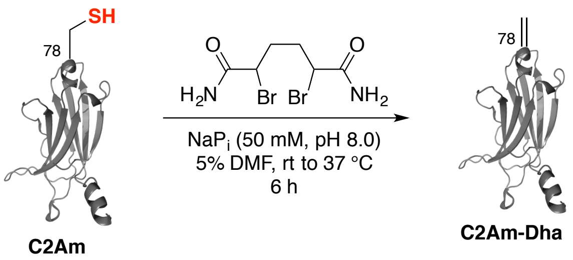

DTT (2.4 mg, 15.6 μmol) was added as a solid to a solution of **C2Am** (500 μL, 1 mg/mL, 30.8 nmol) in 50 mM sodium phosphate buffer at pH 8.0 and the resulting mixture shaken at rt for 1 h. Small molecules were removed from the reaction mixture by loading the sample onto a Zeba^{TM} Spin desalting column (ThermoFisher Scientific) previously equilibrated with 50 mM sodium phosphate buffer at pH 8.0. The sample was eluted by centrifugation (2 min, 1500 × g). A freshly prepared solution of α,α′-di-bromo-adipyl(bis)amide (14 mg, 46.2 μmol) in DMF (25 μL) was added and the resulting mixture vortexed for 30 seconds and then shaken at rt for 2 h. After 4 h of additional shaking at 37 ºC, a 3 μL aliquot was analysed by LC–MS (3 μL aliquot diluted by 7 μL of 50 mM sodium phosphate buffer at pH 8.0) and complete conversion to **C2Am-Dha** (calculated mass, 16188; observed mass, 16188) was observed. Small molecules were removed from the reaction mixture by loading the sample onto a Zeba^{TM} Spin desalting column (ThermoFisher Scientific) previously equilibrated with 50 mM sodium phosphate buffer at pH 8.0. The sample was eluted by centrifugation (2

min, 1500 × g). The protein sample (0.418 mg/mL by Bradford assay) was flash frozen with liquid nitrogen and stored at –20 ºC.

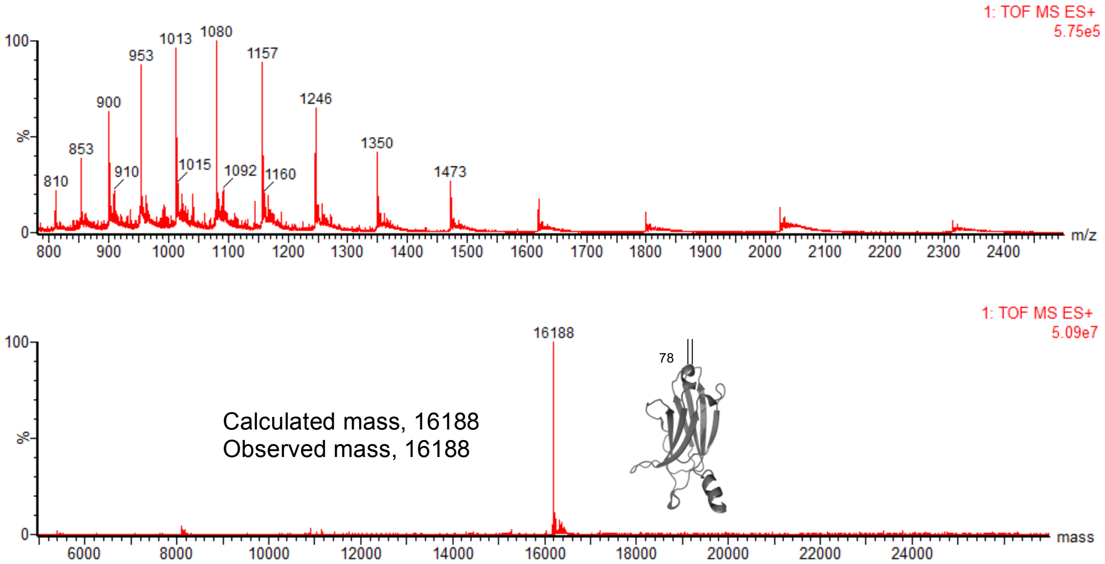

Figure S8. ESI–MS of the reaction of α-α'-di-bromoadipyl(bis)amide with **C2Am**.

<u>Control: reaction of Ellman's reagent with **C2Am-Dha**</u>

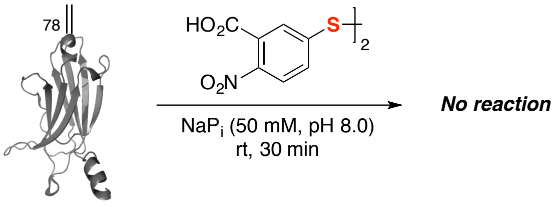

All manipulations were carried out at room temperature. A 10 µL aliquot **C2Am-Dha** (0.169 mg/mL, 0.104 nmol) in 50 mM sodium phosphate buffer at pH 8.0 was transferred to a 0.5 mL eppendorf tube. Ellman's reagent (0.2 µL of a 20 mg/mL stock solution in H_2O) was added and the resulting mixture vortexed for 30 seconds. After 30 min of additional shaking, a 2 µL aliquot was analysed by LC–MS (2 µL aliquot diluted by 8 µL of 50 mM sodium phosphate buffer at pH 8.0) and starting protein **C2Am-Dha** (calculated mass, 16188; observed mass, 16188) was detected unaltered.

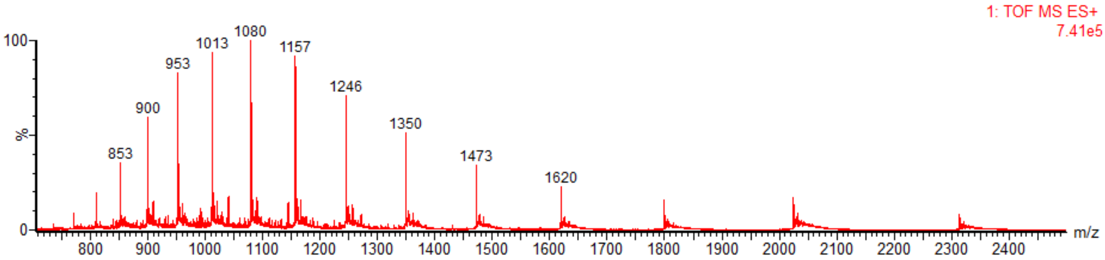

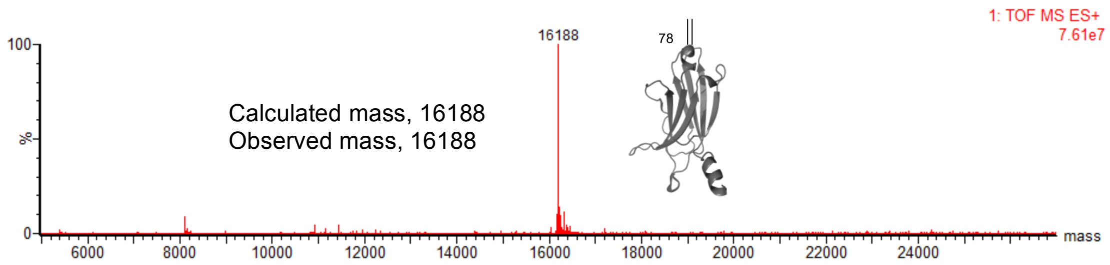

Figure S9. ESI–MS of the reaction of Ellman's reagent with **C2Am-Dha**.

Control: reaction of 2-mercaptoethanol with **C2Am-Dha**

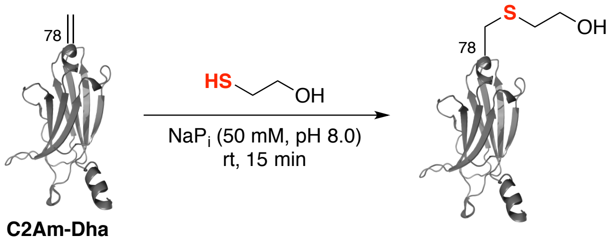

All manipulations were carried out at room temperature. A 10 μL aliquot of **C2Am-Dha** (0.169 mg/mL, 0.104 nmol) in 50 mM sodium phosphate buffer at pH 8.0 was transferred to a 0.5 mL eppendorf tube. 2-Mercaptoethanol (0.2 μL) was added at room temperature and the resulting mixture vortexed for 30 seconds. After 15 min of additional shaking, a 2 μL aliquot was analysed by LC–MS (2 μL aliquot diluted by 8 μL of 50 mM sodium phosphate buffer at pH 8.0) and complete conversion to the expected 2-mercaptoethanol product (calculated mass, 16266; observed mass, 16266) was observed.

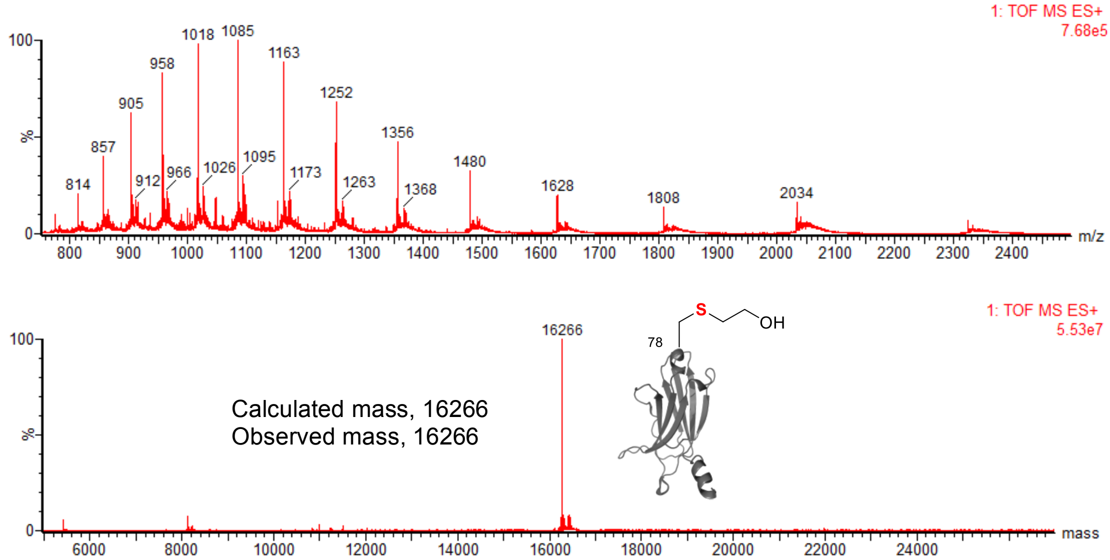

Figure S10. ESI–MS of the reaction of 2-mercaptoethanol with **C2Am-Dha**.

Reaction of alkyne **S15** with **C2Am-Dha**

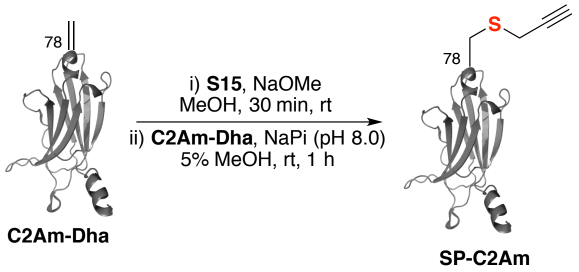

Thiol **S15** solution: NaOMe (123 mg, 2.28 mmol) was added to a stirring solution of **S15** (200 mg, 1.75 mmol) in degassed MeOH (1.2 mL) and stirred for 30 min at rt. Upon completion, as determined by TLC, DOWEX®50WX8 was added to neutralise the reaction. Concentration was determined by ^{1}H-NMR.

Addition to **C2Am-Dha**: All manipulations were carried out at rt. A 100 µL aliquot of **C2Am-Dha** (0.169 mg/mL, 1.04 nmol) in 50 mM sodium phosphate buffer at pH 8.0 was transferred to a 0.5 mL eppendorf tube. Thiol **S15** solution (5 µL of 1.03 M in dry and degassed MeOH) was added at room temperature and the resulting mixture vortexed for 30 seconds. After 1 h of additional shaking, a 2 µL aliquot was analysed by LC–MS (2 µL aliquot diluted by 8 µL of 50 mM sodium phosphate buffer at pH 8.0) and complete conversion to **SP-C2Am** (calculated mass, 16260; observed mass, 16261) was observed. Small molecules were removed from the reaction mixture by loading the sample onto a Zeba™ Spin desalting column (ThermoFisher Scientific) previously equilibrated with 50 mM sodium phosphate buffer at pH 8.0. The sample was eluted by centrifugation (2 min, 1500 × g). The protein sample (0.078 mg/mL by Bradford assay) was flash frozen with liquid nitrogen and stored at –20 ºC.

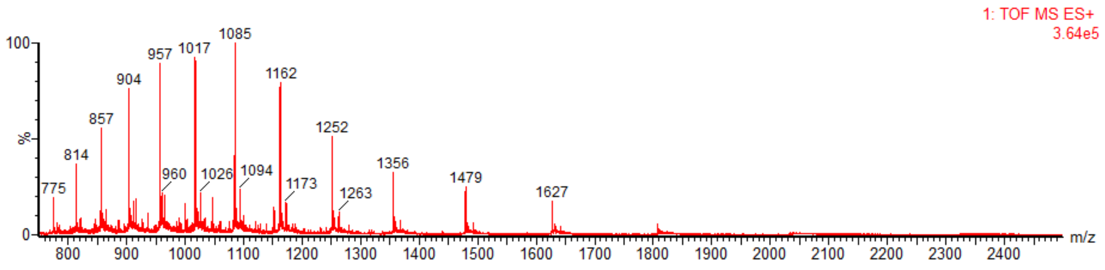

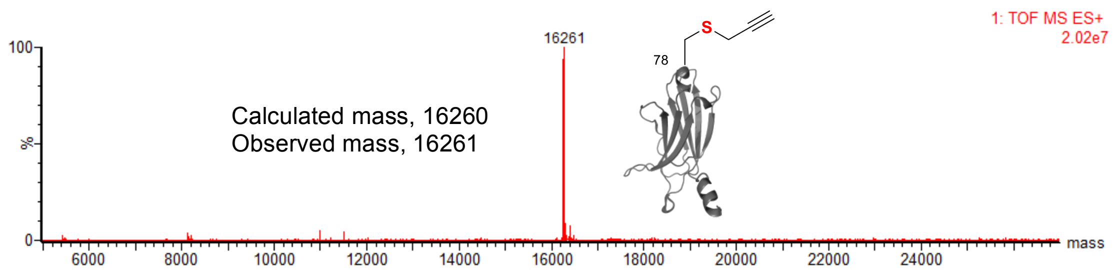

Figure S11. ESI–MS of the reaction of **S15** and **C2Am-Dha** to give **SP-C2Am**.

<u>Reaction of alkyne **40** with **C2Am-Dha**</u>

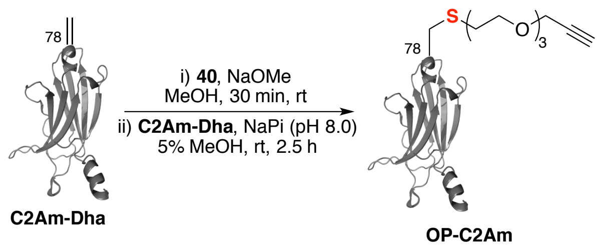

Thiol **40** solution: NaOMe (28.5 mg, 0.528 mmol) was added to a stirring solution of **40** (100 mg, 0.406 mmol) in degassed MeOH (0.1 mL) and stirred for 30 min at rt. Upon completion, as determined by TLC, DOWEX®50WX8 was added to neutralise the reaction. Concentration was determined by ^{1}H-NMR.

All manipulations were carried out at rt. A 100 µL aliquot of **C2Am-Dha** (1.2 mg/mL, 7.41 nmol) in 50 mM sodium phosphate buffer at pH 8.0 was transferred to a 0.5 mL eppendorf tube. Thiol **40** solution (5 µL of 7.4 M in dry and degassed MeOH) was added at room temperature and the resulting mixture vortexed for 30 seconds. After 2.5 h of additional shaking, a 2 µL aliquot was analysed by LC–MS (2 µL aliquot diluted by 8 µL of 50 mM sodium phosphate buffer at pH 8.0) and complete conversion to **OP-C2Am** (calculated mass, 16392; observed mass, 16393) was observed. Small molecules were removed from the reaction mixture by loading the sample onto a Zeba™ Spin desalting column (ThermoFisher Scientific) previously equilibrated with 50 mM sodium phosphate buffer at pH 8.0. The sample was eluted by centrifugation (2 min, 1500 × g). The protein sample (1.19 mg/mL by Bradford assay) was flash frozen with liquid nitrogen and stored at –20 ºC.

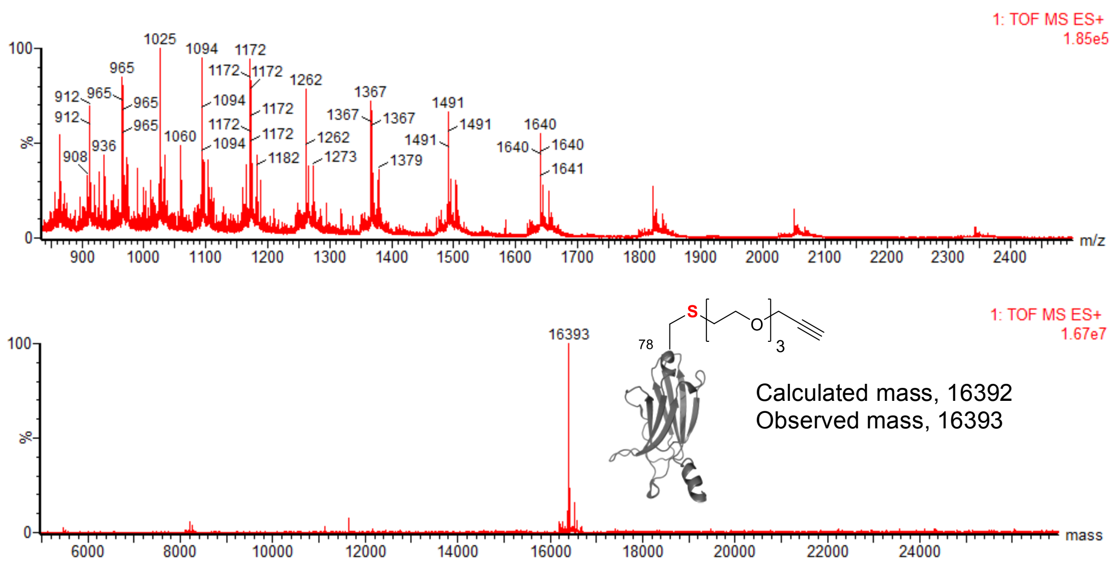

Figure S12. ESI–MS of the crude reaction mixture of alkyne **40** with **C2Am-Dha** after 2.5 h at rt.

Reaction of hydrosilane **8** with **SP-C2Am**

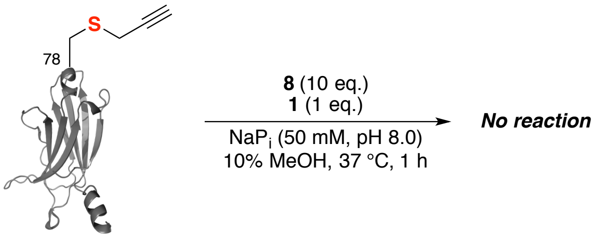

SP-C2Am (4 μM)

A solution of Ru cat. **1** and hippuric acid in PBS buffer at pH 8.0 was prepared by vortexing and gentle warming **1** (0.12 mg, 0.24 μmol) and hippuric acid (0.09 mg, 0.48 μmol) in PBS buffer at pH 8.0 (5 mL). To a 25 μL aliquot of **SP-C2Am** (0.078 mg/mL, 0.12 nmol) in 50 mM sodium phosphate buffer at pH 8.0 was added the catalyst solution (2.5 μL of 48 μM) followed by silane **8** (2.5 μL of 0.48 mM in 50% MeOH in PBS buffer at pH 8.0). The reaction was vortexed for 30 seconds each time after an addition. After 1 h of additional shaking at 37 ºC, a 5 μL aliquot was analysed by LC–MS (5 μL aliquot diluted by 5 μL of 50 mM sodium phosphate buffer at pH 8.0) and starting protein **SP-C2Am** (calculated mass, 16260; observed mass, 16261) was detected unaltered. **Note:** many metal-mediated protein modification protocols are rather sensitive to crowded environments[8,9] and attempts resulted unsuccessful, probably due to the close proximity of the *S*-propargyl cysteine (Spc) handle to the protein surface together with the presence of a sulphur atom, which seems to be detrimental in this transformation (see unsuccessful experiments with *S*- and *Se*-

propargyl handles for further details). These included variations in the amount of silane **8** and/or catalyst **1** added (up to 200 equiv.), reaction temperature (up to 37 ºC), reaction time (>12 h), co-solvent (up to 20% MeOH, *t*-BuOH), additives (hippuric acid, $MgCl_2 \cdot 6H_2O$), and the use of metal scavengers (3-mercaptopropionic acid) and purification methods prior to LC–MS analysis (Zeba^{TM} Spin desalting column from ThermoFisher Scientific).

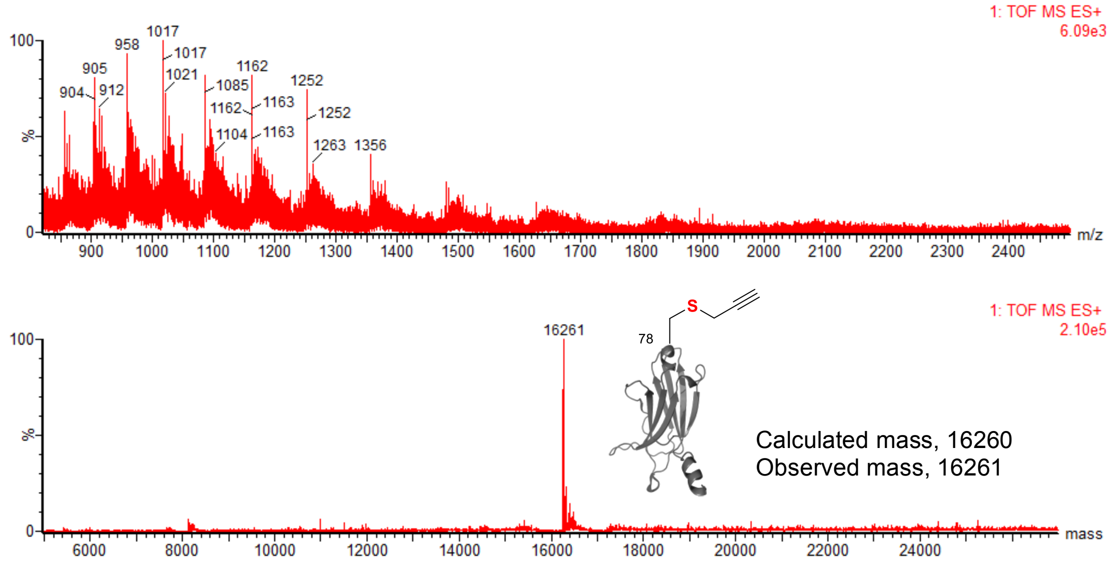

Figure S13. ESI–MS of the reaction of silane **8** with **SP-C2Am** after 1 h at 37 ºC.

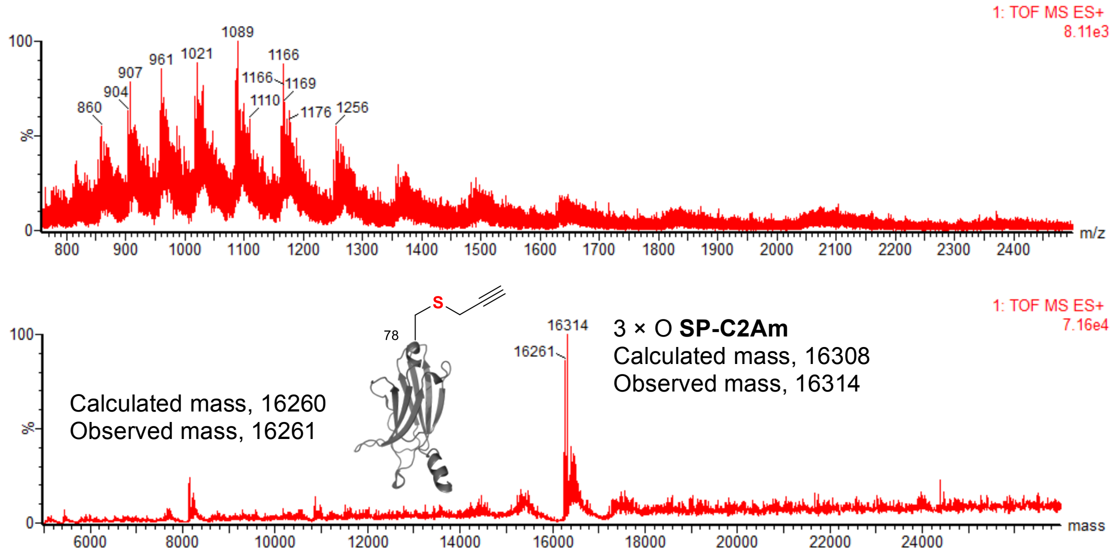

Figure S14. ESI–MS of the reaction of silane **8** with **SP-C2Am** after 3 h at 37 ºC; some oxidation is noticed in this protein upon increasing the loading of catalyst **1** (10 equiv.) and extending the reaction time.

Reaction of silane **8** with **OP-C2Am**

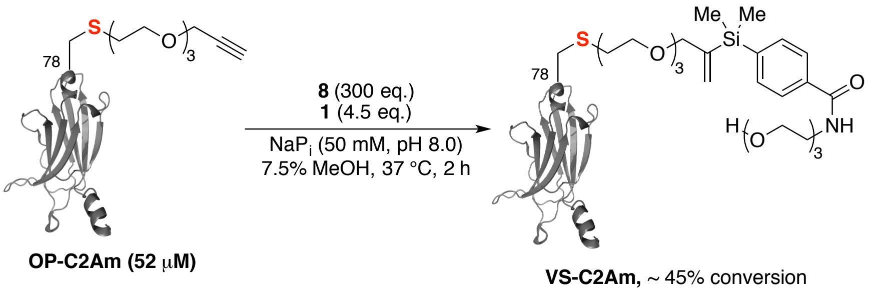

A solution of catalyst **1**/hippuric acid in PBS buffer at pH 8.0 was prepared by vortexing and gentle warming **1** (0.784 mg, 1.55 μmol) and hippuric acid (0.557 mg, 3.11 μmol) in PBS buffer at pH 8.0 (1 mL). To a 7 μL aliquot of **OP-C2Am** (1.19 mg/mL, 0.518 nmol) in 50 mM sodium phosphate buffer at pH 8.0 was added in three portions (every 30 min), the catalyst **1**/hippuric acid solution (0.5 μL) followed by silane **8** (0.5 μL of 104 mM in 50% MeOH in PBS buffer at pH 8.0). The reaction was vortexed for 30 seconds each time after an addition. After 1 h of additional shaking (2 in total) at 37 ºC, a 5 μL aliquot was analysed by LC–MS (5 μL aliquot diluted by 5 μL of 50 mM sodium phosphate buffer at pH 8.0) and conversion to the expected product **VS-C2Am** (calculated mass, 16703; observed mass, 16704) was observed. **Note:** Importantly, this result reinforces the use of an appropriate linker-extended *O*-propargyl handle for protein hydrosilylation with conventional ruthenium catalysts.[10] Attempts to improve conversion by tuning reaction conditions resulted unsuccessful. These included variations in the amount of silane **8** and/or catalyst **1** added (up to 200 equiv.), reaction temperature (up to 37 ºC), reaction time (>12 h), co-solvent (up to 20% MeOH, *t*-BuOH), additives (hippuric acid, $MgCl_2·6H_2O$), and the use of metal scavengers (3-mercaptopropionic acid) and purification methods prior to LC–MS analysis (Zeba™ Spin desalting column from ThermoFisher Scientific).

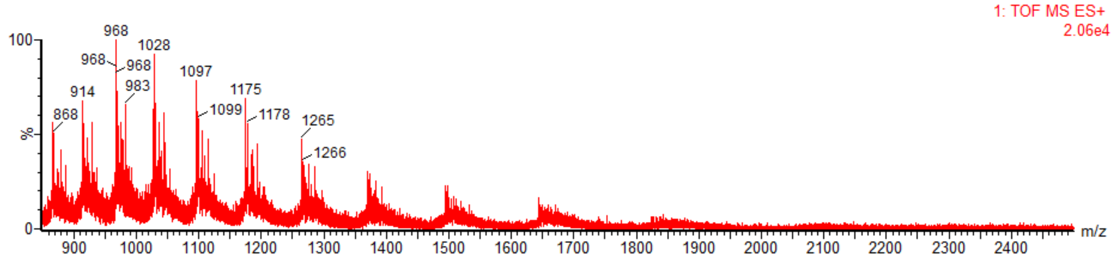

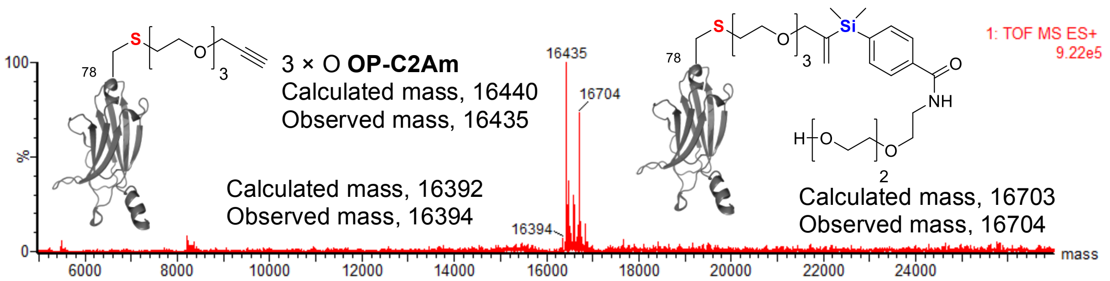

Figure S15. ESI–MS of the reaction of silane **8** with **OP-C2Am** after 1 h at 37 ºC; some oxidation is noticed in this protein under the conditions tested.

3.5. Modification of **VS-Lyz**

Synthesis of **VS-Lyz**

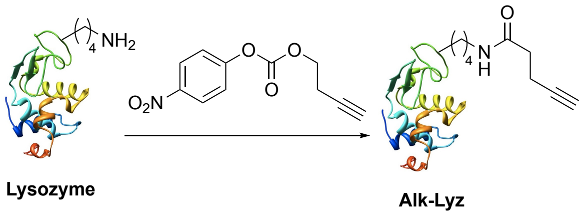

But-3-yn-1-yl (4-nitrophenyl) carbonate (11 mg, 0.05 mmol) was dissolved in DMSO (250 µL) and transferred directly to PBS buffer at pH 7.4 (2.25 mL) containing lysozyme (10 mg, Sigma). The mixture was agitated for 1 h and the protein was purified on a PD-10 desalting column (GE Healthcare Life Sciences). The column was pre-equilibrated with PBS buffer at pH 7.4 (25 mL), the protein mixture added to the column and eluted with PBS buffer at pH 7.4 (3.5 mL). Protein concentrations were assayed by a NanoDrop2000 (Thermo Scientific).

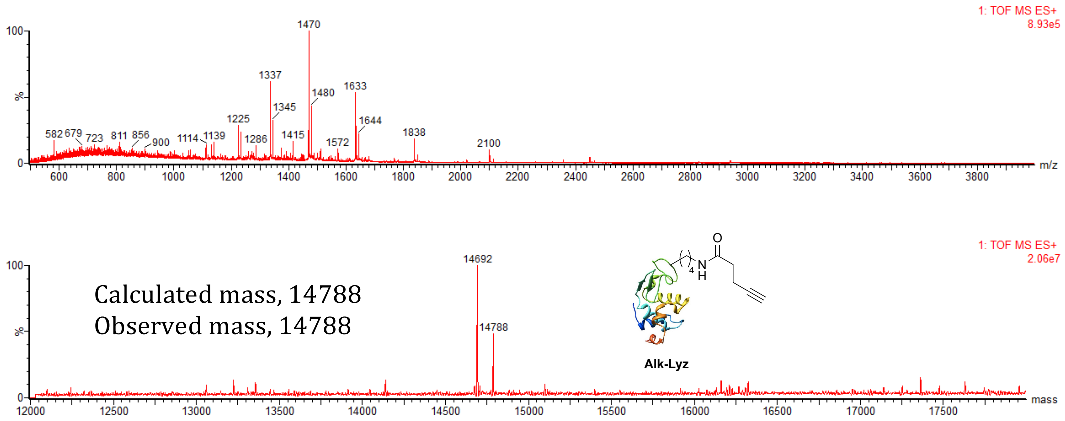

Figure S16. ESI–MS of reaction of lysozyme with but-3-yn-1-yl (4-nitrophenyl) carbonate.

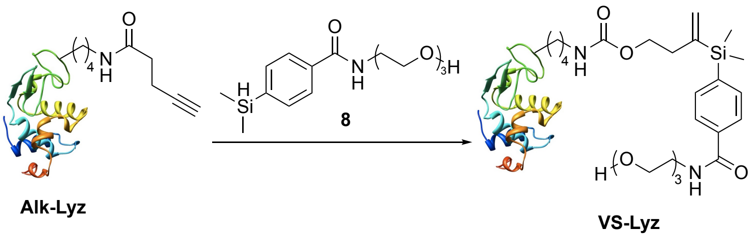

To a 50 µL aliquot of **Alk-Lyz** (3.95 mg/mL, 13.4 nmol) was added **8** (1.34 µL of 50 mM solution), $[Cp^*Ru(MeCN)_3]PF_6$ **1** (1.34 µL of a 5 mM solution, 0.5 equivalents) and hippuric acid (2.68 µL of a 5 mM solution). The mixture was vortexed for 30 seconds followed by incubation at 37 °C for 14 h after which time the small molecules

were removed from the reaction mixture by loading the sample onto a Zeba^{TM} Spin desalting column (ThermoFisher Scientific) previously equilibrated with PBS buffer at pH 7.4. The sample was eluted by centrifugation (2 min, 1500 × g). A 10 µL aliquot was analysed by LC–MS and **VS-Lyz** (calculated mass, 15100; observed mass, 15100) was observed.

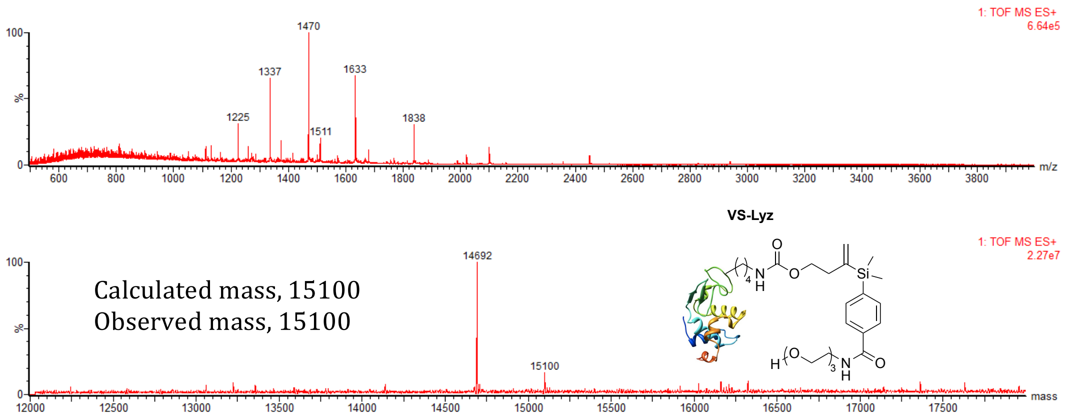

Figure S17. ESI–MS of **VS-Lyz**.

Thiol-ene Reaction of **VS-Lyz**

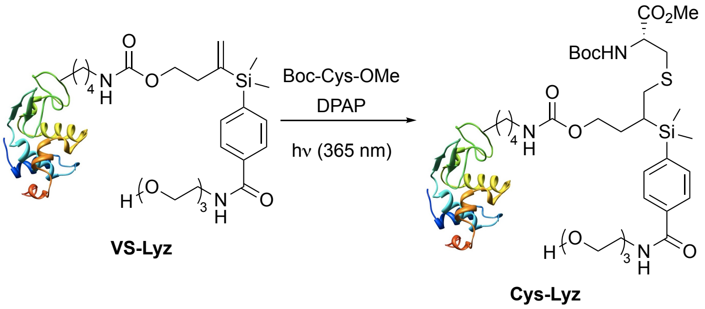

All manipulations were carried out at rt. A 50 µL aliquot of **VS-Lyz** (3.46 mg/mL, 11.5 nmol) in PBS buffer at pH 7.4 was transferred to a 1.5 mL eppendorf tube. *N*-(*tert*-butoxycarbonyl)-L-cysteine methyl ester (2.3 µL of 500 mM in dry MeOH) and 2,2-dimethoxy-2-phenylacetophenone (DMPA, 2.3 µL of 500 mM in dry MeOH) was added at room temperature and the resulting mixture vortexed for 30 seconds. After 3 h of irradiation at 365 nm the small molecules were removed from the reaction mixture by loading the sample onto a Zeba^{TM} Spin desalting column (ThermoFisher Scientific) previously equilibrated with PBS buffer at pH 7.4. The sample was eluted by centrifugation (2 min, 1500 × g). A 10 µL aliquot was analysed by LC–MS and **Cys-Lyz** (calculated mass, 15335; observed mass, 15333, 15335) was observed.

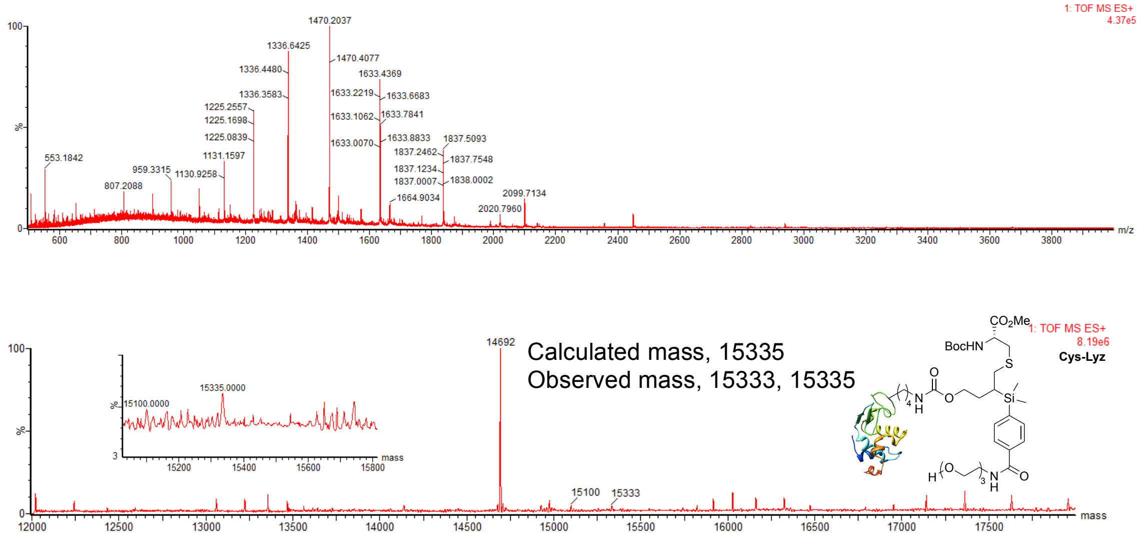

Figure S18. ESI–MS of the reaction of **VS-Lyz** with Boc-Cys-OMe and DMPA under irradiation at 365 nm for 3 h to generate **Cys-Lyz**.

Protodesilylation of **VS-Lys**

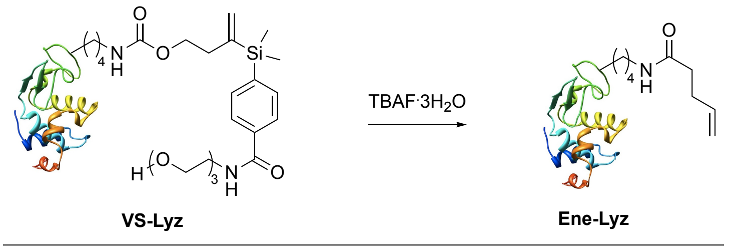

All manipulations were carried out at rt. A 50 µL aliquot of **VS-Lyz** (3.46 mg/mL, 11.5 nmol) in PBS buffer at pH 7.4 was transferred to a 1.5 mL eppendorf tube. TBAF·3H_2O (5.8 µL of 100 mM PBS buffer at pH 7.4) was added at room temperature and the resulting mixture vortexed for 30 seconds. The reaction was incubated at room temperature for 6 h and the small molecules were removed from the reaction mixture by loading the sample onto a Zeba^{TM} Spin desalting column (ThermoFisher Scientific) previously equilibrated with PBS buffer at pH 7.4. The sample was eluted by centrifugation (2 min, 1500 × g). A 10 µL aliquot was analysed by LC–MS and **Ene-Lyz** (calculated mass, 14790; observed mass, 14790) was observed.

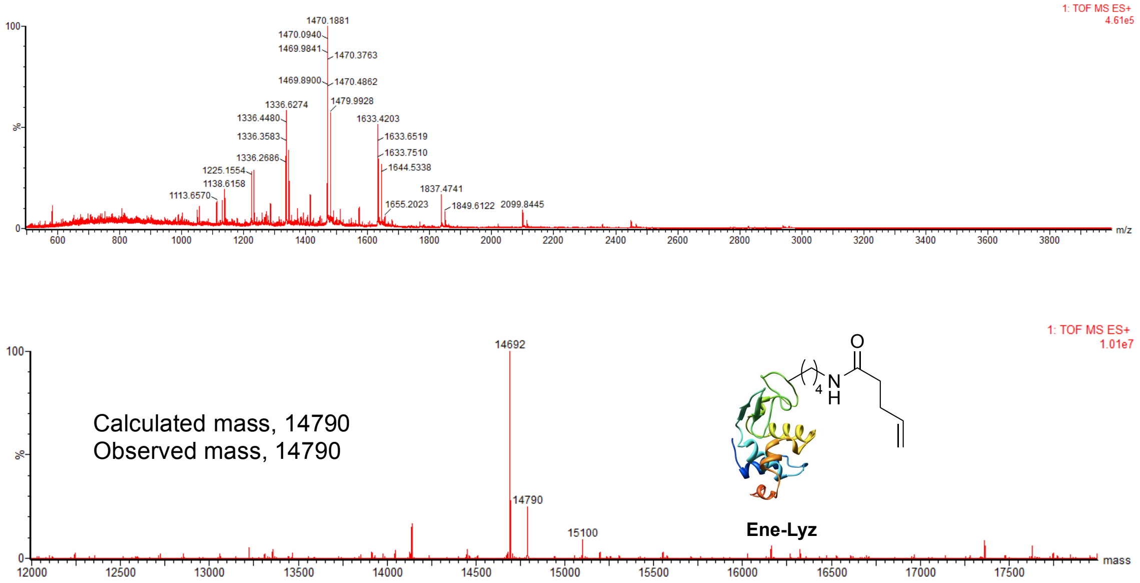

Figure S19. ESI–MS of **Ene-Lyz** formed by reaction of **VS-Lyz** with TBAF·3H_2O for 6 h at rt.

4. Kinetic analysis of aqueous hydrosilylation

Due to the limited insight into the mechanism of hydrosilylation in water, it is assumed the reaction follows a mixed-order rate law. This suggests conventional methods to determine second-order rate constants cannot be applied. In addition, the presence of a side-reaction complicates any kinetic analysis. It was decided to use a kinetic modelling program, DynoChem,[11] to aid the interpretation of the hydrosilylation kinetics. DynoChem uses automated fitting algorithms to fit experimental data to proposed reaction schemes, producing experimental rate constants. The following simplified mechanistic steps were proposed:

Hydrosilane + **Catalyst** $\xrightarrow{k_1}$ **Intermediate**

Intermediate + **Alkyne** $\xrightarrow{k_2}$ **Product** + **Catalyst**

Intermediate + **Water** $\xrightarrow{k_3}$ **Silanol** + $\mathbf{H_2}$

The reaction between hydrosilane **4**, alkyne **2** and Ru catalyst **1** at various concentrations in D_2O were monitored *in situ* by ^{1}H-NMR in reference to an internal standard (4,4-dimethyl-4-silapentane-1-sulfonic acid), with spectra being taken at regular intervals. Curves were fitted against observed data to give approximate rate constants for the reaction steps: k_1 >10 M^{-1} s^{-1}, $k_2 \approx 1$ M^{-1} s^{-1} and $k_3 \approx 10^{-4}$ M^{-1} s^{-1}.

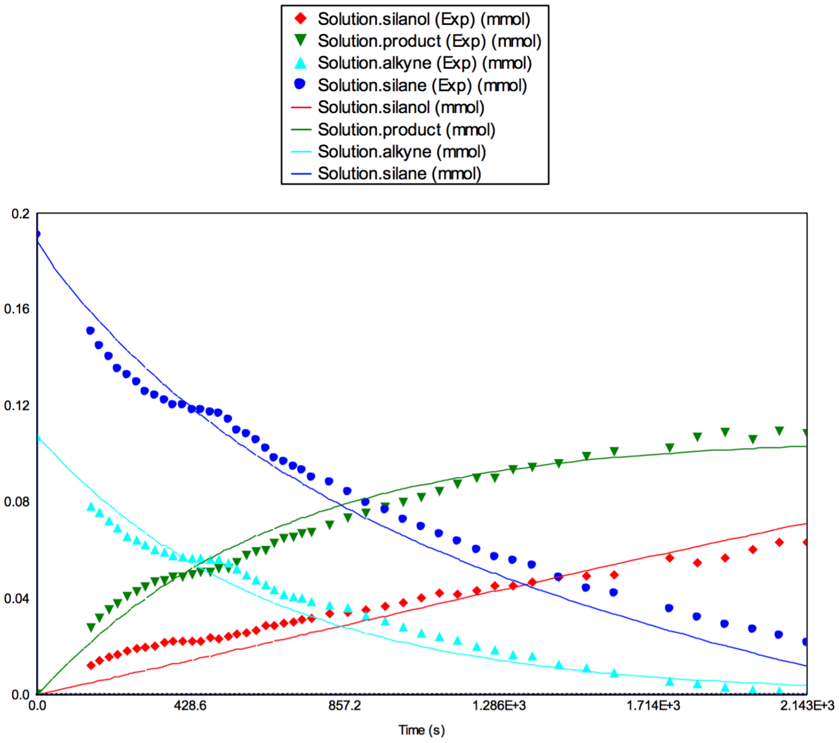

Figure S20. Fitting of experimental data to obtain an approximation of rate constants

5. Stability of hydrosilane

Hydrosilane **4** (10 mg, 29 mmol) was dissolved in 0.7 mL of deuterated buffer (Table S1) and ^{1}H-NMR spectrums were recorded at periodic intervals. Hydrolysis of **4** to the corresponding silanol was monitored by evaluating the integration values of distinct ^{1}H signal corresponding to (SiH(C$\underline{H}_3$)$_2$) of the two compounds: $\boldsymbol{\delta}_{\mathbf{H}}$ = 0.33, 6H, d, J = 3.7 Hz for **4**, $\boldsymbol{\delta}_{\mathbf{H}}$ = 0.45, 6H, s for silanol.

pD	Buffering Agent	k_{obs} (min^{-1})	r^2
2	KCl / DCl	–4.63E–04	0.989
3	Potassium Hydrogen Phthalate	–2.18E–04	0.936
4		–1.61E–04	1.000
5		–1.03E–04	1.000
6	Potassium dihydrogen phosphate KH_2PO_4	–3.75E–05	0.998
6.5		–6.93E–05	0.997
7		–5.94E–05	1.000
7.5		–9.38E–05	0.985
8		–3.09E–04	1.000
9	Sodium tetraborate $Na_2B_4O_7 \cdot 10H_2O$	–1.20E–03	0.999
10		–1.08E–02	0.996

Table S1–Buffering agent used to prepare range of pD buffers, and their respective rate constant for the hydrolysis of **4** (k_{obs}) and r-squared values (r^2).

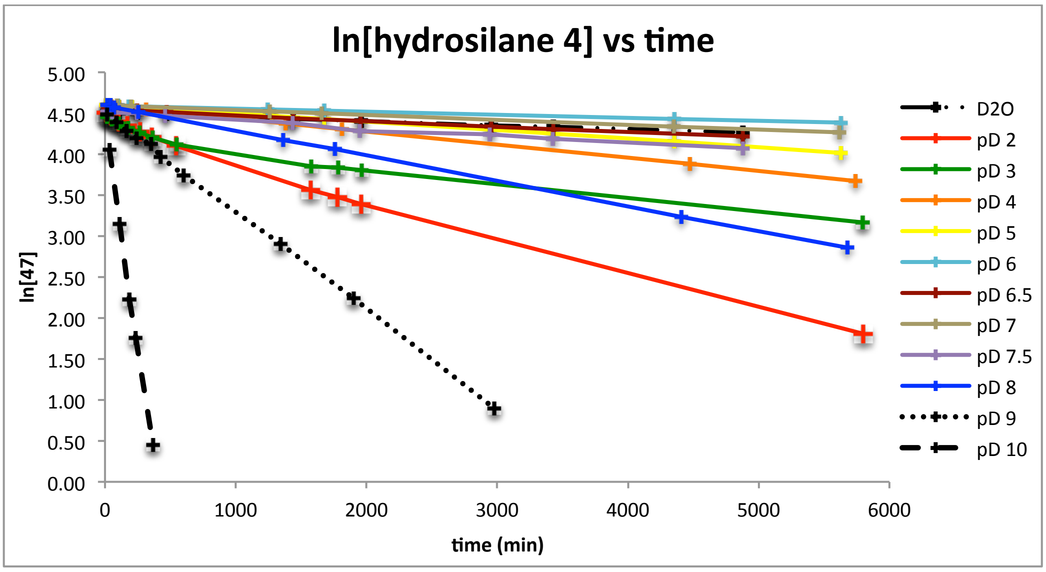

Figure S21. Plot of ln[hydrosilane **4**] against time to determine k_{obs} (min^{-1}) for different pDs

6. Organic co-solvent effect on aqueous alkyne hydrosilylation

[RuCp*(MeCN)$_3$]PF$_6$ **1** (5 mol%) was added to a solution of hydrosilane **4** (77.9 mg, 225 mmol) and alkyne **2** (16.2 mg, 75 mmol) in deuterated solvent (3 mL) at RT and ^{1}H-NMR spectra were recorded at periodic intervals in the presence of DSS as an internal standard. Distinct δ_H signals – Si(C$\underline{H}_3$)$_2$ 0.36 (d, J = 3.8 Hz, **4**), 0.45 (s, **S16**); C$\underline{H}_2$C≡CH 4.24 (d, J = 2.4 Hz, **2**); C=C$\underline{H}_2$ 5.66–5.67 and 5.95–5.96 (m, **S16**).

Figure **S21** highlights the difference in the initial hydrosilylation rates when different organic co-solvents were used in aqueous alkyne hydrosilylation, where the significant reduction in rate was observed for 10% DMSO and 10% MeCN. As DMSO– and MeCN–Ru complexes have been reported previously,[12–15] these solvents' high affinity for the Ru catalyst may potentially prevent the formation of active catalytic complex, hindering hydrosilylation.

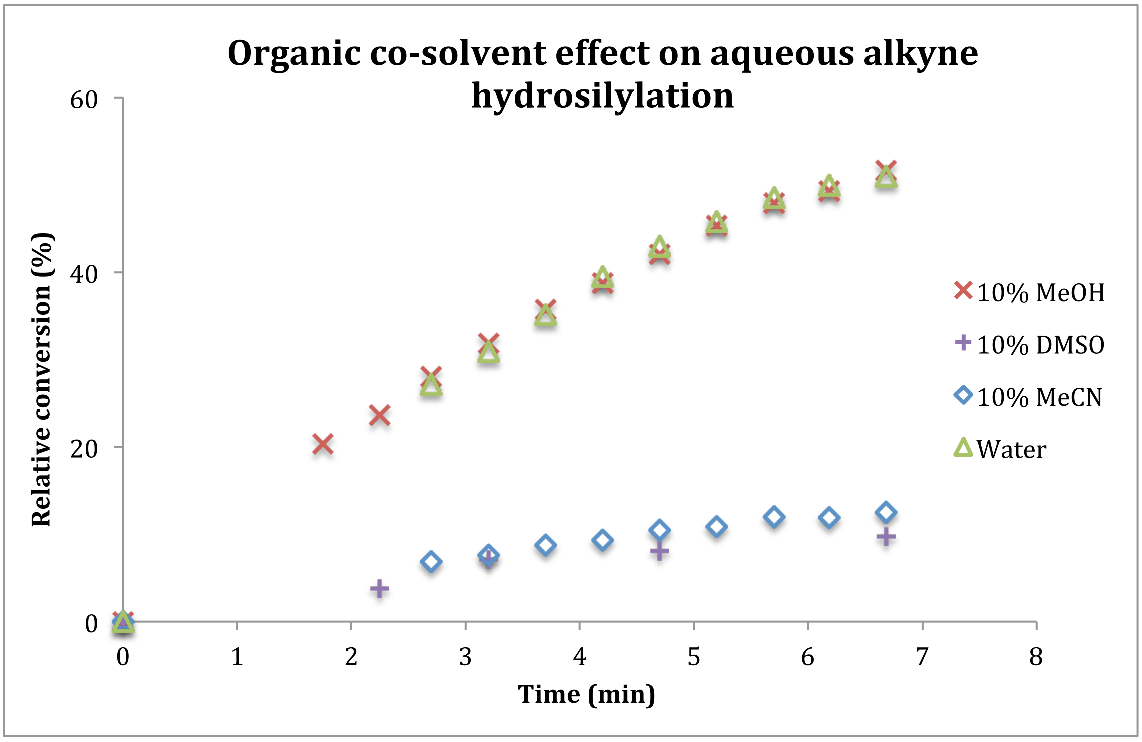

Figure S22. Plot of relative conversion (%) to vinylsilane **S16** against time to determine the effects of different organic co-solvents (MeOH, MeCN and DMSO) in aqueous alkyne hydrosilylation.

7. Compatibility study with carbonyl condensation

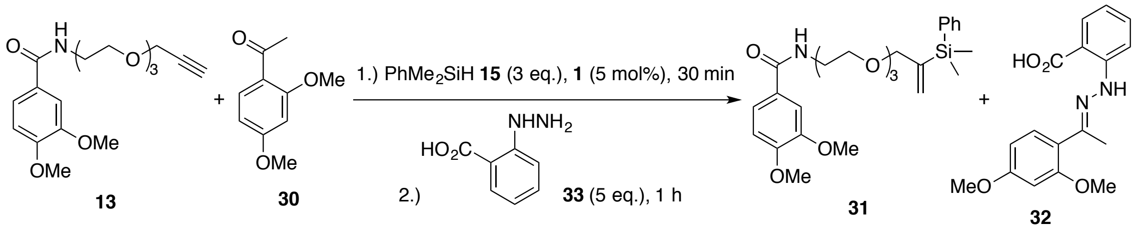

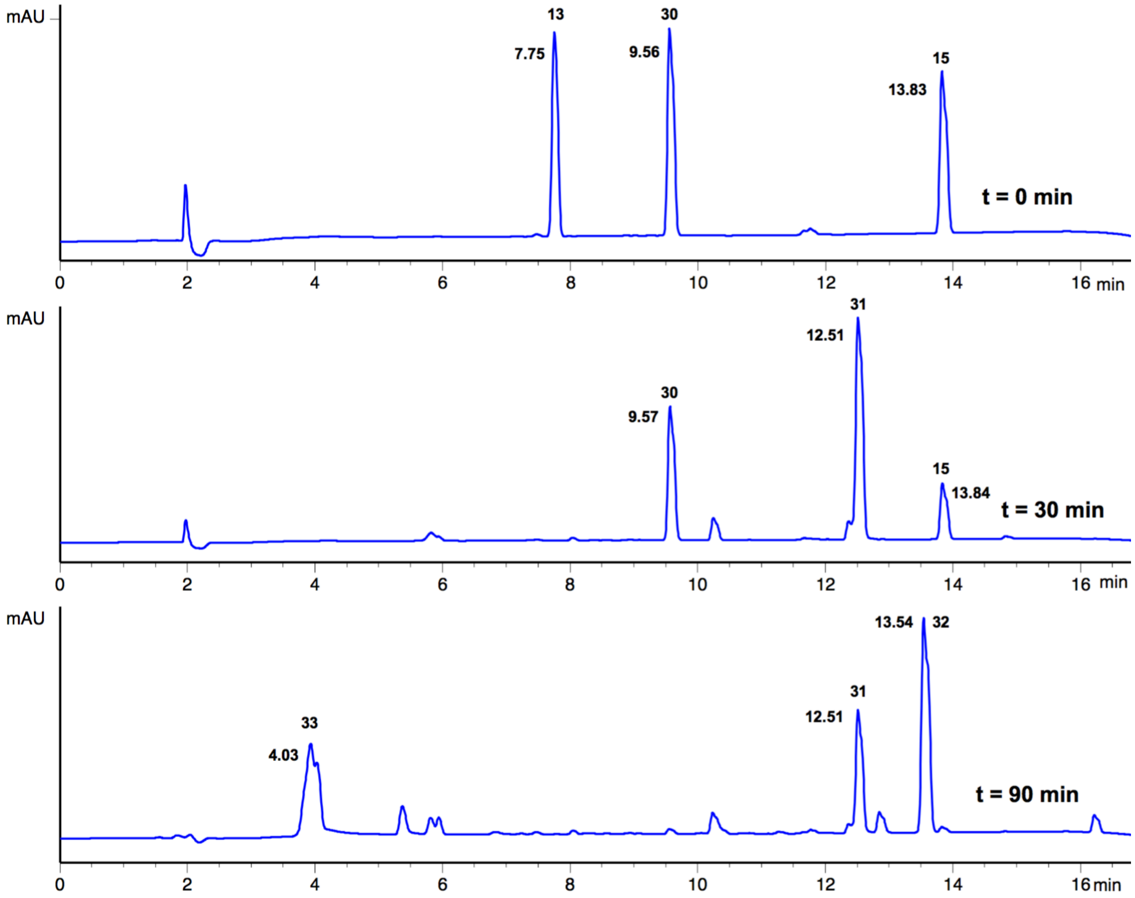

Figure S23. Step-wise, one-pot reaction between hydrosilylation and condensation reaction. **13** (25 μmol), **15** (75 μmol), **30** (50 μmol), **1** (5 mol%) and hippuric acid (2.5 μmol) were dissolved in 1 mL of 50% MeOH in water at 37 ºC for 30 min, **33** (250 μmol) was added and the reaction was monitored by HPLC over 1.5 h.

3,4-Dimethoxy-*N*-(2-methyl-3-methylene-2-phenyl-5,8,11-trioxa-2-silatridecan-13-yl) benzamide (31)

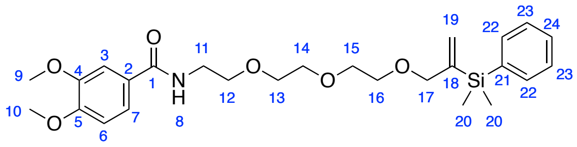

Alkyne **13** (35.1 mg, 100 µmol), silane **15** (20.4 mg, 150 µmol) and [Cp*Ru(MeCN)$_3$]PF$_6$ **1** (2.52 mg, 5.00 mmol) were reacted according to GSP 3 and purified by FCC (1:49 MeOH:CH_2Cl_2) to give **31** as a pale yellow oil (45.8 mg, 93.9 µmol, 94%).

R$_f$ 0.15 (1:49 MeOH:CH_2Cl_2); ***ν$_{max}$*** (neat)/cm^{-1} 2868, 1635, 1505, 1267, 1230, 1130, 1109; ***δ*$_H$** (500 MHz, $CDCl_3$) 7.50–7.51 (2H, m, 2 × H22), 7.44 (1H, d, *J* = 2.0 Hz, H3), 7.33–7.34 (3H, m, 2 × H23, H24), 7.29 (1H, dd, *J* = 8.4, 2.0 Hz, H7), 6.83 (1H, d, *J* = 8.4 Hz, H6), 6.68 (1H, s, H8), 5.86–5.88 (1H, m, H19–a), 5.47 (1H, m, H19–b), 4.04 (2H, t, *J* = 1.4 Hz, H17), 3.93 (3H, s, H9), 3.90 (3H, s, H10), 3.45–3.67 (12H, m, H11–H16), 0.38 (6H, s, 2 × H20); ***δ*$_C$** (126 MHz, $CDCl_3$) 167.2 (C1), 151.8 (C5), 149.1 (C4), 147.2 (C18), 137.9 (C21), 134.1 (2 × C22), 129.2 (C24), 127.9 (2 × C23), 127.4 (C2), 127.0 (C19), 119.6 (C7), 110.9 (C3), 110.3 (C6), 75.3 (C17), 70.7 (1 C from C12–C16), 70.6 (1 C from C12–C16), 70.4 (1 C from C12–C16), 70.1 (1 C from C12–C16), 69.6 (1 C from C12–C16), 56.2 (C9 or C10), 56.1 (C9 or C10), 39.9 (C11), –2.9 (2 × C20); **HRMS** (ESI) *m/z* found $[M+H]^+$ 488.2455, $C_{26}H_{38}NO_6{}^{28}Si^+$ required 488.2468.

(*E*)-2-(2-(1-(2,4-Dimethoxyphenyl)ethylidene)hydrazinyl)benzoic acid (32)

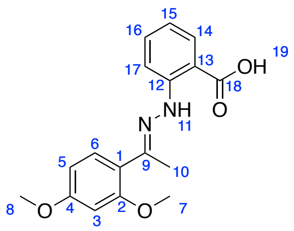

A solution of 2',4'-dimethoxyacetophenone **30** (100 mg, 0.555 mmol) in MeOH (15 mL) was added dropwise to a stirring solution of 2-hydrazinobenzoic acid hydrochloride **33** (105 mg, 0.555 mmol) in MeOH (15 mL) and the resulting solution was stirred for 16 h at rt. Upon completion, the reaction mixture was filtered,

concentrated *in vacuo* and recrystallised from hot MeOH to obtain hydrazone **32** as a yellow solid (102 mg, 60%). ***R_f*** 0.32 (1:19 MeOH:CH_2Cl_2); **m.p.** 212.8–213.0 °C (MeOH); ***ν_{max}*** (neat)/cm^{-1} 3323, 2834, 1665, 1614, 1578, 1503, 1454, 1422, 1207, 1161; ***δ_H*** (500 MHz, DMSO-*d_6*) 13.08 (1H, br s, H19), 11.04 (1H, s, H11), 7.85 (1H, dd, *J* = 8.0, 1.5 Hz, H14), 7.58 (1H, dd, *J* = 8.5, 1.0 Hz, H17), 7.44–7.48 (1H, m, H16), 7.33 (1H, d, *J* = 8.4 Hz, H6), 6.75–6.78 (1H, m, H15), 6.62 (1H, d, *J* = 2.4 Hz, H3), 6.56 (1H, dd, *J* = 8.5, 2.4 Hz, H5), 3.81 (3H, s, H7), 3.79 (3H, s, H8), 2.18 (3H, s, H10); ***δ_C*** (126 MHz, DMSO-*d_6*) 170.1 (C18), 160.9 (C4), 158.2 (C2), 147.6 (C12), 145.5 (C9), 134.6 (C16), 131.2 (C14), 130.1 (C6), 122.0 (C1), 117.2 (C15), 112.9 (C17), 109.7 (C13), 105.1 (C5), 98.7 (C3), 55.6 (C7), 55.3 (C8), 16.8 (C10); **HRMS** (ESI) *m/z* found $[M+H]^+$ 315.1357, $C_{17}H_{19}N_2O_4^+$ required 315.1345; **Elemental analysis** calculated for $C_{17}H_{18}N_2O_4$ (%) C 64.96, H 5.77, N 8.91; found C 64.93, H 5.80, N 8.91.

8. Stability of vinylsilane 26

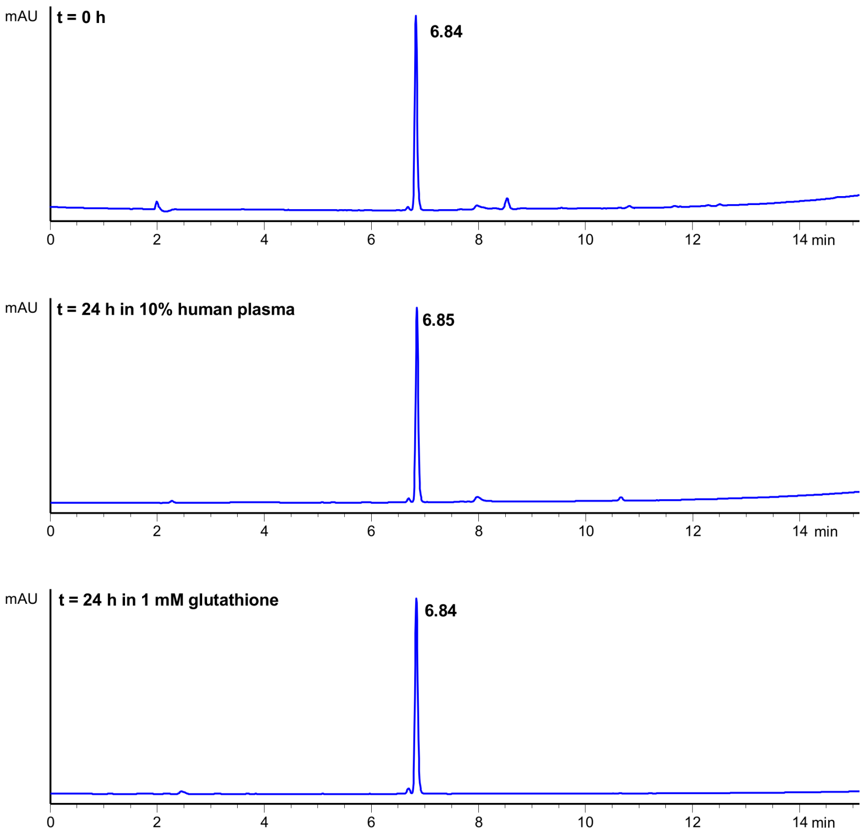

Figure S24. Vinylsilane **26** is stable in physiologically relevant conditions. **26** (1 mM) was incubated with 10% human plasma and glutathione (1 mM) respectively in PBS buffer (pH 7.4) at 37 ºC for 24 h and analysed *via* HPLC.

9. Thiol-ene and protodesilylation of model vinylsilane 31

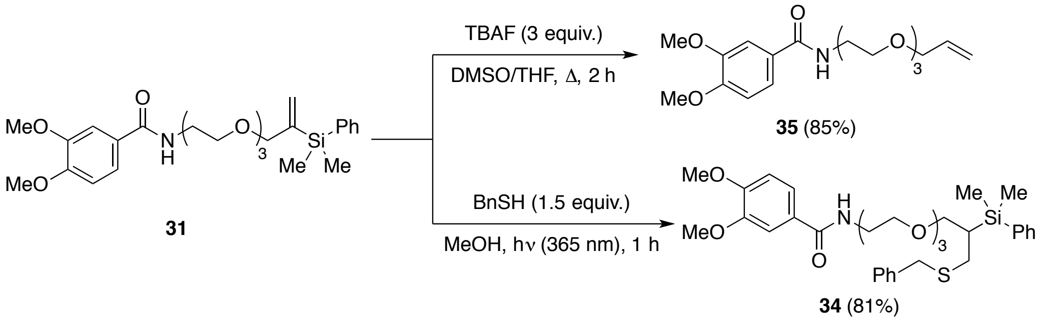

Scheme S1. TBAF protodesilylation and thiol-ene reaction to further chemically modify vinylsilane **31**.

N-(2-(2-(2-(Allyloxy)ethoxy)ethoxy)ethyl)-3,4-dimethoxybenzamide (35)

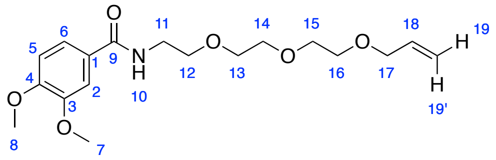

1 M TBAF in THF (462 μL, 0.461 mmol) was added to a solution of **31** (75.0 mg, 0.154 mmol) in DMSO (225 μL) under Ar atmosphere and stirred at 80 ºC for 2 h. The resulting mixture was diluted with EtOAc (5 mL) and washed with water (5 × 10 mL). The aqueous layer was back extracted with EtOAc (2 × 10 mL) and the combined organic layers were dried (Na_2SO_4), concentrated *in vacuo* and purified by FCC (3:97 MeOH:CH_2Cl_2) to give alkene **35** as a clear oil (46.0 mg, 85%). $\boldsymbol{R_f}$ 0.34 (1:19 MeOH:CH_2Cl_2); $\boldsymbol{\nu_{max}}$ (neat)/cm^{-1} 3318, 2864, 1636, 1603, 1506, 1268, 1230, 1130, 1024; $\boldsymbol{\delta_H}$ (500 MHz, $CDCl_3$) 7.44 (1H, d, *J* = 2.0 Hz, H2), 7.31 (1H, dd, *J* = 8.4, 2.1 Hz, H6), 6.85 (1H, d, *J* = 8.4 Hz, H5), 6.73 (1H, s, H10), 5.87 (1H, ddt, *J* = 17.2, 11.4, 5.2 Hz, H18), 5.24 (1H, ddt, *J* = 17.2, 1.6, 1.6, H19’), 5.15 (1H, ddt, *J* = 10.4, 1.2, 1.5, H19), 3.97 (2H, dt, *J* = 5.7, 1.4 Hz, H17), 3.93 (3H, s, H7), 3.91 (3H, S, H8), 3.57–3.68 (12H, m, H11–H16); $\boldsymbol{\delta_C}$ (126 MHz, $CDCl_3$) 167.2 (C9), 151.8 (C4), 149.0 (C3), 134.7 (C18), 127.4 (C1), 119.6 (C6), 117.4 (C19), 110.9 (C2), 110.3 (C5), 72.4 (C17), 70.7 (2 C from C12–C16), 70.4 (1 C from C12–C16), 70.1 (1 C from C12–C16), 69.5 (1 C from C12–C16), 56.2 (C7 or C8), 56.1 (C7 or C8), 39.9 (C11); **HRMS** (ESI) *m/z* found $[M+H]^+$ 354.1917, $C_{18}H_{28}NO_6^+$ required 354.1917. **Note:** this transformation also occurred with a similar efficiency at 37 ºC for 16 h.

N-(3-((Benzylthio)methyl)-2-methyl-2-phenyl-5,8,11-trioxa-2-silatridecan-13-yl)-3,4-dimethoxybenzamide (34)

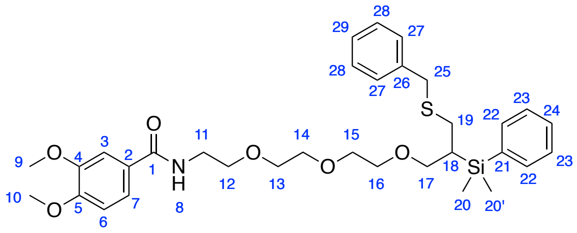

To a stirring solution of **31** (100 mg, 0.205 mmol) in MeOH (2 mL) was added benzyl mercaptan (38.2 mg, 0.308 mmol) and 2,2-dimethoxy-2-phenylacetophenone (21.0 mg, 0.082 mmol) and irradiated at 365 nm for 1 h at rt. Upon completion, the solvent was removed *in vacuo* and purified by FCC (1:49 MeOH:CH_2Cl_2) to give thioether **34** as a clear oil (125 mg, 81%). $\boldsymbol{R_f}$ 0.46 (1:19 MeOH:CH_2Cl_2); $\boldsymbol{\nu}_{max}$ (neat)/cm^{-1} 3329, 2871, 1637, 1506, 1269, 1231, 1112, 1026; $\boldsymbol{\delta}_{\mathbf{H}}$ (700 MHz, $CDCl_3$) 7.46–7.48 (2H, m, 2 × H22), 7.44 (1H, d, J = 2.0 Hz, H3), 7.31–7.36 (3H, m, 2 × H23, H24), 7.26–7.28 (1H, m, H7), 7.22–7.25 (2H, m, 2 × H28), 7.18–7.20 (1H, m, H29), 7.15–7.16 (2H, m, 2 × H27), 6.83 (1H, d, J = 8.3 Hz, H6), 6.63 (1H, s, H8), 3.92 (3H, s, H9), 3.90 (3H, s, H10), 3.69 (1H, dd, J = 9.1, 4.1 Hz, H19–a), 3.44–3.65 (15H, m, H11–H16, H19–b, H25), 2.59 (1H, dd, J = 13.0, 4.0 Hz, H17–a), 2.53 (1H, dd, J = 13.0, 10.6 Hz, H17–b), 1.28–1.31 (1H, m, H18), 0.29 (3H, s, H20 or H20’), 0.28 (3H, s, H20 or H20’); $\boldsymbol{\delta}_{\mathbf{C}}$ (176 MHz, $CDCl_3$) 167.2 (C1), 151.8 (C5), 149.1 (C4), 138.7 (C26), 138.0 (C21), 134.1 (2 × C22), 129.1 (C24), 128.9 (2 × C27), 128.5 (2 × C28), 127.8 (2 × C23), 127.4 (C2), 126.9 (C29), 119.5 (C7), 110.9 (C3), 110.3 (C6), 70.7 (3 C from C12–C16 or C19), 70.5 (1 C from C12–C16 or C19), 70.3 (1 C from C12–C16 or C19), 70.1 (1 C from C12–C16 or C19), 56.2 (C9), 56.1 (C10), 39.9 (C11), 36.4 (C25), 30.5 (C17), 27.8 (C18), –3.3 (C20 or C20’), –3.7 (C20 or C20’); **HRMS** (ESI) *m/z* found $[M+Na]^+$ 634.2620, $C_{33}H_{45}NO_6{}^{32}S^{28}SiNa^+$ required 634.2629.

10. References

(1) Dong, Y.; Lam, J. W. Y.; Qin, A.; Li, Z.; Liu, J.; Sun, J.; Dong, Y.; Tang, B. Z. *Chem. Phys. Lett.* **2007**, *446*, 124.

(2) Wang, M.; Gan, D.; Wooley, K. L. *Macromolecules* **2001**, *34*, 3215.

(3) Banert, K.; Toth, C. *Angew. Chem., Int. Ed.* **1995**, *34*, 1627.

(4) Ning, X.; Lee, S.; Wang, Z.; Kim, D.; Stubblefield, B.; Gilbert, E.; Murthy, N. *Nat. Mater.* **2011**, *10*, 602.

(5) Castro, J.; Moyano, A.; Pericàs, M. A.; Riera, A. *Synthesis* **1997**, *1997*, 518.

(6) Li, J.; Yu, J.; Zhao, J.; Wang, J.; Zheng, S.; Lin, S.; Chen, L.; Yang, M.; Jia, S.; Zhang, X.; Chen, P. R. *Nat. Chem.* **2014**, *6*, 352.

(7) Alam, I. S.; Neves, A. A.; Witney, T. H.; Boren, J.; Brindle, K. M. *Bioconjugate Chem.* **2010**, *21*, 884.

(8) Boutureira, O.; Bernardes, G. J. L. *Chem. Rev.* **2015**, *115*, 2174.

(9) Lin, Y. A.; Boutureira, O.; Lercher, L.; Bhushan, B.; Paton, R. S.; Davis, B. G. *J. Am. Chem. Soc.* **2013**, *135*, 12156.

(10) Lin, Y. A.; Chalker, J. M.; Davis, B. G. *J. Am. Chem. Soc.* **2010**, *132*, 16805.

(11) DynoChem, Performance Fluid Dynamics Ltd www.scale-up.com.

(12) Díaz-Torres, R.; Alvarez, S. *Dalton Trans.* **2011**, *40*, 10742.

(13) Xie, Y.-F.; Zhu, H.; Shi, H.-T.; Jia, A.-Q.; Zhang, Q.-F. *Inorg. Chim. Acta* **2015**, *428*, 147.

(14) Wang, Y.; Duan, L.; Wang, L.; Chen, H.; Sun, J.; Sun, L.; Ahlquist, M. S. G. *ACS Catal.* **2015**, *5*, 3966.

(15) Yamamura, T.; Nakane, S.; Nomura, Y.; Tanaka, S.; Kitamura, M. *Tetrahedron* **2016**, *72*, 3781.

11. NMR Spectra

N-(4-Bromobenzyl)-*N*-ethylethanamine (**S1**), ^{1}H NMR ($CDCl_3$, 400 MHz)

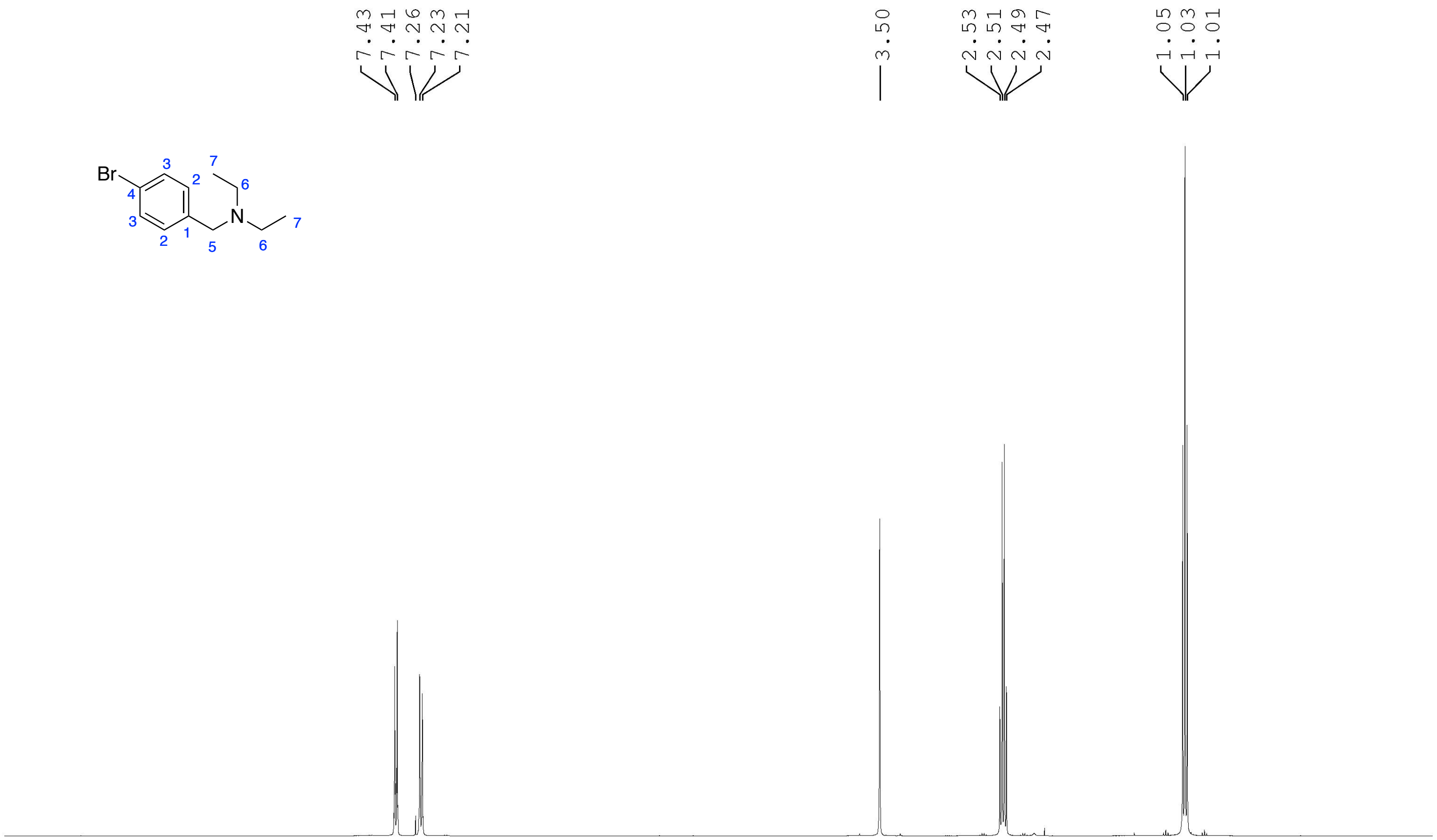

N-(4-Bromobenzyl)-*N*-ethylethanamine (**S1**), ^{13}C NMR ($CDCl_3$, 101 MHz)

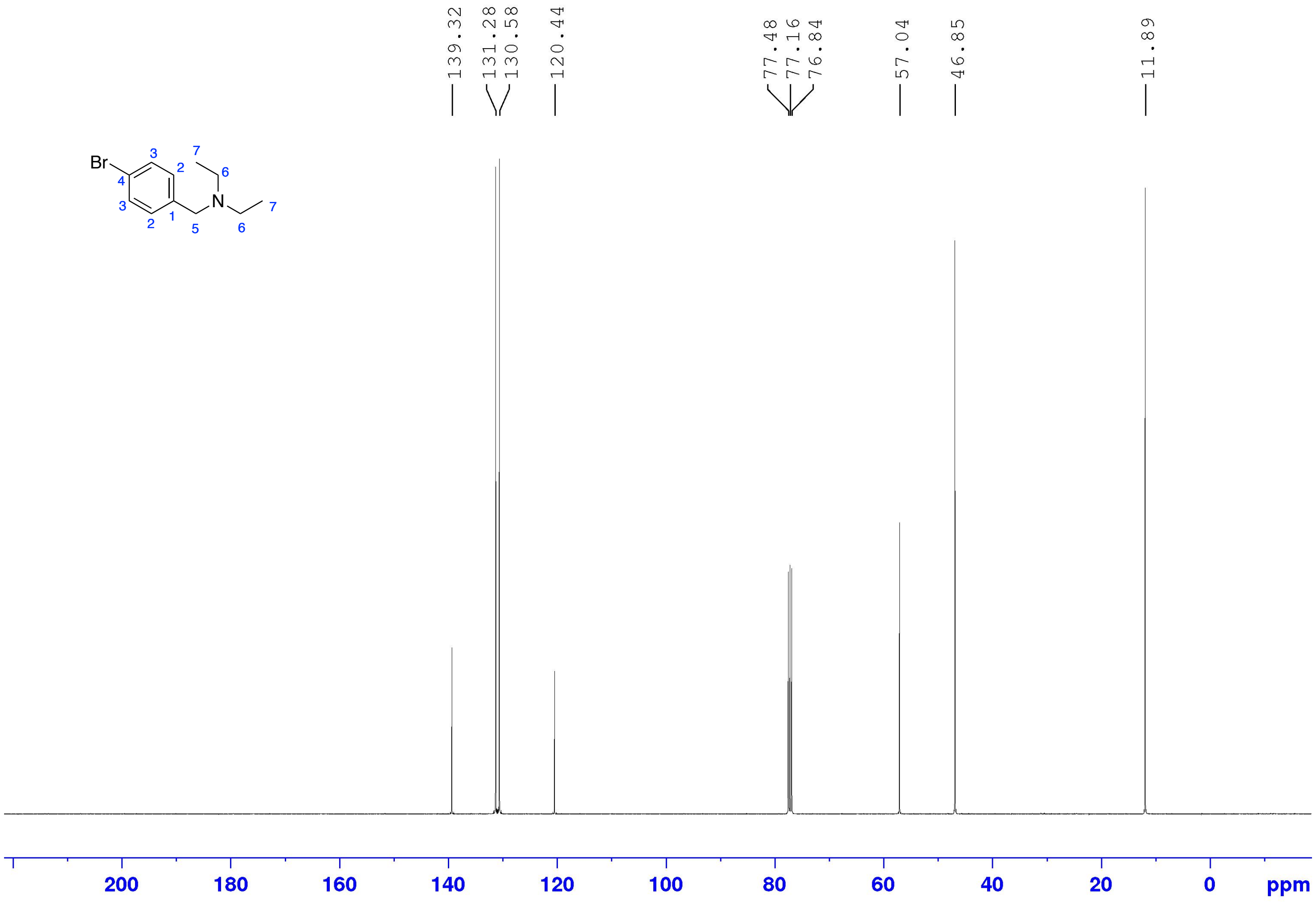

N-(4-(Dimethylsilyl)benzyl)-*N*-ethylethanamine (**S2**), ^{1}H NMR ($CDCl_3$, 500 MHz)

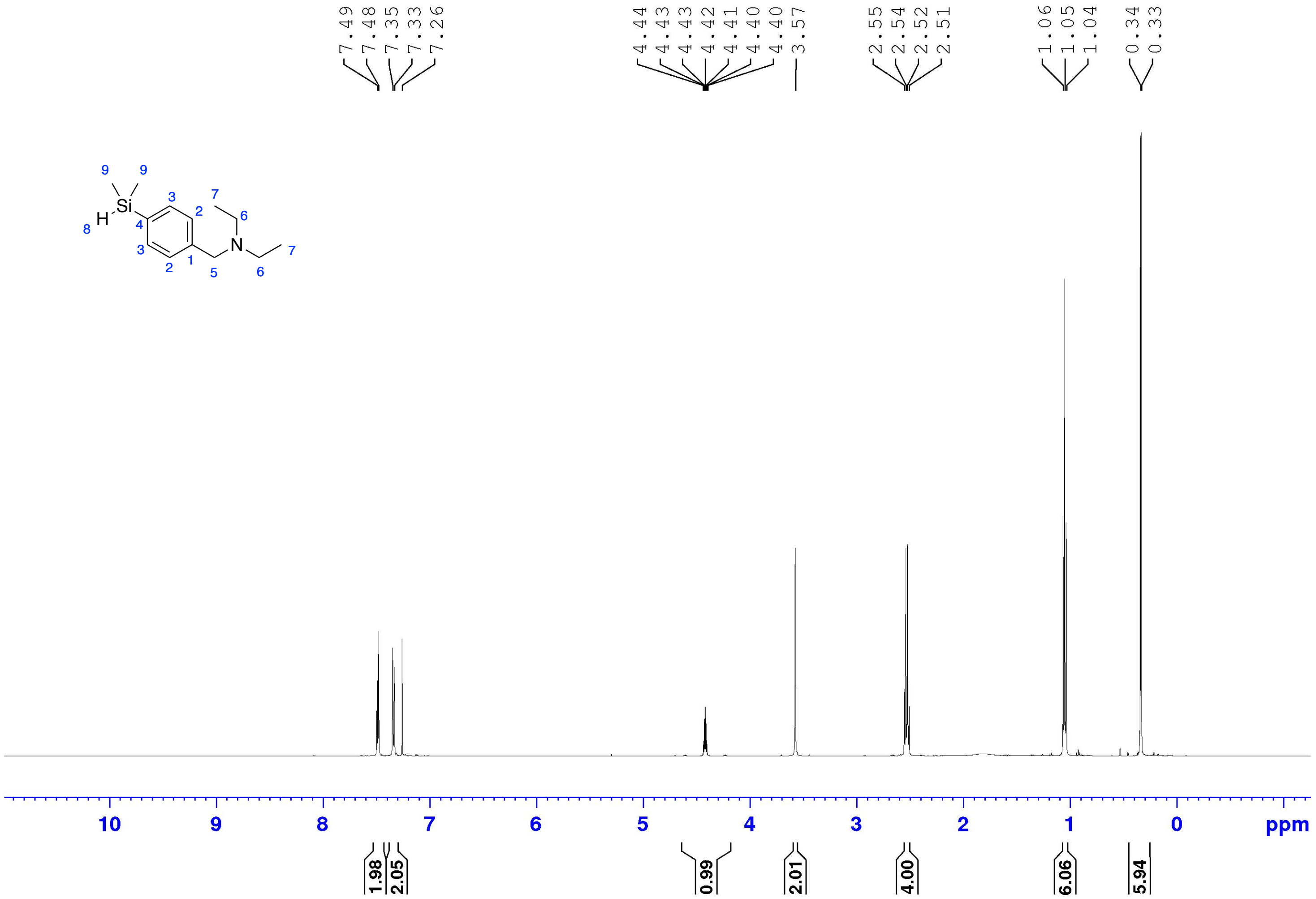

N-(4-(Dimethylsilyl)benzyl)-*N*-ethylethanamine (**S2**), ^{13}C NMR ($CDCl_3$, 126 MHz)

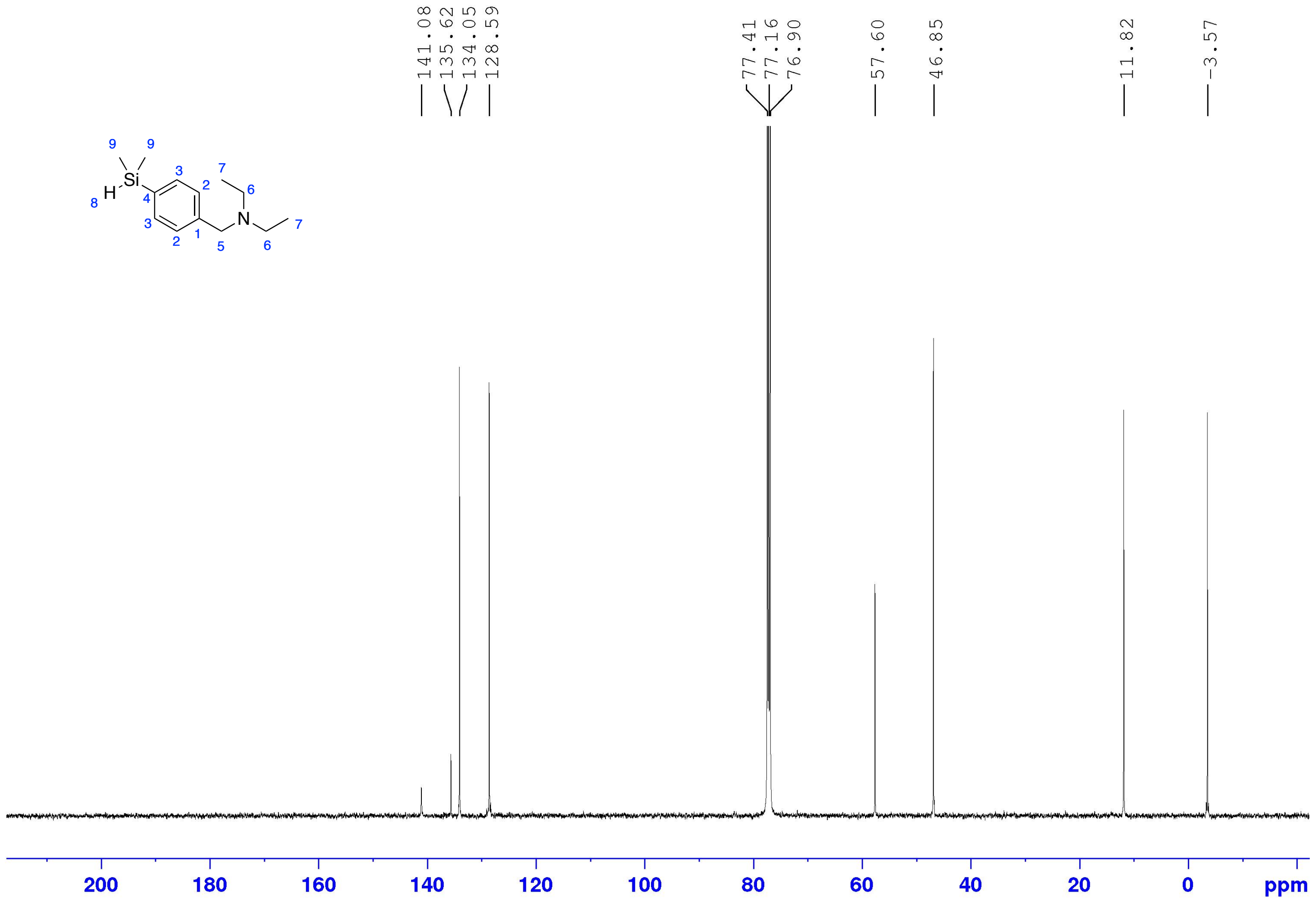

N-(4-(Diisopropylsilyl)benzyl)-*N*-ethylethanamine (**S3**), ^{1}H NMR ($CDCl_3$, 400 MHz)

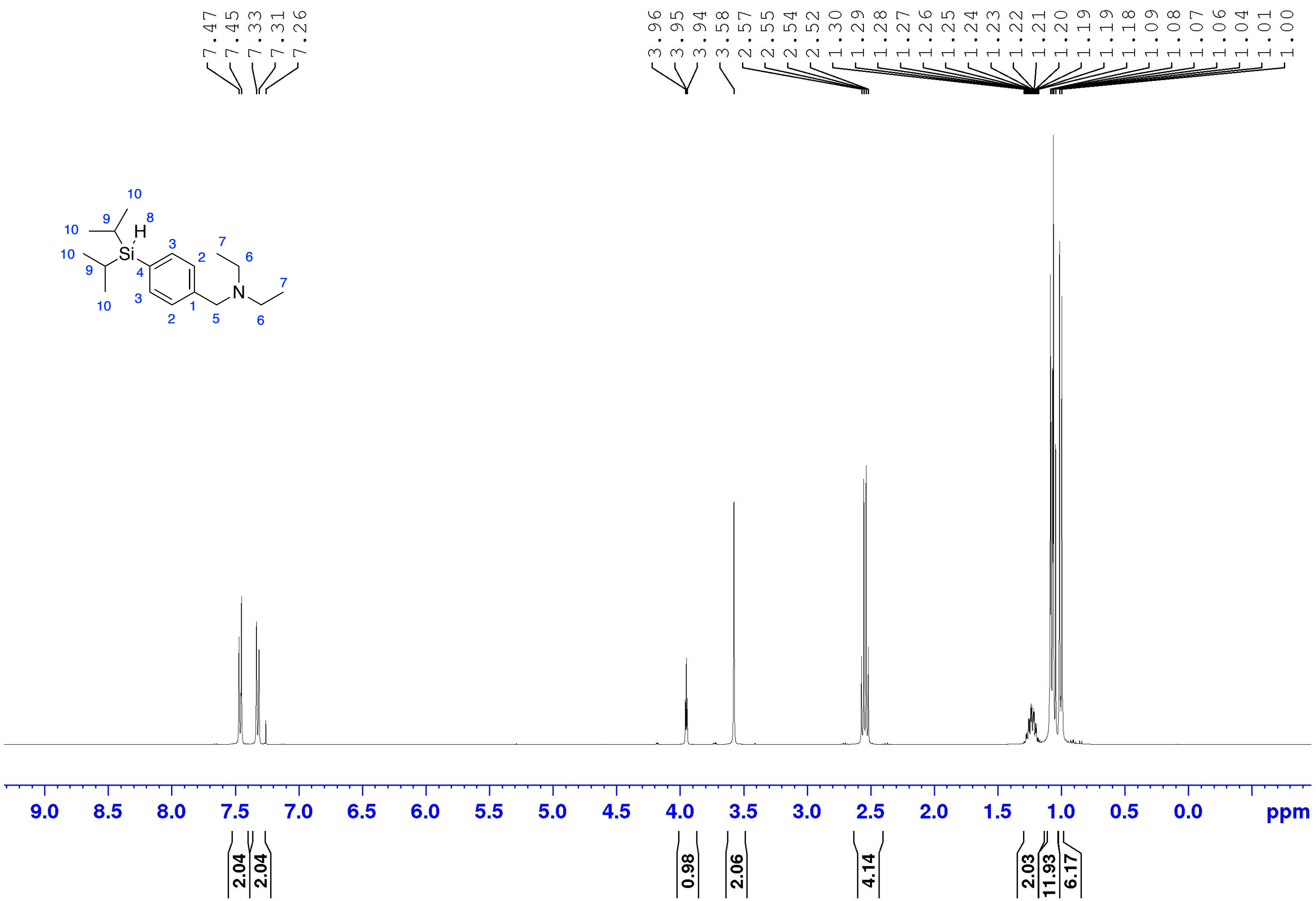

N-(4-(Diisopropylsilyl)benzyl)-*N*-ethylethanamine (**S3**), ^{13}C NMR ($CDCl_3$, 101 MHz)

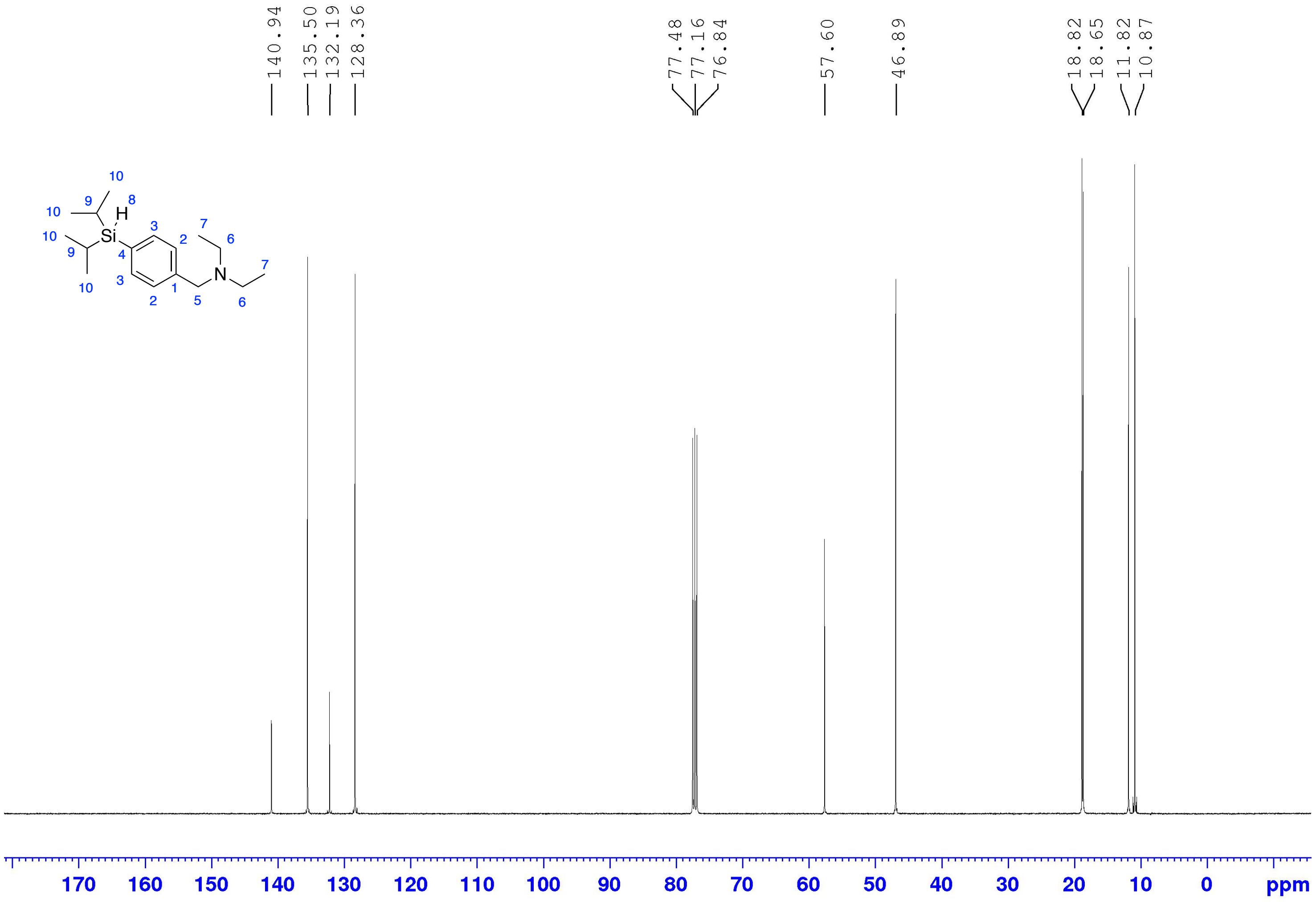

N-(4-(Diphenylsilyl)benzyl)-*N*-ethylethanamine (**S4**), ^{1}H NMR ($CDCl_3$, 500 MHz)

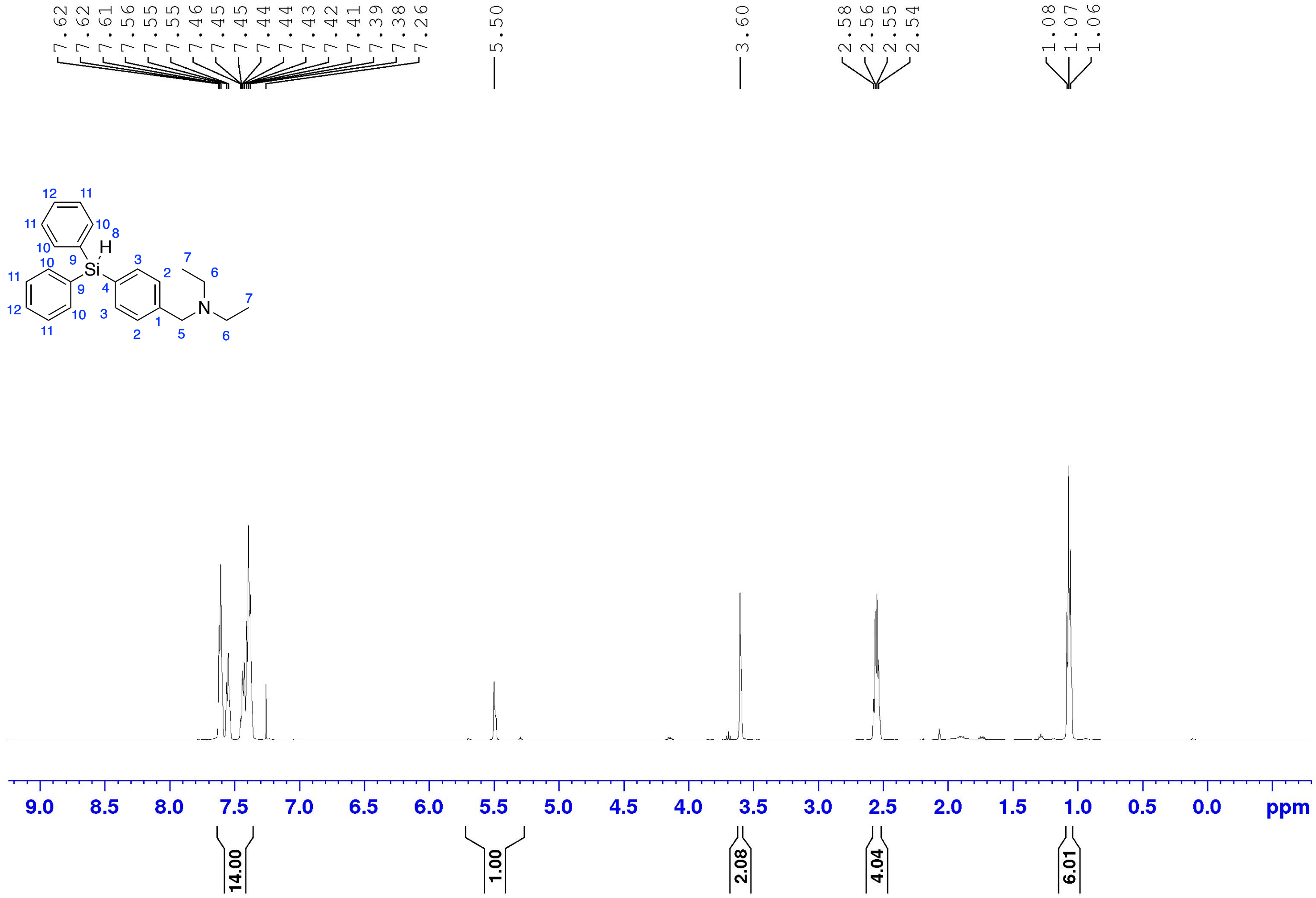

N-(4-(Diphenylsilyl)benzyl)-*N*-ethylethanamine (**S4**), ^{13}C NMR ($CDCl_3$, 126 MHz)

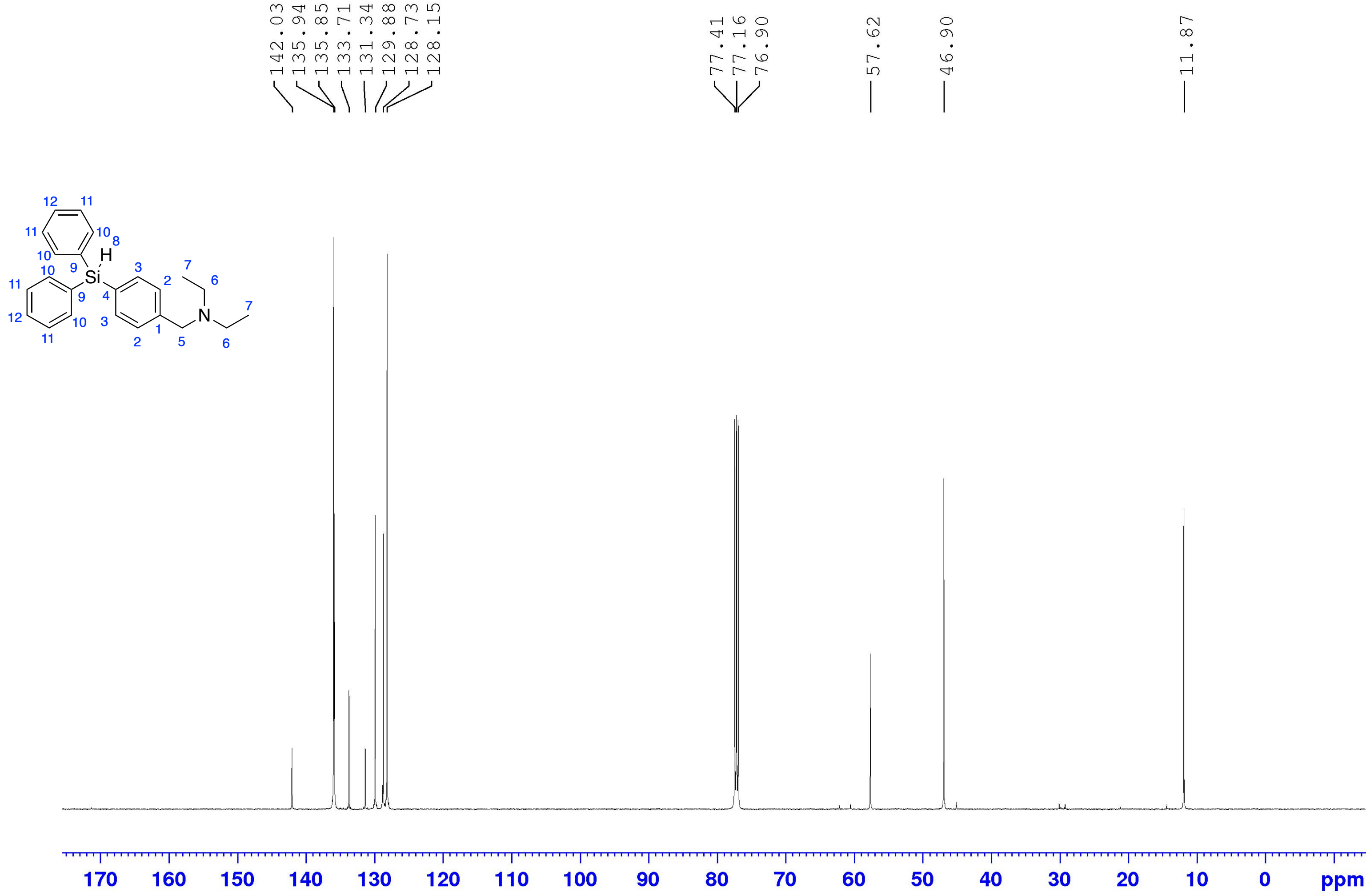

N-(4-(Diethylsilyl)benzyl)-*N*-ethylethanamine (**S5**), ^{1}H NMR ($CDCl_3$, 400 MHz)

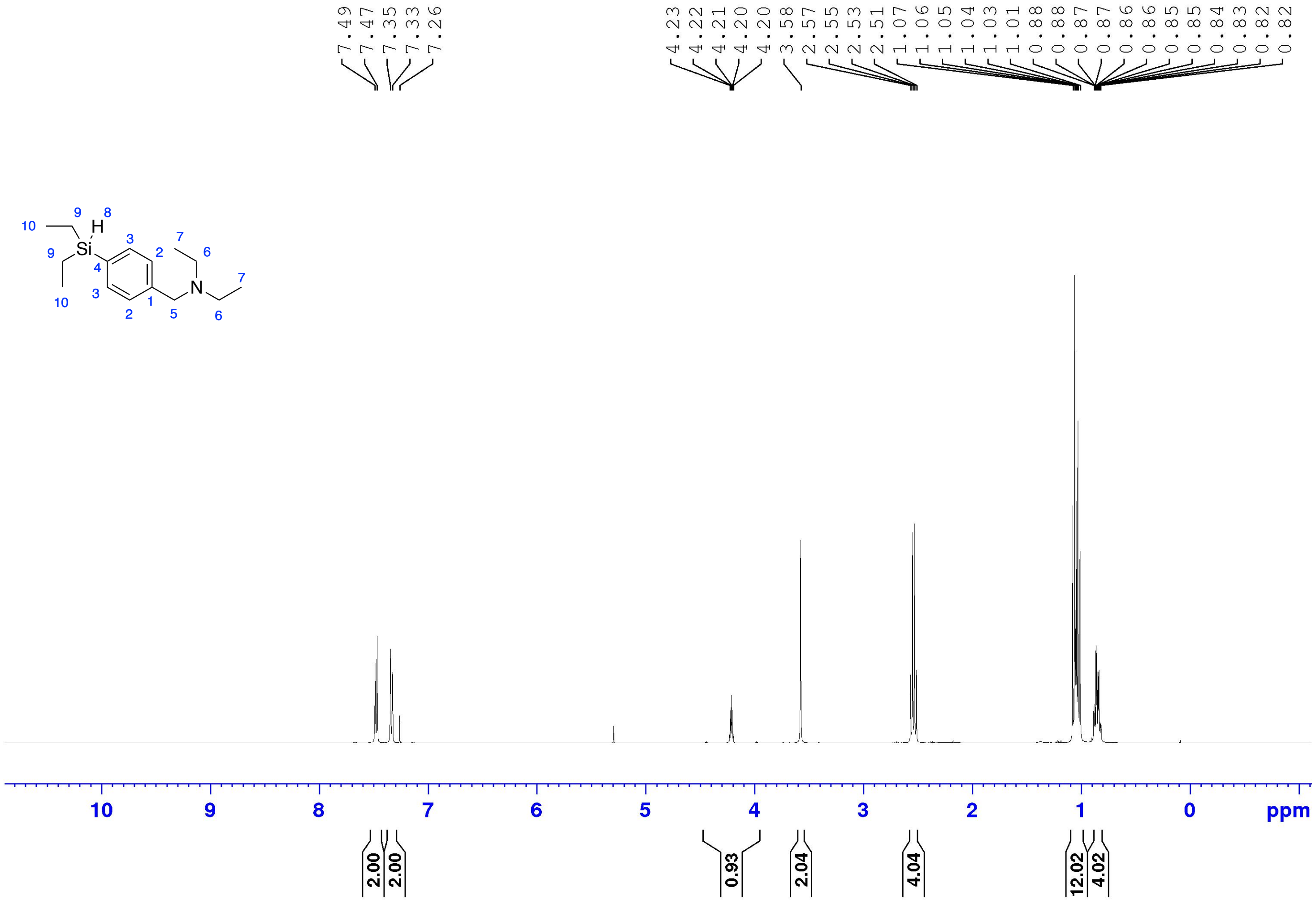

N-(4-(Diethylsilyl)benzyl)-*N*-ethylethanamine (**S5**), ^{13}C NMR ($CDCl_3$, 101 MHz)

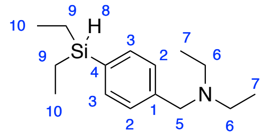

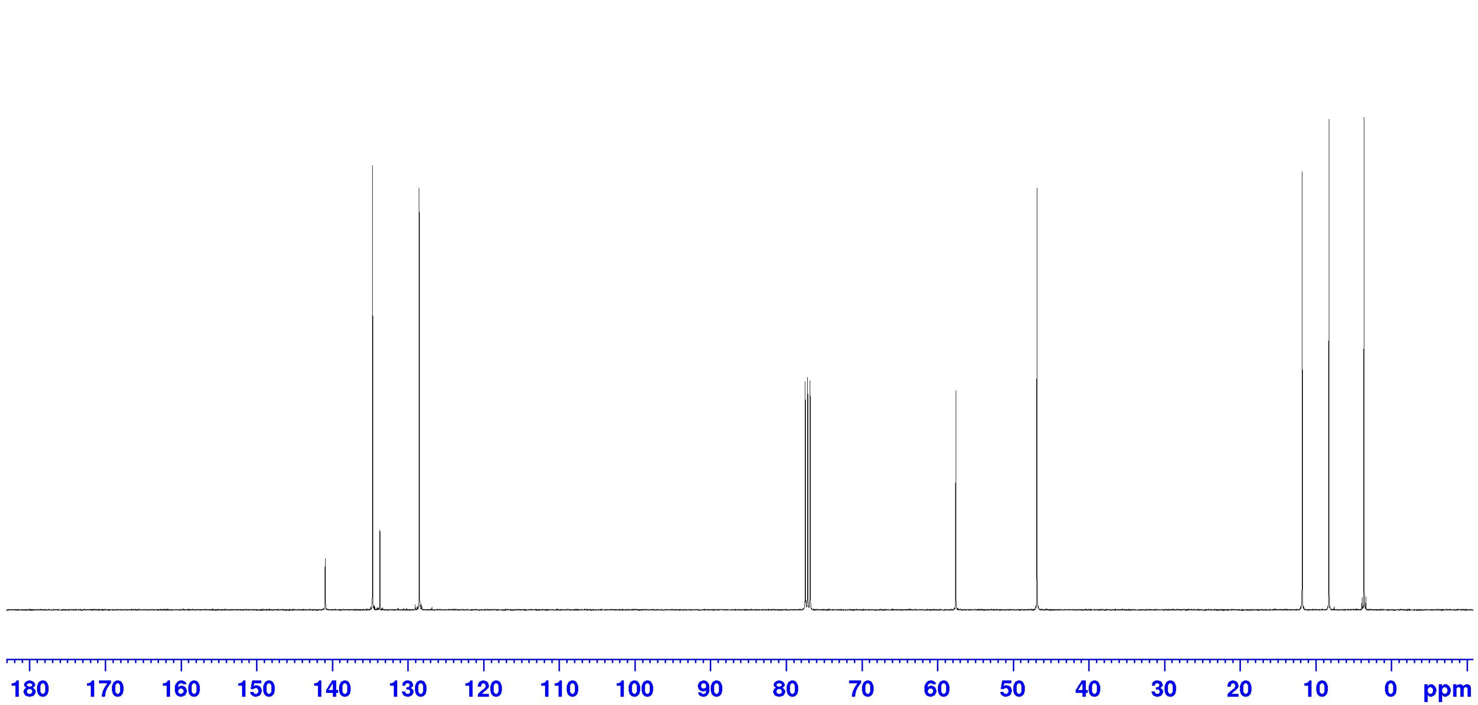

N-(4-(Dimethylsilyl)benzyl)-*N*,*N*-diethyl-2-hydroxyethanammonium bromide (**4**), ^{1}H NMR ($CDCl_3$, 500 MHz)

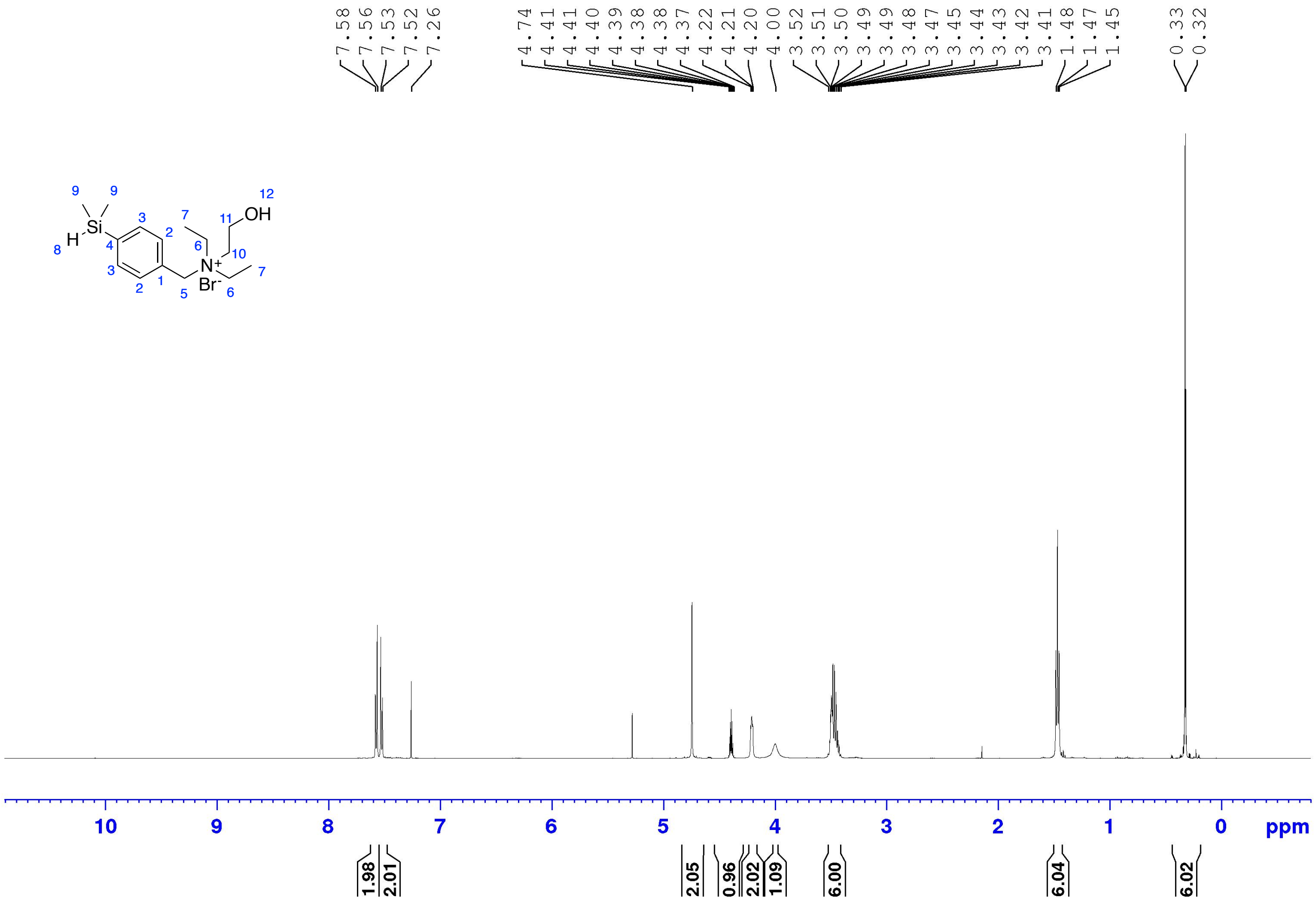

N-(4-(Dimethylsilyl)benzyl)-*N*,*N*-diethyl-2-hydroxyethanammonium bromide (**4**), ^{13}C NMR ($CDCl_3$, 126 MHz)

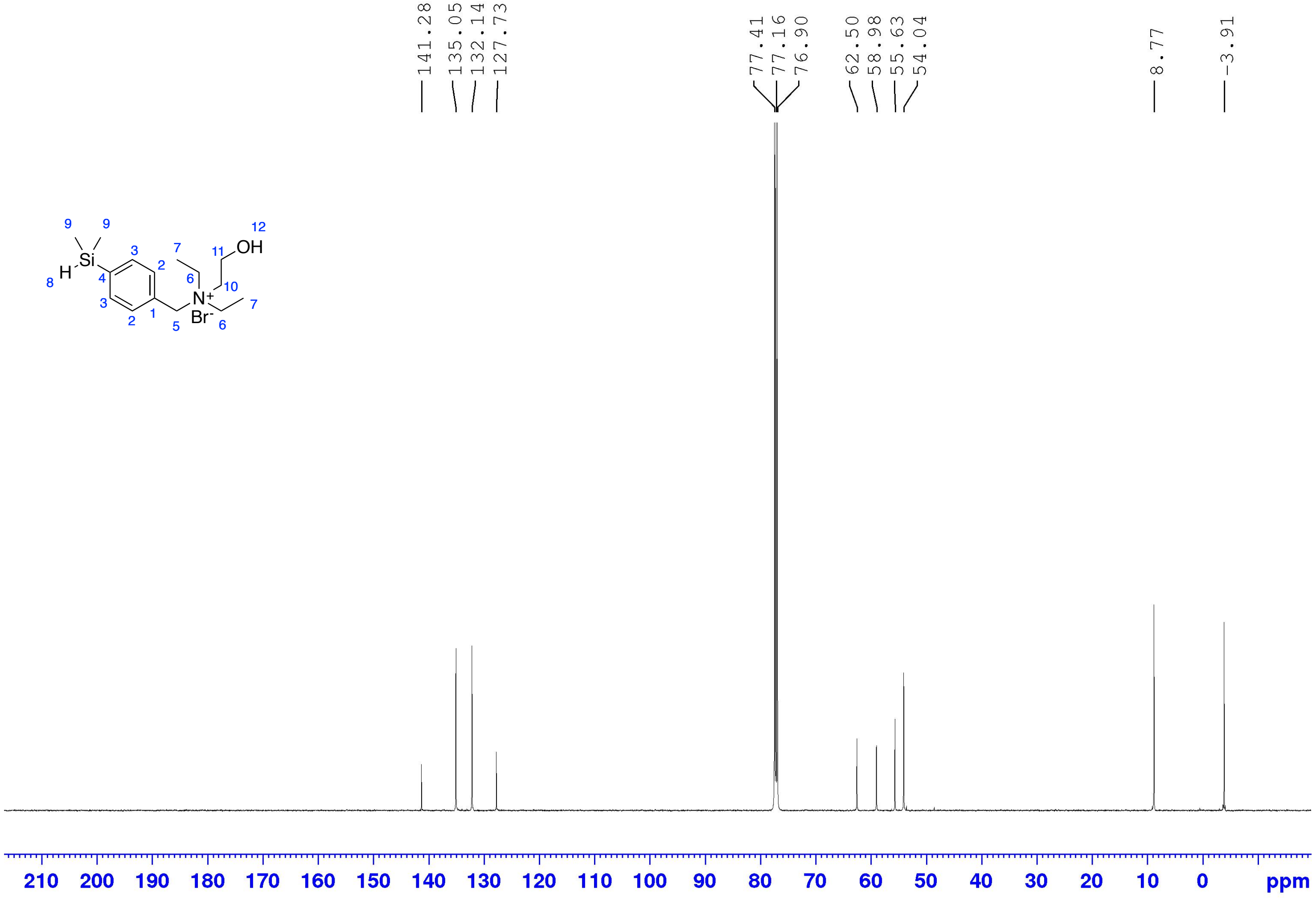

N-(4-(Diisopropylsilyl)benzyl)-*N*,*N*-diethyl-2-hydroxyethanammonium bromide (**6**), ^{1}H NMR ($CDCl_3$, 500 MHz)

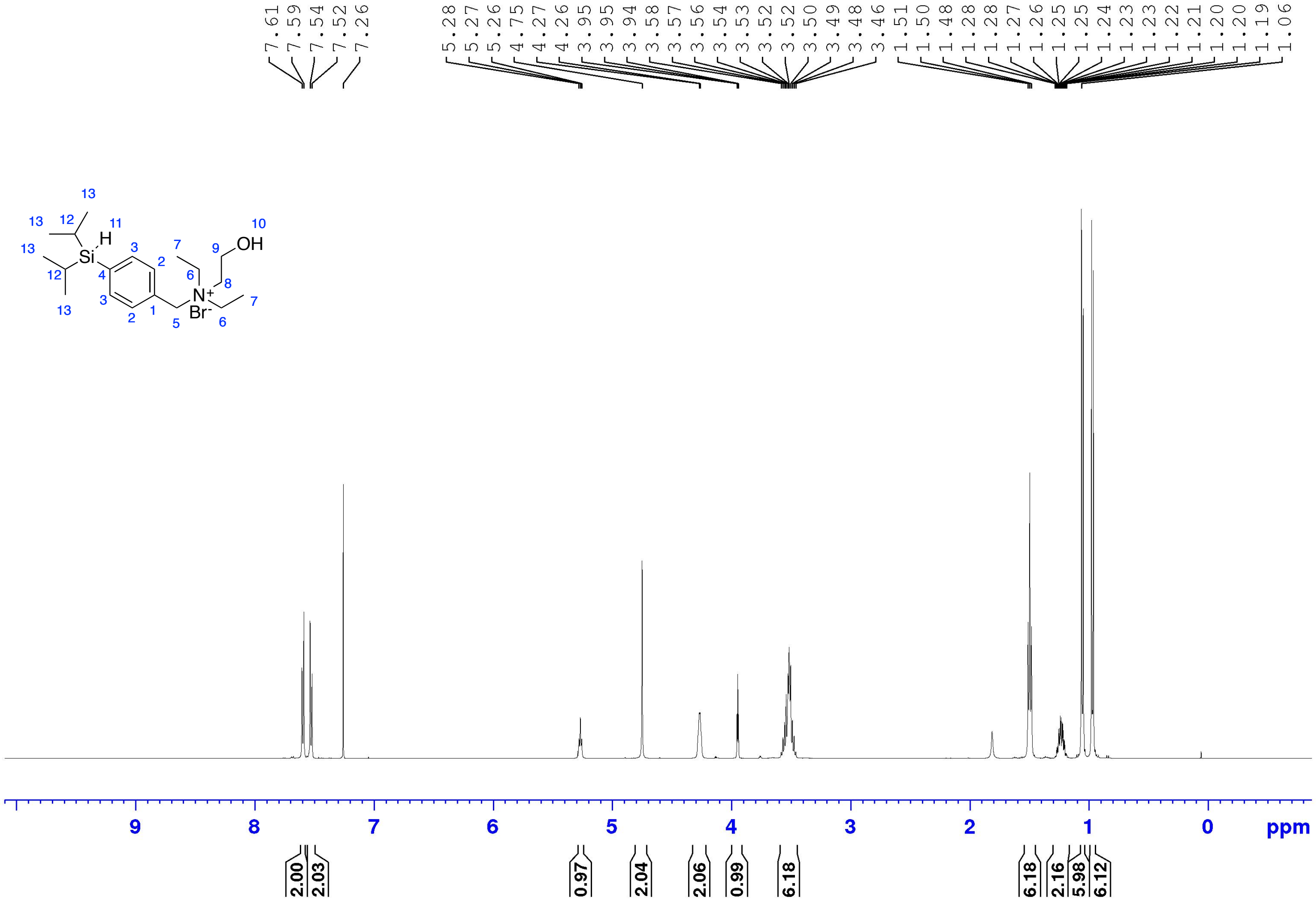

N-(4-(Diisopropylsilyl)benzyl)-*N*,*N*-diethyl-2-hydroxyethanammonium bromide (**6**), ^{13}C NMR ($CDCl_3$, 126 MHz)

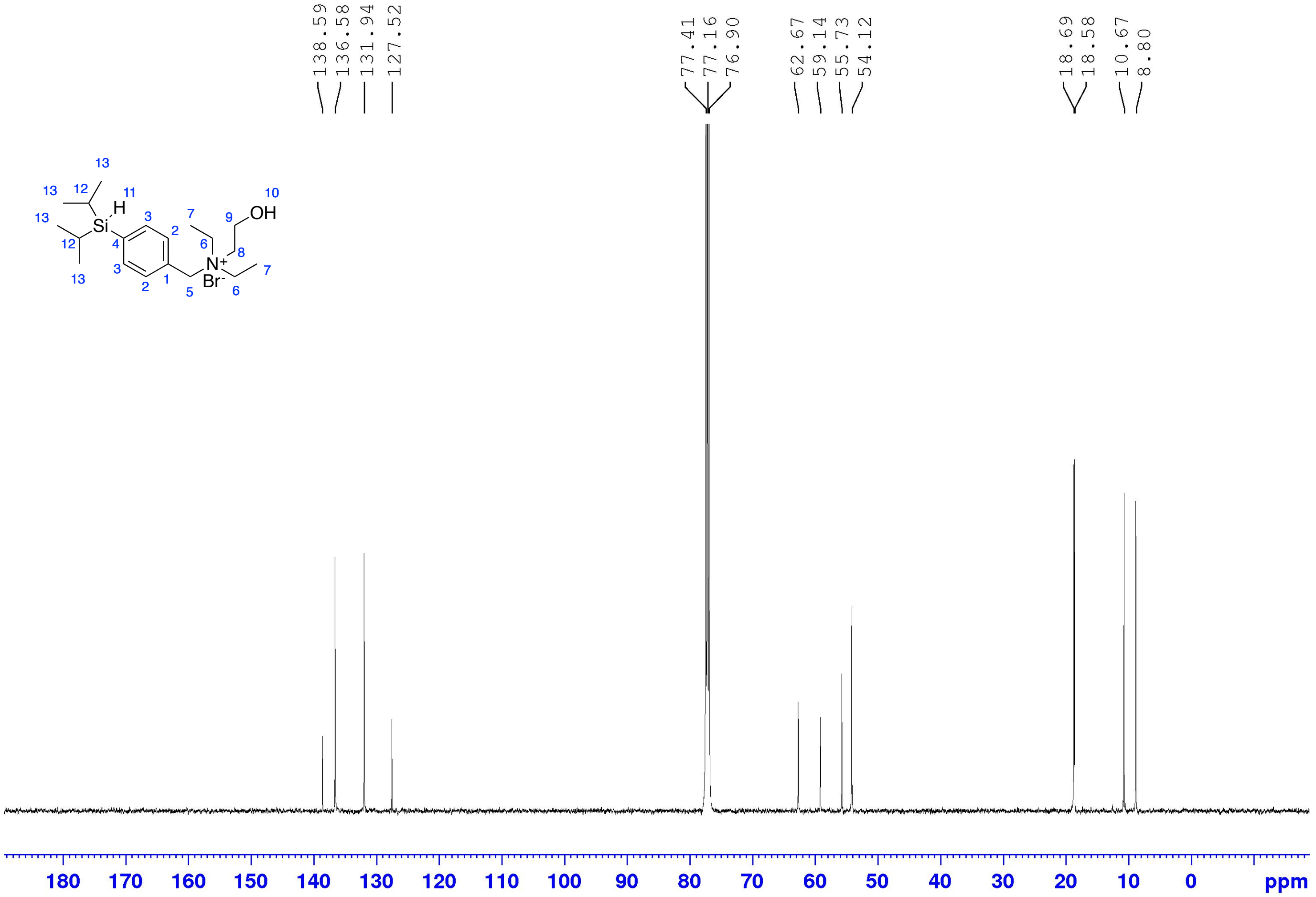

N-(4-(Diphenylsilyl)benzyl)-*N*,*N*-diethyl-2-hydro-xyethanammonium bromide (**7**), ^{1}H NMR (CD_3OD, 500 MHz)

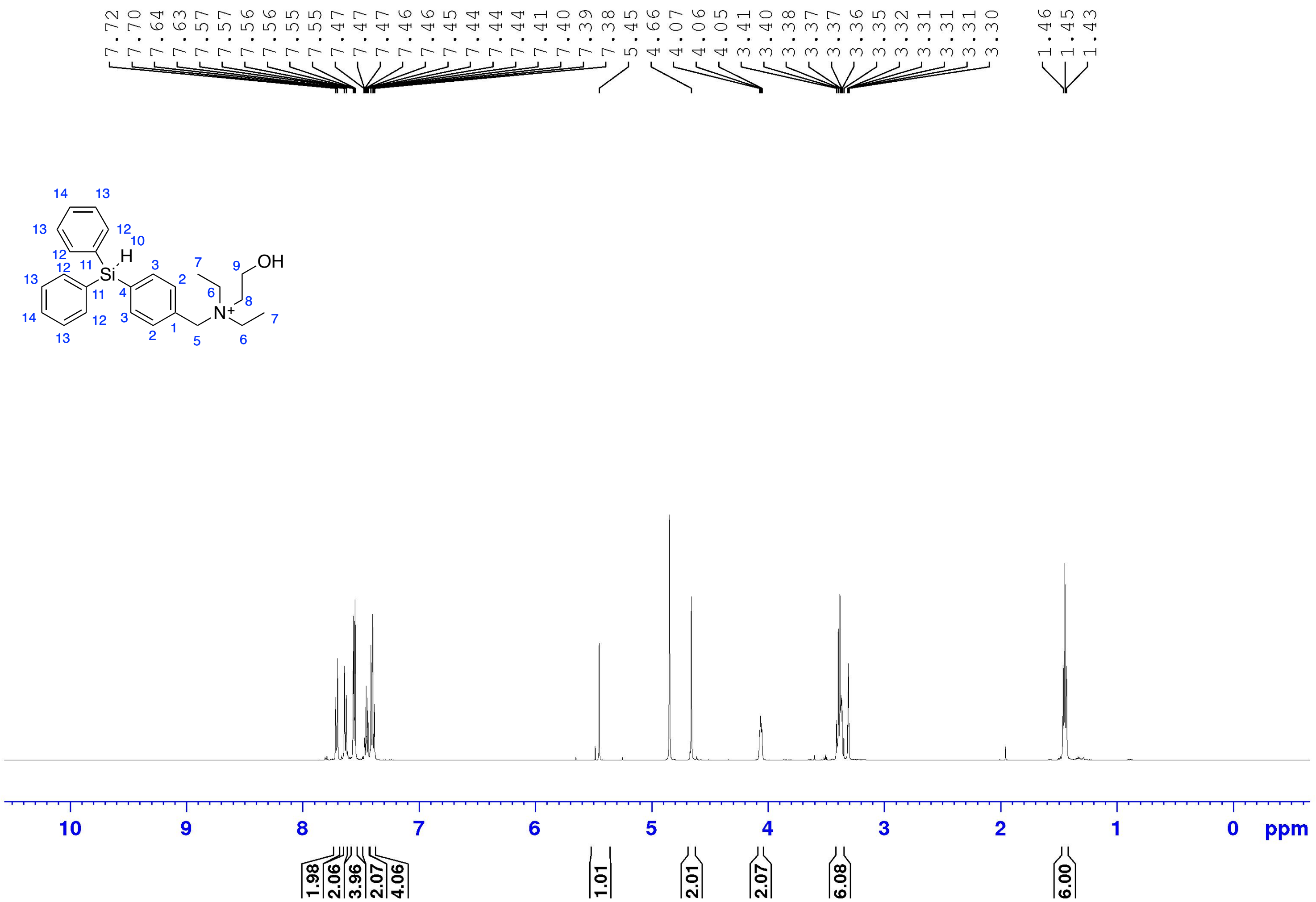

N-(4-(Diphenylsilyl)benzyl)-*N*,*N*-diethyl-2-hydro-xyethanammonium bromide (**7**), ^{13}C NMR (CD_3OD, 126 MHz)

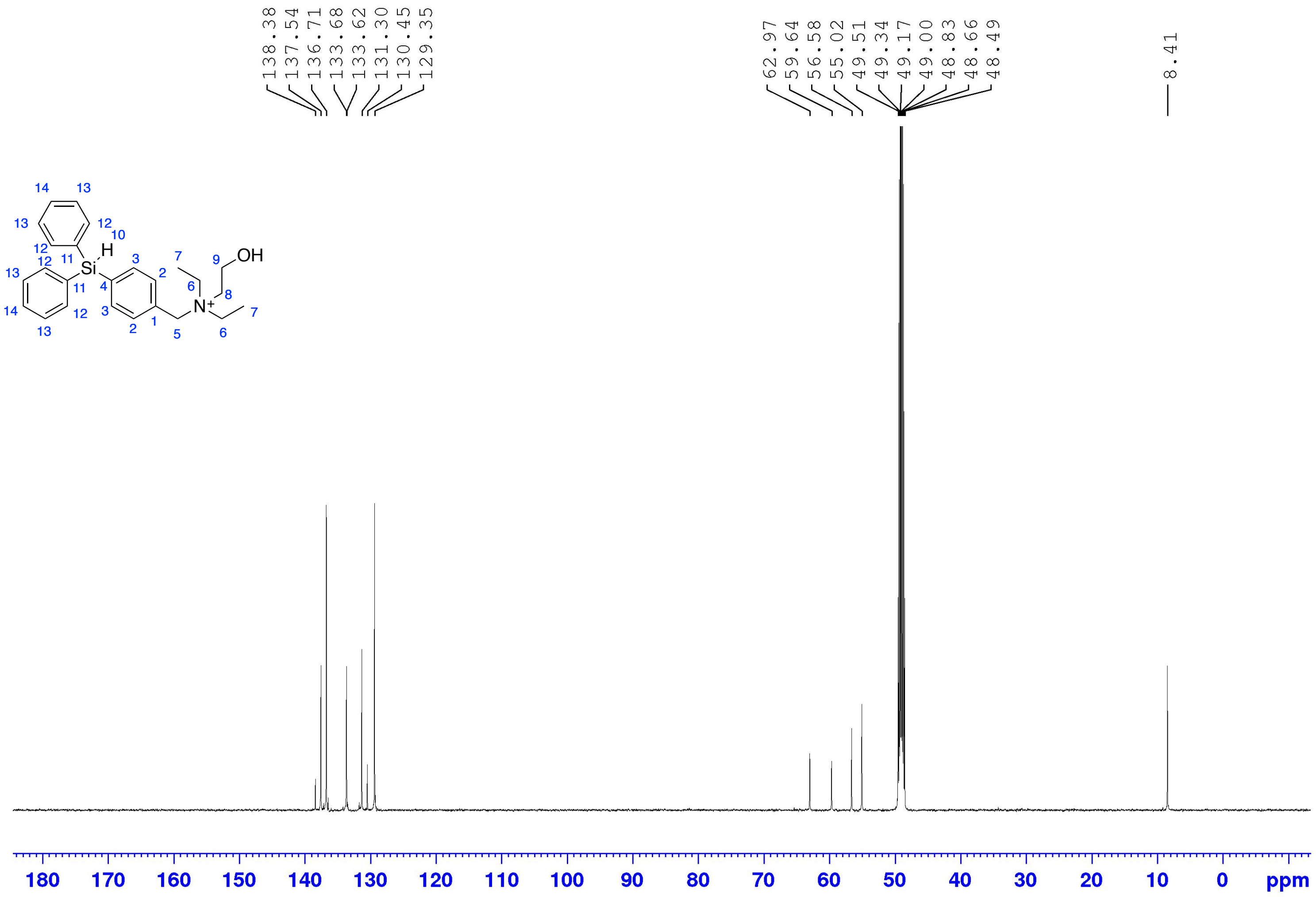

N-(4-(Diethylsilyl)benzyl)-*N*,*N*-diethyl-2-hydroxyethanammonium bromide (**5**), ^{1}H NMR ($CDCl_3$, 500 MHz)

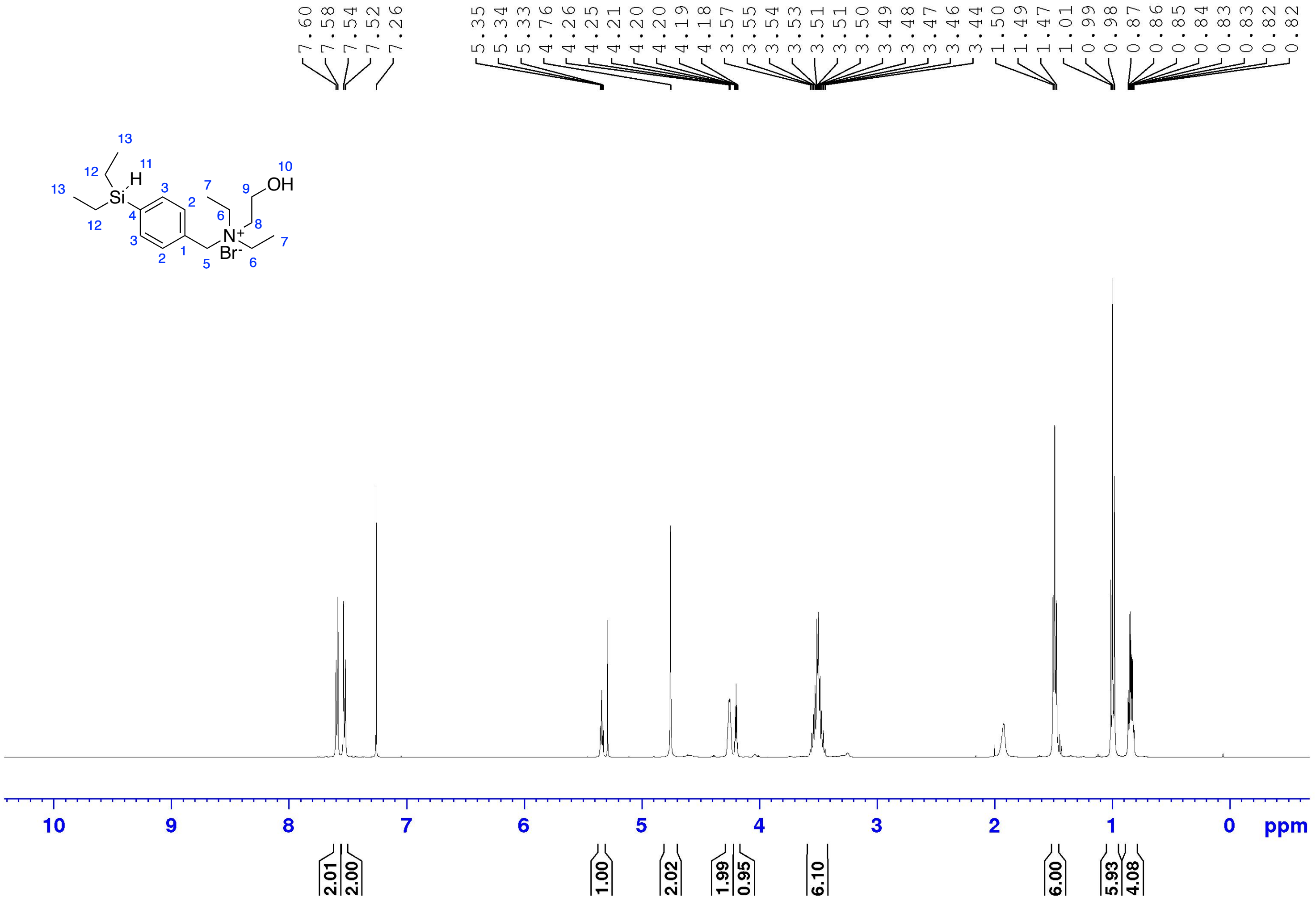

N-(4-(Diethylsilyl)benzyl)-*N*,*N*-diethyl-2-hydroxyethanammonium bromide (**5**), ^{13}C NMR ($CDCl_3$, 126 MHz)

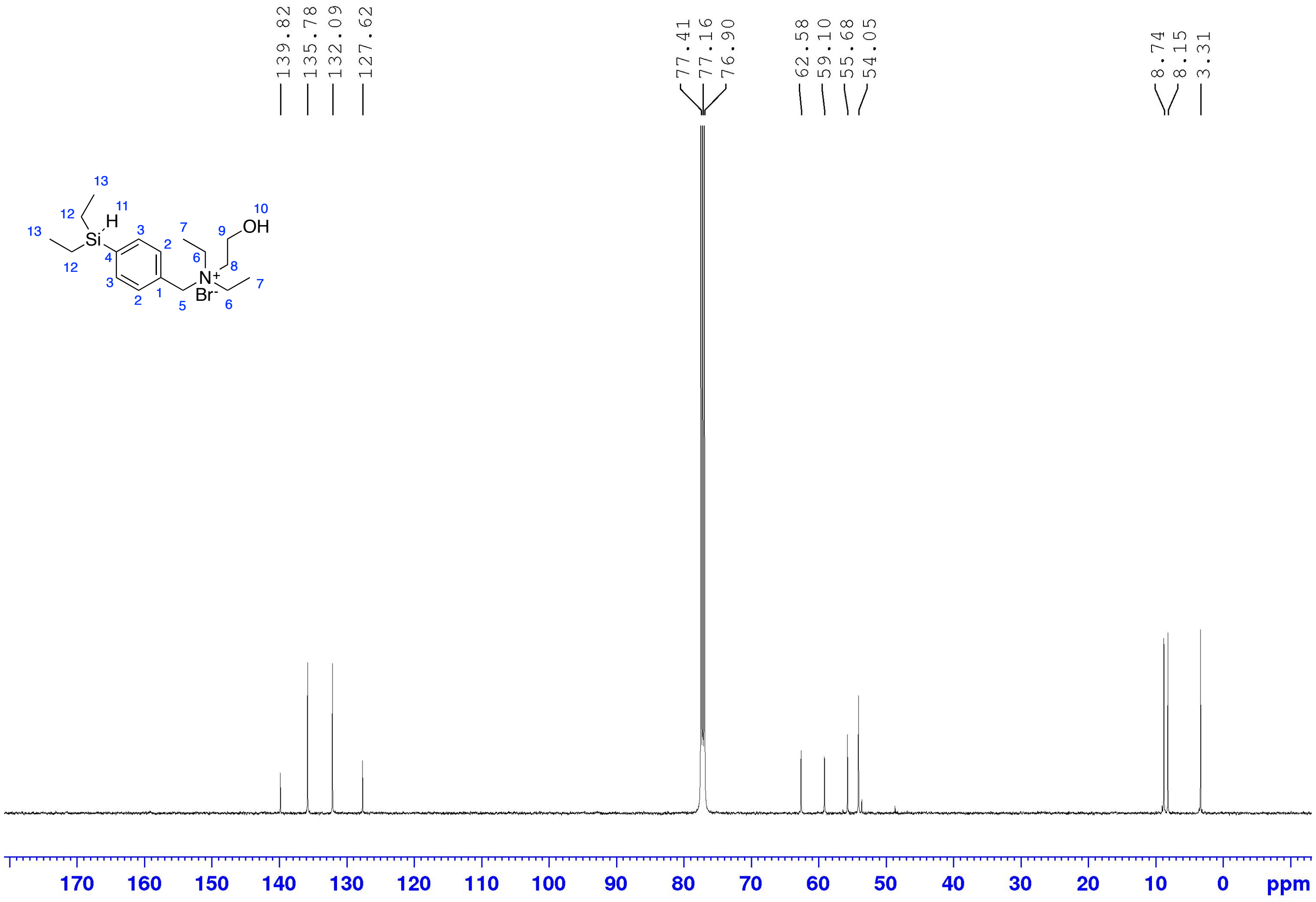

16-Bromo-3,6,9,12-tetraoxahexadecane (**S6**), ^{1}H NMR ($CDCl_3$, 400 MHz)

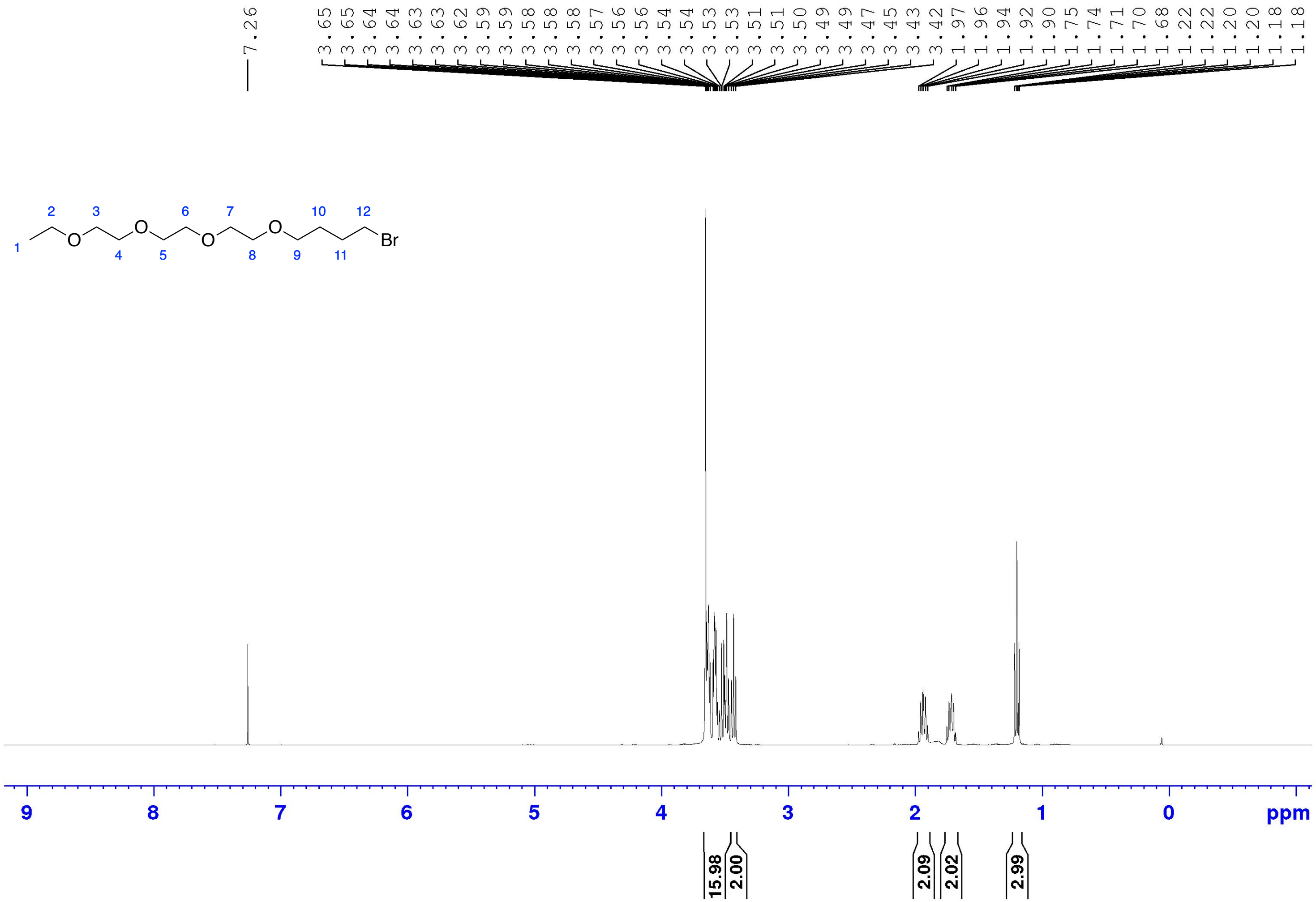

16-Bromo-3,6,9,12-tetraoxahexadecane (**S6**), ^{13}C NMR ($CDCl_3$, 101 MHz)

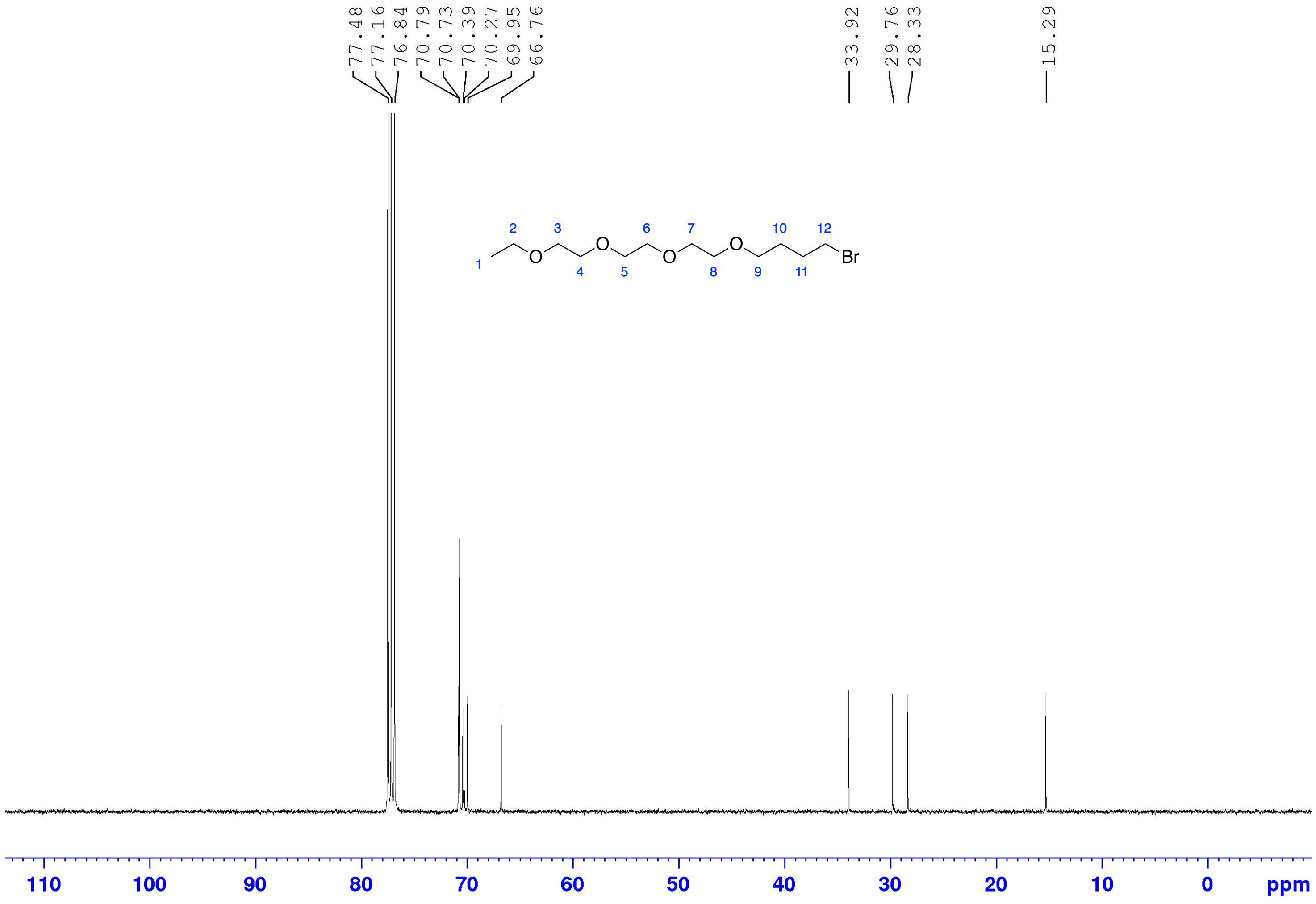

2-Methyl-7,10,13,16-tetraoxa-2-silaoctadecane (**3**), ^{1}H NMR ($CDCl_3$, 400 MHz)

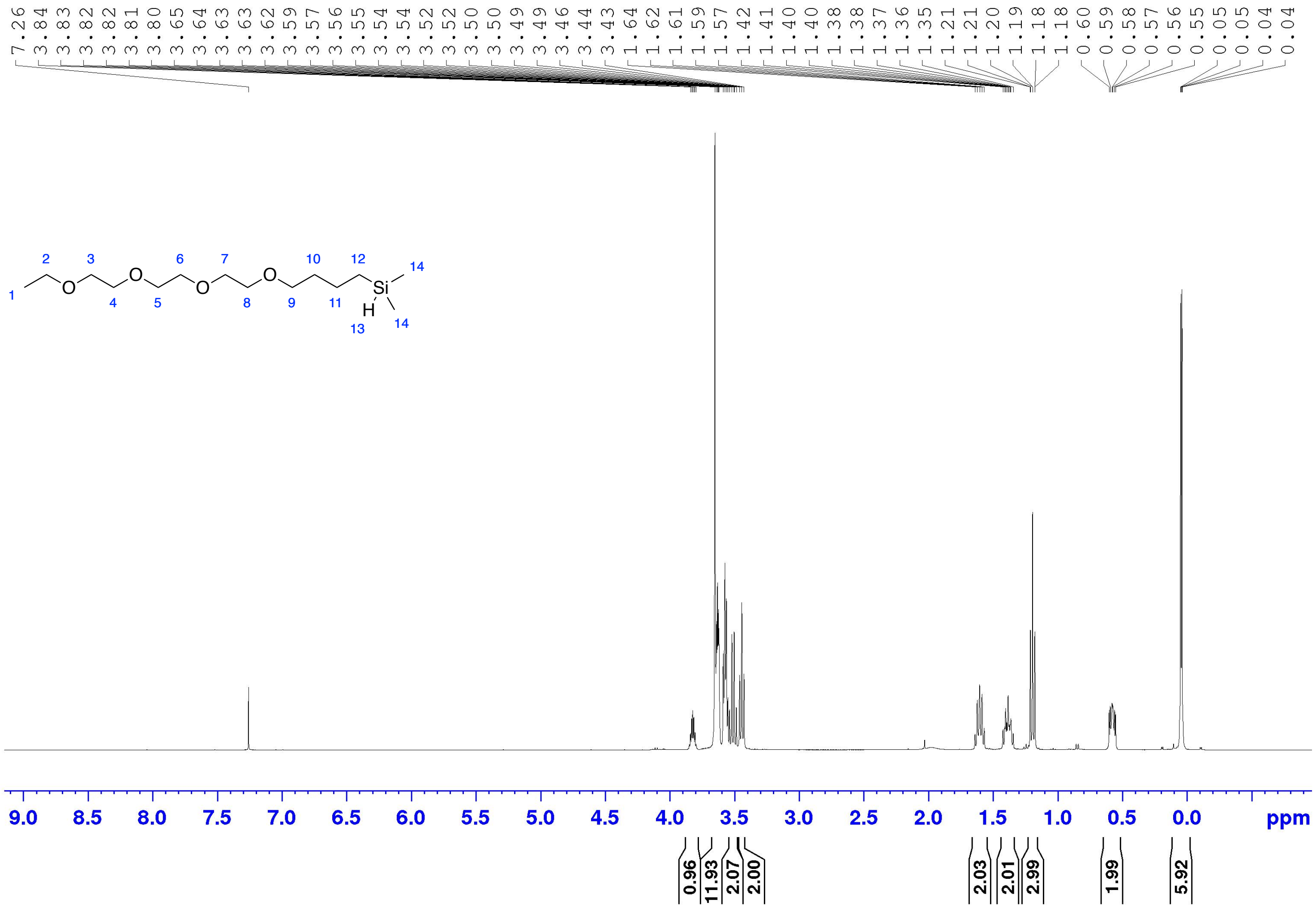

2-Methyl-7,10,13,16-tetraoxa-2-silaoctadecane (**3**), ^{13}C NMR ($CDCl_3$, 101 MHz)

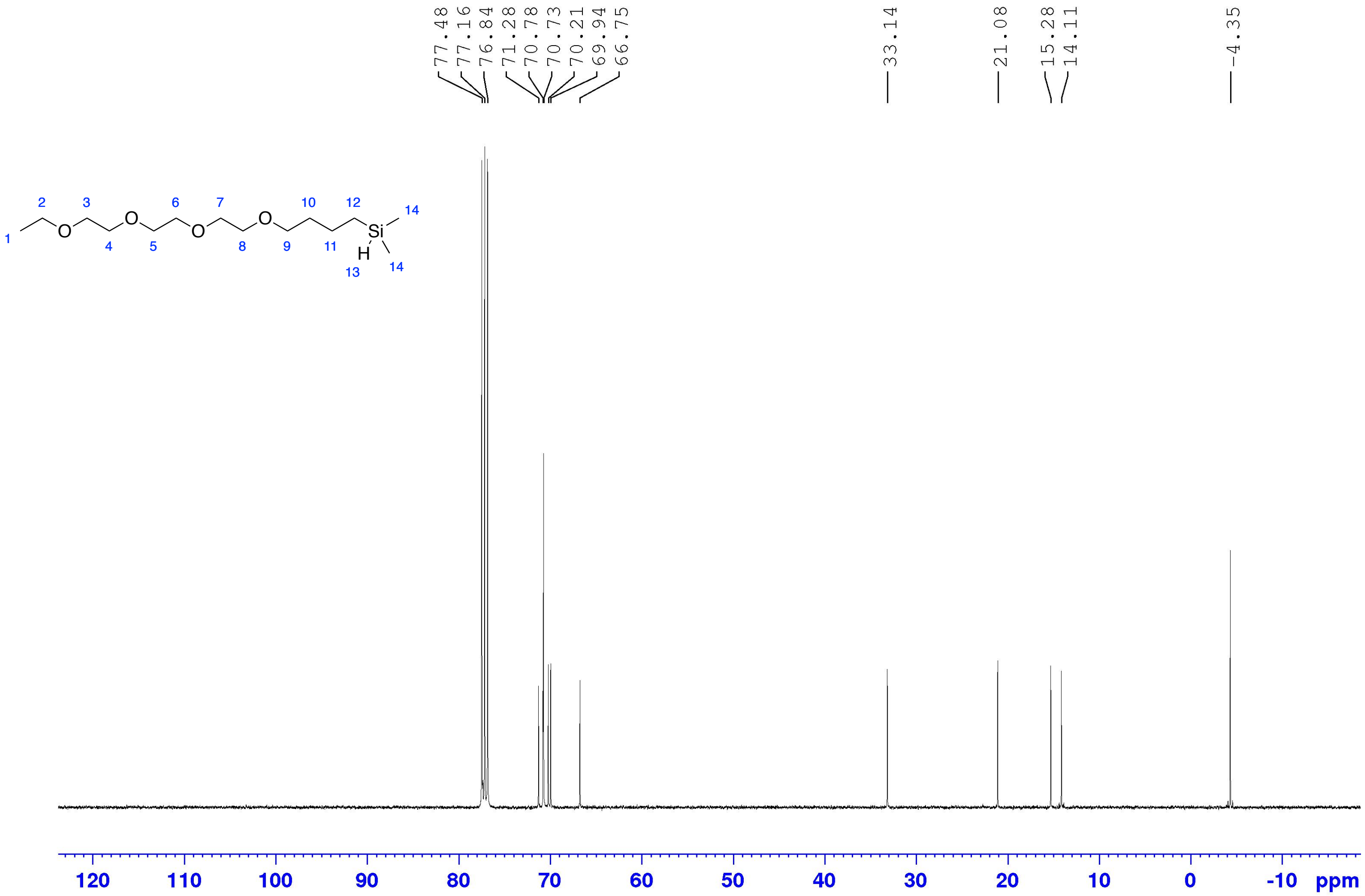

4-(Dimethylsilyl)benzoic acid (**S7**), ^{1}H NMR ($CDCl_3$, 500 MHz)

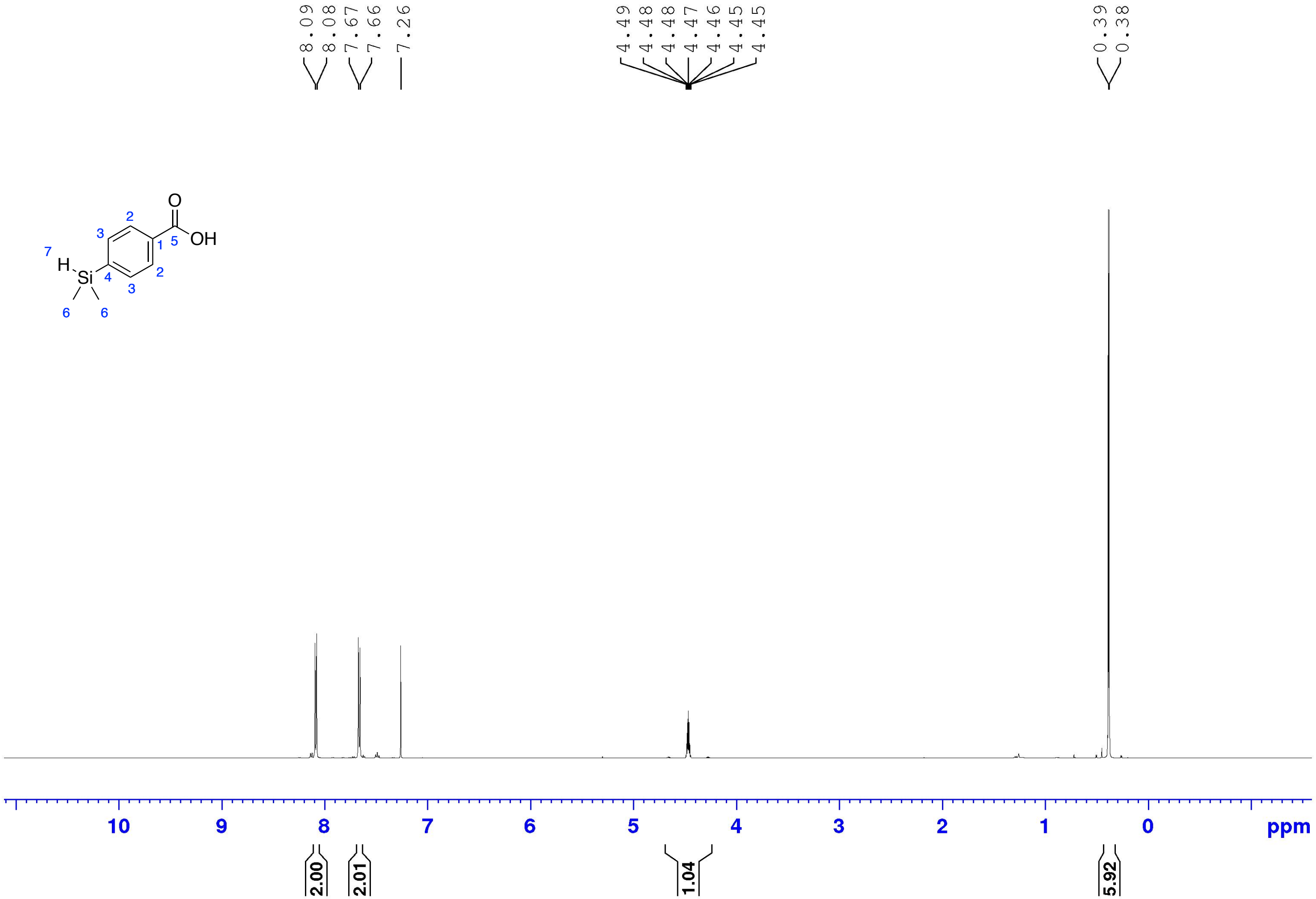

4-(Dimethylsilyl)benzoic acid (**S7**), ^{13}C NMR ($CDCl_3$, 126 MHz)

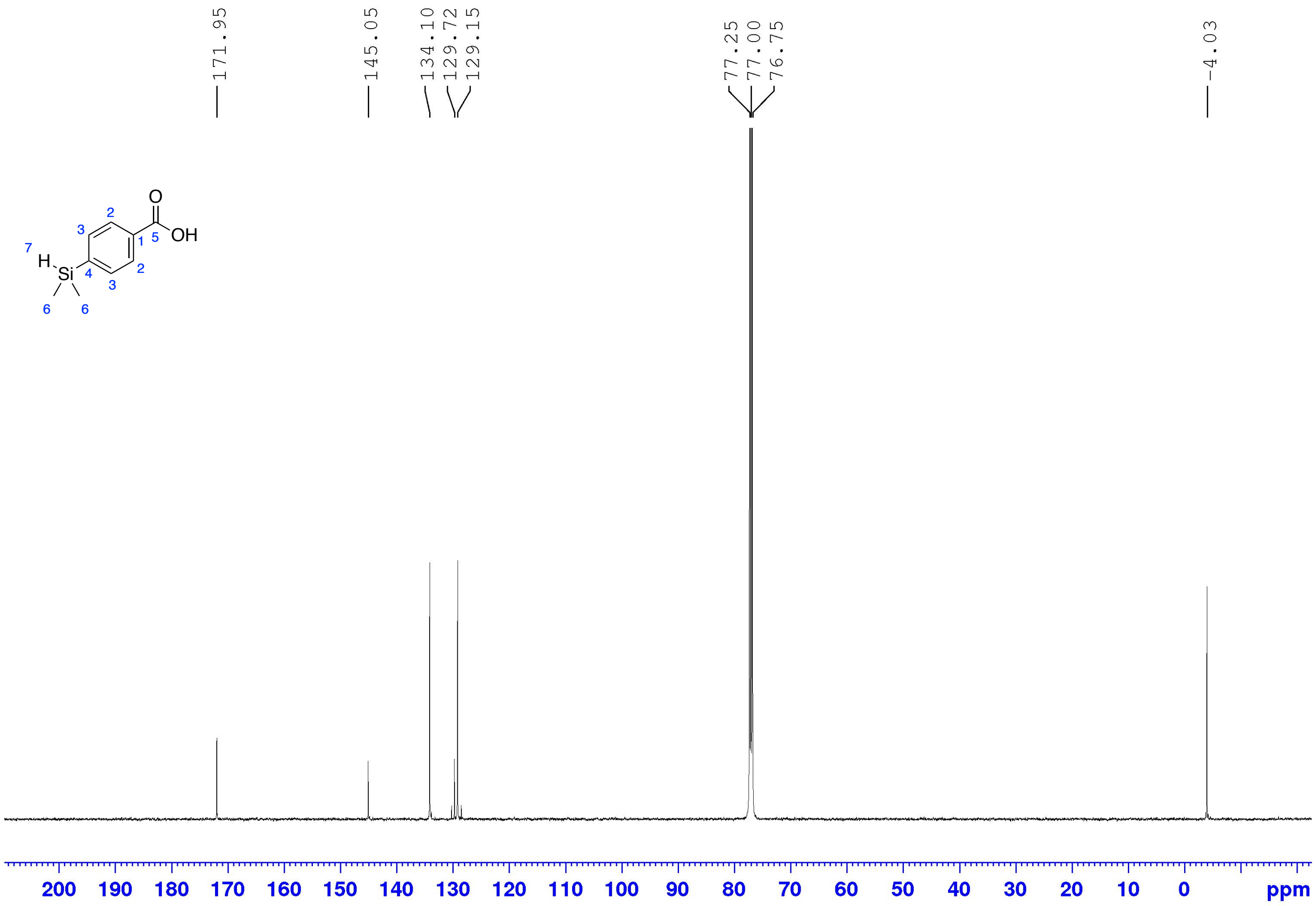

4-(Dimethylsilyl)-*N*-(2-(2-(2-hydroxyethoxy)ethoxy)ethyl)benzamide (**8**), ^{1}H NMR ($CDCl_3$, 500 MHz)

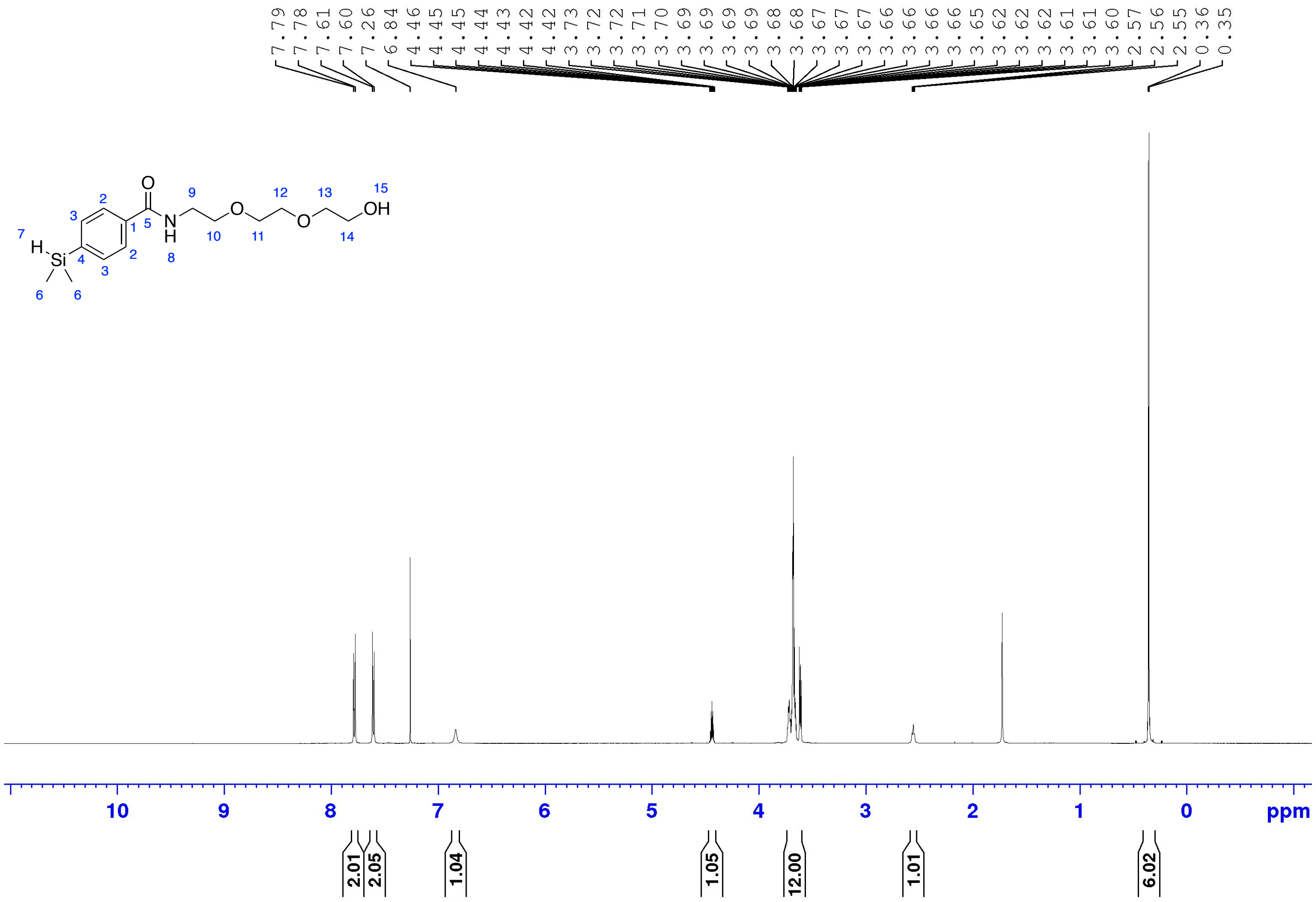

4-(Dimethylsilyl)-*N*-(2-(2-(2-hydroxyethoxy)ethoxy)ethyl)benzamide (**8**), ^{13}C NMR ($CDCl_3$, 126 MHz)

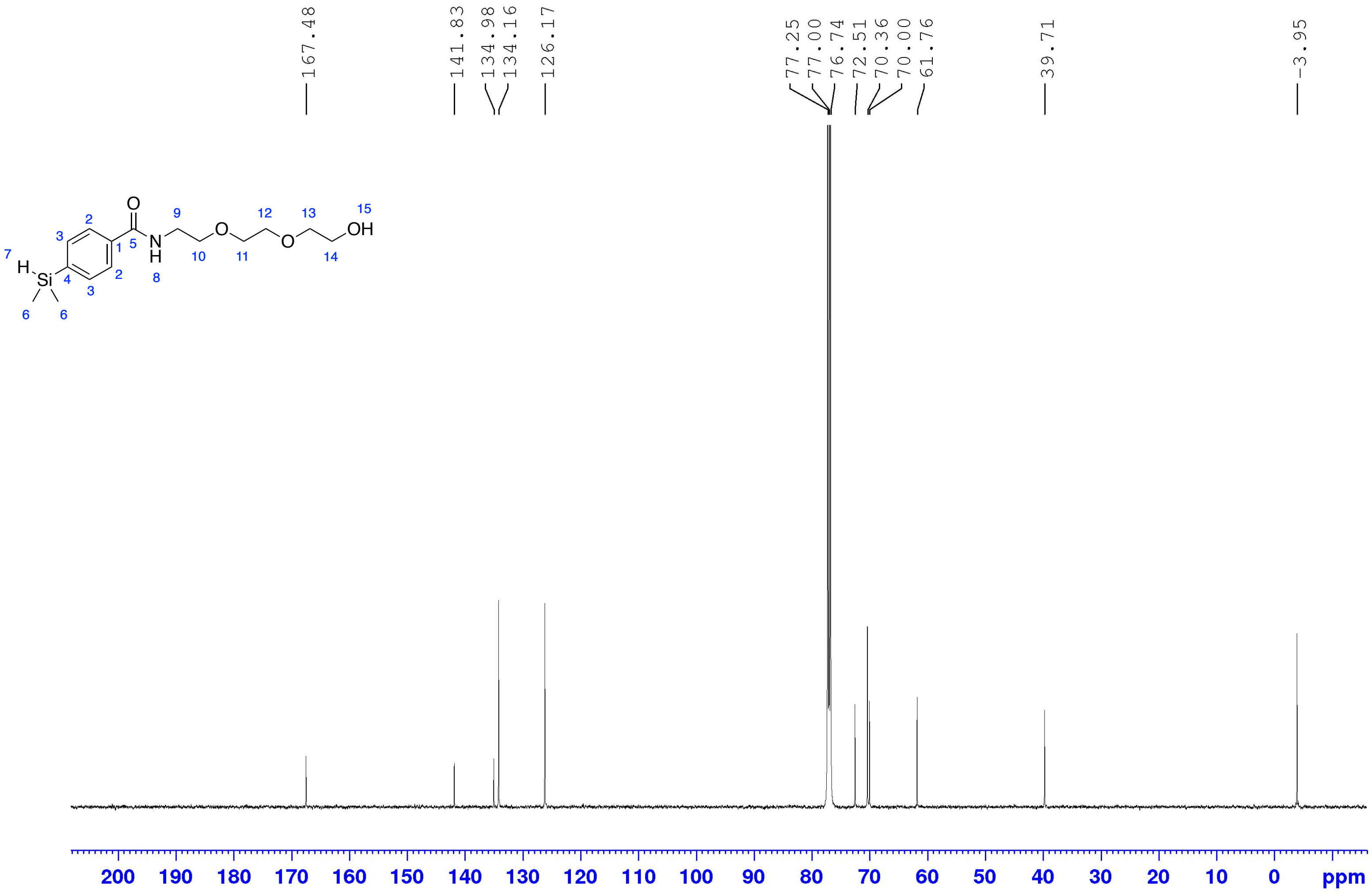

4-(Dimethylsilyl)-*N*-(15-oxo-19-((3a*S*,4*S*,6a*R*)-2-oxohexahydro-1*H*-thieno[3,4-*d*]imidazol-4-yl)-4,7,10-trioxa-14-azanonadecyl)benzamide (**29**), ^{1}H NMR ($CDCl_3$, 500 MHz)

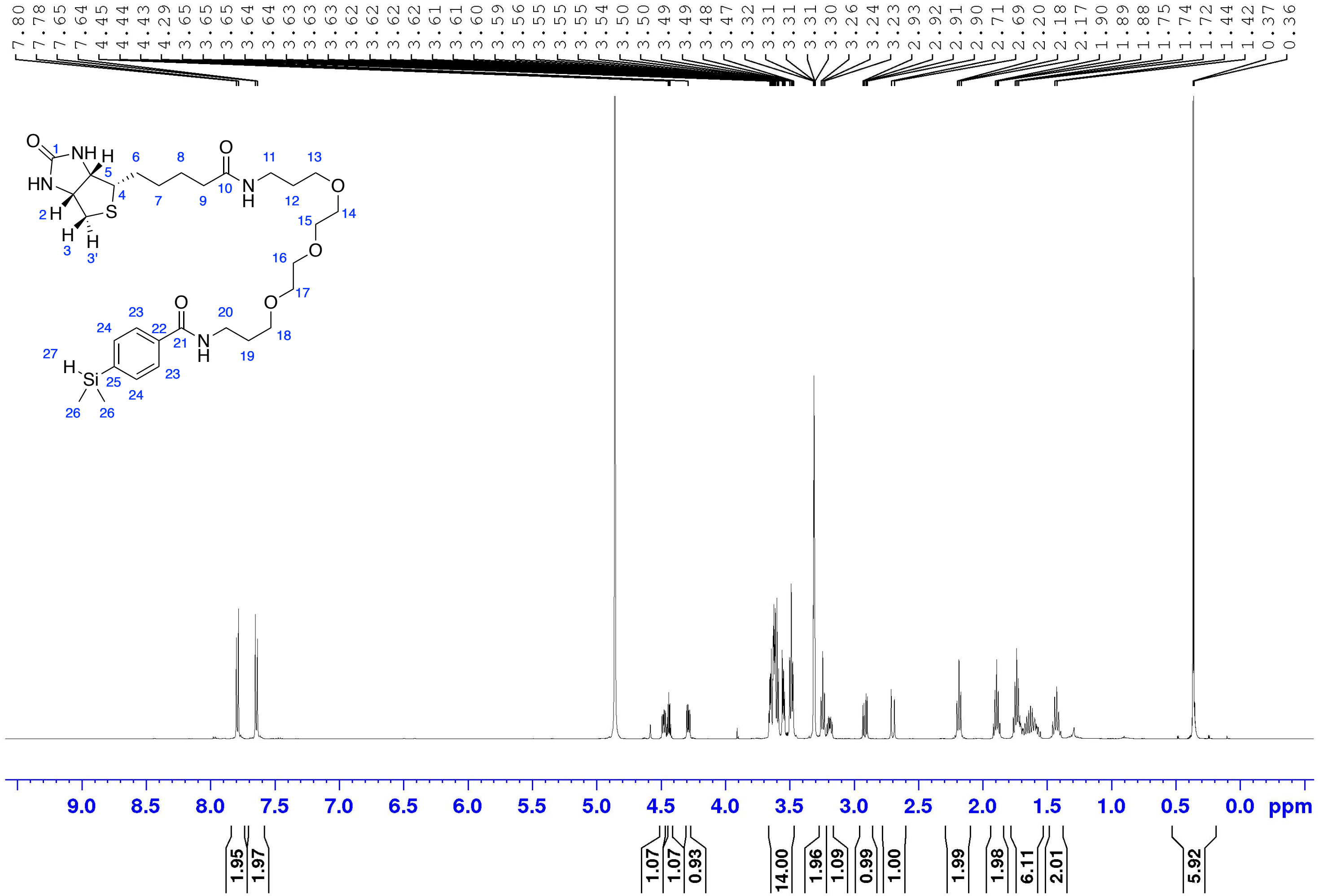

4-(Dimethylsilyl)-*N*-(15-oxo-19-((3a*S*,4*S*,6a*R*)-2-oxohexahydro-1*H*-thieno[3,4-*d*]imidazol-4-yl)-4,7,10-trioxa-14-azanonadecyl)benzamide (**29**), ^{13}C NMR ($CDCl_3$, 126 MHz)

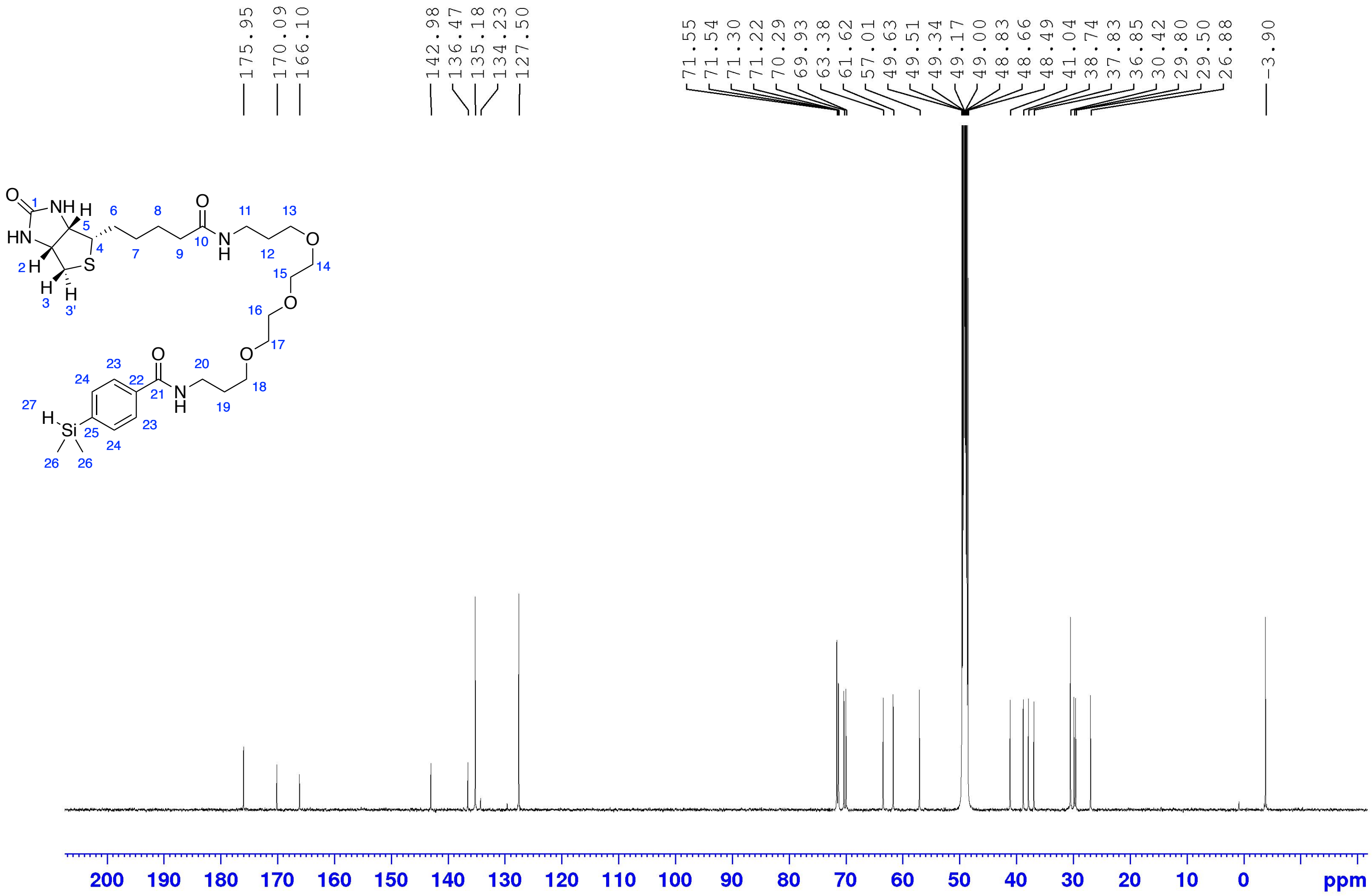

tert-Butyl (1-(3',6'-bis(dimethylamino)-3-oxo-3*H*-spiro[isobenzofuran-1,9'-xanthen]-5-yl)-1-oxo-6,9,12-trioxa-2-azapentadecan-15-yl)carbamate (**S8**), ^1H NMR (CD_3OD, 500 MHz)

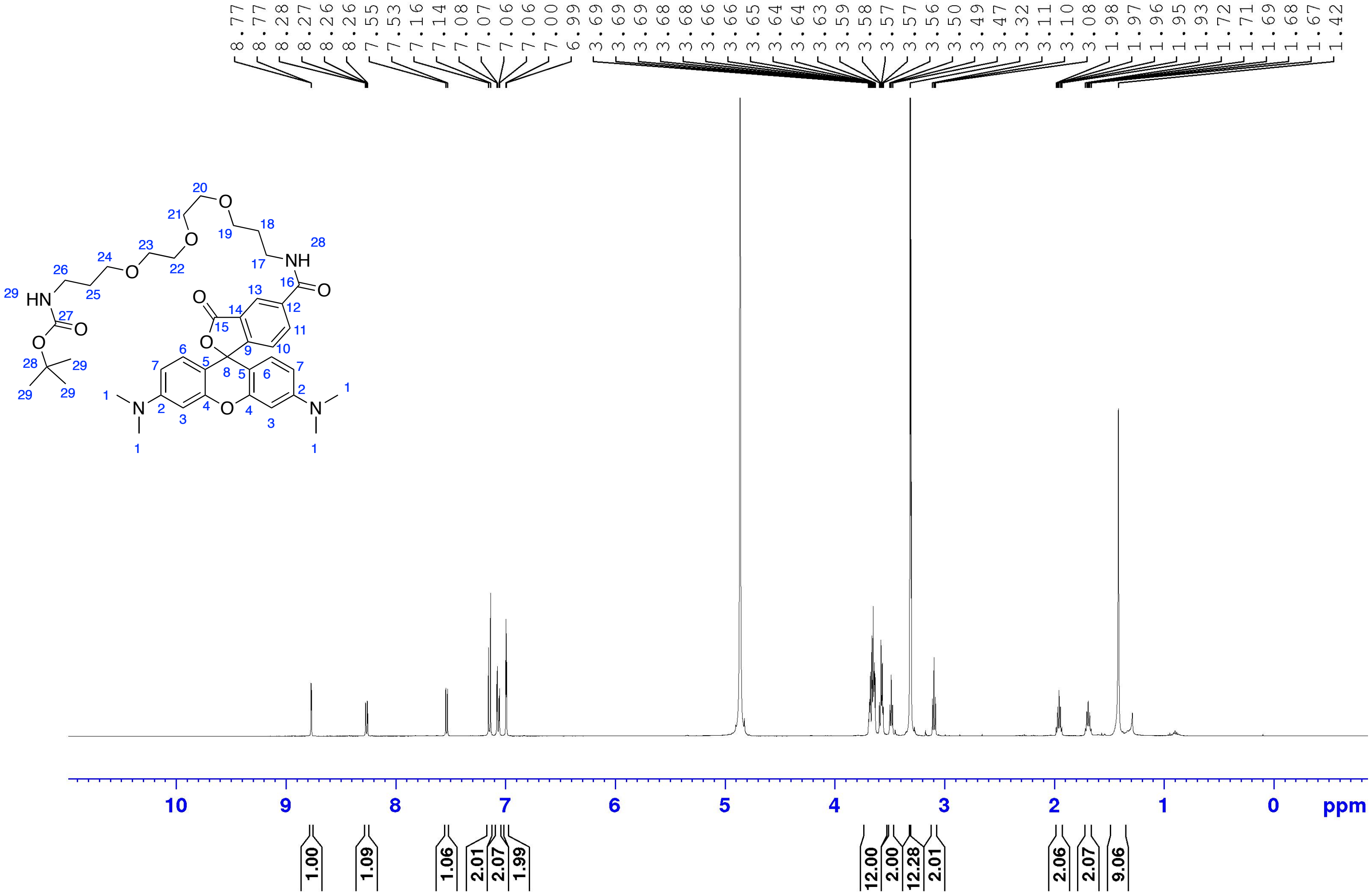

tert-Butyl (1-(3',6'-bis(dimethylamino)-3-oxo-3*H*-spiro[isobenzofuran-1,9'-xanthen]-5-yl)-1-oxo-6,9,12-trioxa-2-azapentadecan-15-yl)carbamate (**S8**), ^{13}C NMR (CD_3OD, 126 MHz)

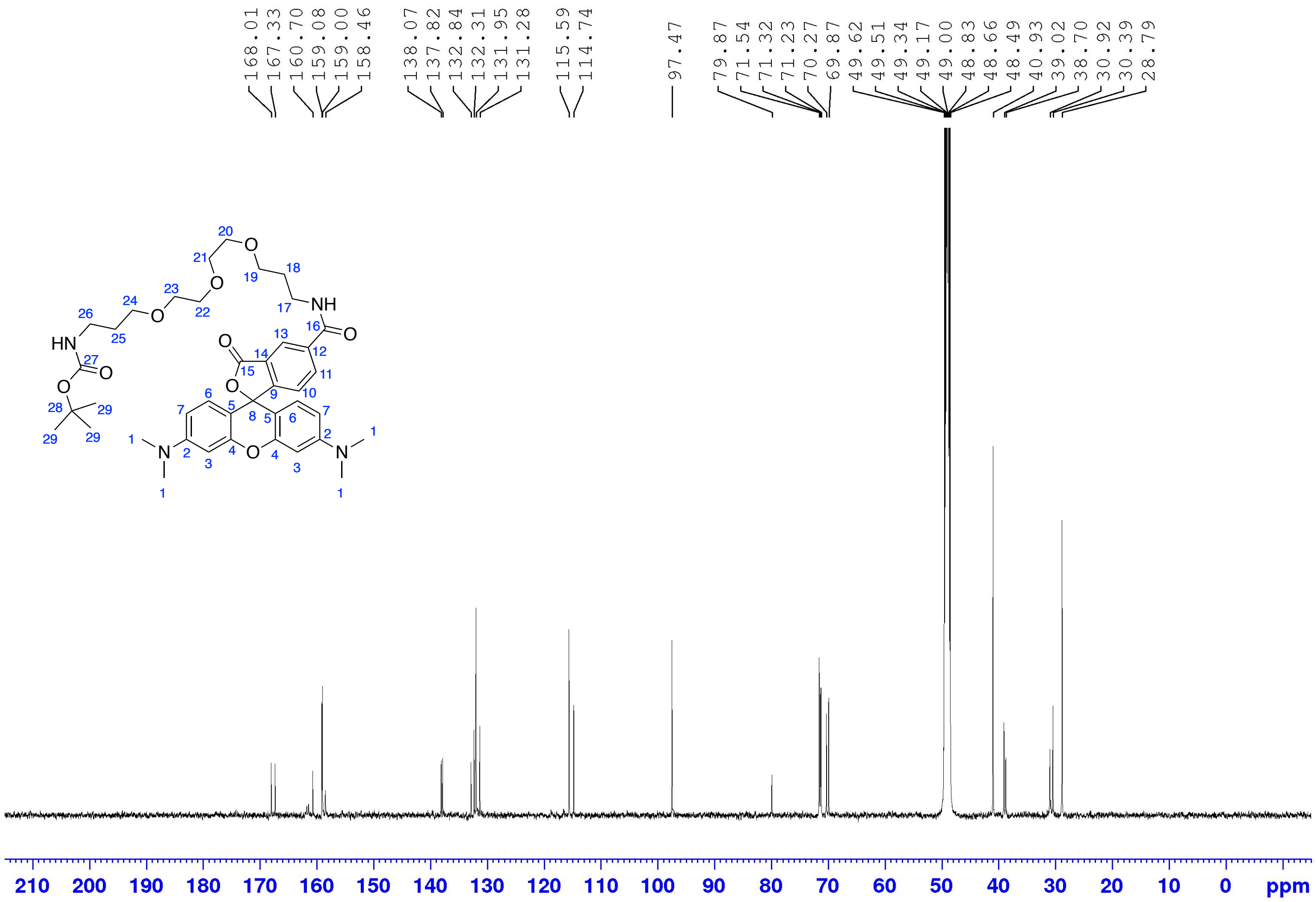

3',6'-Bis(dimethylamino)-*N*-(1-(4-(dimethylsilyl)phenyl)-1-oxo-6,9,12-trioxa-2-azapentadecan-15-yl)-3-oxo-3*H*-spiro[isobenzofuran-1,9'-xanthene]-5-carboxamide (**39**), ^{1}H NMR (DMSO-d_6, 500 MHz)

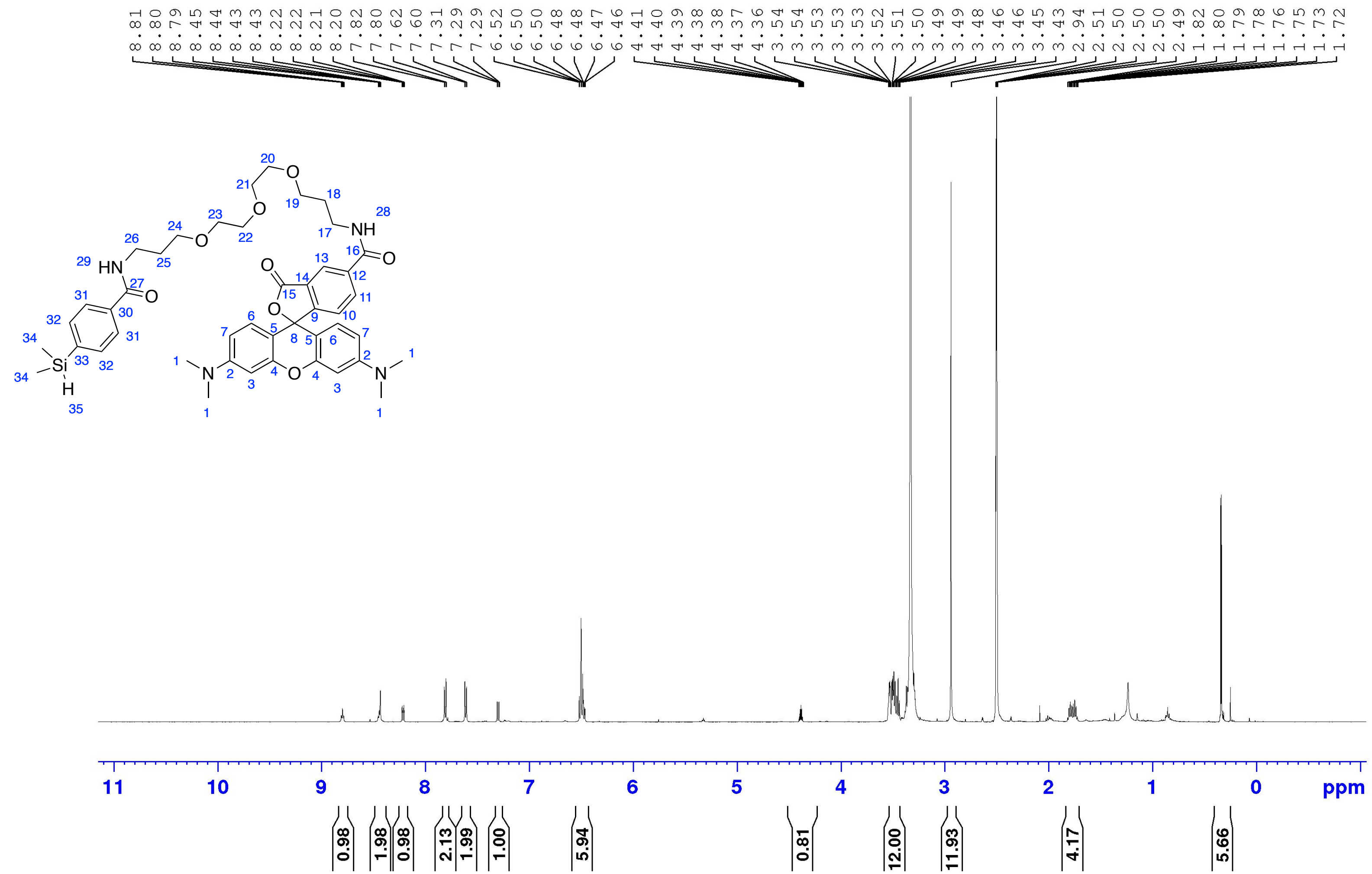

3',6'-Bis(dimethylamino)-*N*-(1-(4-(dimethylsilyl)phenyl)-1-oxo-6,9,12-trioxa-2-azapentadecan-15-yl)-3-oxo-3*H*-spiro[isobenzofuran-1,9'-xanthene]-5-carboxamide (**39**), ^{13}C NMR (DMSO-d_6, 126 MHz)

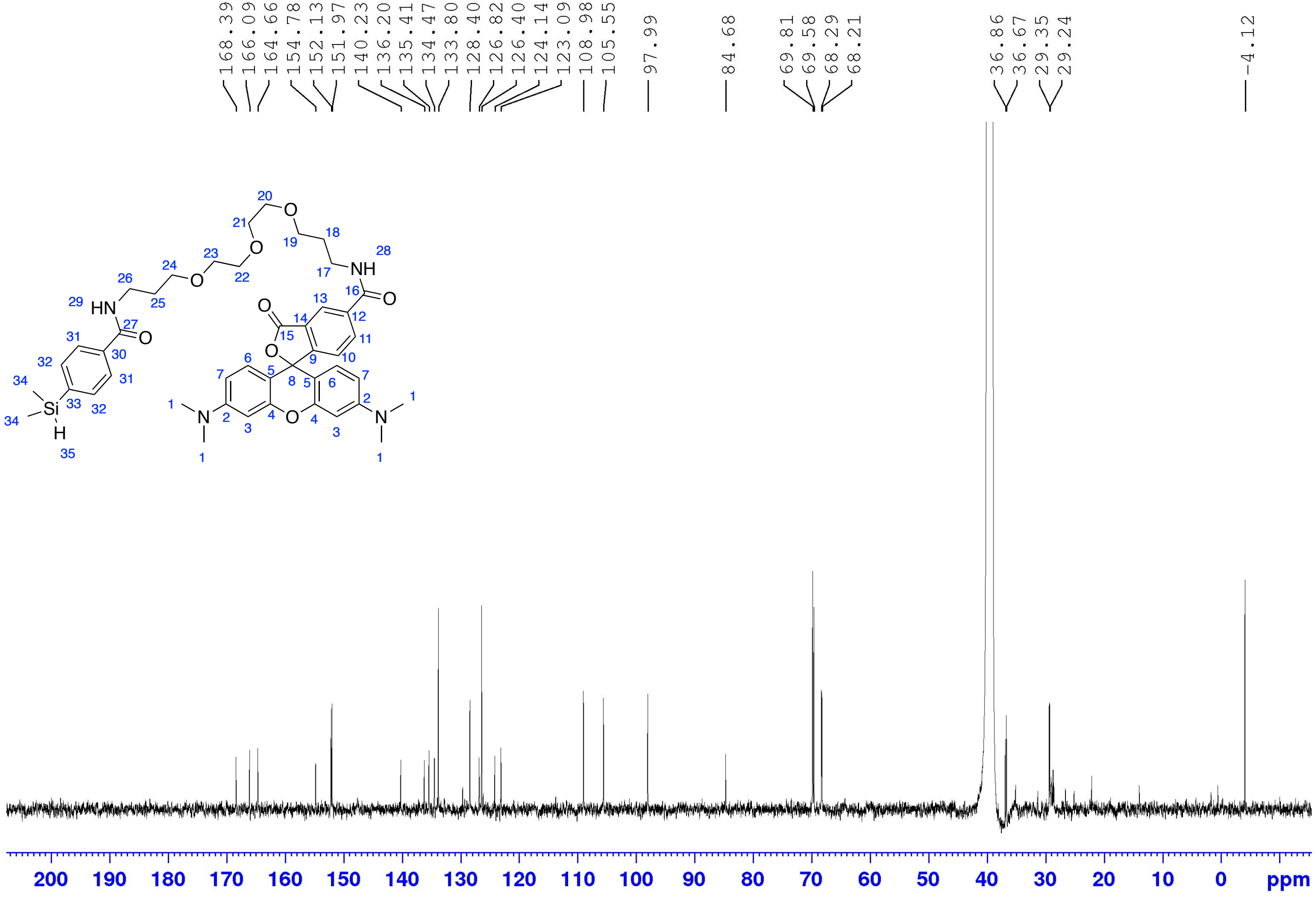

3,6,9,12-Tetraoxapentadec-14-yne (**2**), ^{1}H NMR ($CDCl_3$, 500 MHz)

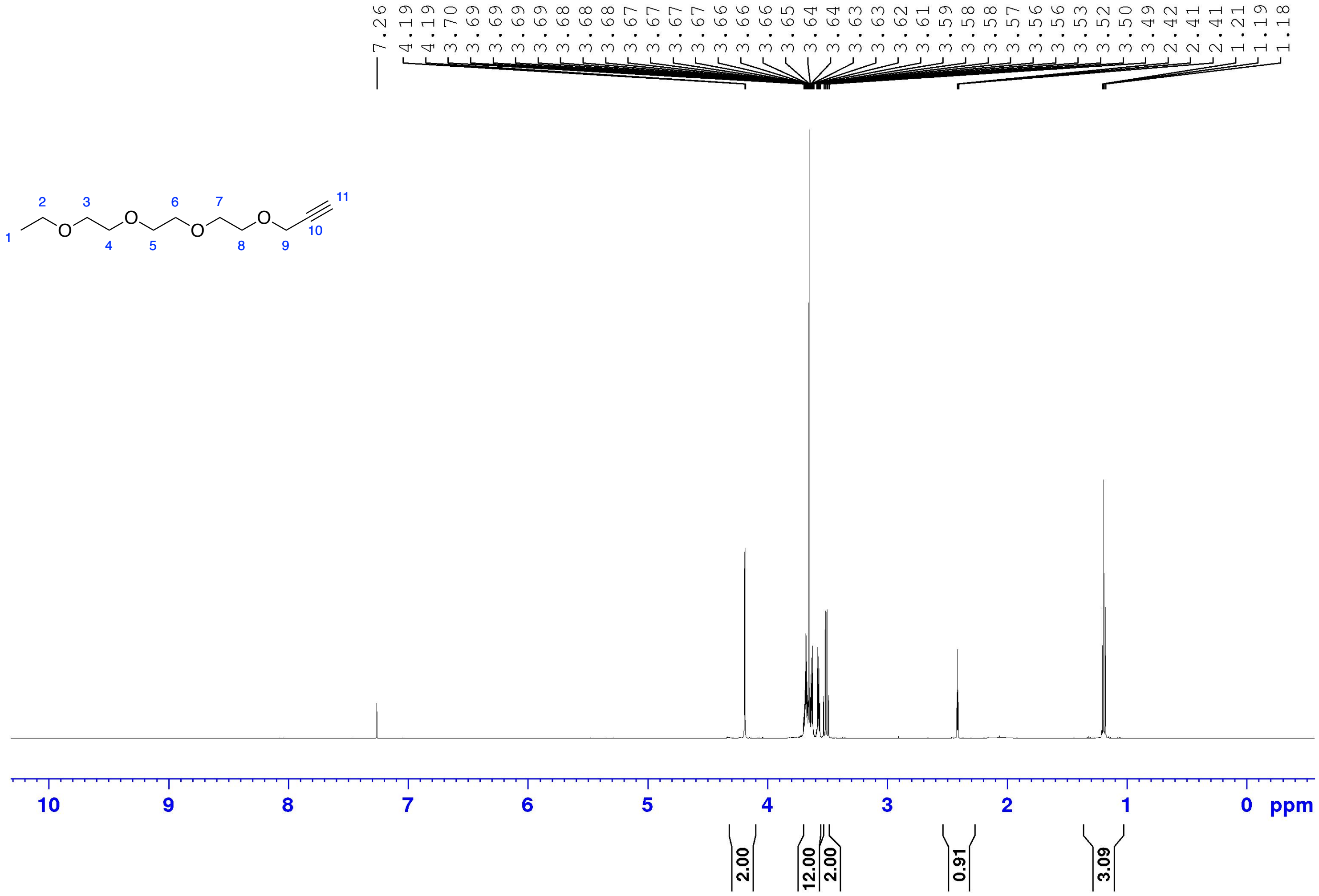

3,6,9,12-Tetraoxapentadec-14-yne (**2**), ^{13}C NMR ($CDCl_3$, 126 MHz)

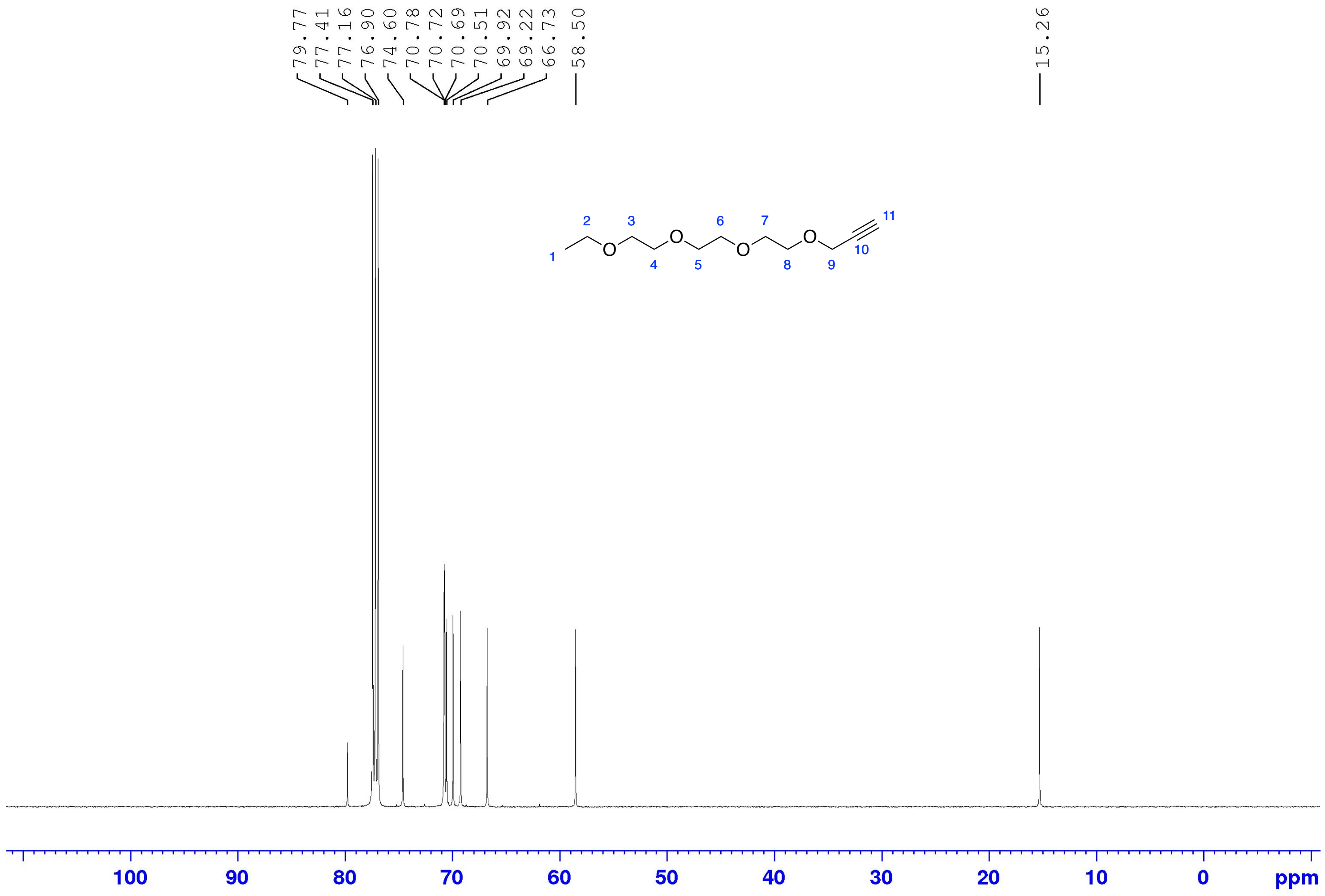

3,4-Dimethoxy-*N*-(pent-4-yn-1-yl)benzamide (**9**), ^{1}H NMR ($CDCl_3$, 500 MHz)

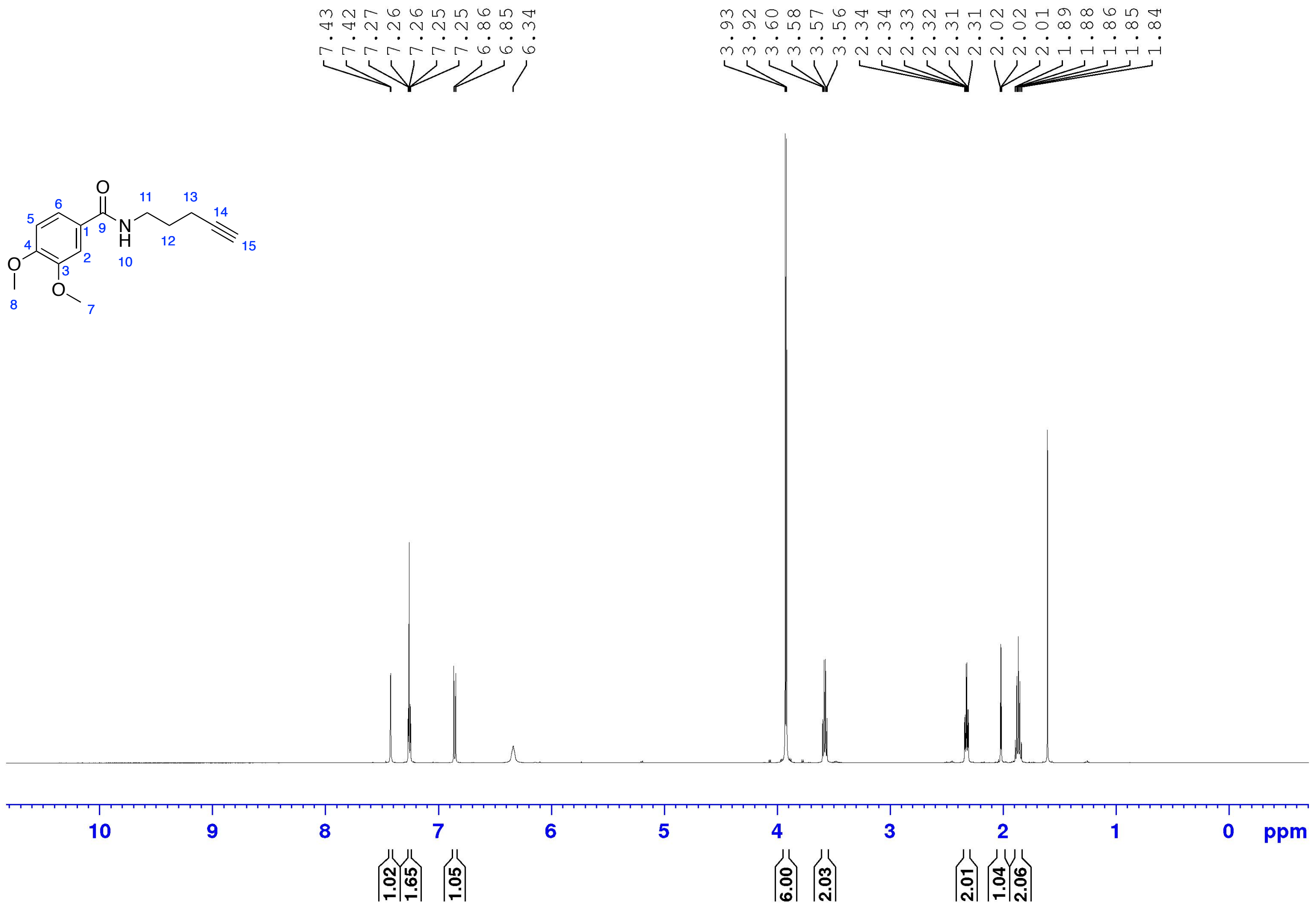

3,4-Dimethoxy-*N*-(pent-4-yn-1-yl)benzamide (**9**), ^{13}C NMR ($CDCl_3$, 126 MHz)

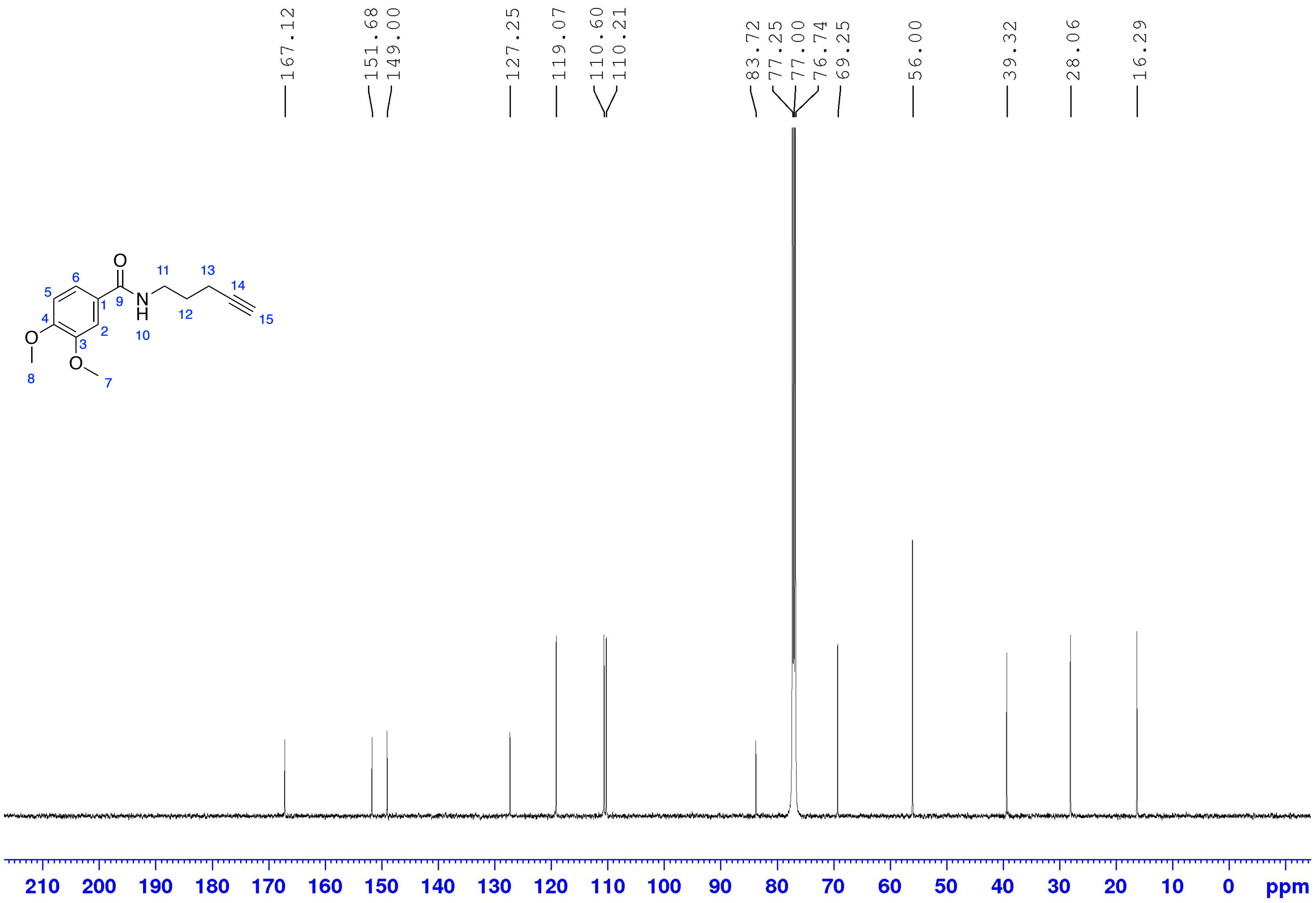

N-(2-(2-(2-Hydroxyethoxy)ethoxy)ethyl)-3,4-dimethoxybenzamide (**S9**), ^{1}H NMR ($CDCl_3$, 500 MHz)

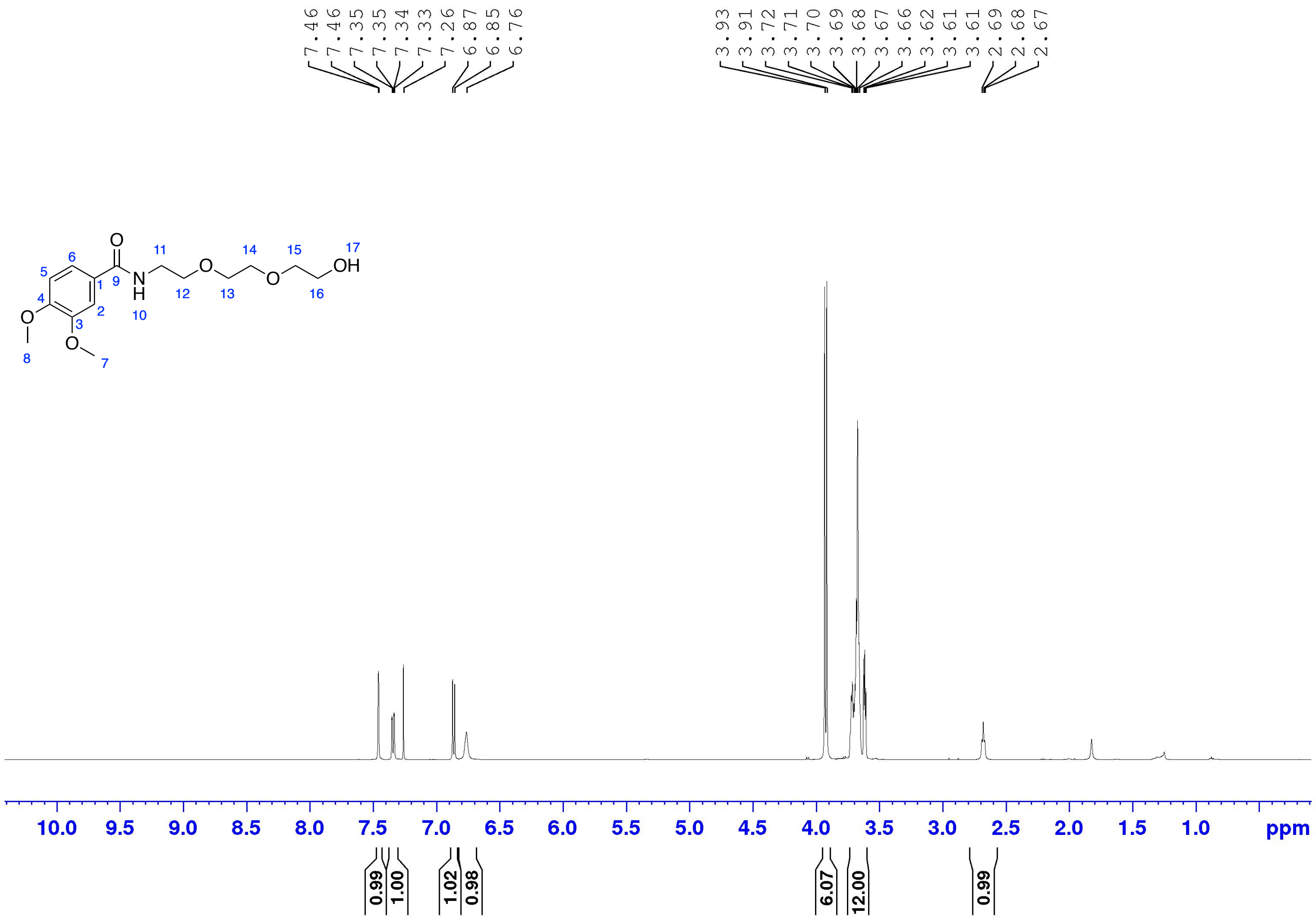

N-(2-(2-(2-Hydroxyethoxy)ethoxy)ethyl)-3,4-dimethoxybenzamide (**S9**), ^{13}C NMR ($CDCl_3$, 126 MHz)

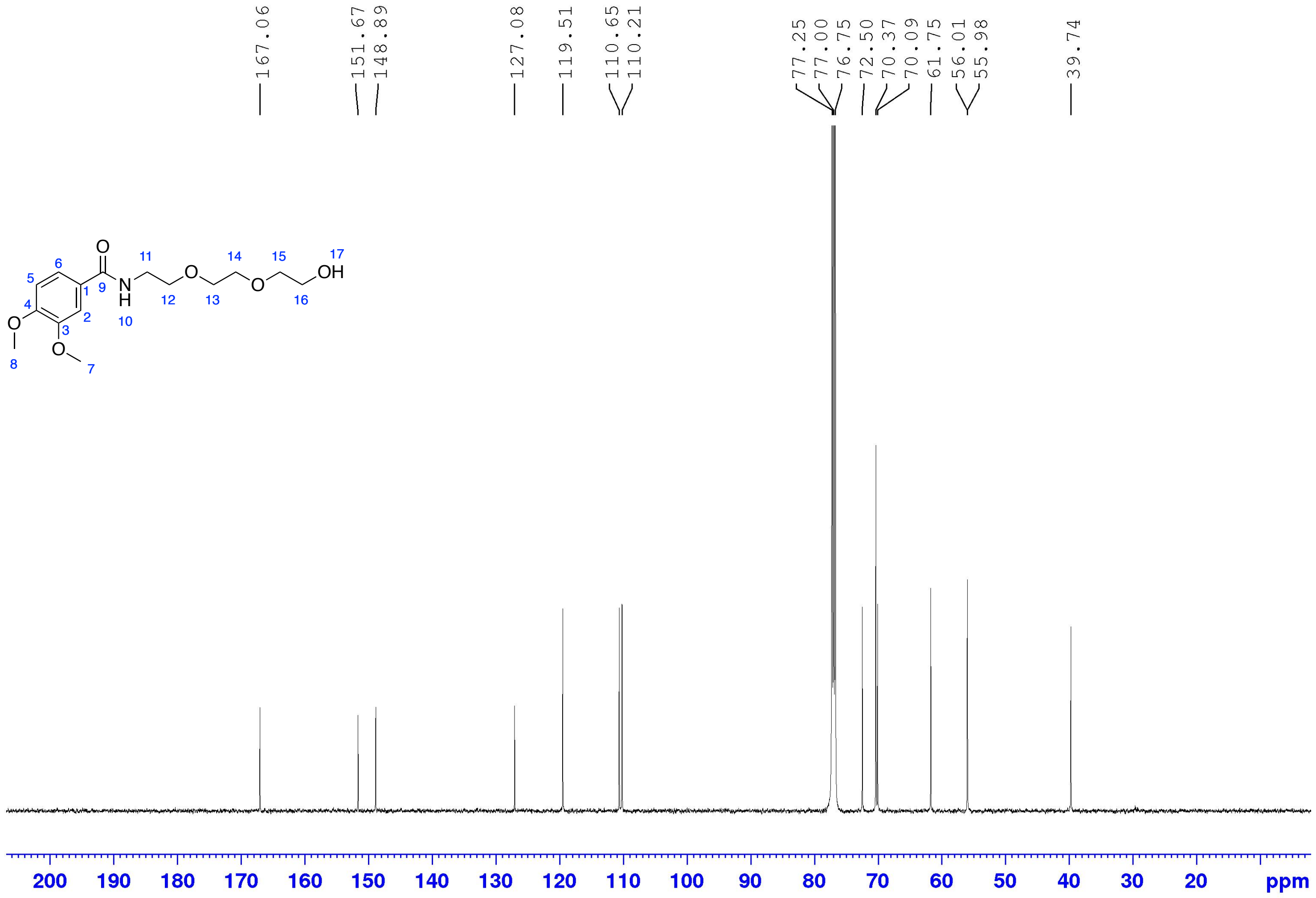

2-(2-(2-(3,4-Dimethoxybenzamido)ethoxy)ethoxy)ethyl 4-methylbenzene-sulfonate (**S10**), ^{1}H NMR ($CDCl_3$, 500 MHz)

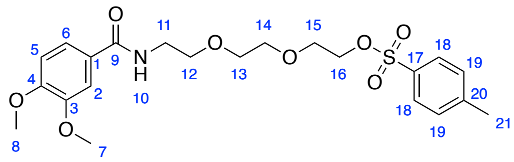

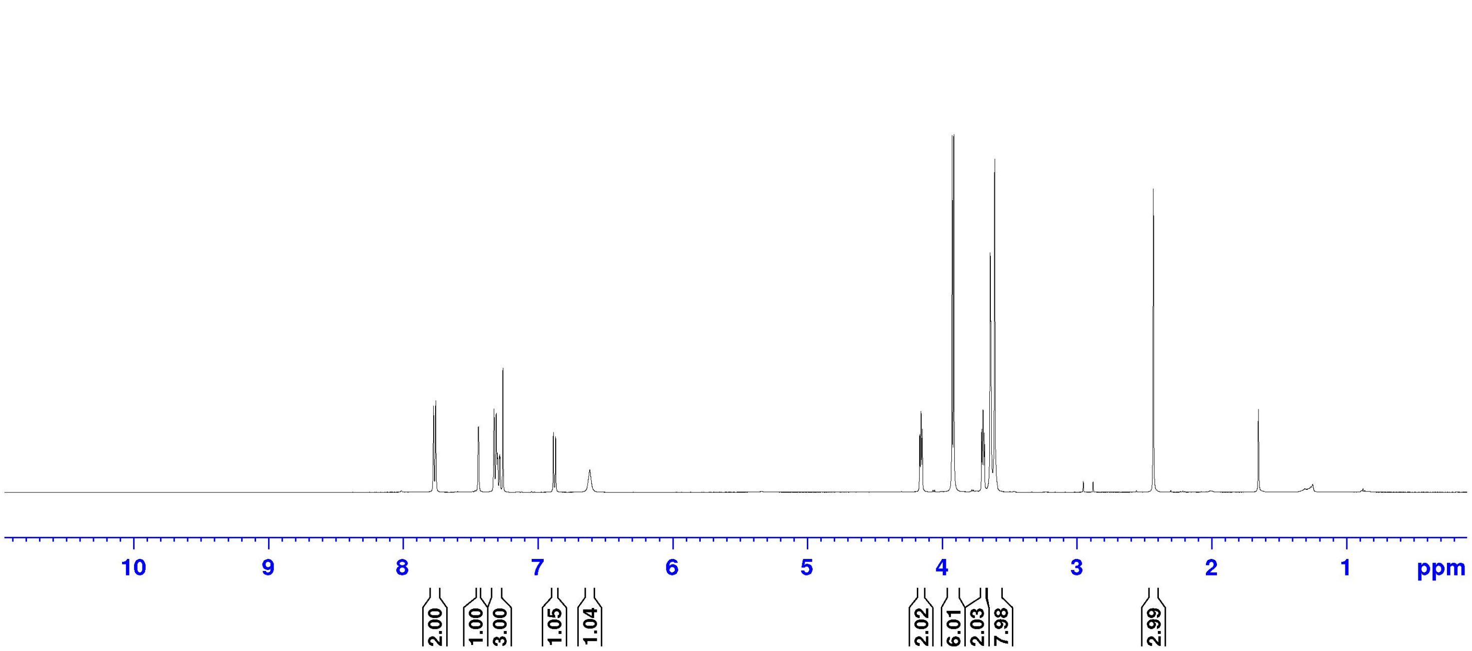

2-(2-(2-(3,4-Dimethoxybenzamido)ethoxy)ethoxy)ethyl 4-methylbenzene-sulfonate (**S10**), ^{13}C NMR ($CDCl_3$, 126 MHz)

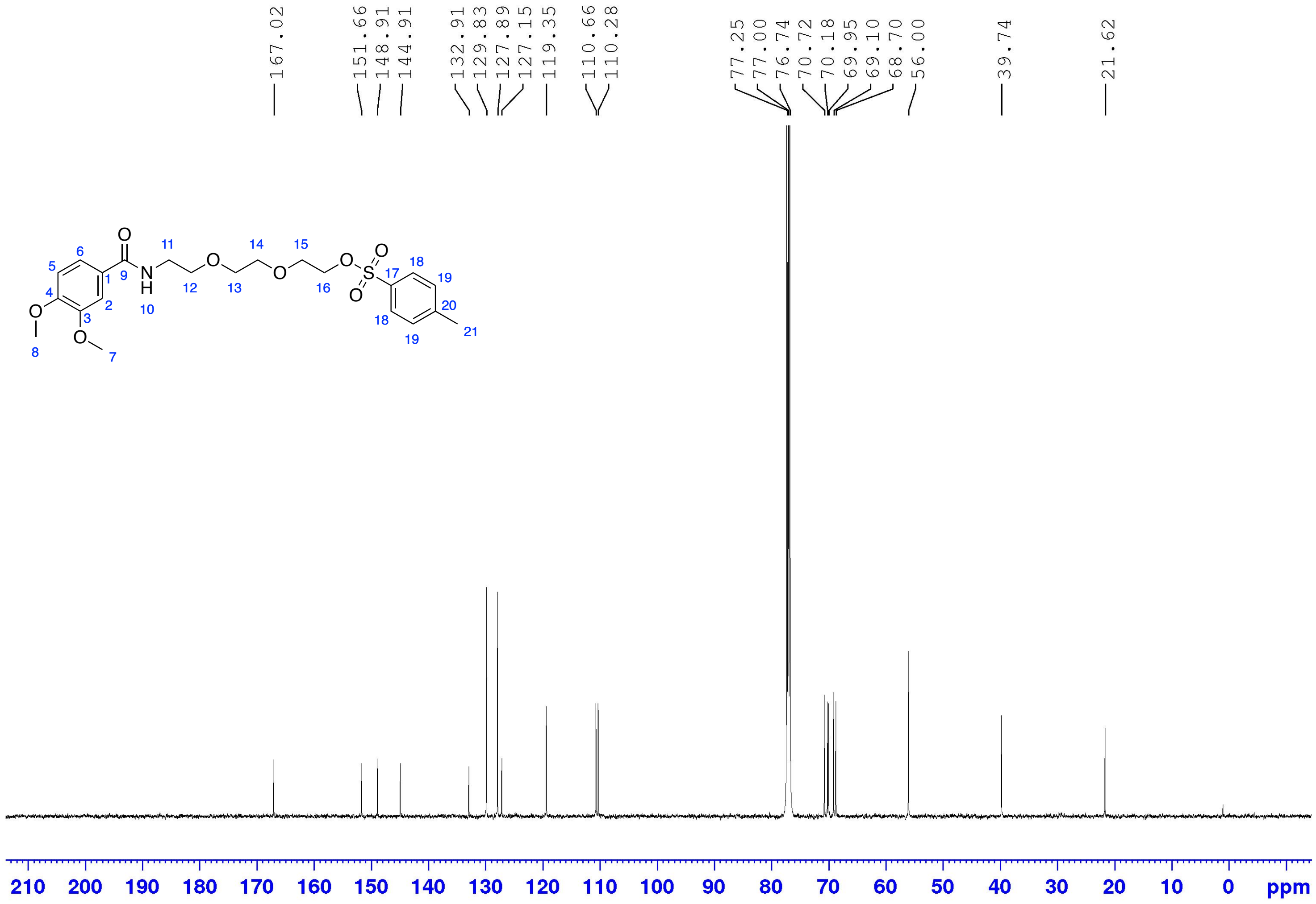

S-(2-(2-(2-(3,4-Dimethoxybenzamido)ethoxy)ethoxy)ethyl) ethanethioate (**S11**), ^{1}H NMR (CD_3OD, 500 MHz)

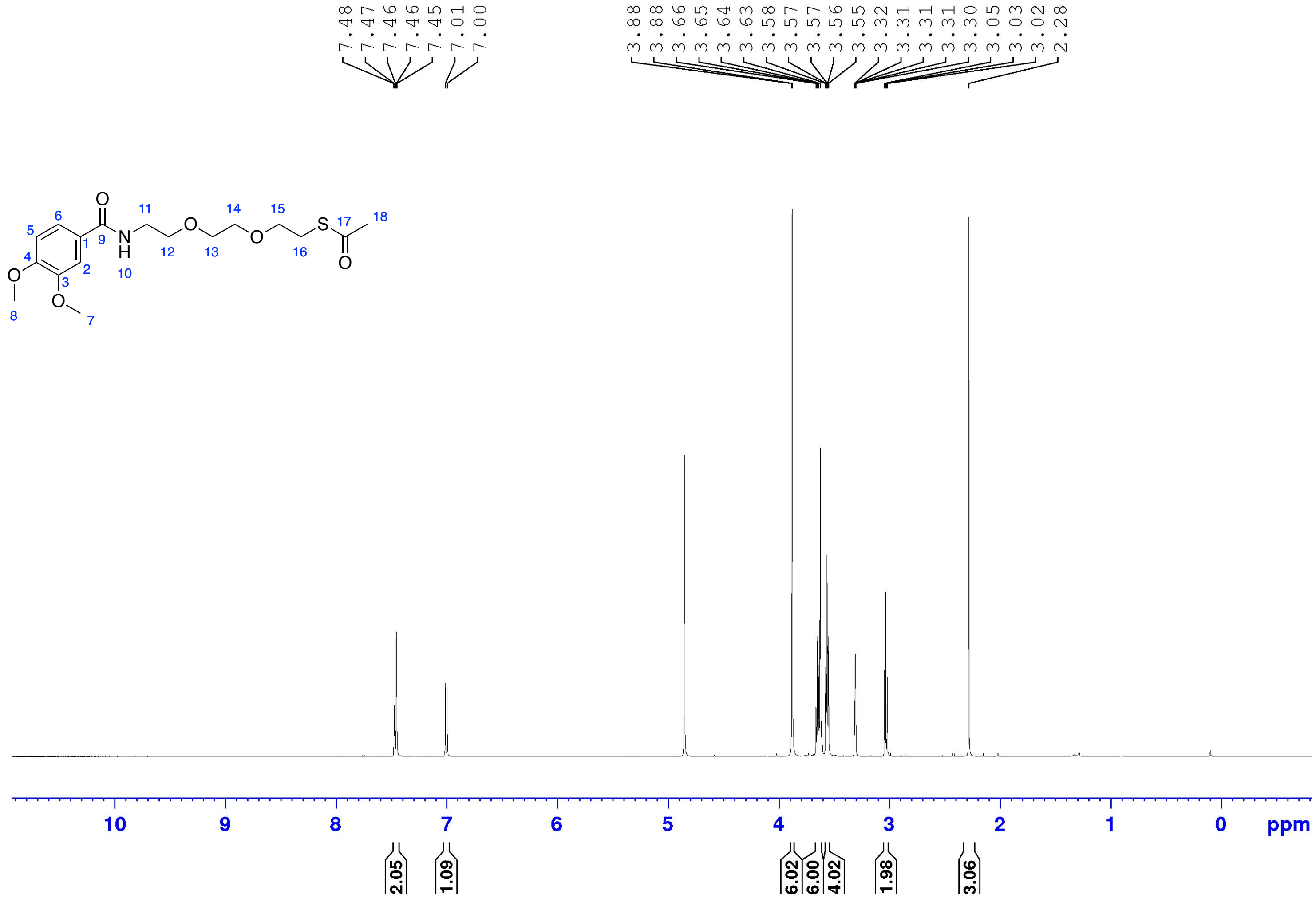

S-(2-(2-(2-(3,4-Dimethoxybenzamido)ethoxy)ethoxy)ethyl) ethanethioate (**S11**), ^{13}C NMR (CD_3OD, 126 MHz)

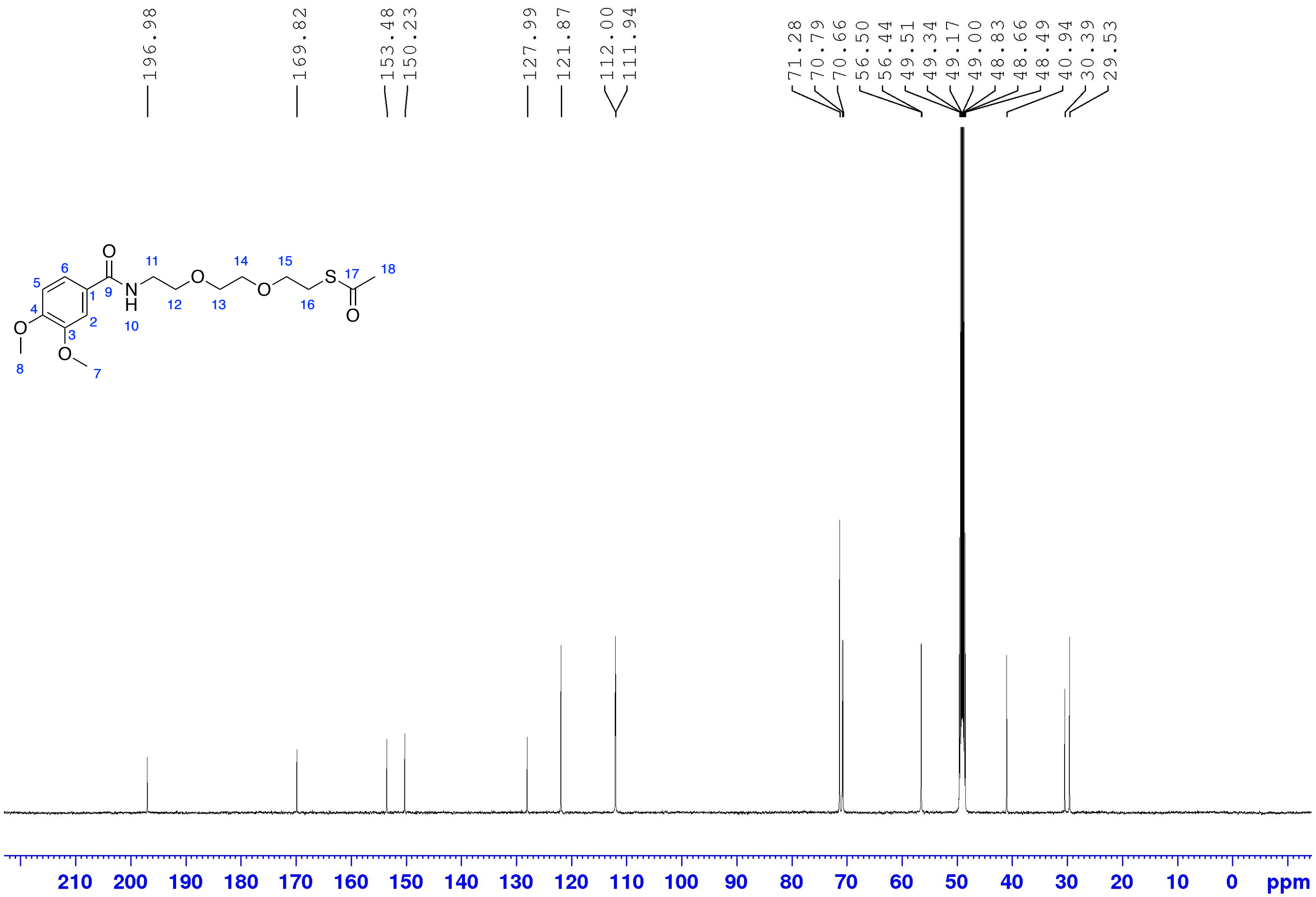

3,4-Dimethoxy-*N*-(2-(2-(2-(prop-2-yn-1-ylthio)ethoxy)ethoxy)ethyl)-benzamide (**11**), ^{1}H NMR ($CDCl_3$, 500 MHz)

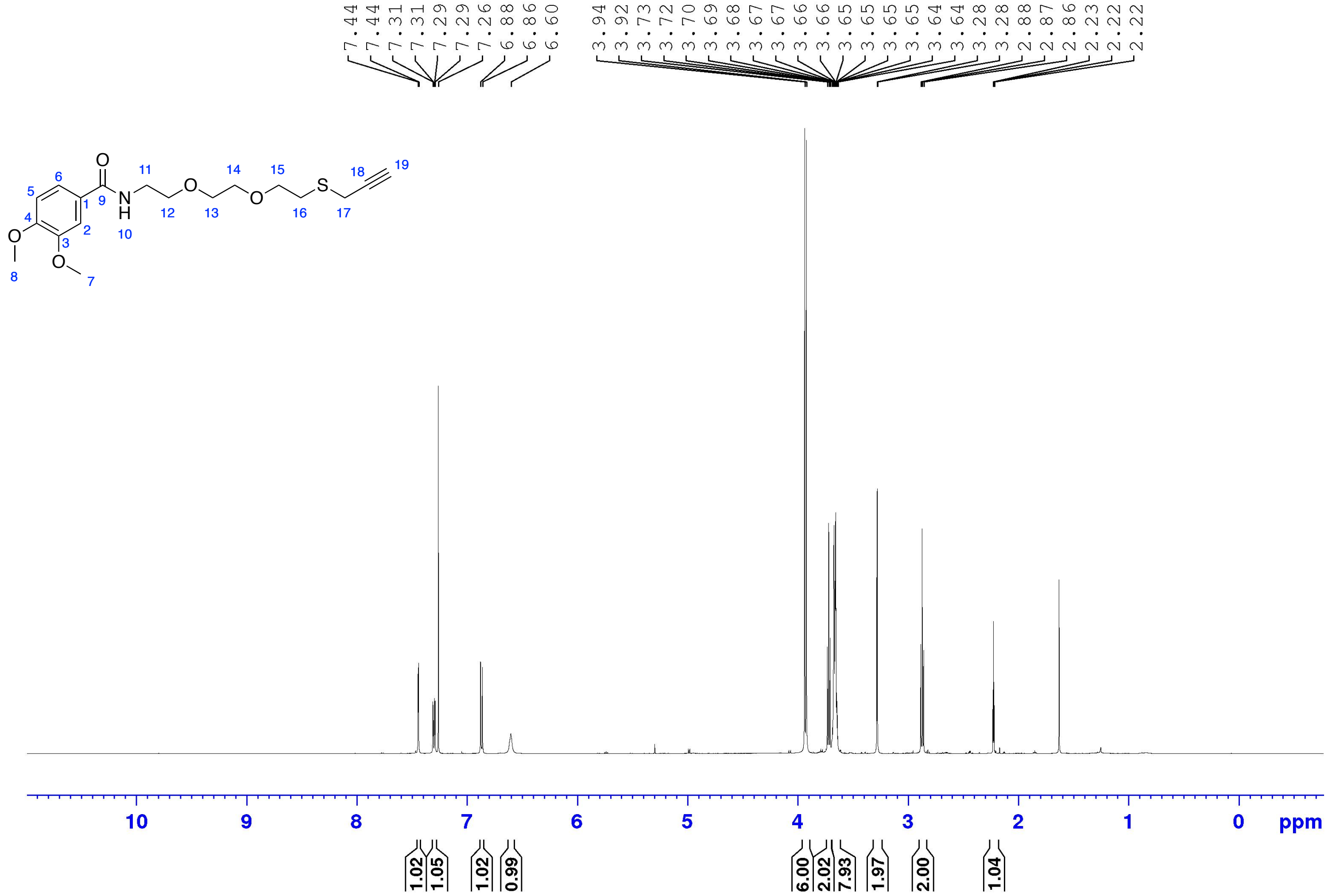

3,4-Dimethoxy-*N*-(2-(2-(2-(prop-2-yn-1-ylthio)ethoxy)ethoxy)ethyl)-benzamide (**11**), ^{13}C NMR ($CDCl_3$, 126 MHz)

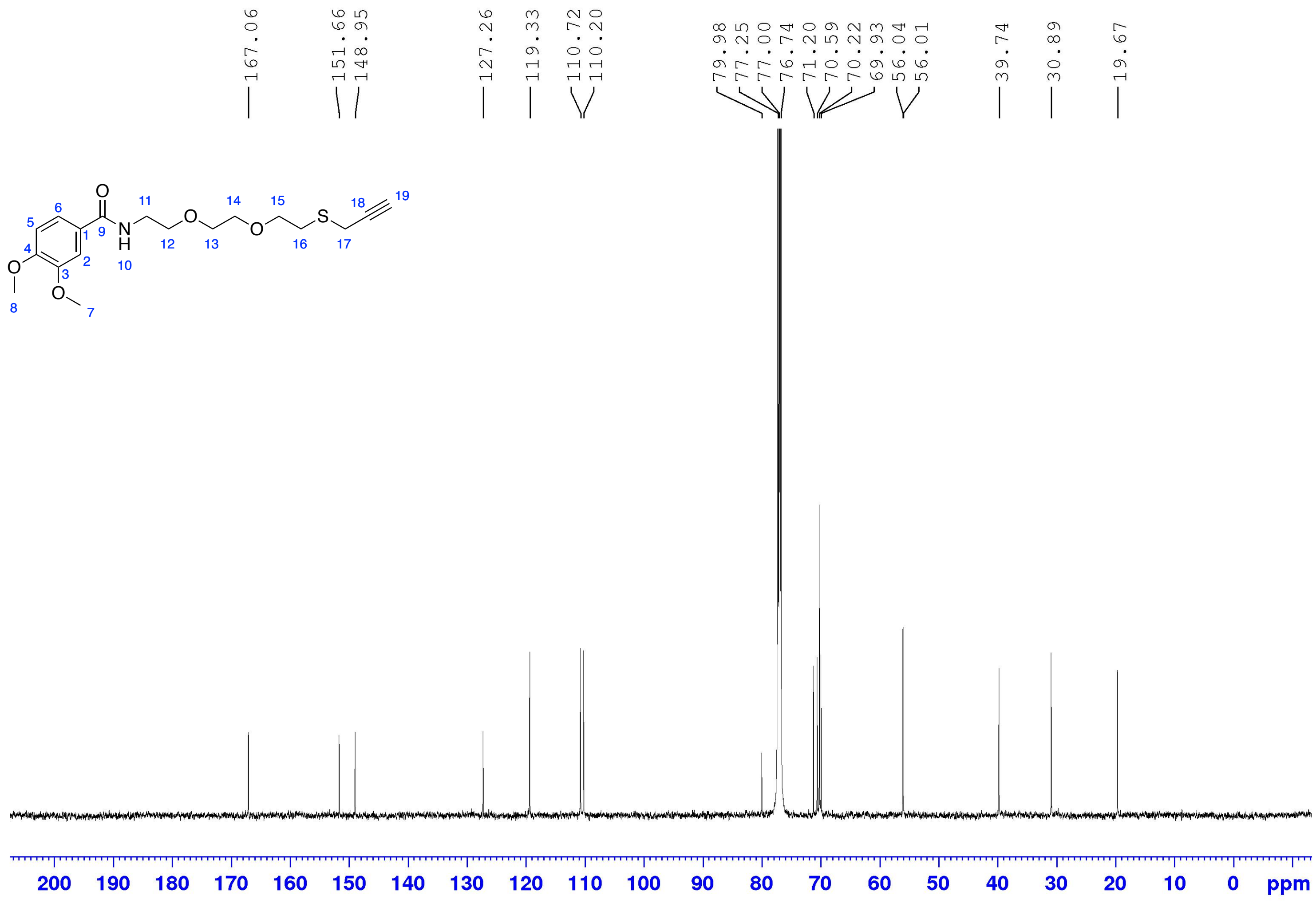

3,4-Dimethoxy-*N*-(2-(2-(2-selenocyanatoethoxy)ethoxy)ethyl)benzamide (**S12**), ^{1}H NMR ($CDCl_3$, 500 MHz)

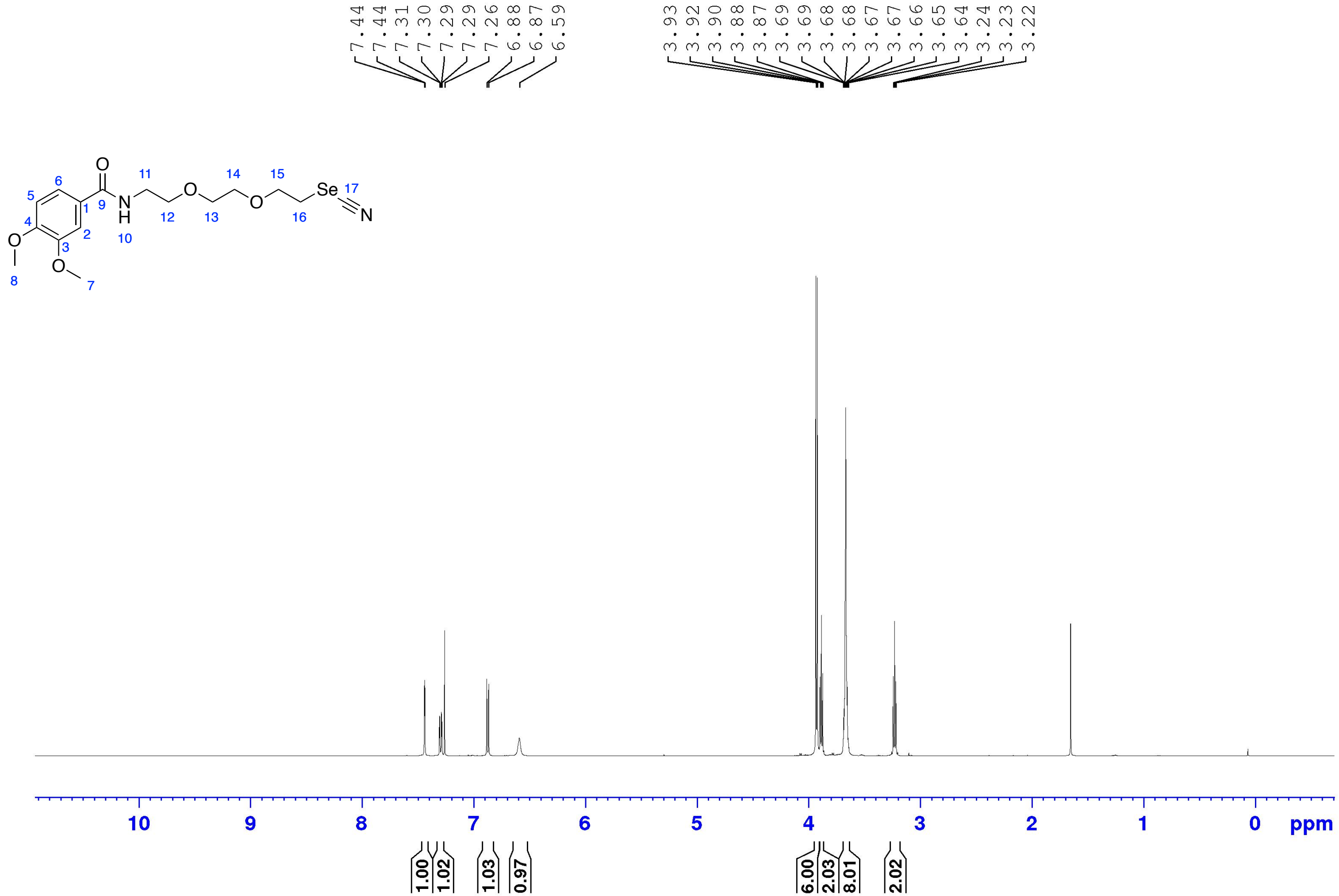

3,4-Dimethoxy-*N*-(2-(2-(2-selenocyanatoethoxy)ethoxy)ethyl)benzamide (**S12**), ^{13}C NMR ($CDCl_3$, 126 MHz)

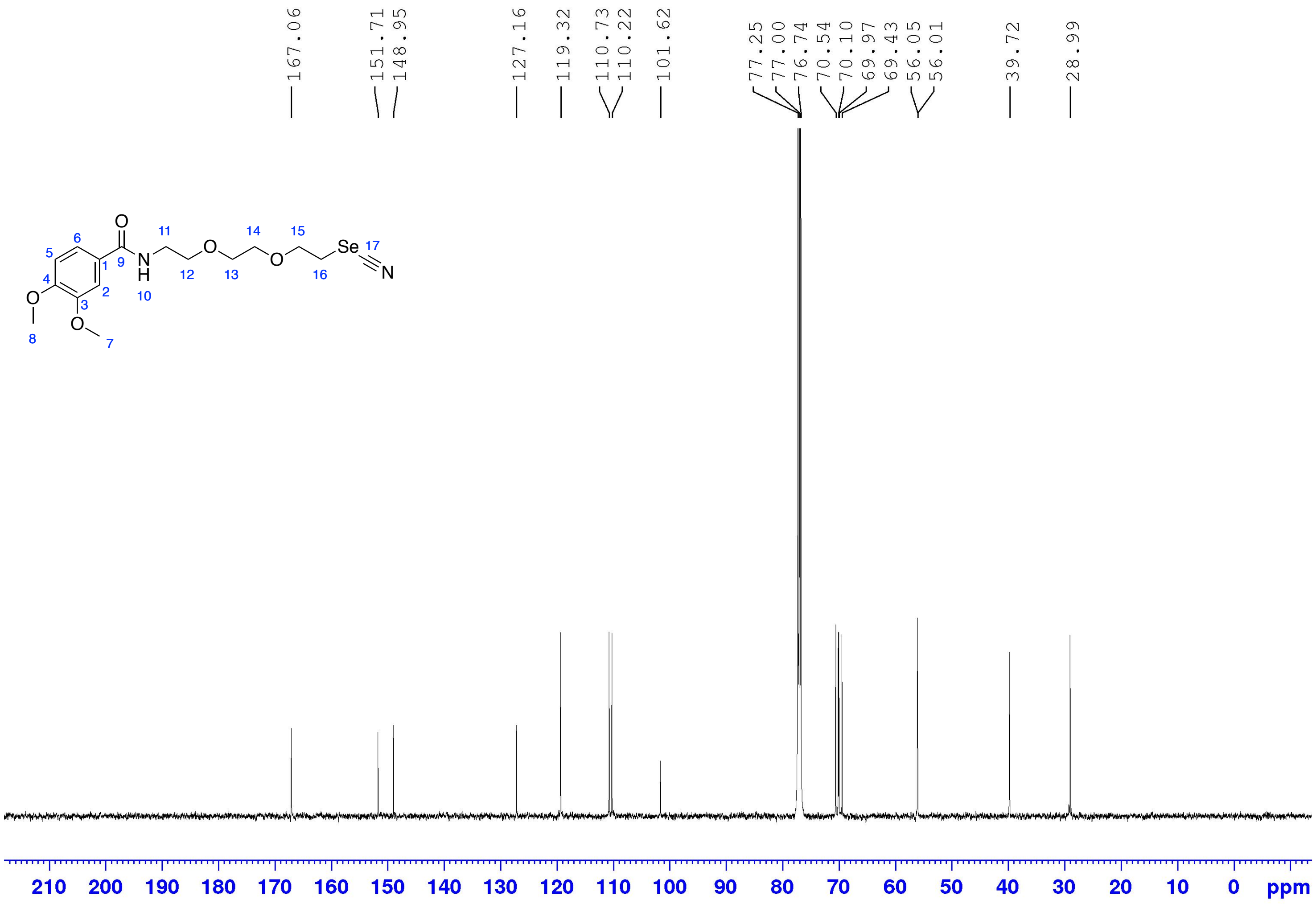

3,4-Dimethoxy-*N*-(2-(2-(2-(prop-2-yn-1-ylselanyl)ethoxy)ethoxy)ethyl)-benzamide (**12**), ^{1}H NMR ($CDCl_3$, 500 MHz)

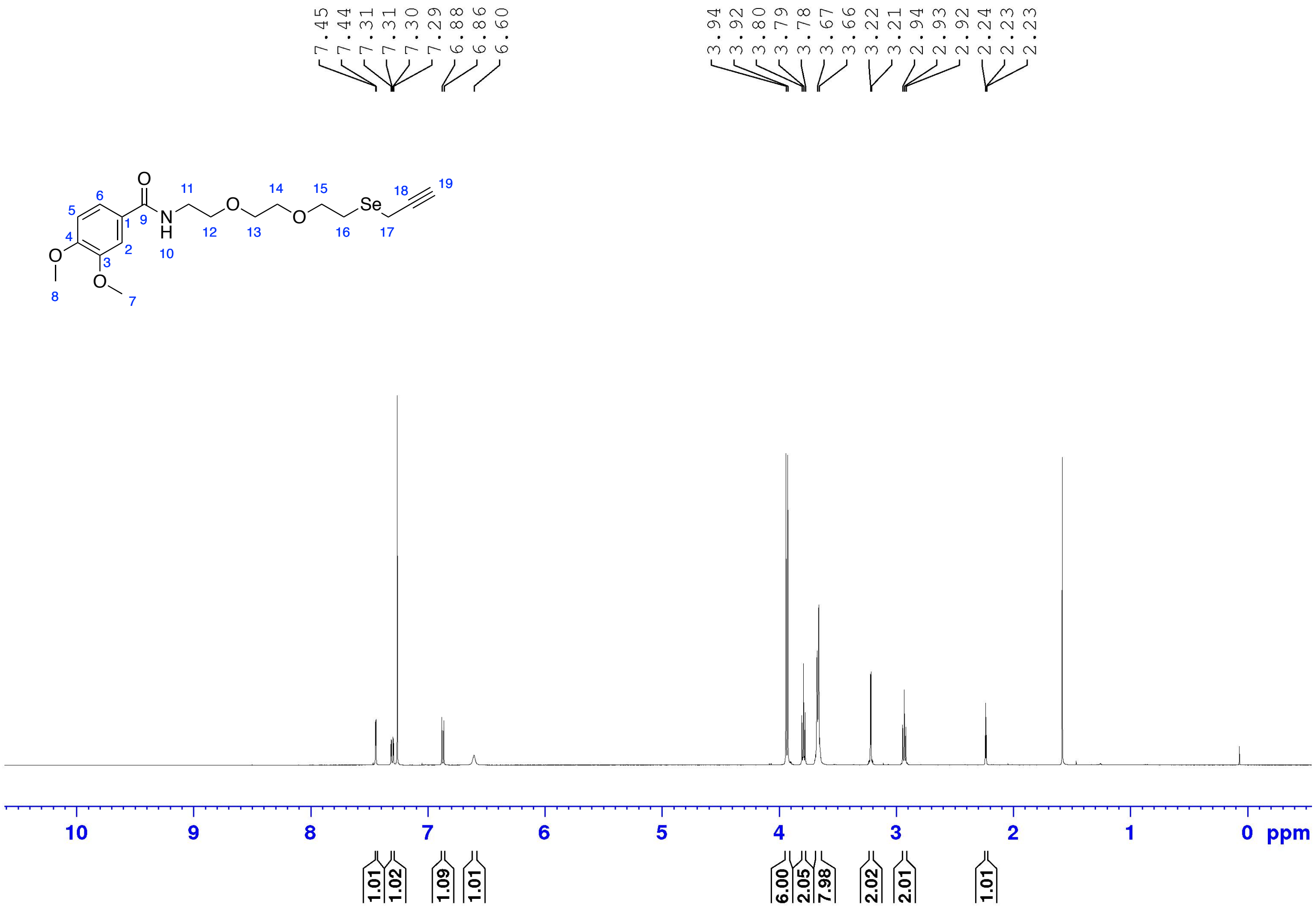

3,4-Dimethoxy-*N*-(2-(2-(2-(prop-2-yn-1-ylselanyl)ethoxy)ethoxy)ethyl)-benzamide (**12**), ^{13}C NMR ($CDCl_3$, 126 MHz)

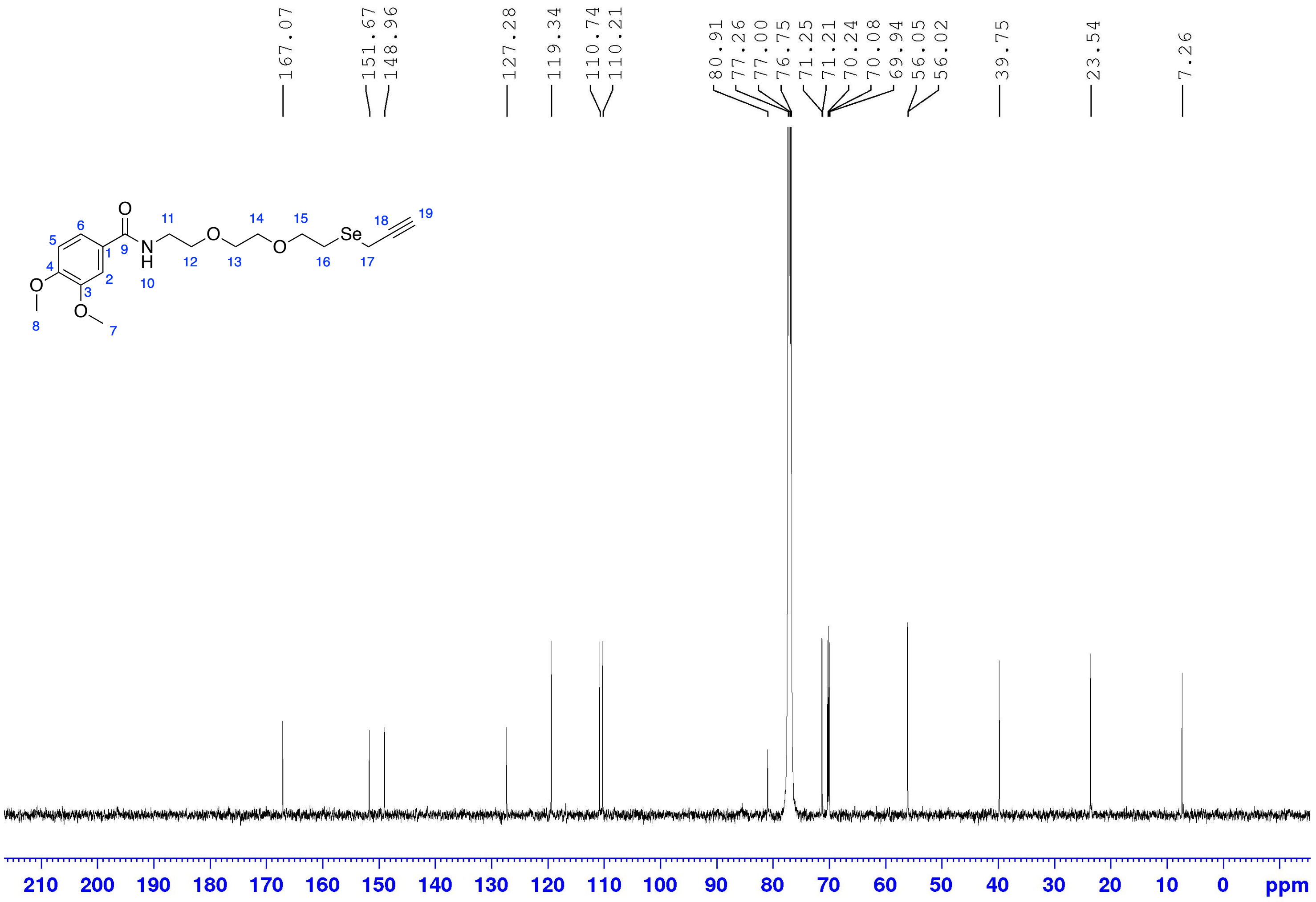

3,4-Dimethoxy-*N*-(2-(2-(2-(prop-2-yn-1-yloxy)ethoxy)ethoxy)ethyl)benzamide (**13**), ^{1}H NMR ($CDCl_3$, 500 MHz)

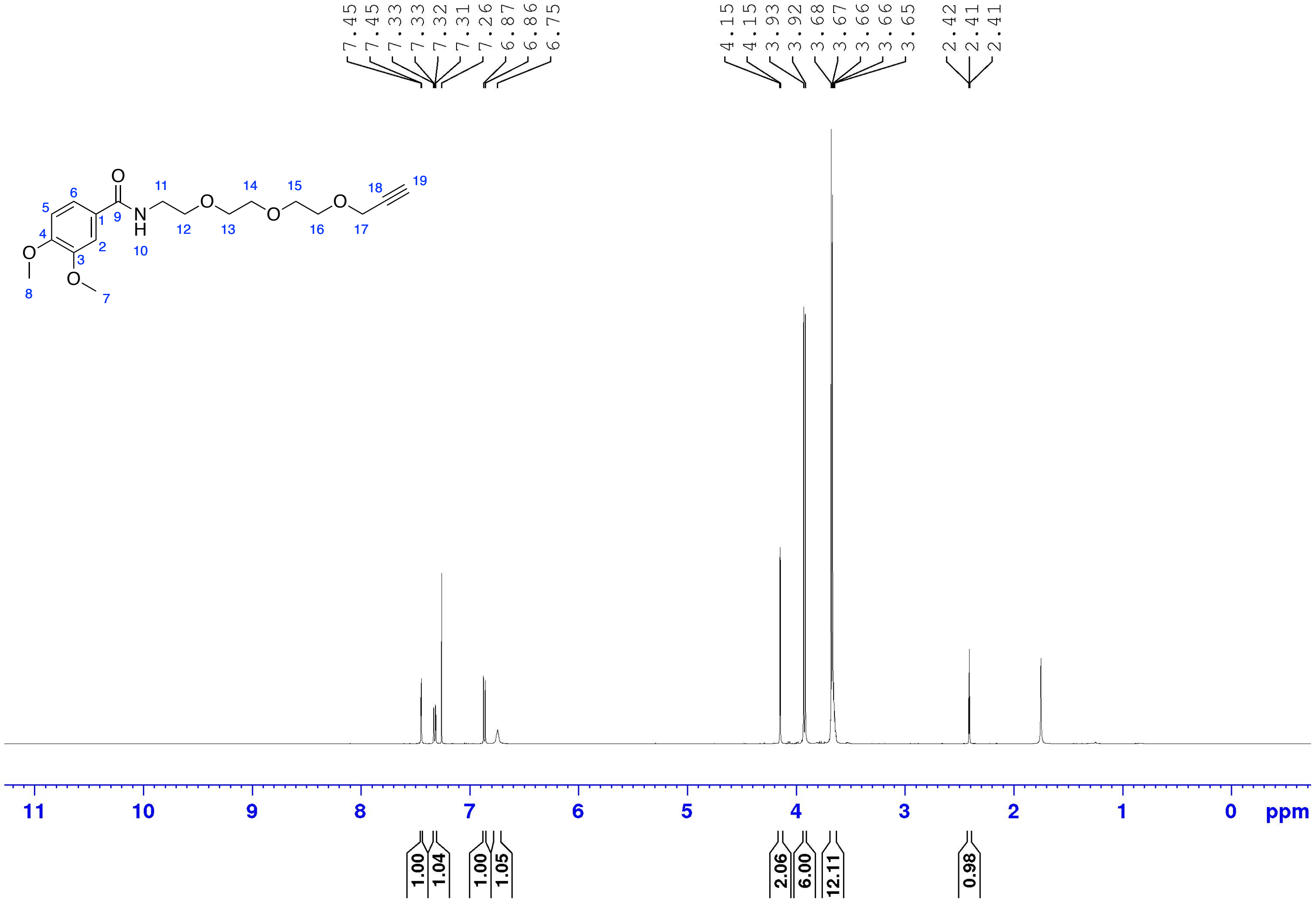

3,4-Dimethoxy-*N*-(2-(2-(2-(prop-2-yn-1-yloxy)ethoxy)ethoxy)ethyl)benzamide (**13**), ^{13}C NMR ($CDCl_3$, 126 MHz)

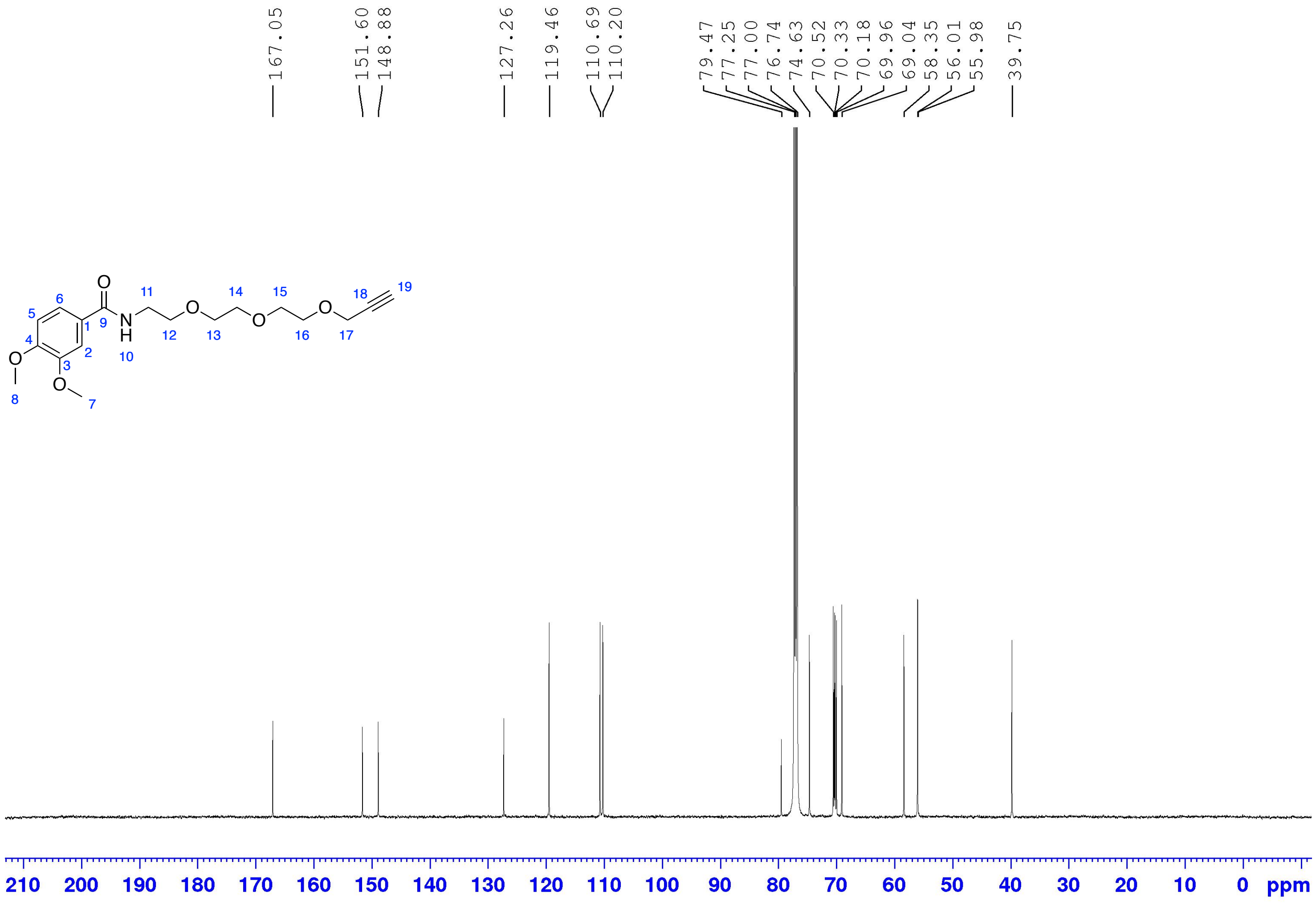

3-Selenocyanatoprop-1-yne (**S13**), ^{1}H NMR ($CDCl_3$, 500 MHz)

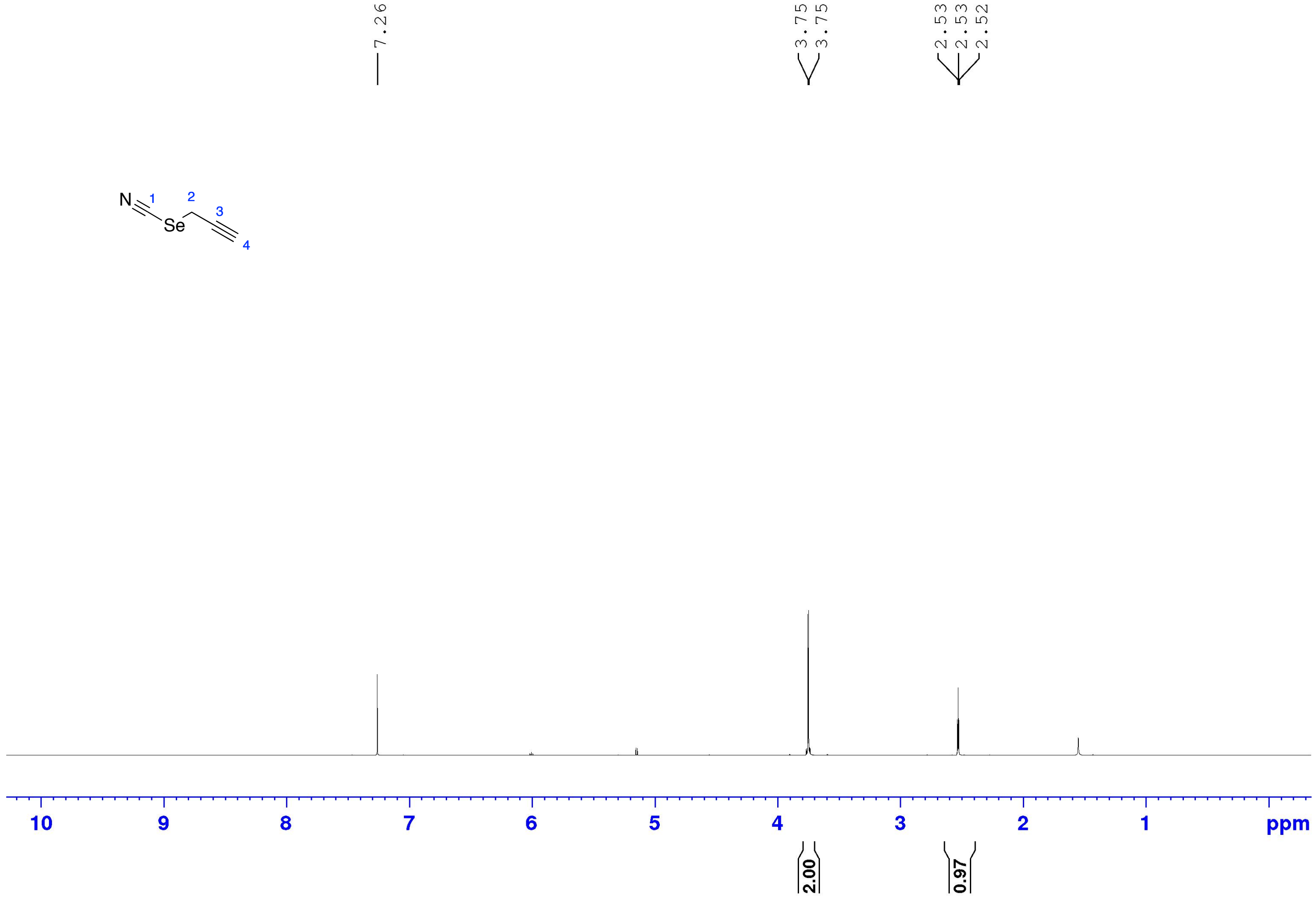

3-Selenocyanatoprop-1-yne (**S13**), ^{13}C NMR ($CDCl_3$, 126 MHz)

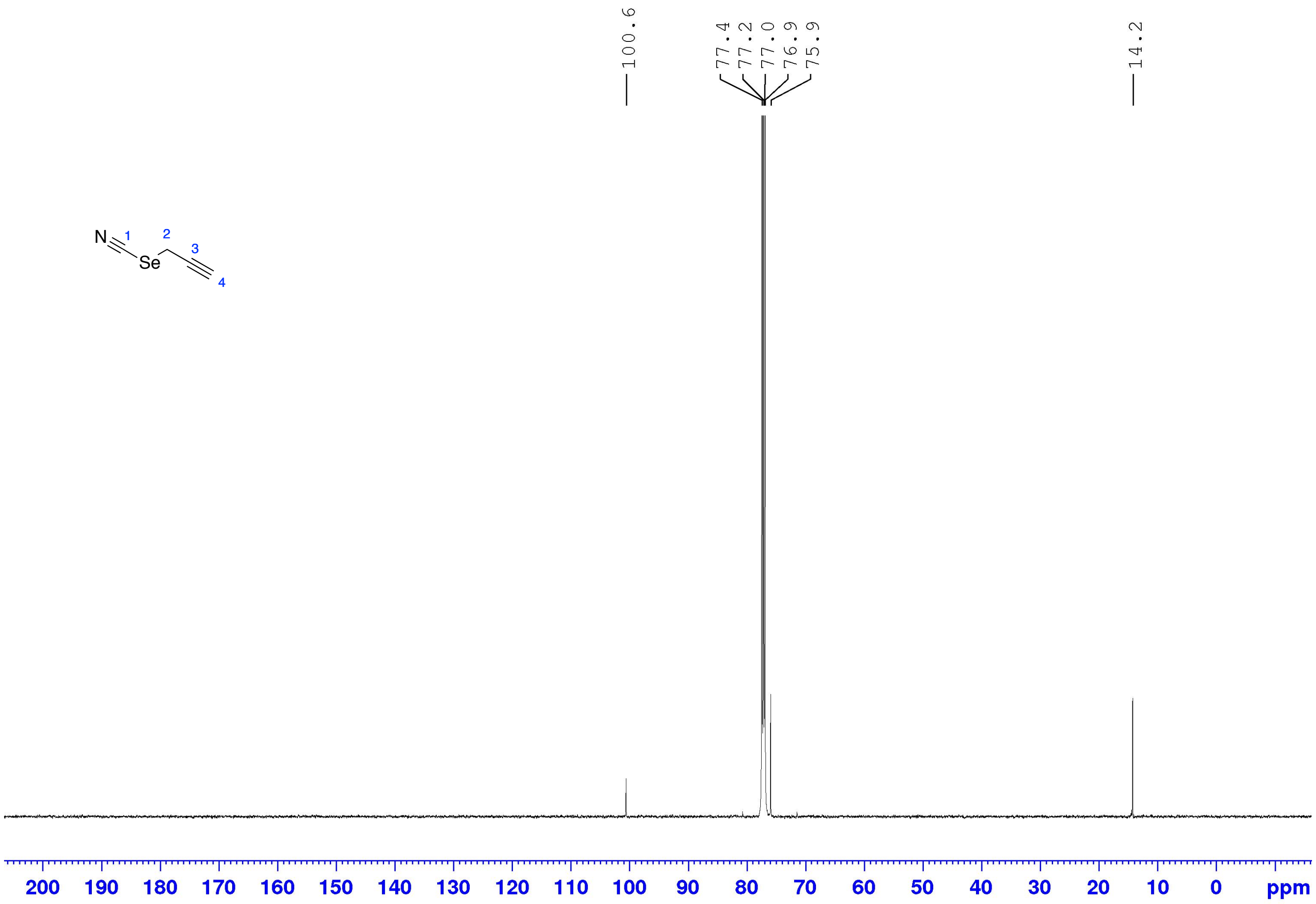

Methyl 2-acetamido-3-(prop-2-yn-1-ylselanyl)propanoate (**20**), ^{1}H NMR ($CDCl_3$, 400 MHz)

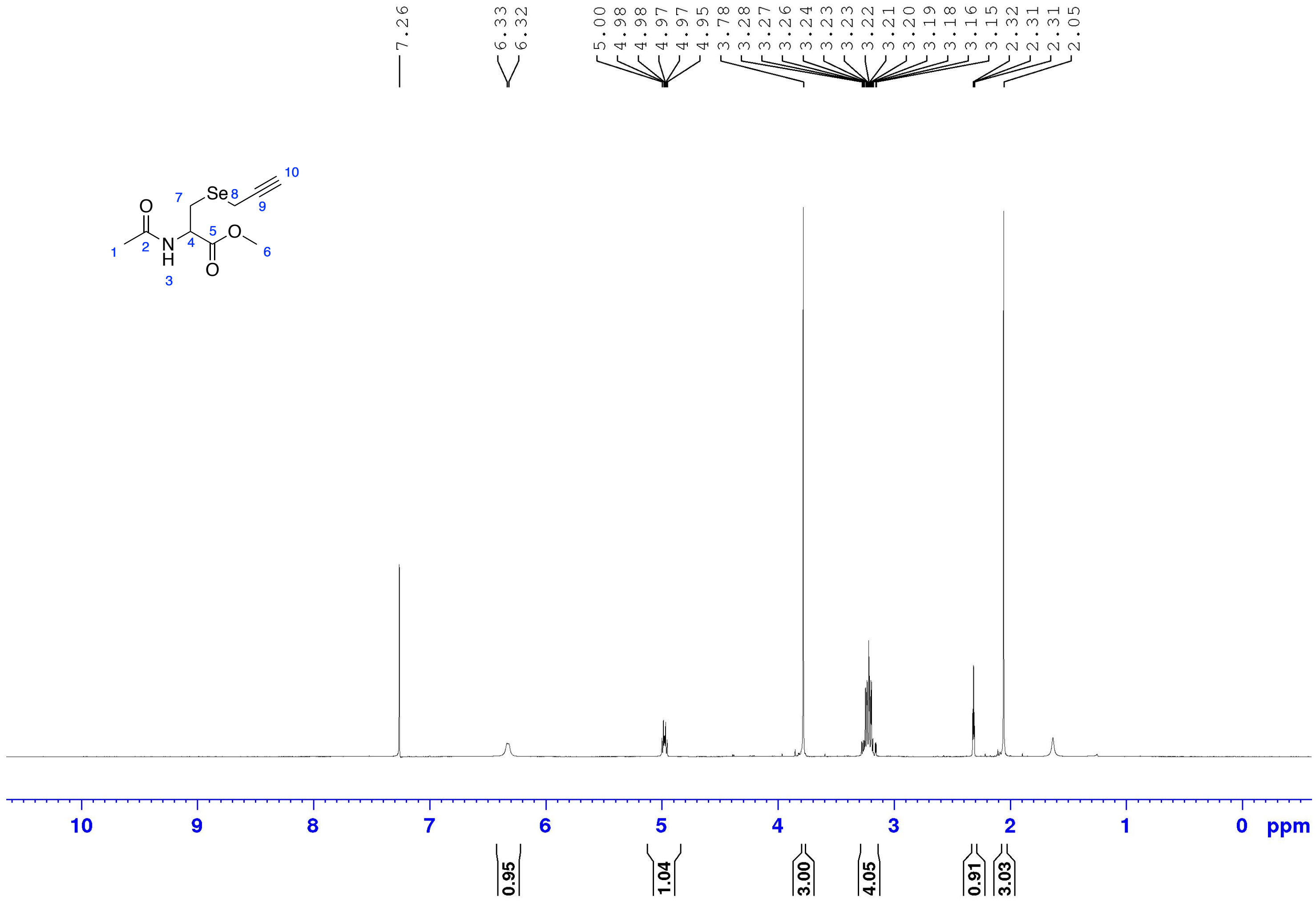

Methyl 2-acetamido-3-(prop-2-yn-1-ylselanyl)propanoate (**20**), ^{13}C NMR ($CDCl_3$, 101 MHz)

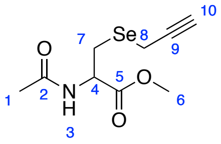

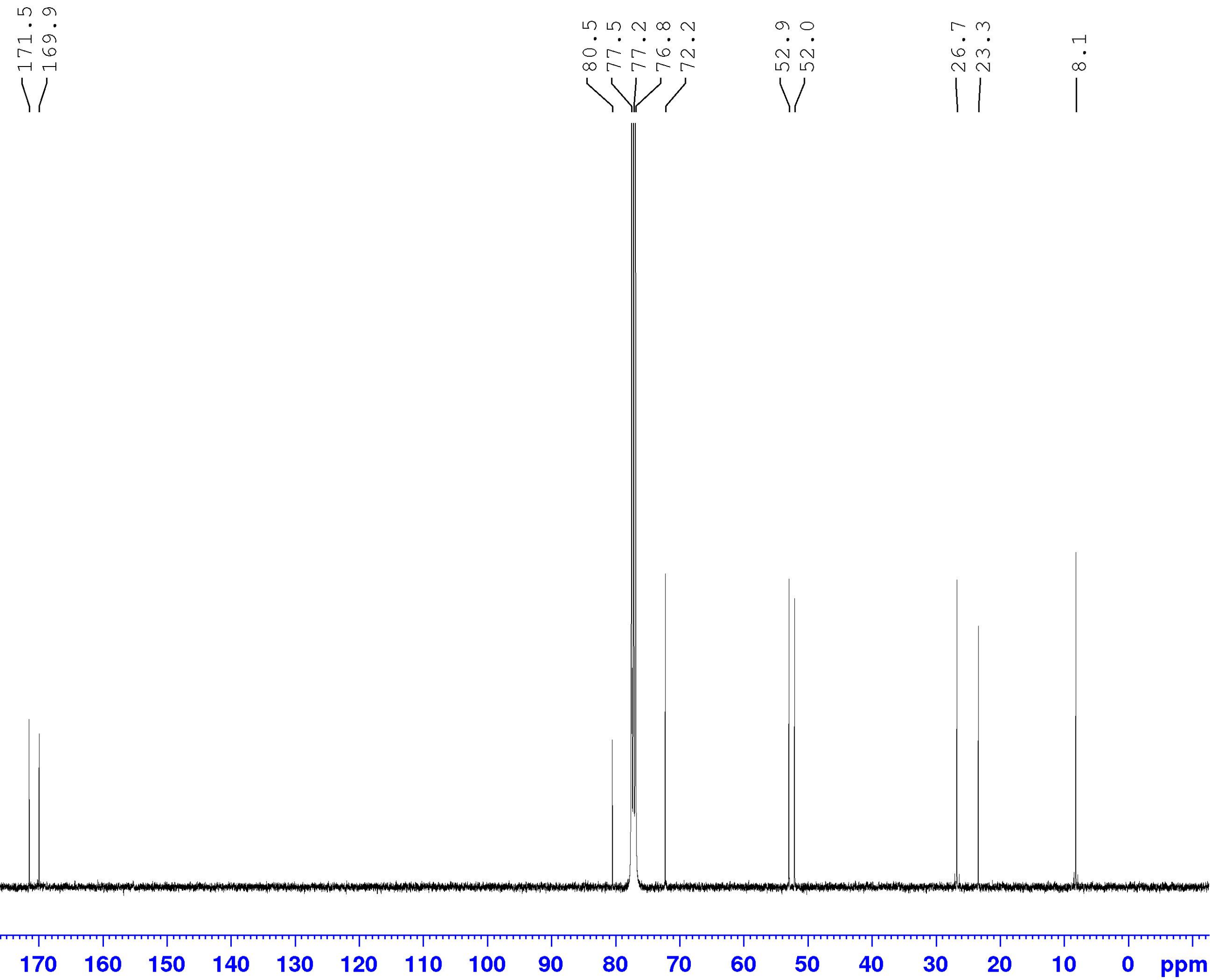

S-(2-(2-(2-(Prop-2-yn-1-yloxy)ethoxy)ethoxy)ethyl) ethanethioate (**40**), ^{1}H NMR ($CDCl_3$, 500 MHz)

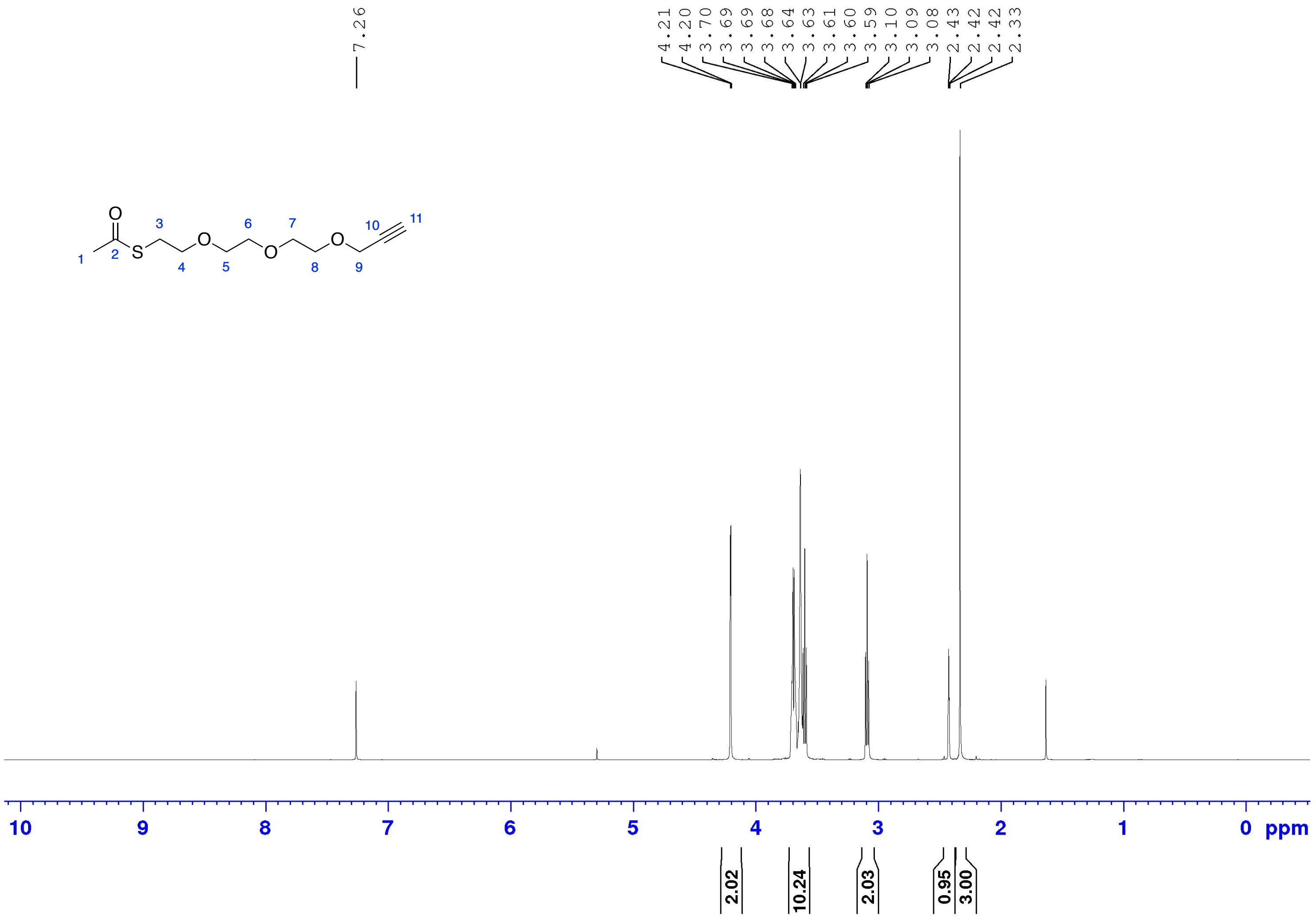

S-(2-(2-(2-(Prop-2-yn-1-yloxy)ethoxy)ethoxy)ethyl) ethanethioate (**40**), ^{13}C NMR ($CDCl_3$, 126 MHz)

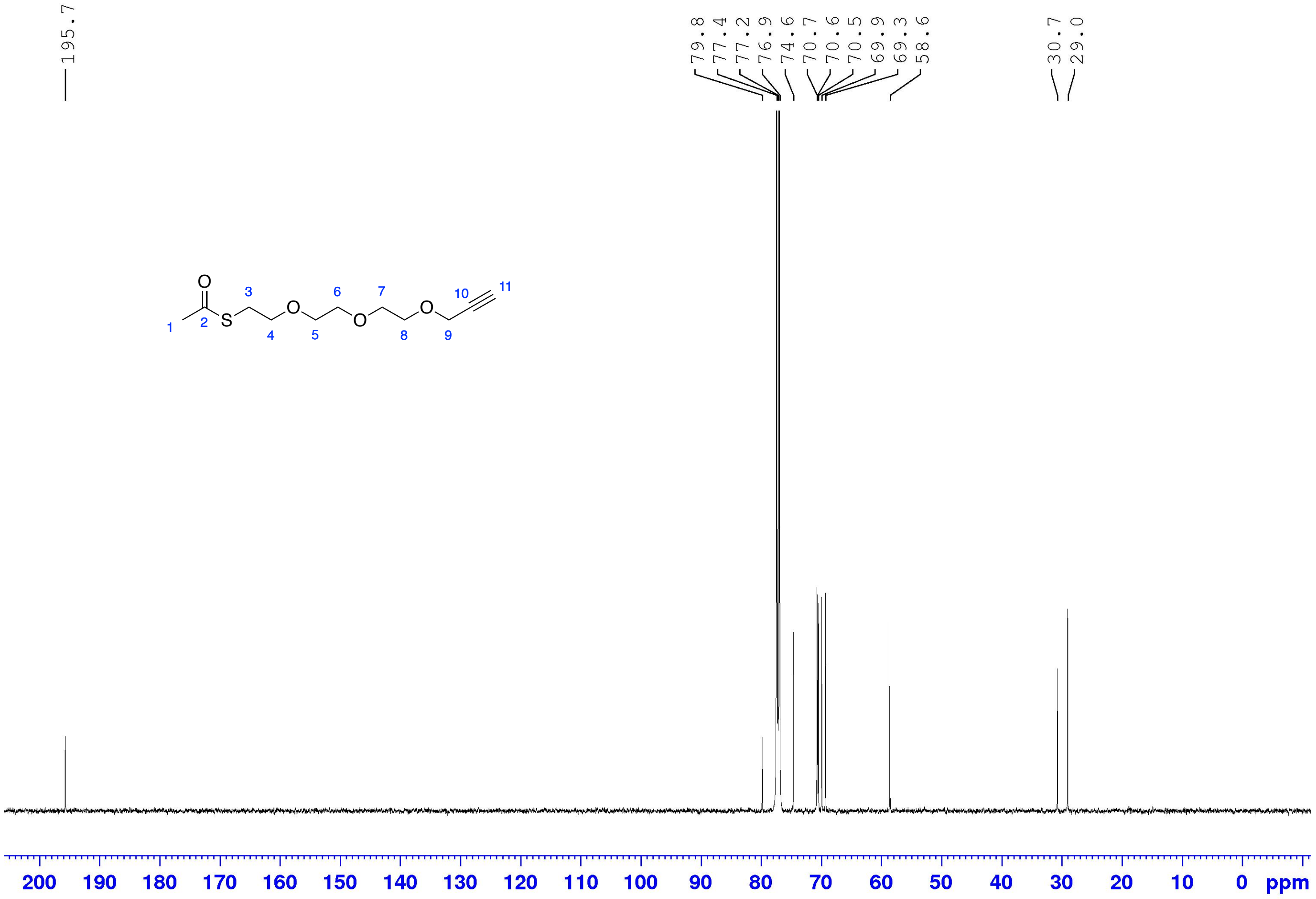

Methyl 15-acetamido-4,7,10-trioxa-13-thiahexadec-1-yn-16-oate (**21**), ^{1}H NMR ($CDCl_3$, 500 MHz)

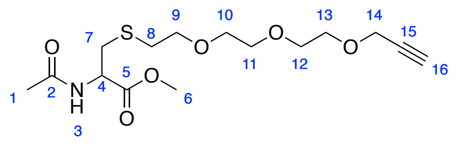

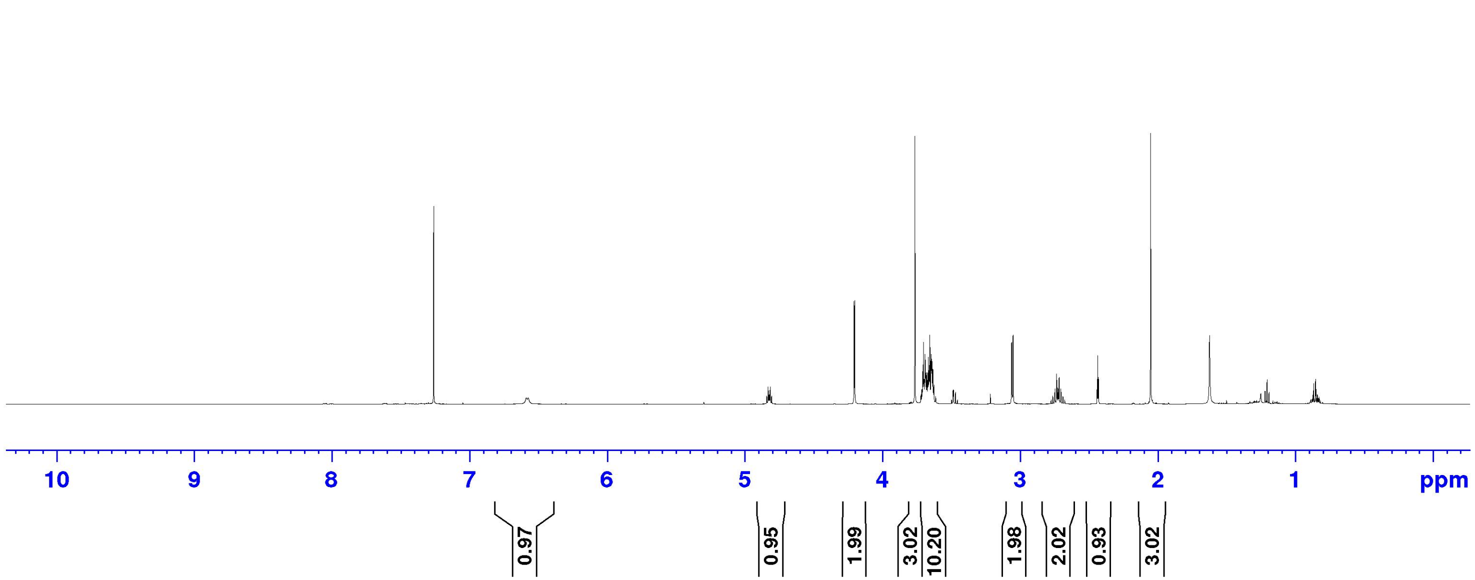

Methyl 15-acetamido-4,7,10-trioxa-13-thiahexadec-1-yn-16-oate (**21**), ^{13}C NMR ($CDCl_3$, 126 MHz)

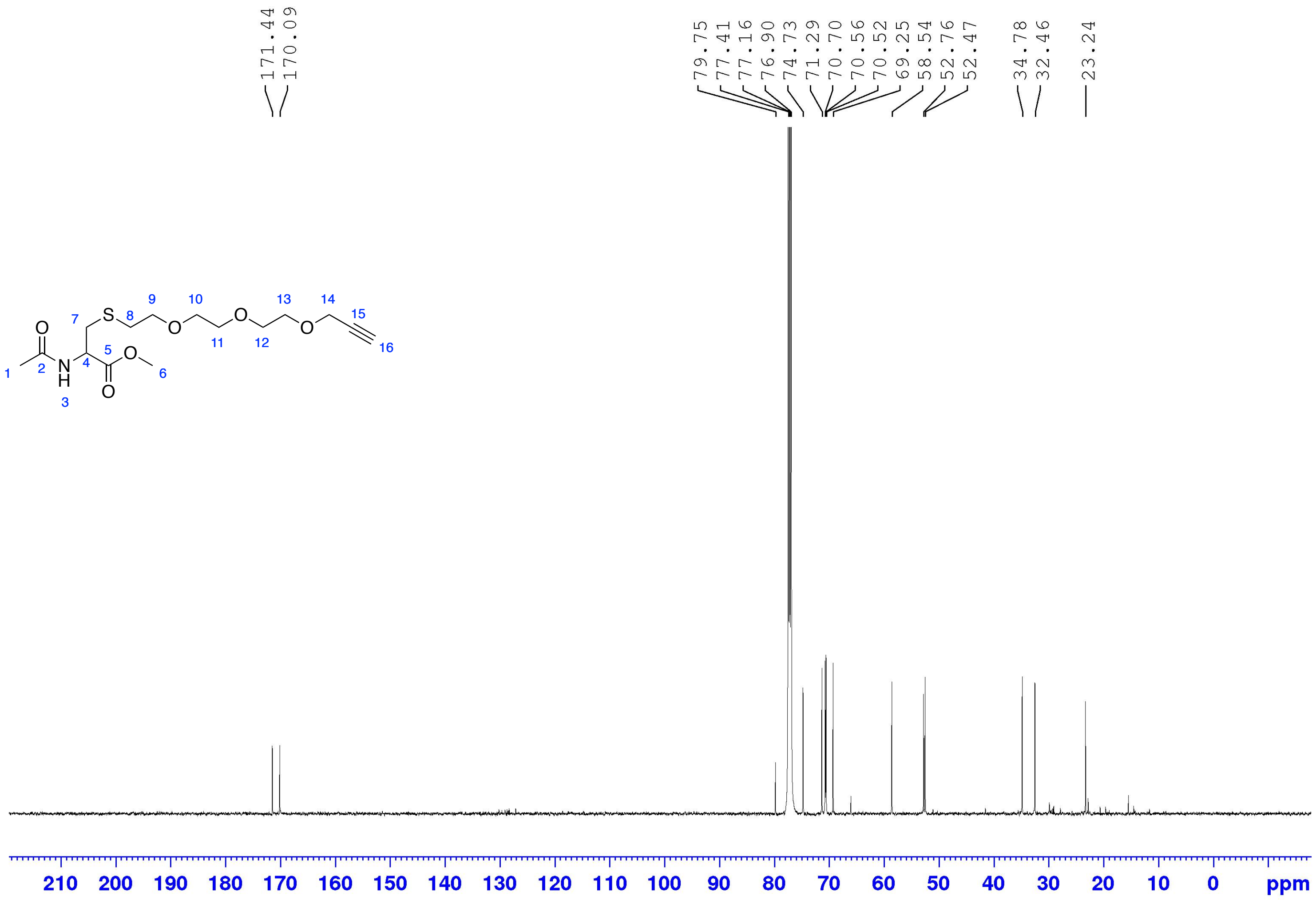

S-(Prop-2-yn-1-yl) ethanethioate (**S15**), ^{1}H NMR ($CDCl_3$, 400 MHz)

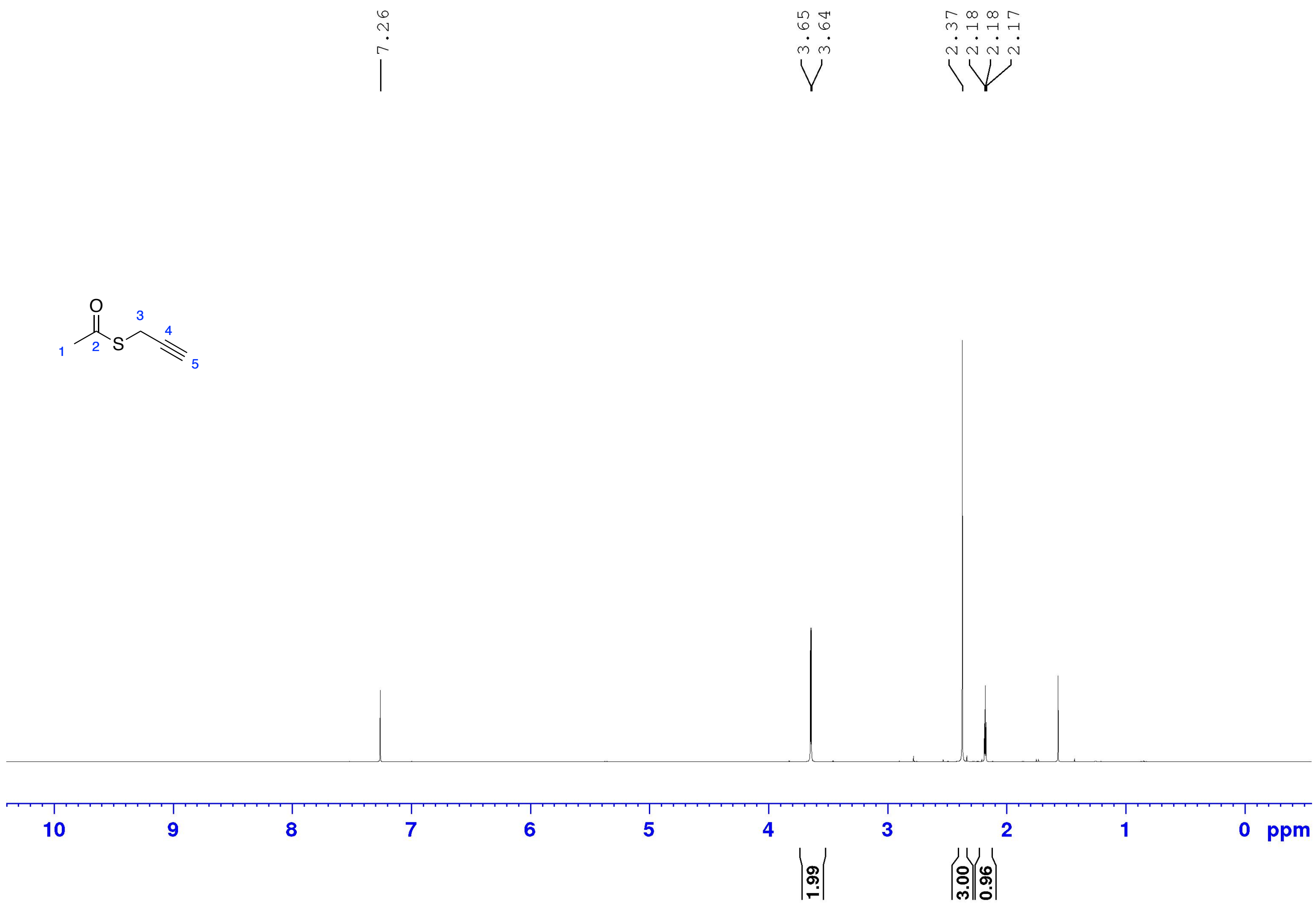

S-(Prop-2-yn-1-yl) ethanethioate (**S15**), ^{13}C NMR ($CDCl_3$, 101 MHz)

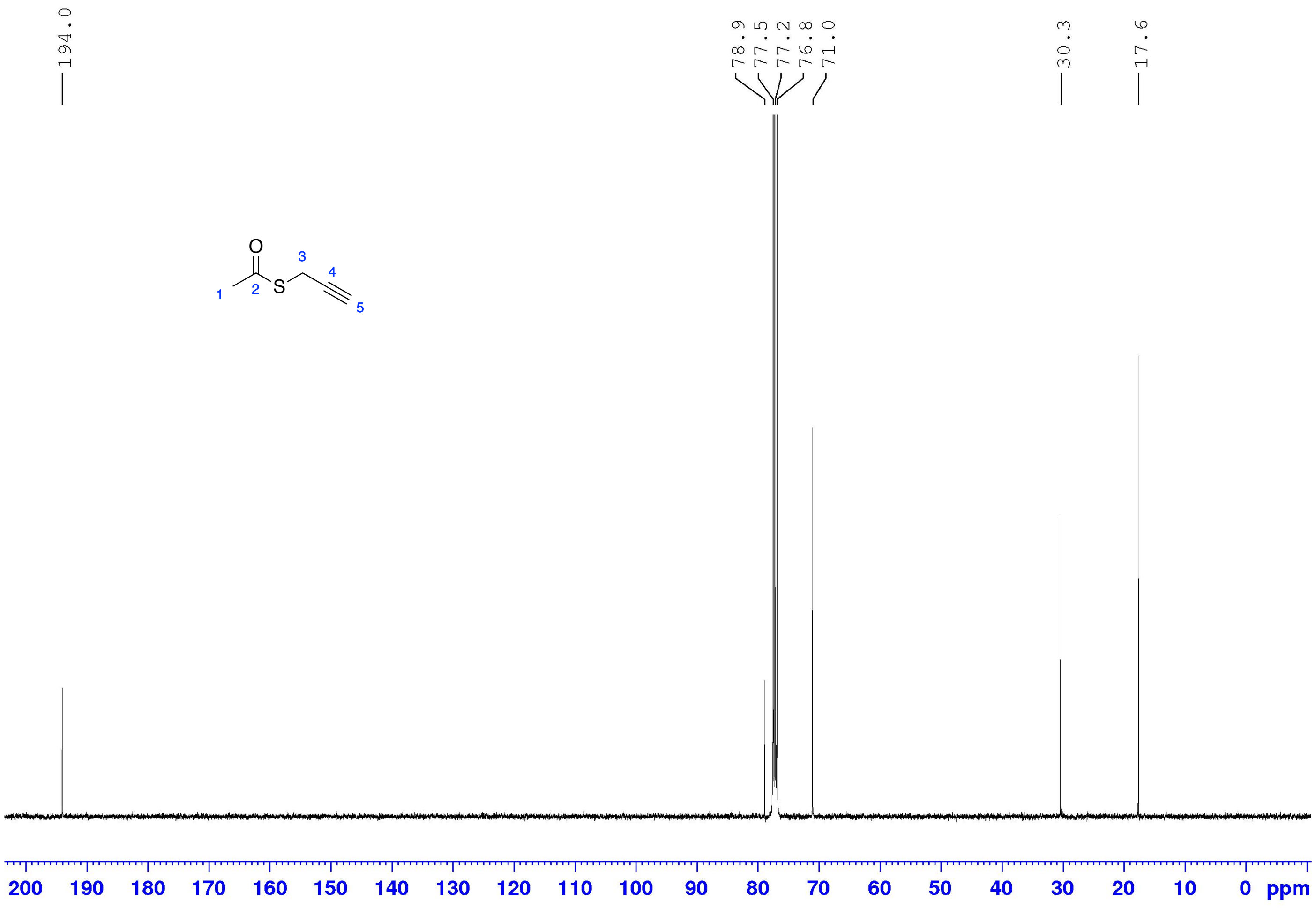

N,*N*-Diethyl-2-hydroxy-*N*-(4-(2-methyl-3-methylene-5,8,11,14-tetraoxa-2-silahexadecan-2-yl)benzyl)ethanammonium bromide (**S16**), ^{1}H NMR ($CDCl_3$, 500 MHz)

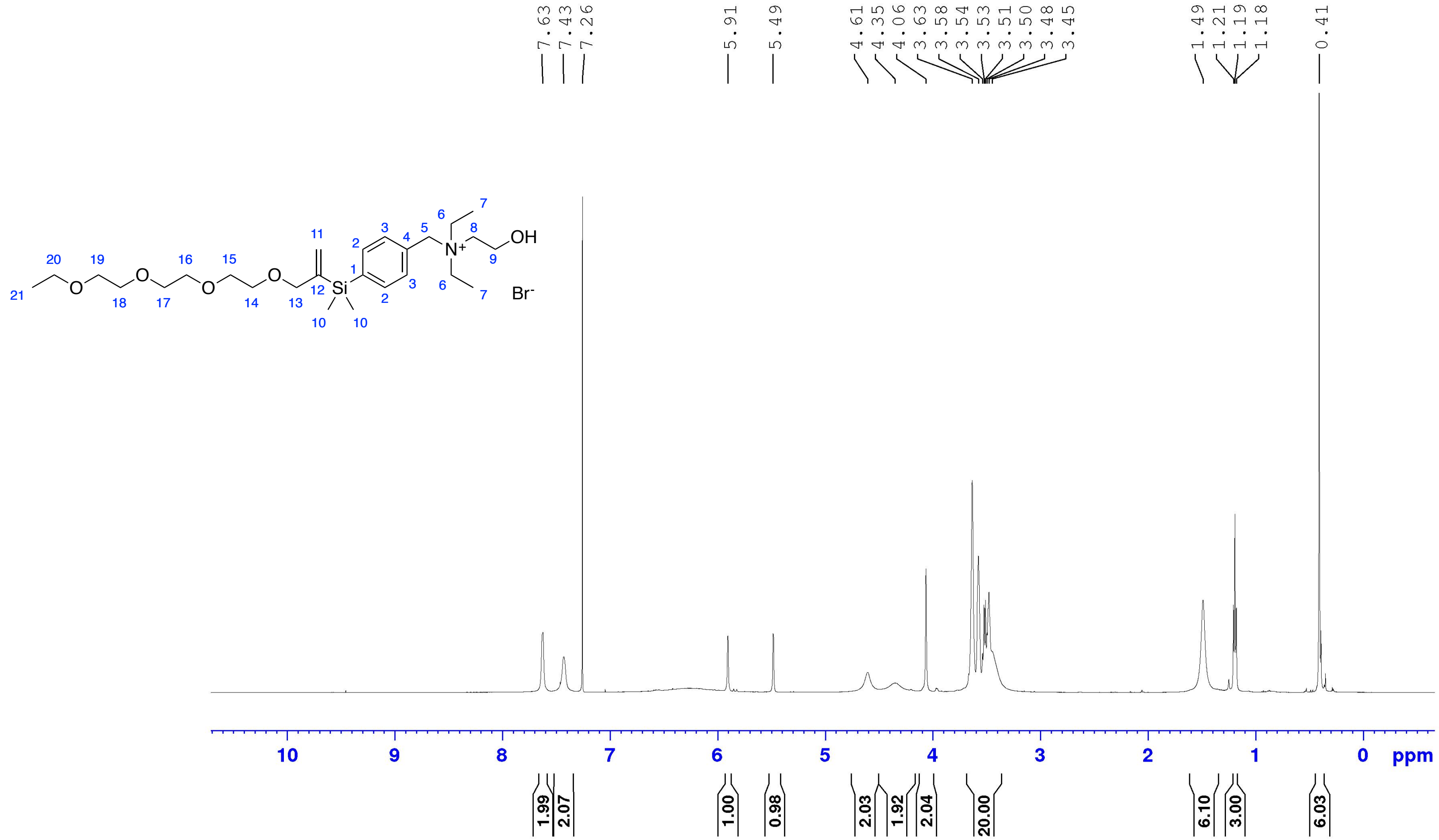

N,*N*-Diethyl-2-hydroxy-*N*-(4-(2-methyl-3-methylene-5,8,11,14-tetraoxa-2-silahexadecan-2-yl)benzyl)ethanammonium bromide (**S16**), ^{13}C NMR ($CDCl_3$, 126 MHz)

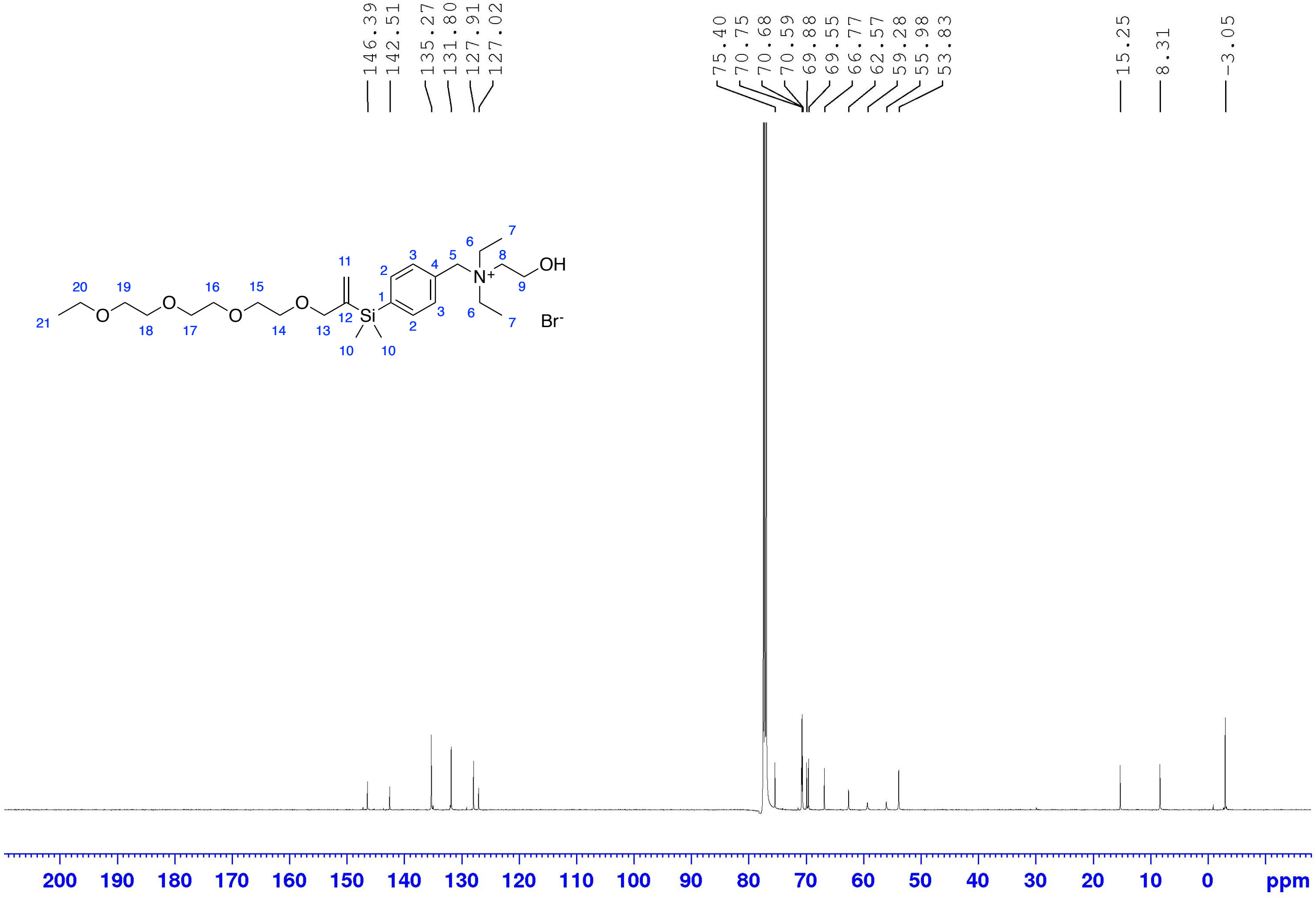

N-(2-(2-(2-Hydroxyethoxy)ethoxy)ethyl)-4-(2-methyl-3-methylene-5,8,11,14-tetraoxa-2-silahexadecan-2-yl)benzamide (**S17**), ^{1}H NMR (DMSO-d_6, 500 MHz)

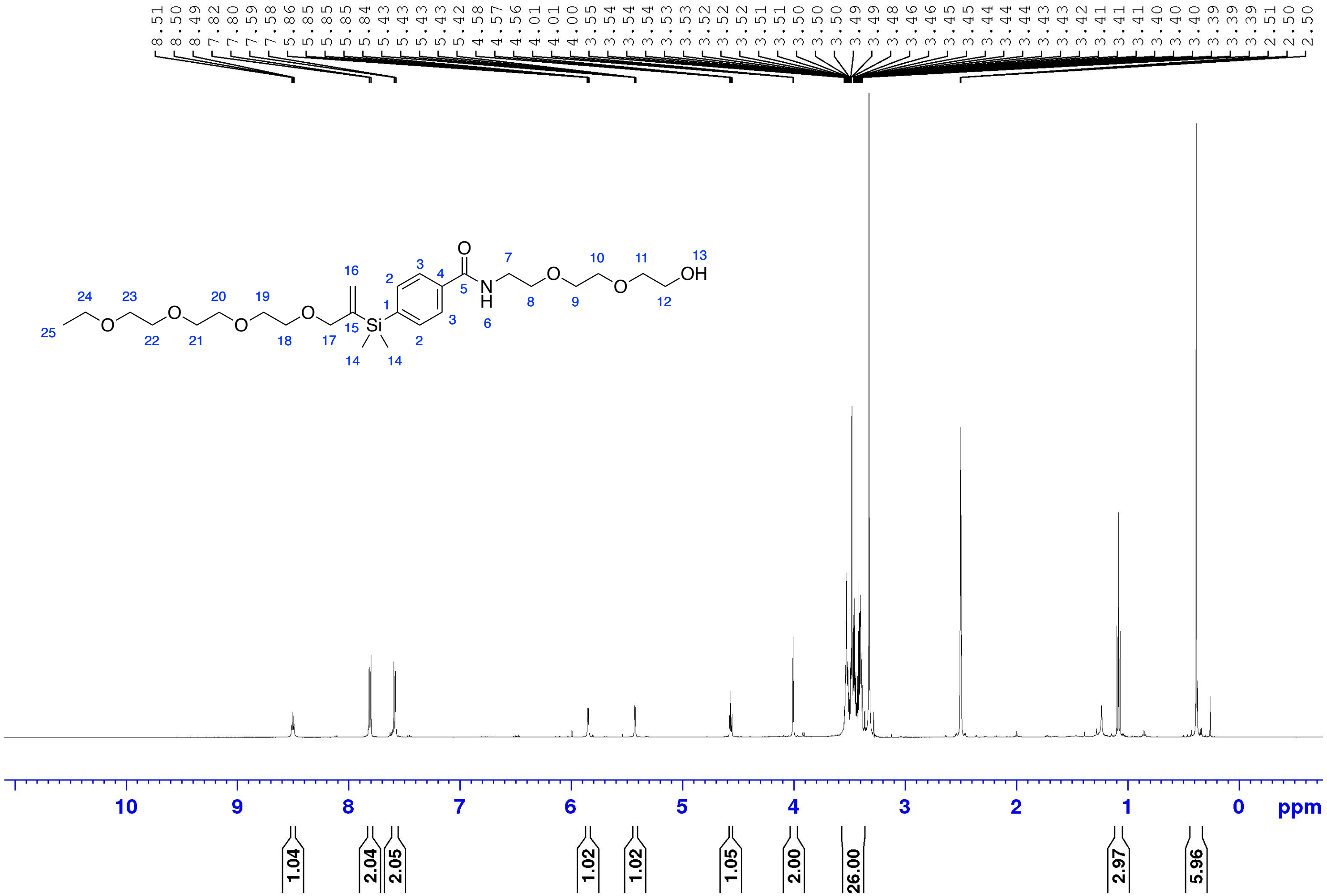

N-(2-(2-(2-Hydroxyethoxy)ethoxy)ethyl)-4-(2-methyl-3-methylene-5,8,11,14-tetraoxa-2-silahexadecan-2-yl)benzamide (**S17**), ^{13}C NMR (DMSO-d_6, 126 MHz)

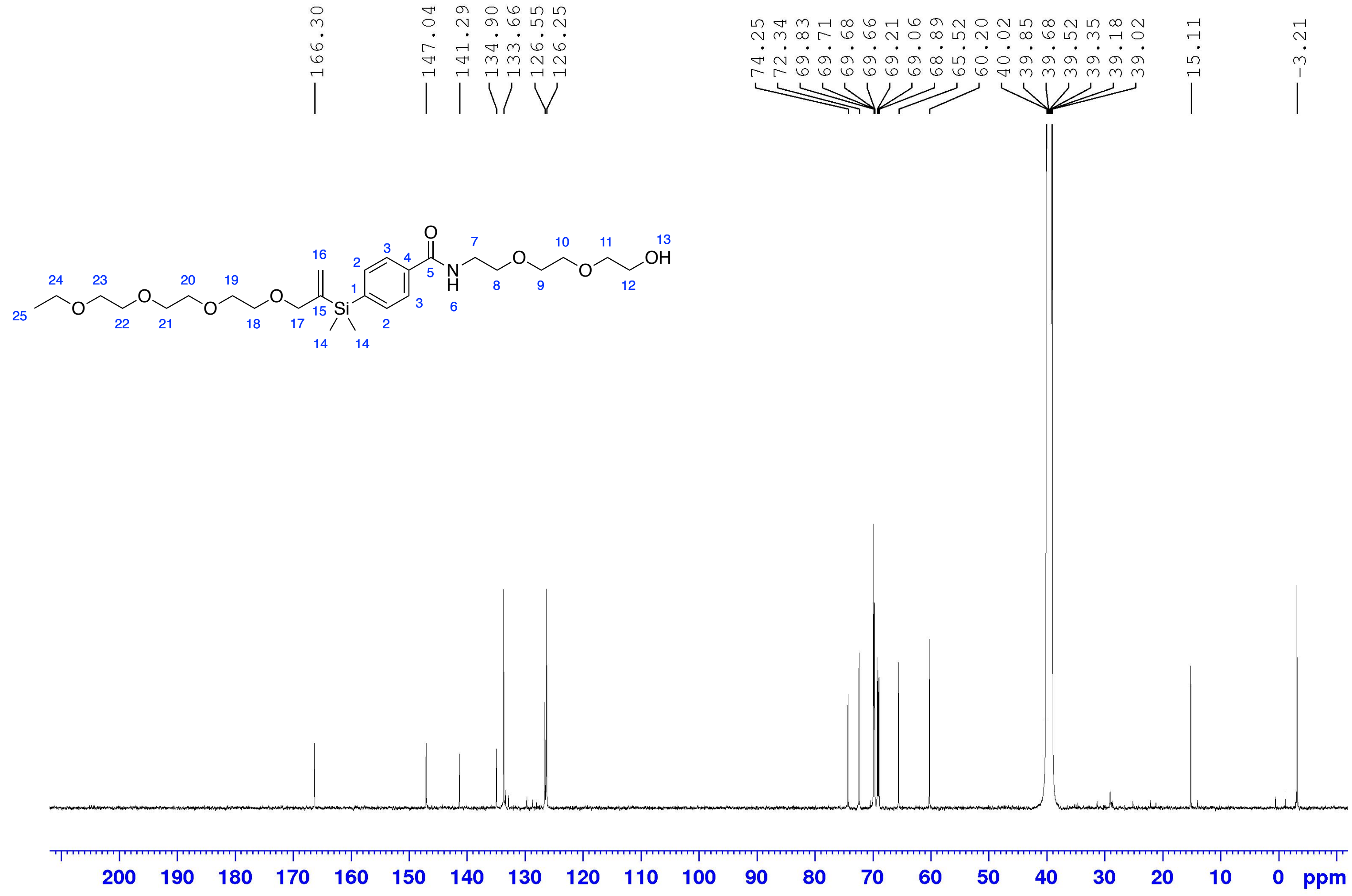

N-(4-((4-((2-(2-(2-Hydroxyethoxy)ethoxy)ethyl)carbamoyl)phenyl)dimethylsilyl)pent-4-en-1-yl)-3,4-dimethoxybenzamide (**10**), ^{1}H NMR (DMSO-d_6, 500 MHz)

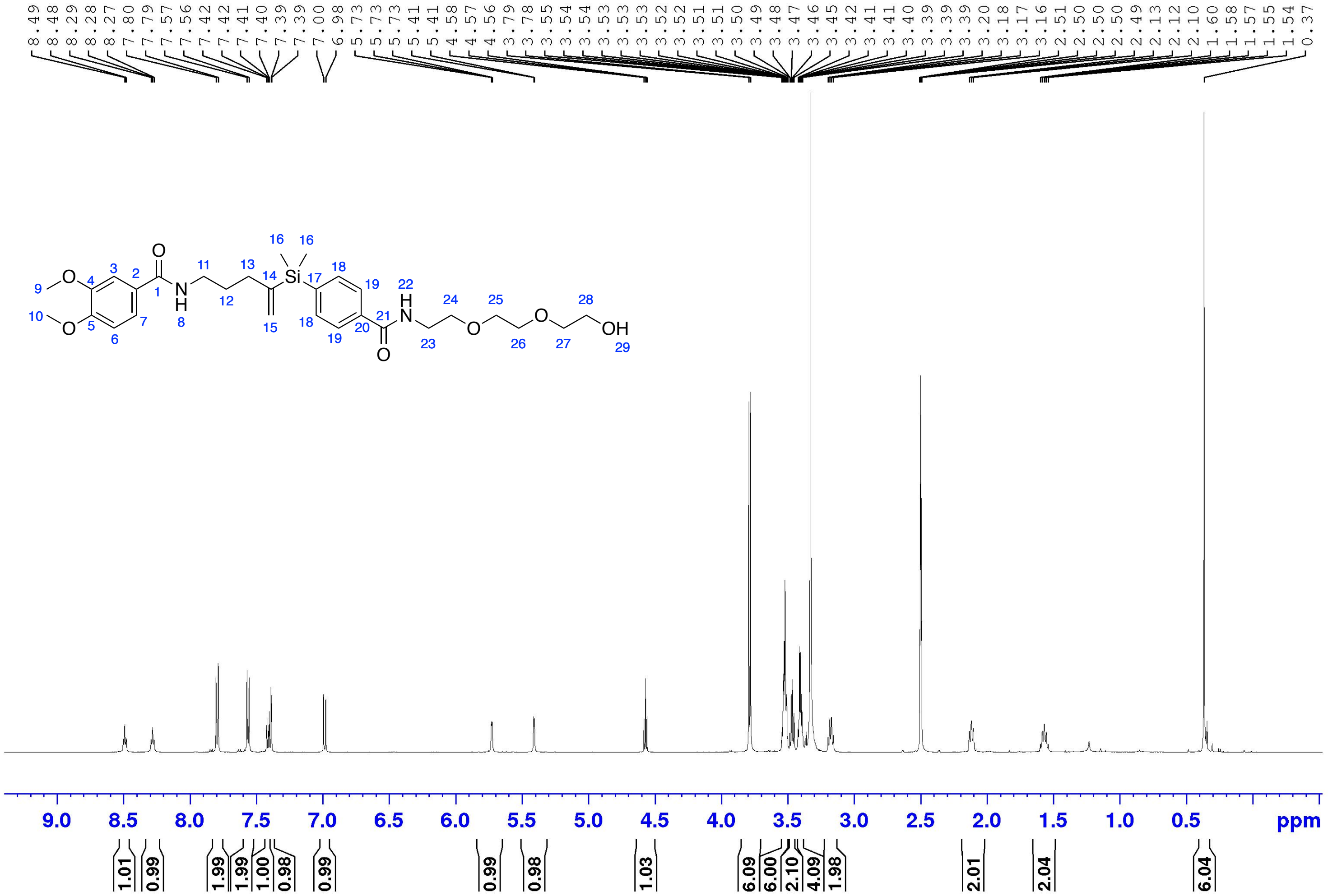

N-(4-((4-((2-(2-(2-Hydroxyethoxy)ethoxy)ethyl)carbamoyl)phenyl)dimethylsilyl)pent-4-en-1-yl)-3,4-dimethoxybenzamide (**10**), ^{13}C NMR (DMSO-d_6, 126 MHz)

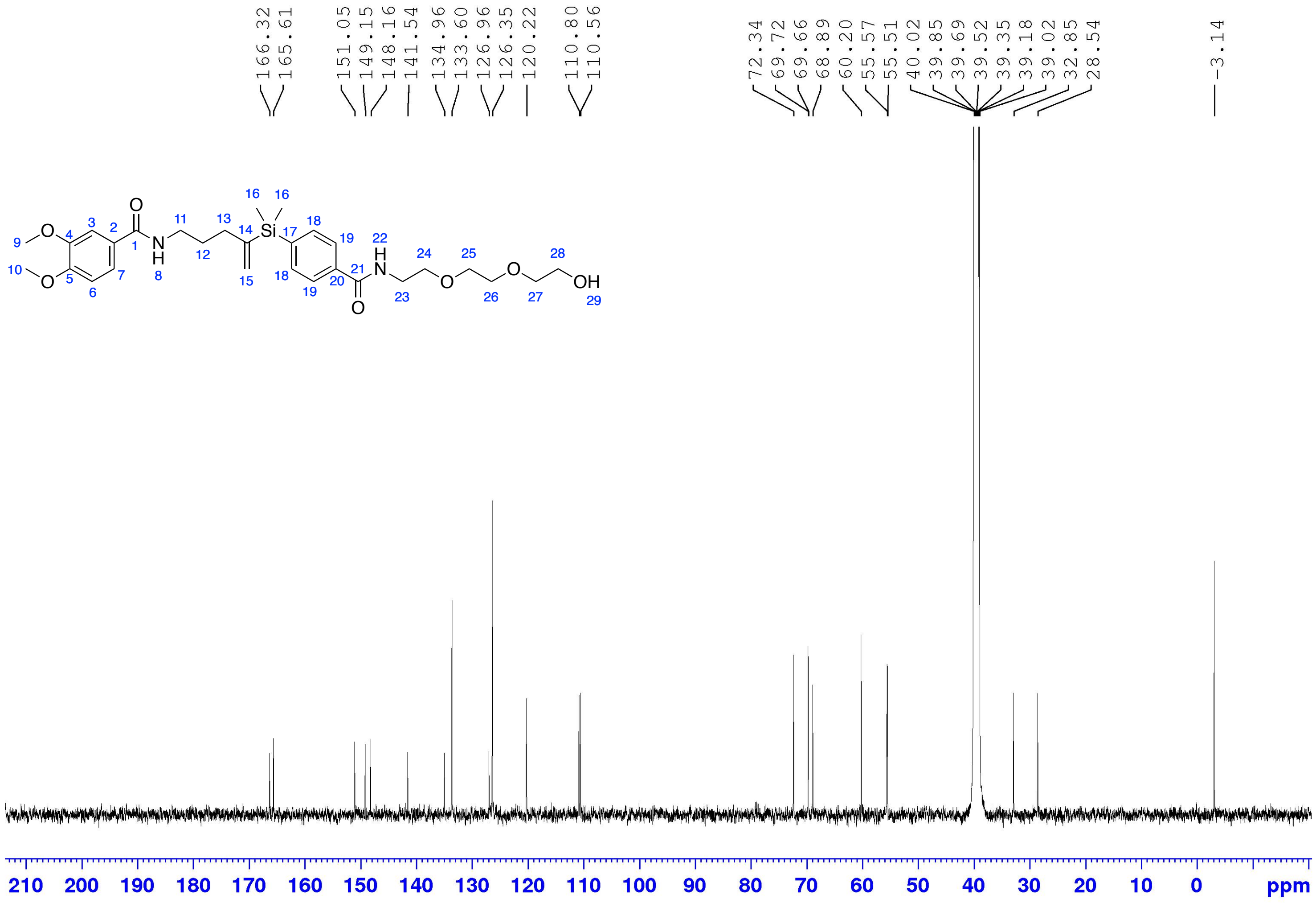

N-(2-(4-((2-(2-(2-Hydroxyethoxy)ethoxy)ethyl)carbamoyl)phenyl)-2-methyl-3-methylene-5,8,11-trioxa-2-silatridecan-13-yl)-3,4-dimethoxybenzamide (**14**), ^{1}H NMR (DMSO-d_6, 500 MHz)

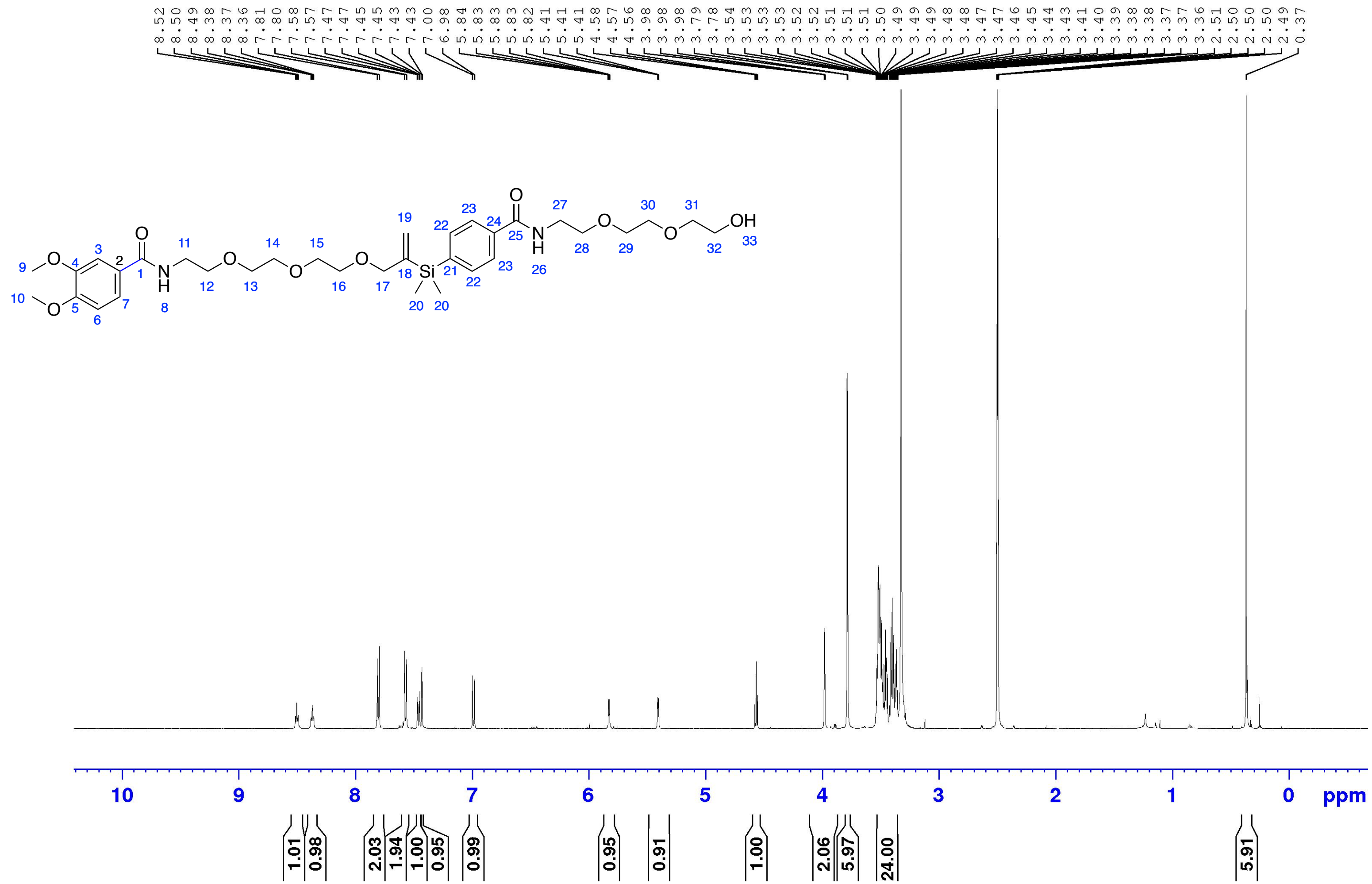

N-(2-(4-((2-(2-(2-Hydroxyethoxy)ethoxy)ethyl)carbamoyl)phenyl)-2-methyl-3-methylene-5,8,11-trioxa-2-silatridecan-13-yl)-3,4-dimethoxybenzamide (**14**), ^{13}C NMR (DMSO-d_6, 126 MHz)

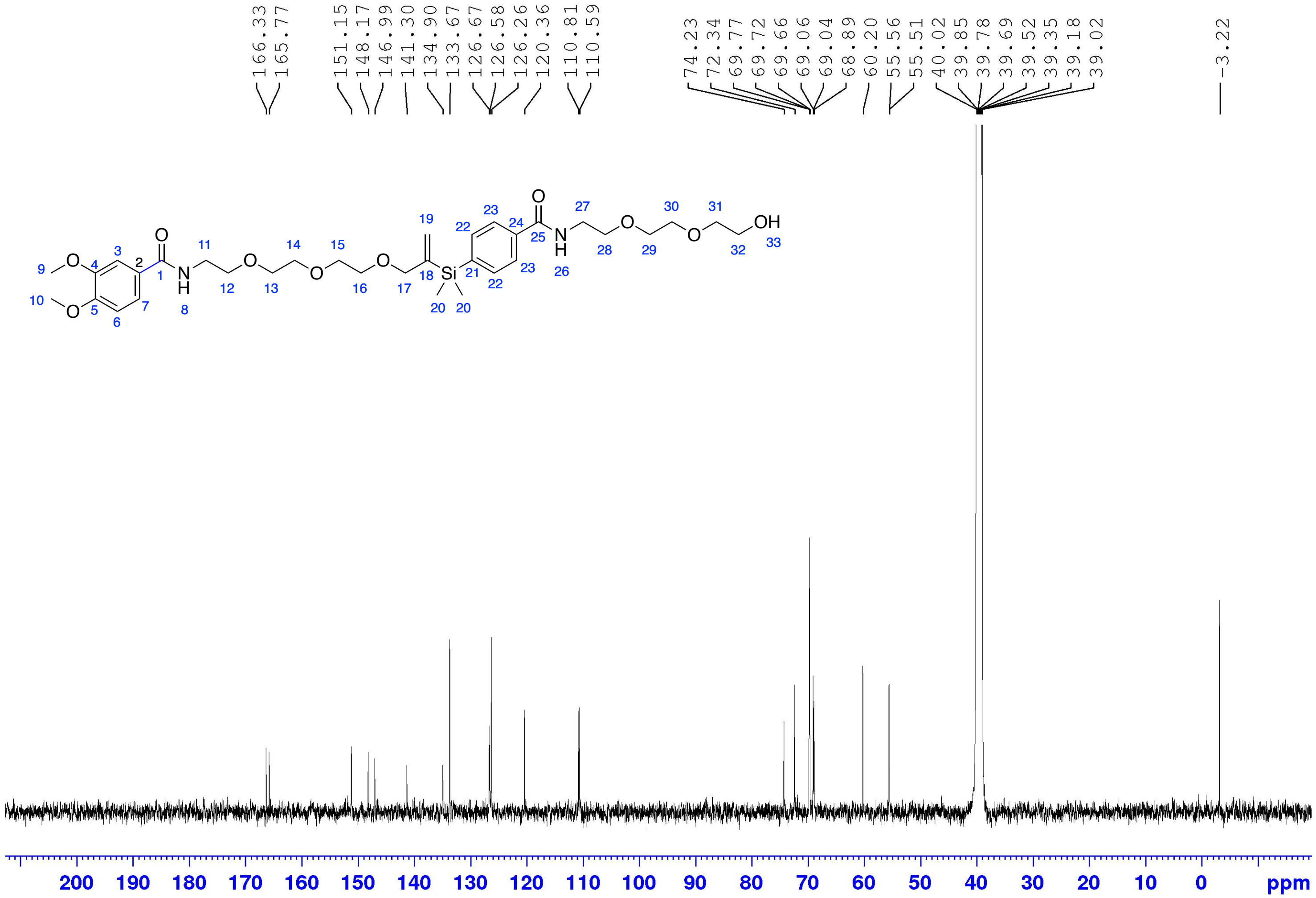

Methyl *N*-(Boc)-*O*-(2-(dimethyl(phenyl)silyl)allyl)-L-serinate (**17**), ^{1}H NMR ($CDCl_3$, 500 MHz)

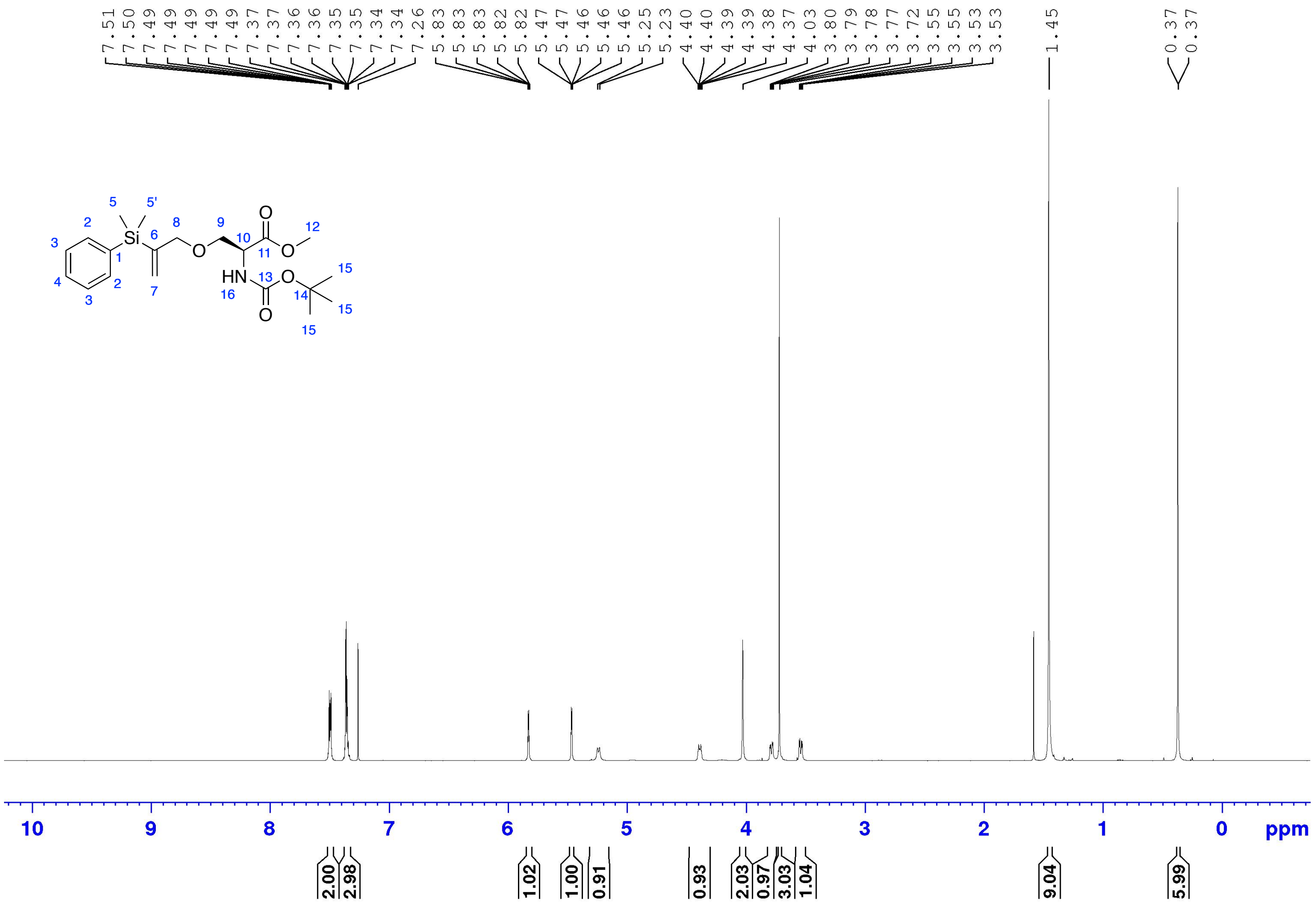

Methyl *N*-(Boc)-*O*-(2-(dimethyl(phenyl)silyl)allyl)-L-serinate (**17**), ^{13}C NMR ($CDCl_3$, 126 MHz)

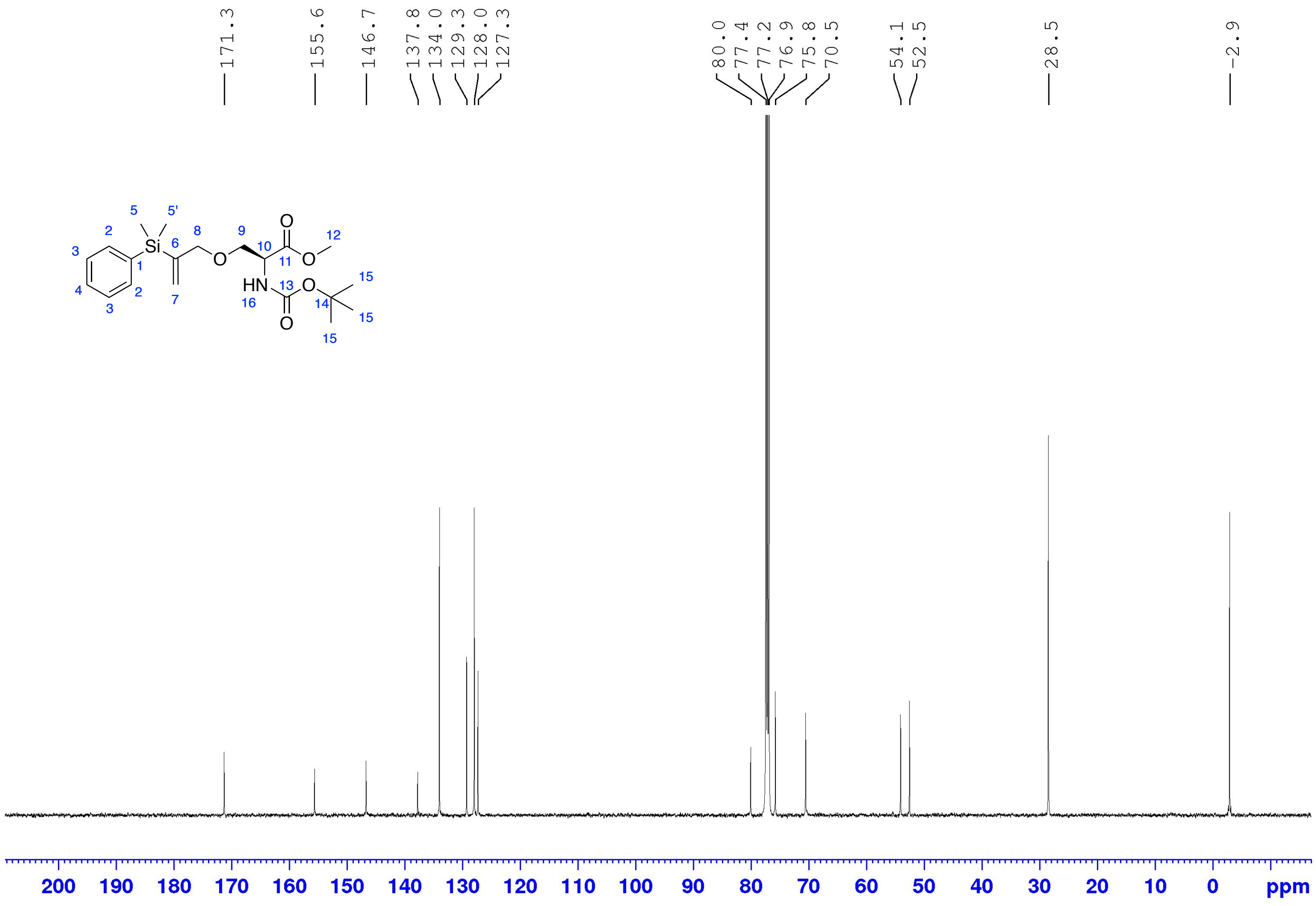

Methyl *N*-(Boc)-*S*-(2-(dimethyl(phenyl)silyl)allyl)-L-cysteinate (**19**), ^{1}H NMR ($CDCl_3$, 400 MHz)

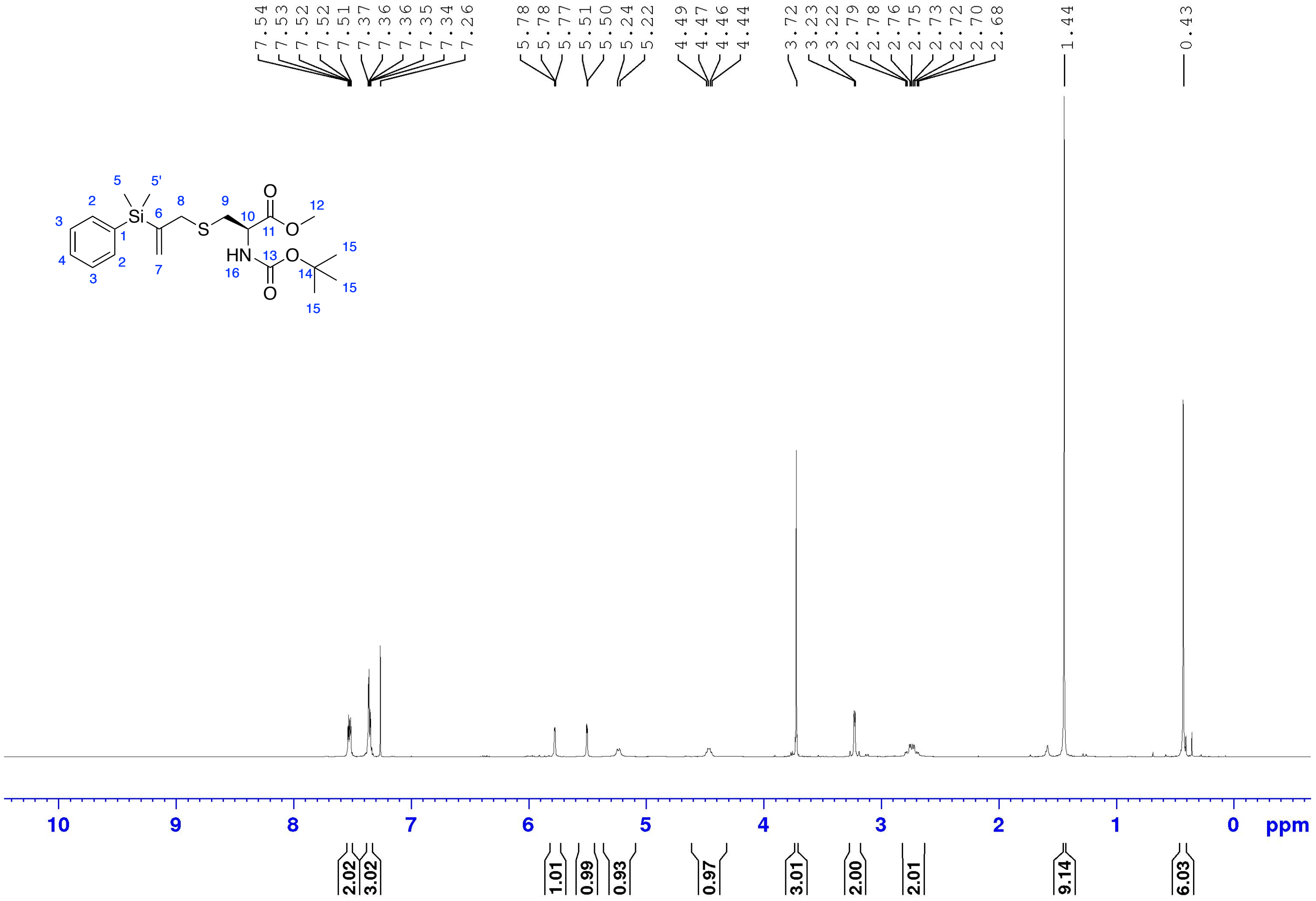

Methyl *N*-(Boc)-*S*-(2-(dimethyl(phenyl)silyl)allyl)-L-cysteinate (**19**), ^{13}C NMR ($CDCl_3$, 101 MHz)

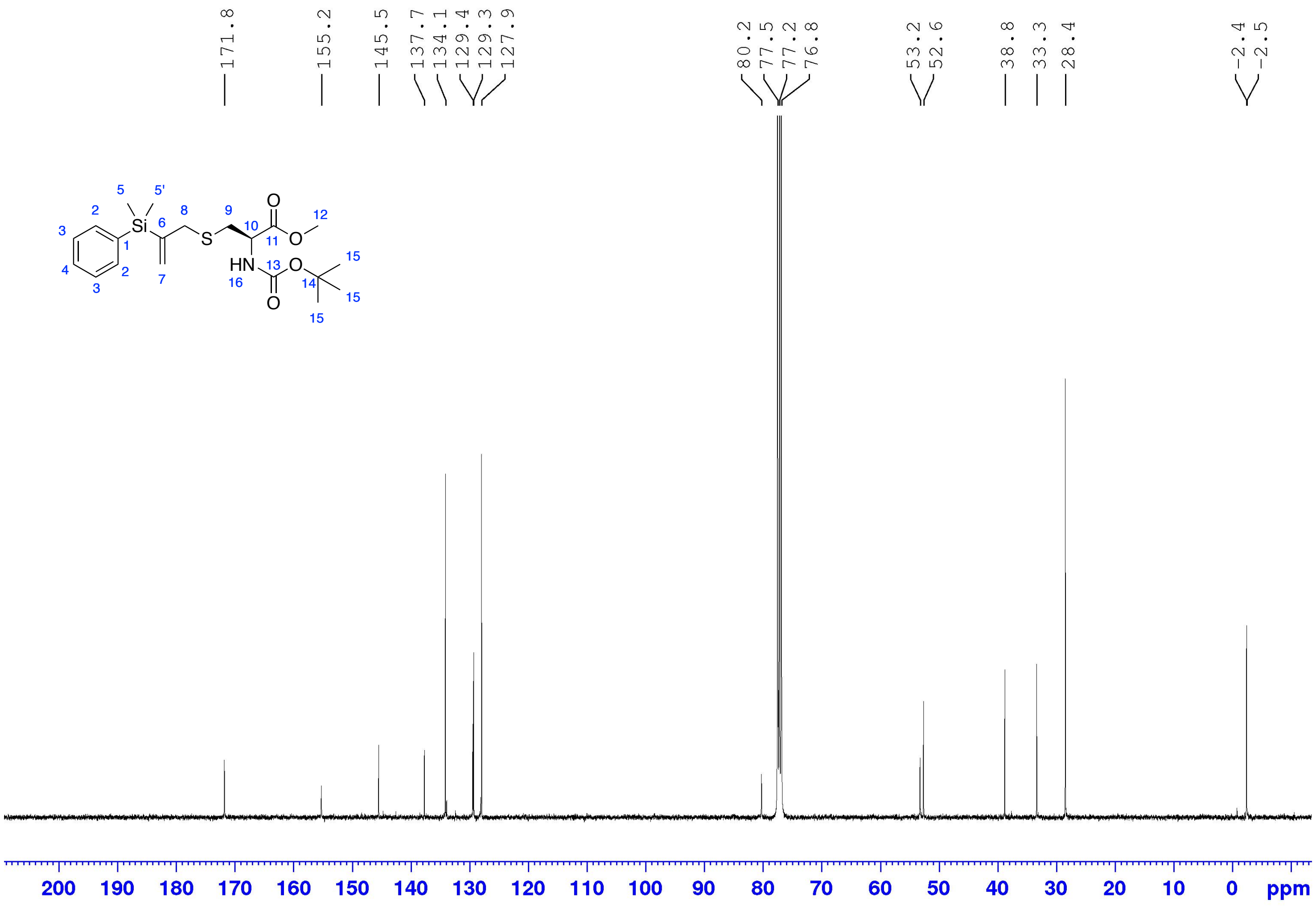

4-(Dimethyl(3-(((2*R*,3*R*,4*S*,5*S*,6*R*)-3,4,5-trihydroxy-6-(hydroxymethyl)-tetrahydro-2*H*-pyran-2-yl)oxy)prop-1-en-2-yl)silyl)-*N*-(2-(2-(2-hydroxyethoxy)ethoxy)ethyl) benzamide (**26**), ^{1}H NMR (DMSO-d_6, 500 MHz)

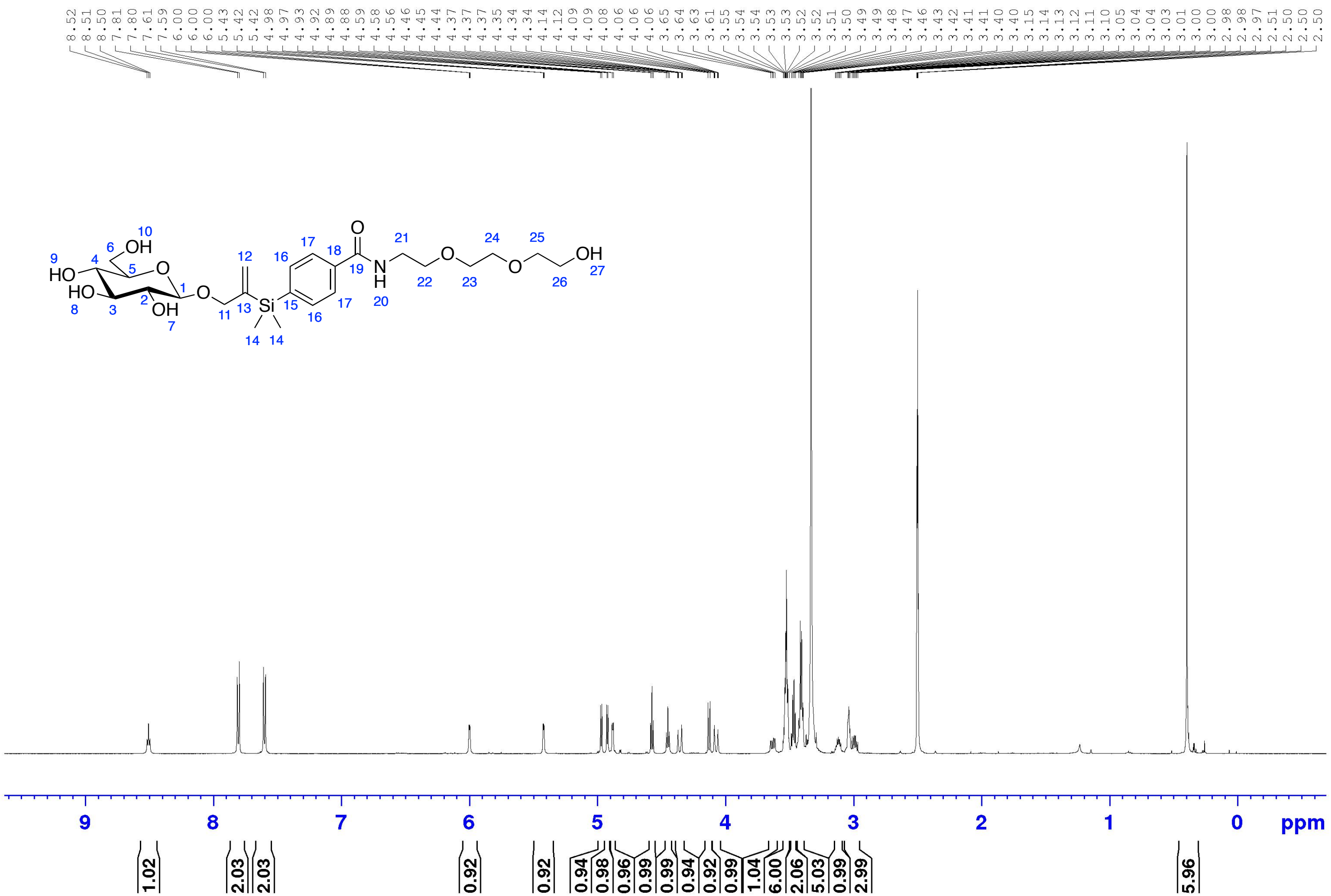

4-(Dimethyl(3-(((2*R*,3*R*,4*S*,5*S*,6*R*)-3,4,5-trihydroxy-6-(hydroxymethyl)-tetrahydro-2*H*-pyran-2-yl)oxy)prop-1-en-2-yl)silyl)-*N*-(2-(2-(2-hydroxyethoxy)ethoxy)ethyl) benzamide (**26**), ^{13}C NMR (DMSO-d_6, 126 MHz)

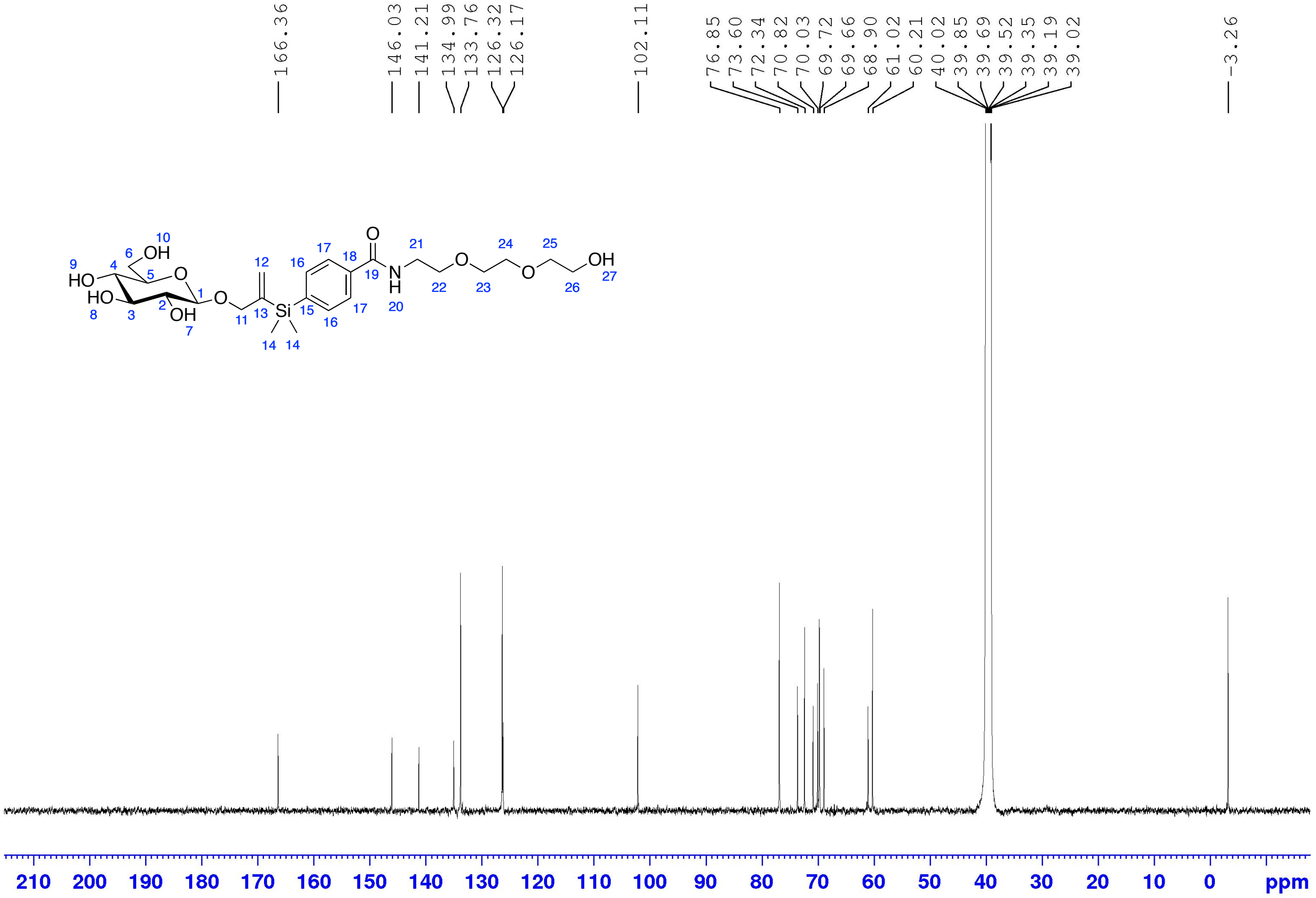

Methyl N^2-(Boc)-N^6-(((2-((4-((2-(2-(2-hydroxyethoxy)ethoxy)ethyl)carbamoyl) phenyl)dimethylsilyl)allyl)oxy)carbonyl)-L-lysinate (**24**), ^{1}H NMR (DMSO-d_6, 500 MHz)

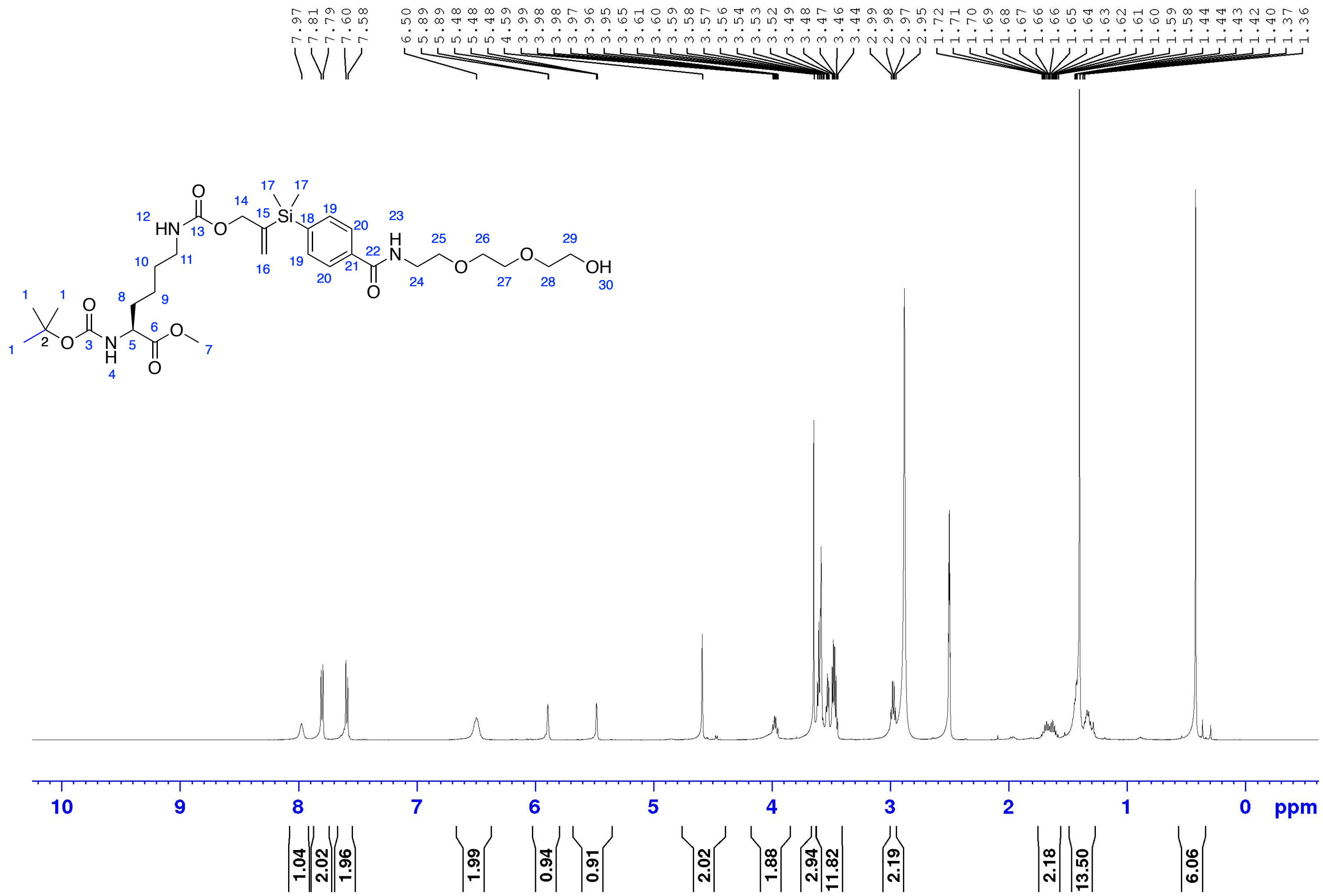

Methyl N^2-(Boc)-N^6-(((2-((4-((2-(2-(2-hydroxyethoxy)ethoxy)ethyl)carbamoyl) phenyl)dimethylsilyl)allyl)oxy)carbonyl)-L-lysinate (**24**), ^{13}C NMR (DMSO-d_6, 126 MHz)

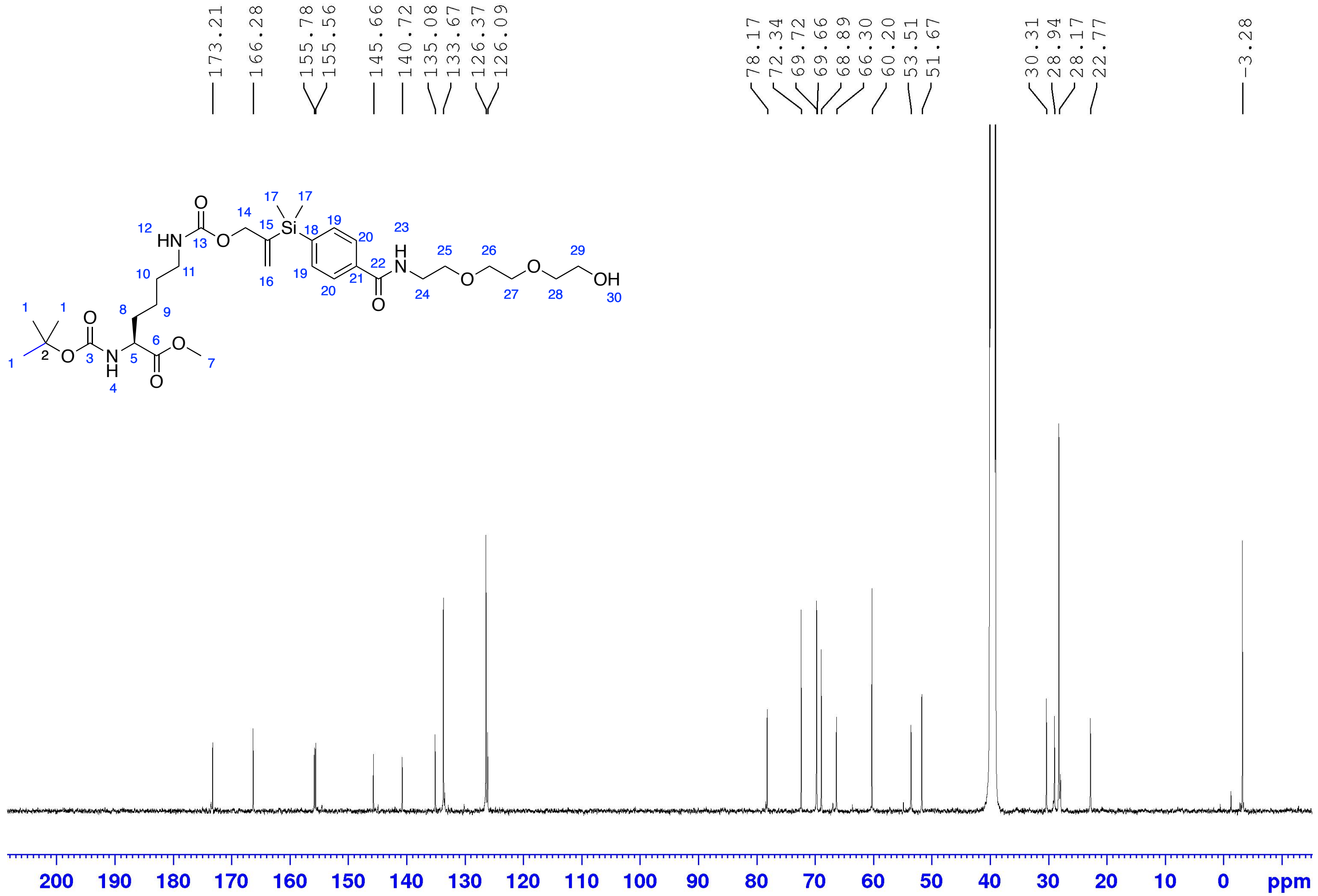

Methyl 16-acetamido-2-(4-((2-(2-(2-hydroxyethoxy)ethoxy)ethyl)carbamoyl)phenyl)-2-methyl-3-methylene-5,8,11-trioxa-14-thia-2-sila-heptadecan-17-oate (**22**), ^{1}H NMR ($CDCl_3$, 500 MHz)

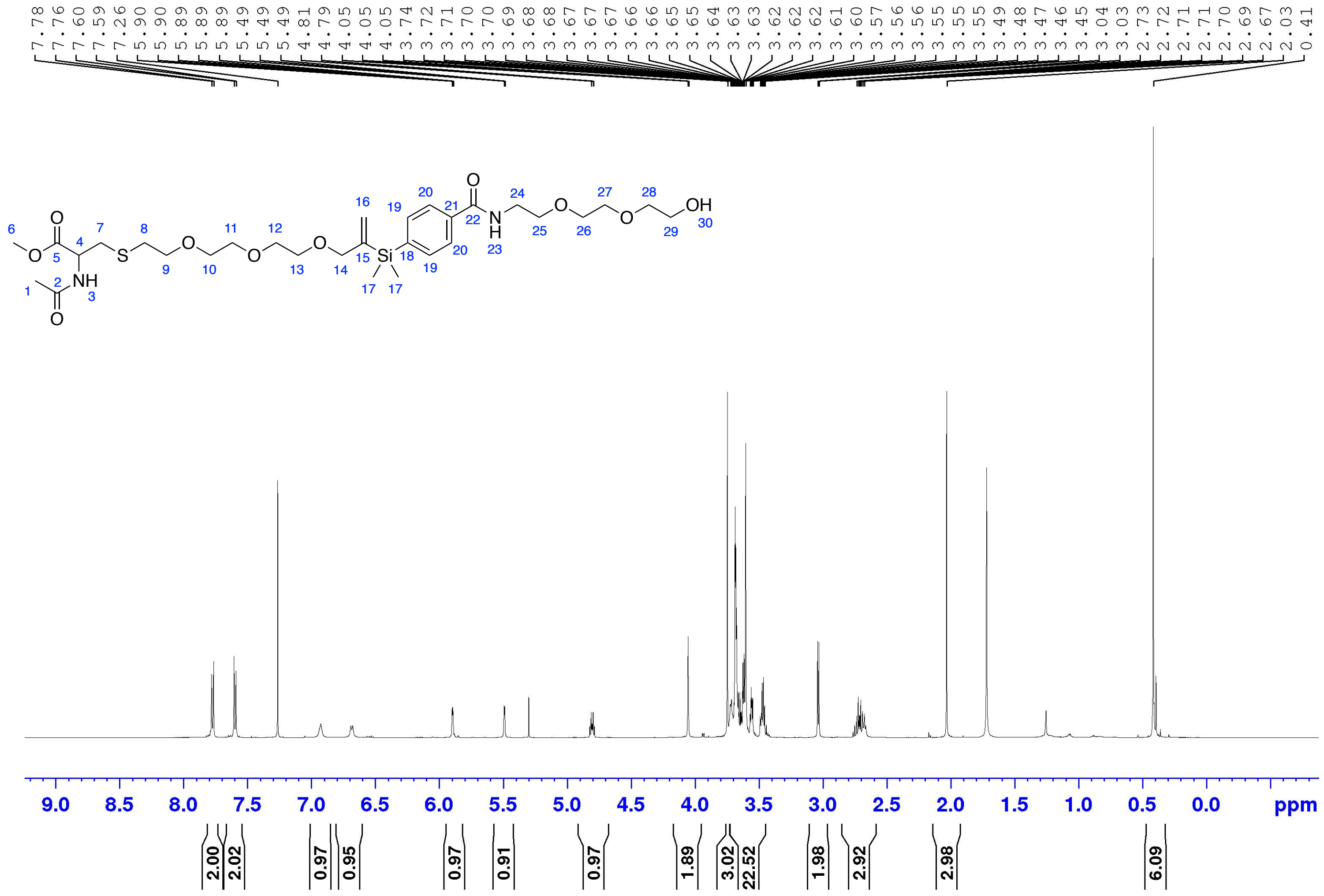

Methyl 16-acetamido-2-(4-((2-(2-(2-hydroxyethoxy)ethoxy)ethyl)carbamoyl)phenyl)-2-methyl-3-methylene-5,8,11-trioxa-14-thia-2-sila-heptadecan-17-oate (**22**), ^{13}C NMR ($CDCl_3$, 126 MHz)

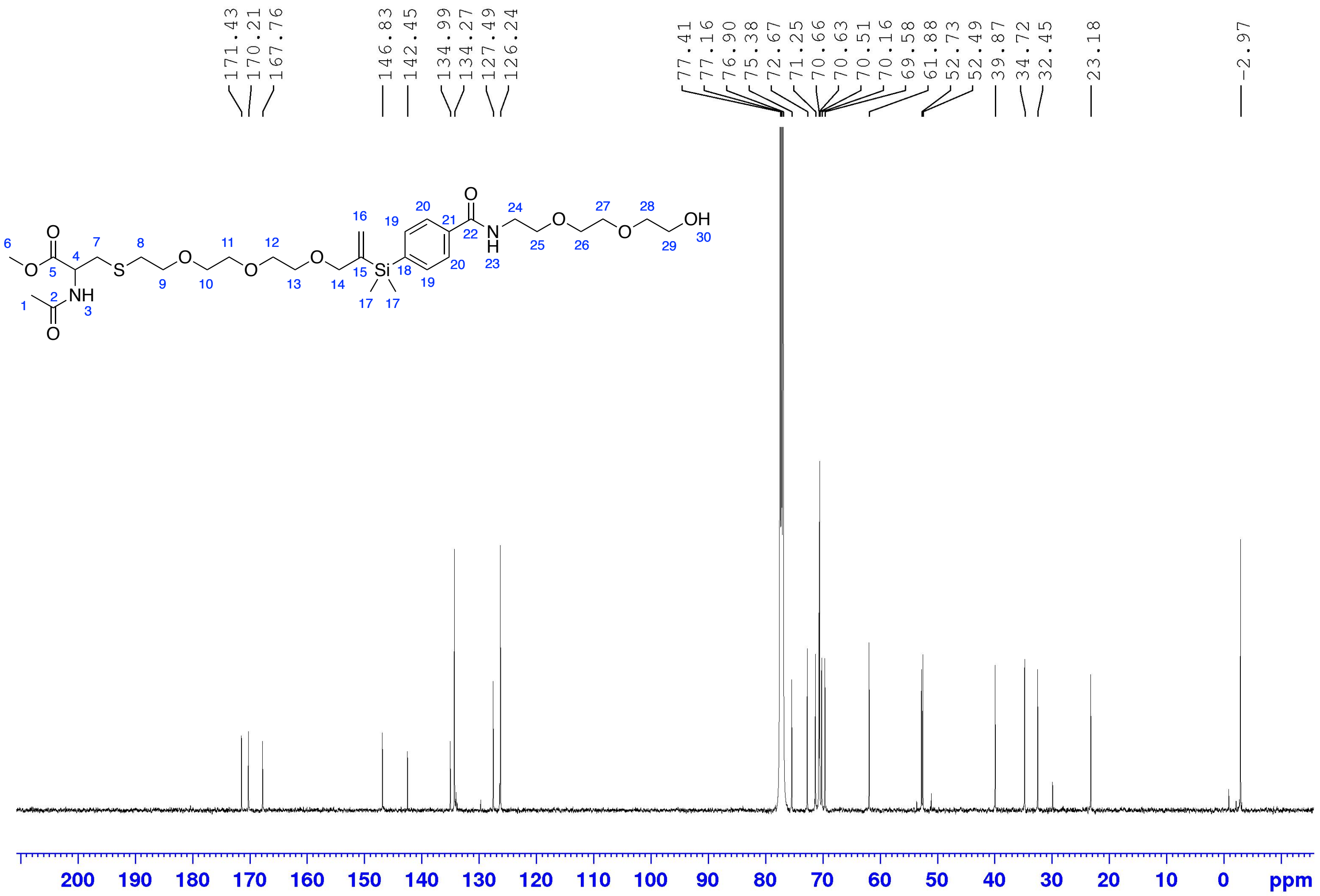

N^2-(Fmoc)-N^6-((prop-2-yn-1-yloxy)carbonyl)-L-Lys (**X**), ^{1}H NMR (DMSO-d_6, 400 MHz)

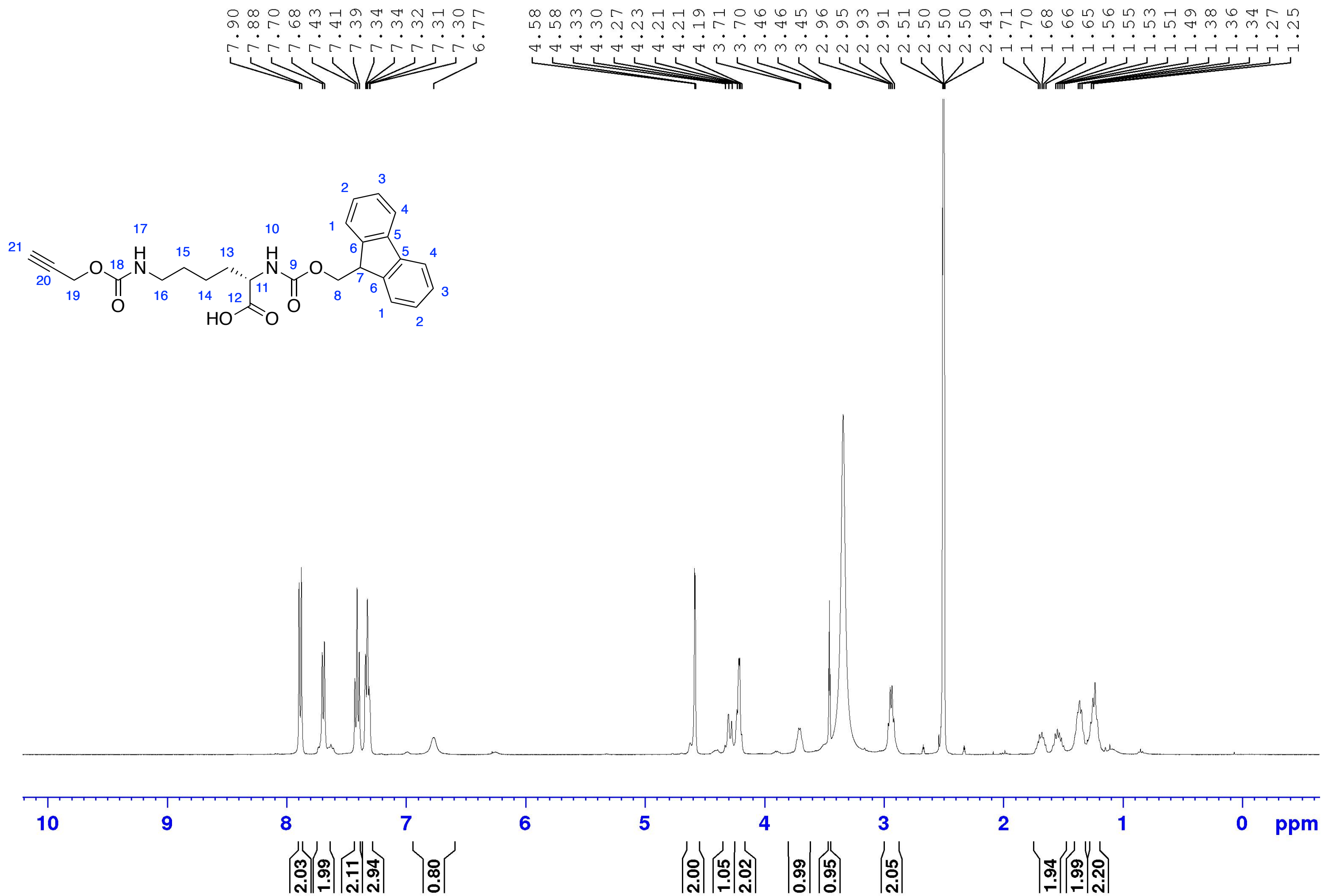

N^2-(Fmoc)-N^6-((prop-2-yn-1-yloxy)carbonyl)-L-Lys (**X**), ^{13}C NMR (DMSO-d_6, 126 MHz)

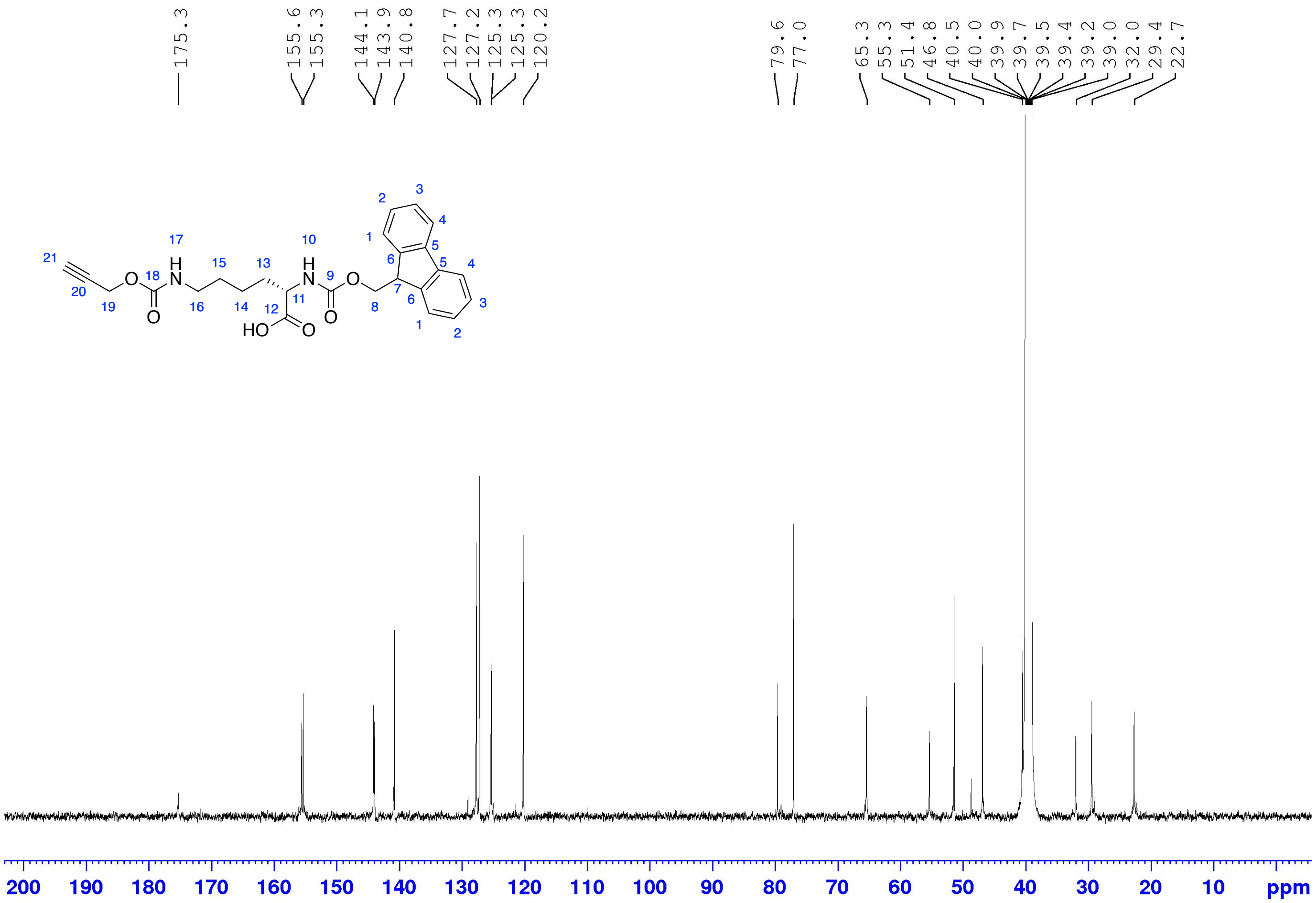

3,4-Dimethoxy-*N*-(2-methyl-3-methylene-2-phenyl-5,8,11-trioxa-2-silatridecan-13-yl) benzamide (**31**), ^{1}H NMR ($CDCl_3$, 500 MHz)

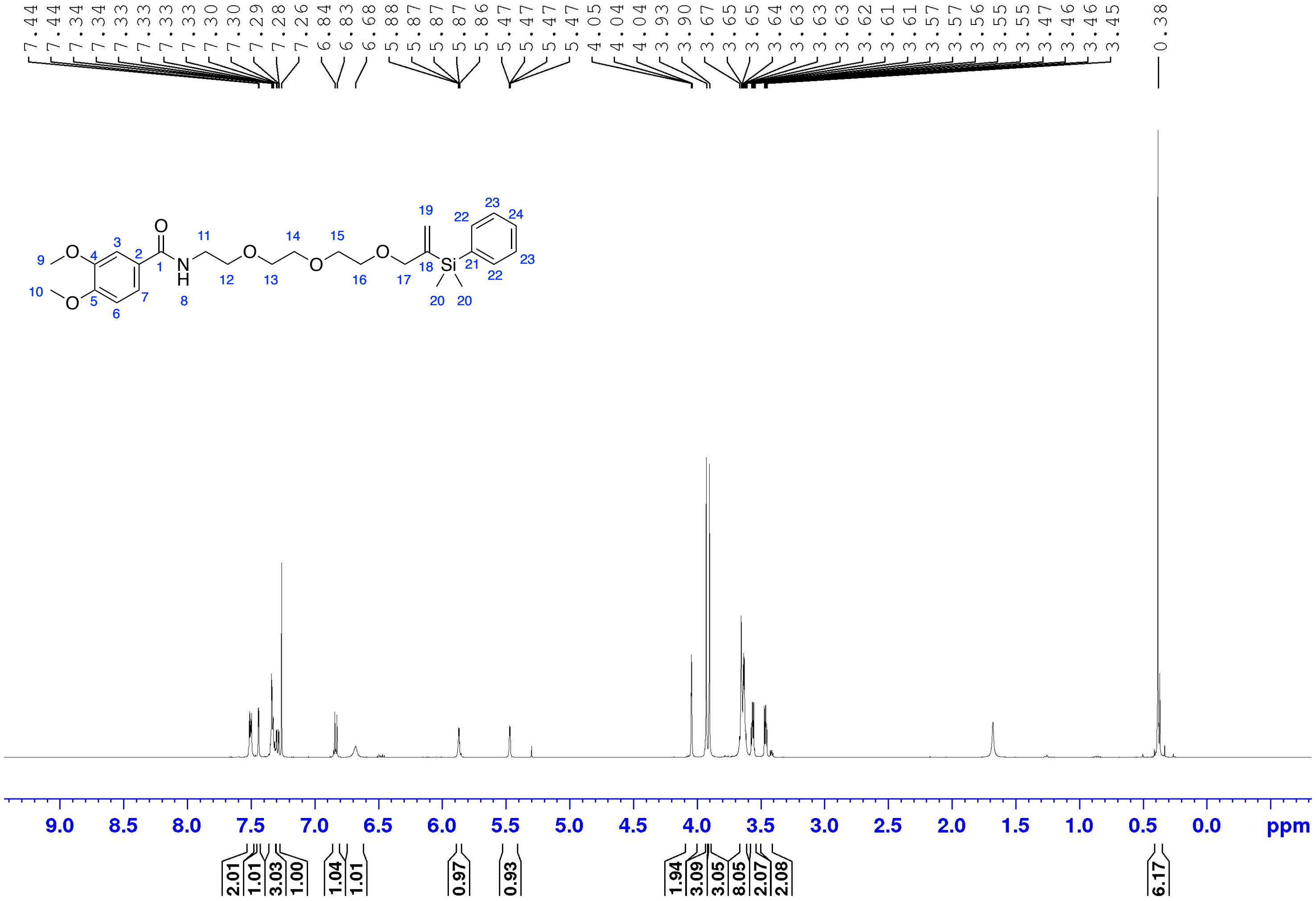

3,4-Dimethoxy-*N*-(2-methyl-3-methylene-2-phenyl-5,8,11-trioxa-2-silatridecan-13-yl) benzamide (**31**), ^{13}C NMR ($CDCl_3$, 126 MHz)

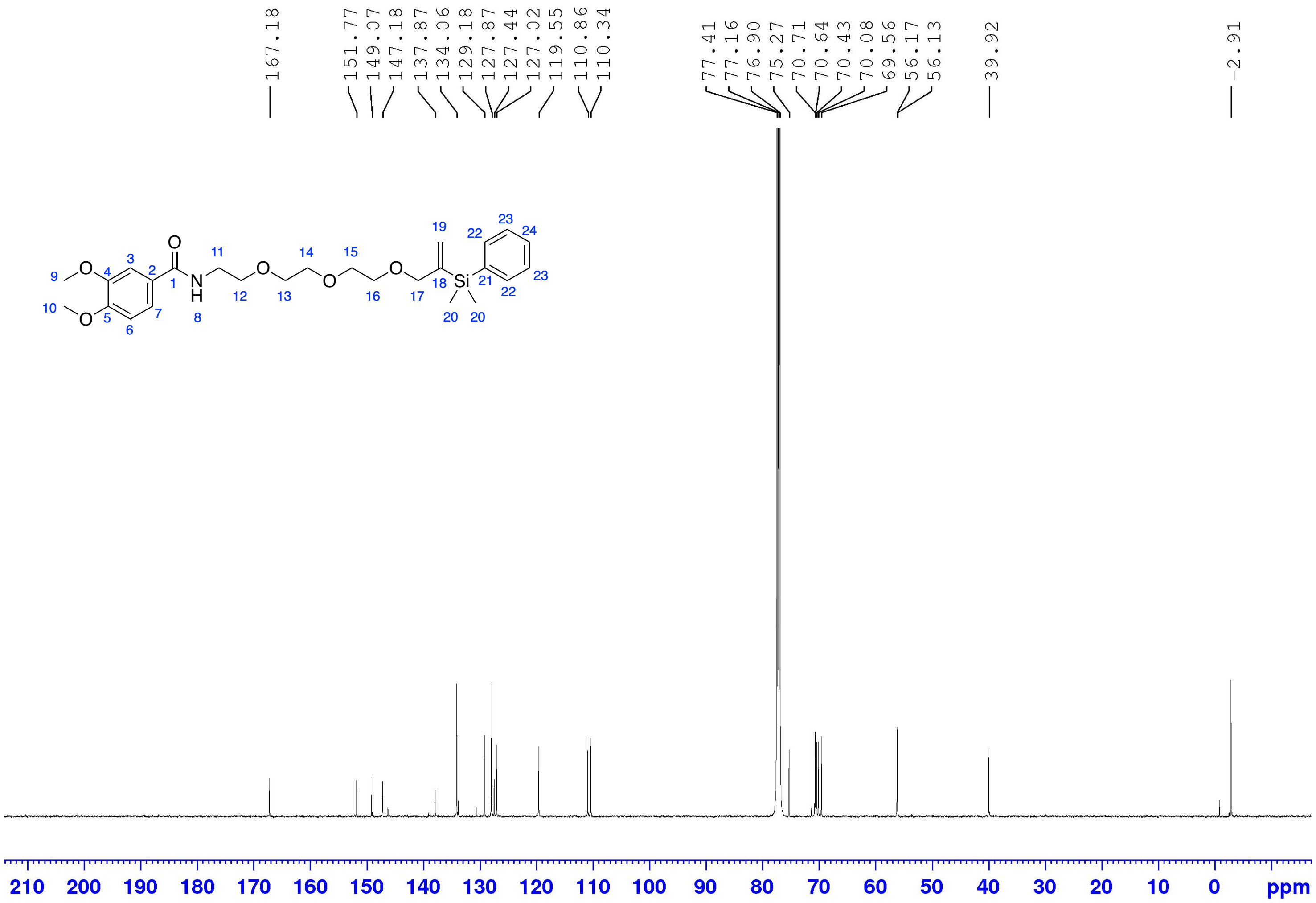

(*E*)-2-(2-(1-(2,4-Dimethoxyphenyl)ethylidene)hydrazinyl)benzoic acid (**32**), ^{1}H NMR (DMSO-d_6, 500 MHz)

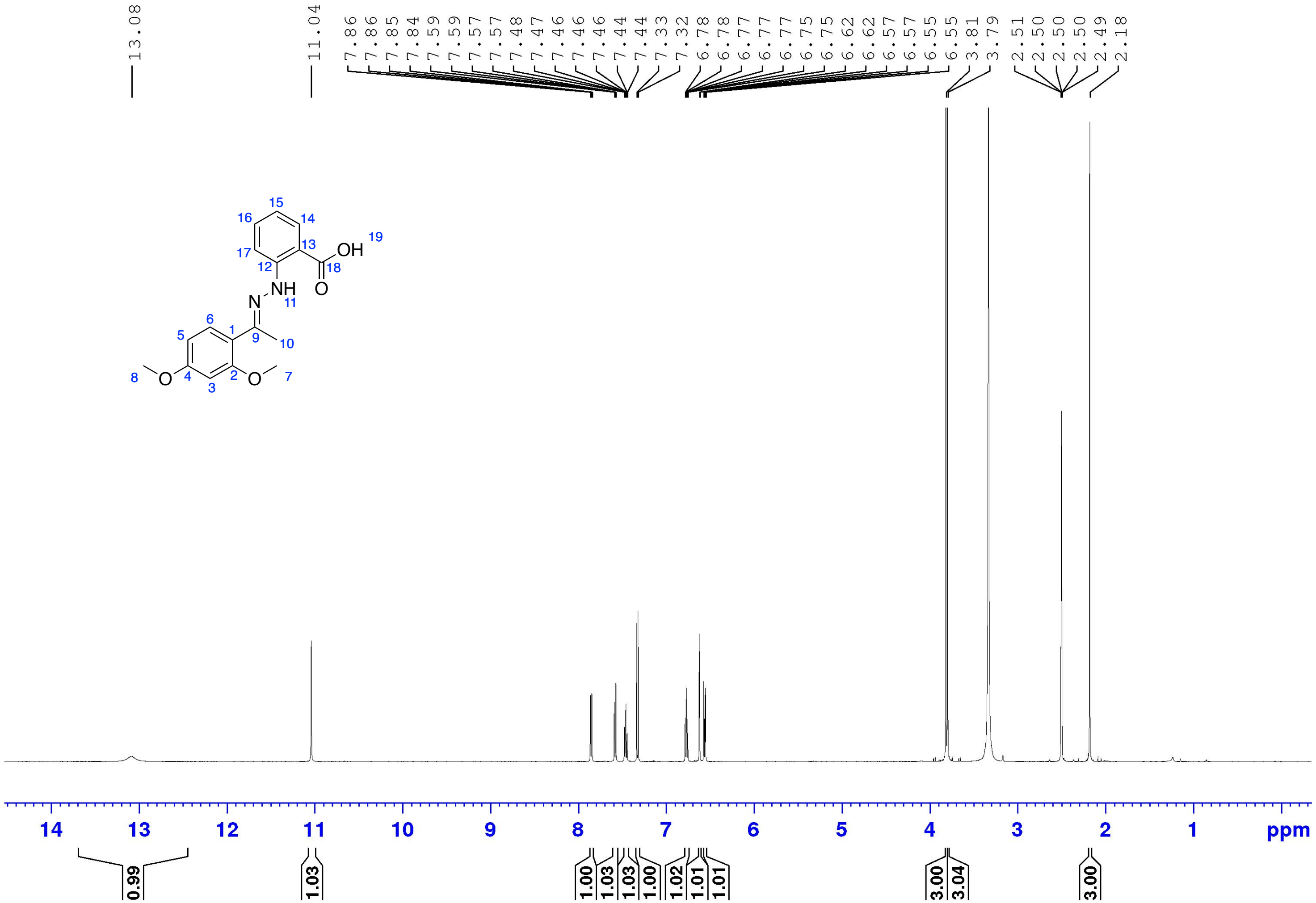

(*E*)-2-(2-(1-(2,4-Dimethoxyphenyl)ethylidene)hydrazinyl)benzoic acid (**32**), ^{13}C NMR (DMSO-d_6, 126 MHz)

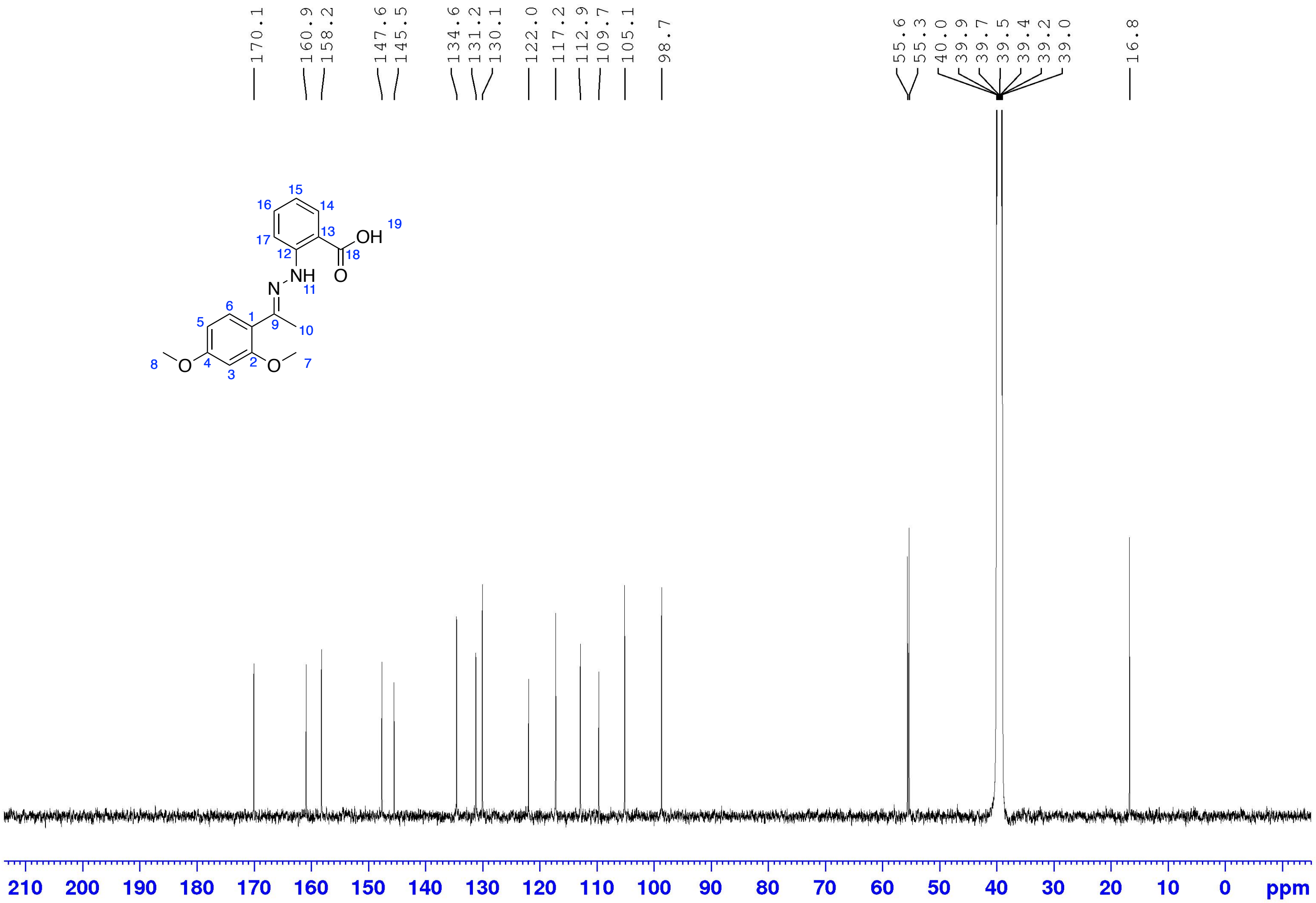

N-(2-(2-(2-(Allyloxy)ethoxy)ethoxy)ethyl)-3,4-dimethoxybenzamide (**35**), ^{1}H NMR ($CDCl_3$, 500 MHz)

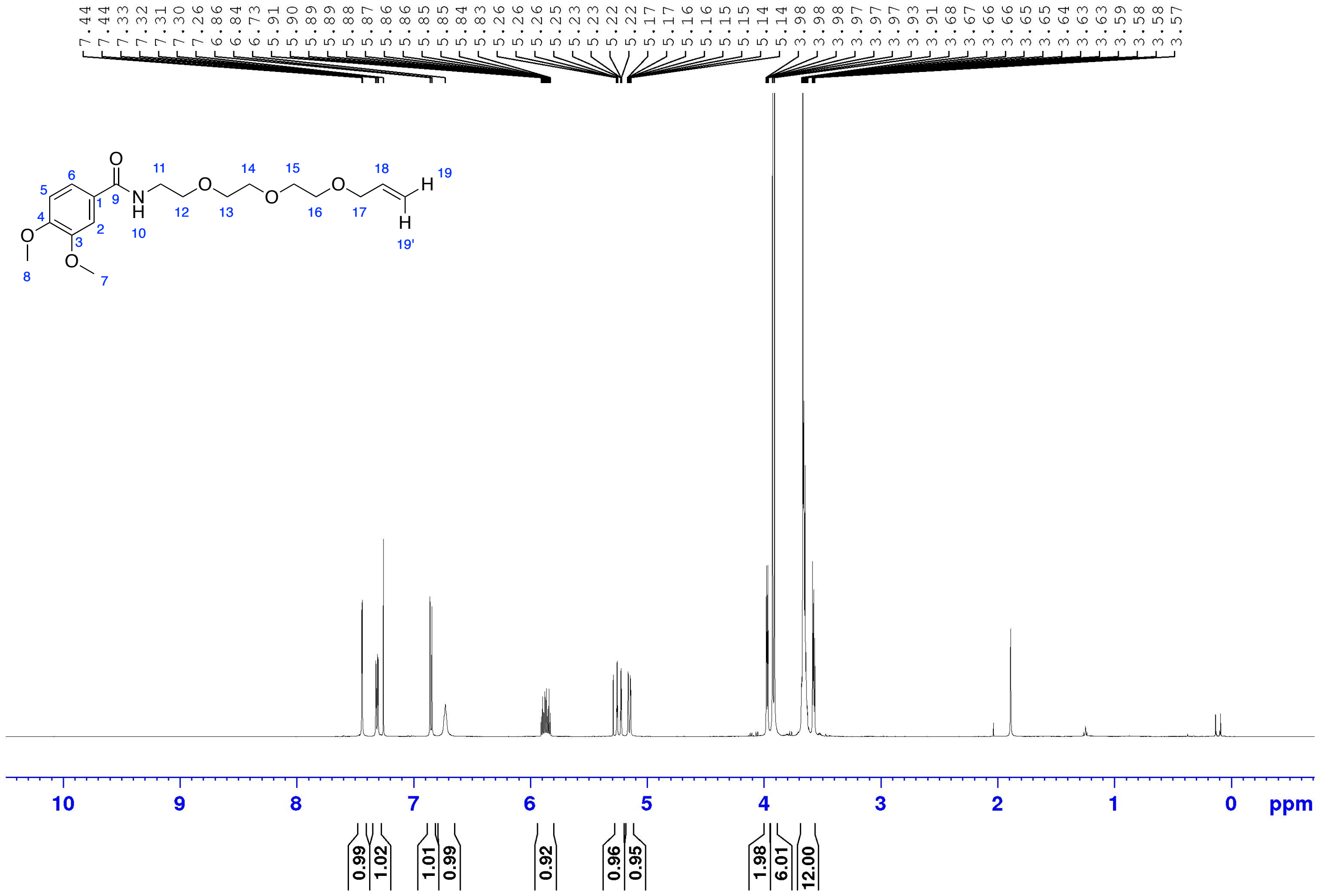

N-(2-(2-(2-(Allyloxy)ethoxy)ethoxy)ethyl)-3,4-dimethoxybenzamide (**35**), ^{13}C NMR ($CDCl_3$, 126 MHz)

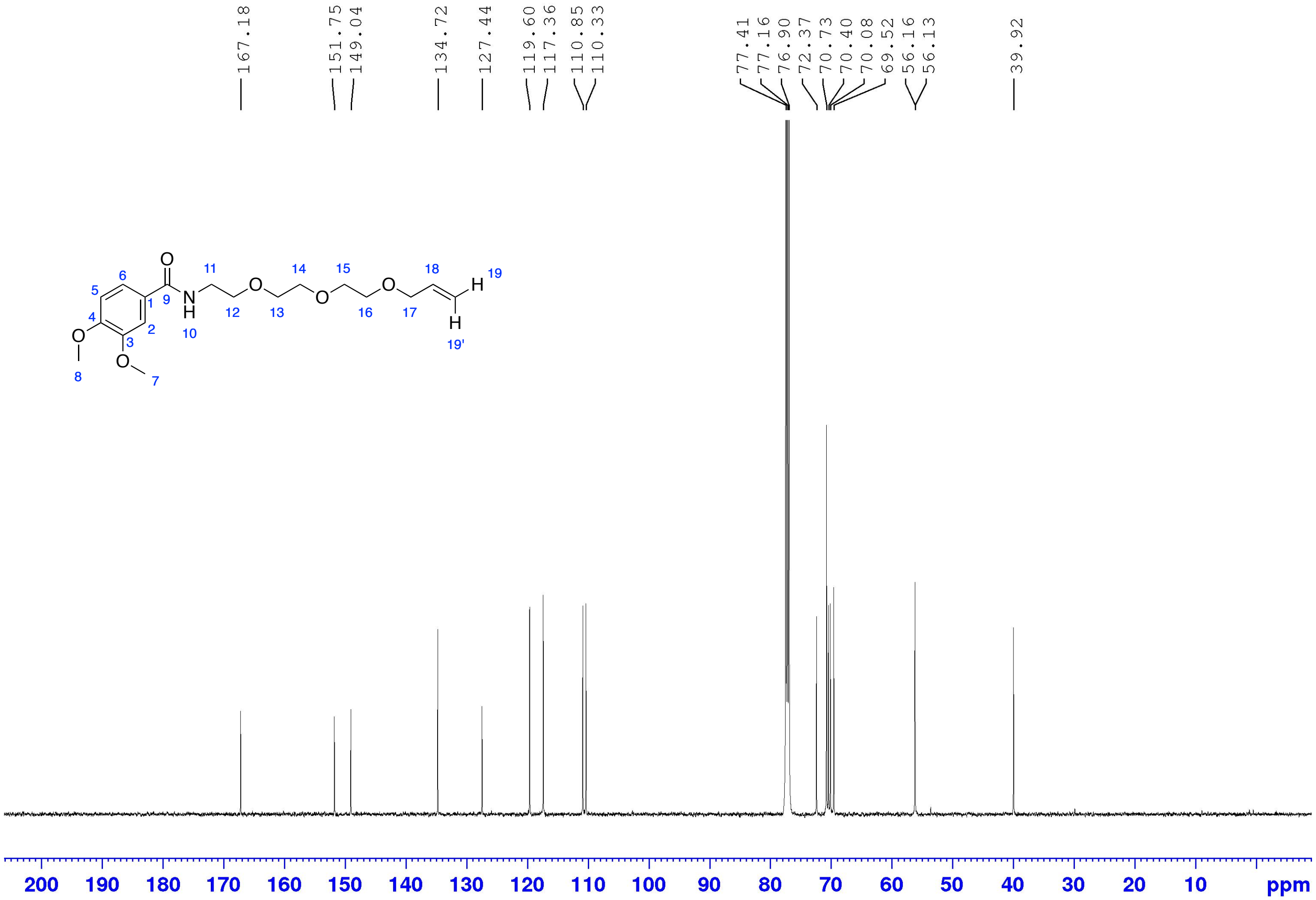

N-(3-((Benzylthio)methyl)-2-methyl-2-phenyl-5,8,11-trioxa-2-silatridecan-13-yl)-3,4-dimethoxybenzamide (**34**), ^{1}H NMR ($CDCl_3$, 700 MHz)

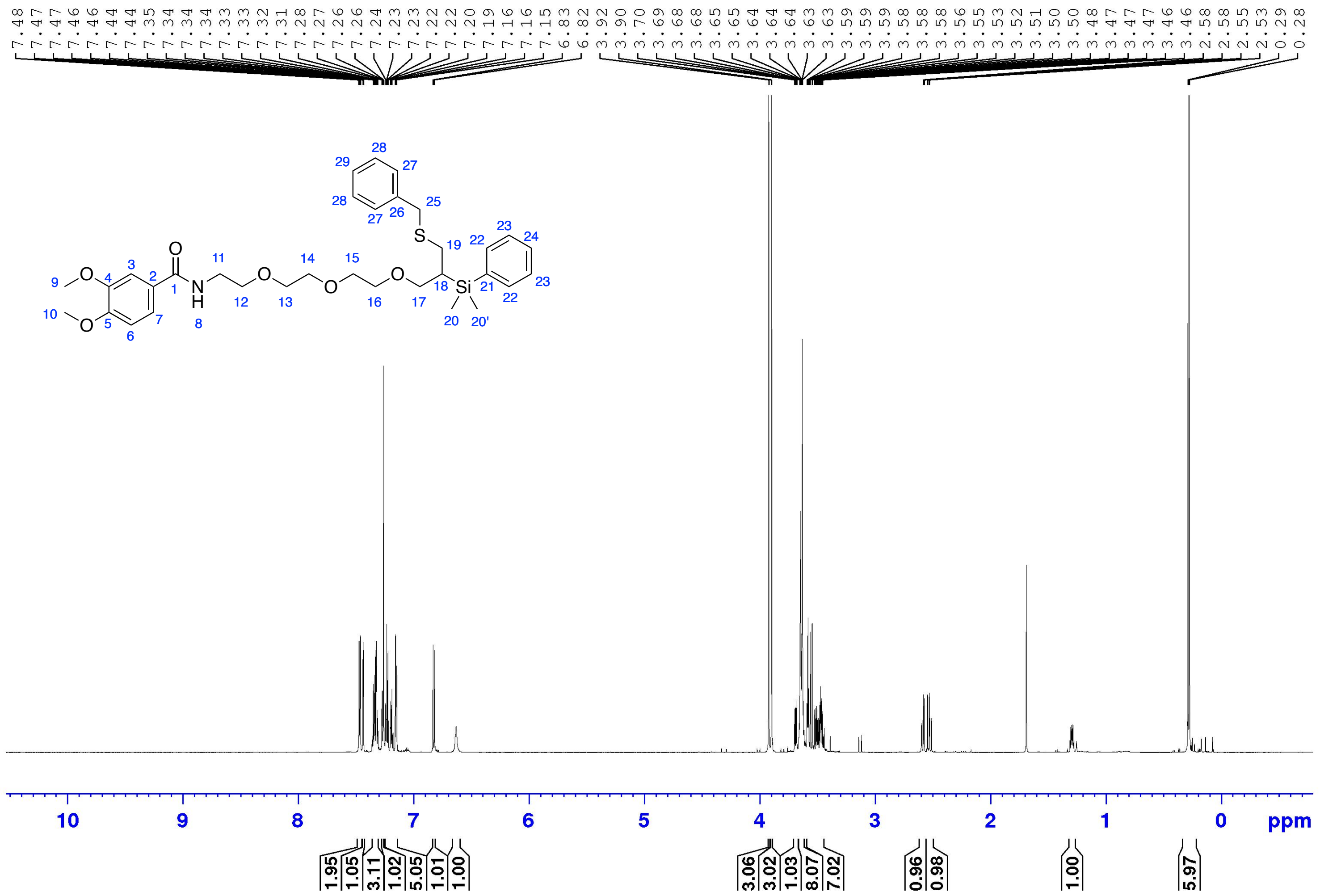

N-(3-((Benzylthio)methyl)-2-methyl-2-phenyl-5,8,11-trioxa-2-silatridecan-13-yl)-3,4-dimethoxybenzamide (**34**), ^{13}C NMR ($CDCl_3$, 176 MHz)

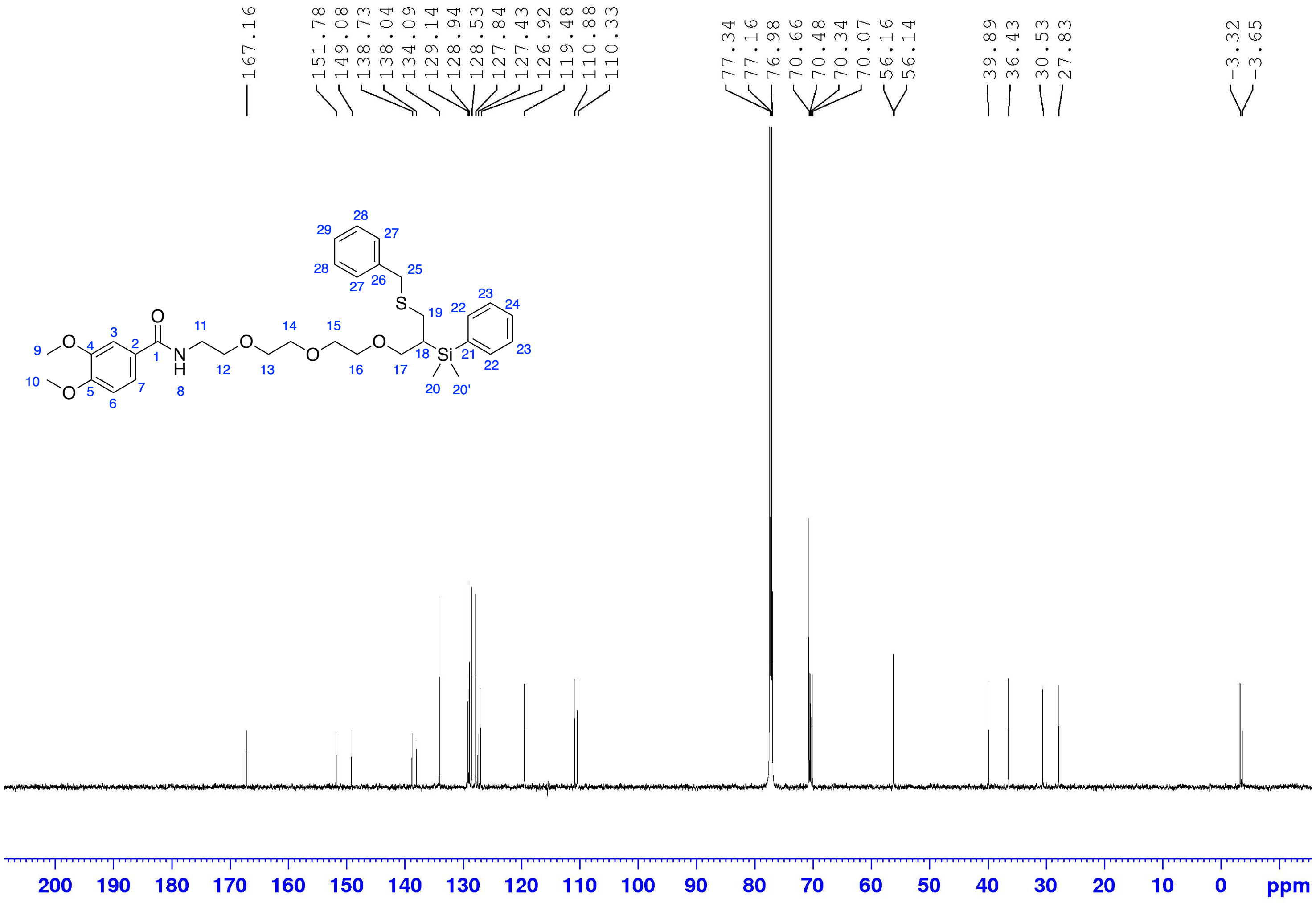

Supplement: Supplementary file 1 [file SC-008-C6SC05313K-s001.pdf]
